# Lysine Propionylation is a Widespread Post-Translational Modification Involved in Regulation of Photosynthesis and Metabolism in Cyanobacteria

**DOI:** 10.3390/ijms20194792

**Published:** 2019-09-26

**Authors:** Mingkun Yang, Hui Huang, Feng Ge

**Affiliations:** 1 Key Laboratory of Algal Biology, Institute of Hydrobiology, Chinese Academy of Sciences, Wuhan 430072, China; 2 University of Chinese Academy of Sciences, Beijing 100039, China

**Keywords:** lysine propionylation, post-translational modification, *Synechocystis* sp. PCC 6803, cyanobacteria, fructose-1,6-bisphosphatase, PsaD, proteomic analysis

## Abstract

Lysine propionylation is a reversible and widely distributed post-translational modification that is known to play a regulatory role in both eukaryotes and prokaryotes. However, the extent and function of lysine propionylation in photosynthetic organisms remains unclear. Cyanobacteria are the most ancient group of Gram-negative bacteria capable of oxygenic photosynthesis, and are of great importance to global carbon and nitrogen cycles. Here, we carried out a systematic study of lysine propionylaiton in cyanobacteria where we used *Synechocystis* sp. PCC 6803 (*Synechocystis*) as a model. Combining high-affinity anti-propionyllysine pan antibodies with high-accuracy mass spectrometry (MS) analysis, we identified 111 unique lysine propionylation sites on 69 proteins in *Synechocystis*. Further bioinformatic analysis showed that a large fraction of the propionylated proteins were involved in photosynthesis and metabolism. The functional significance of lysine propionylation on the enzymatic activity of fructose-1,6-bisphosphatase (FbpI) was studied by site-directed mutagenesis and biochemical studies. Further functional studies revealed that the propionylation level of subunit II of photosystem I (PsaD) was obviously increased after high light (HL) treatment, suggesting that propionylation may be involved in high light adaption in *Synechocystis*. Thus, our findings provide novel insights into the range of functions regulated by propionylation and reveal that reversible propionylation is a functional modification with the potential to regulate photosynthesis and carbon metabolism in *Synechocystis*, as well as in other photosynthetic organisms.

## 1. Introduction

Lysine propionylation is a reversible and widely distributed post-translational modification (PTM) in which a propionyl group (CH_3_-CH_2_-CO-) is added to the ɛ-amino group of lysine residue on a protein moiety [1,2]. Pioneering work by Zhao et al. opened new avenues to identify lysine propionylation events in both histone and non-histone protein substrates around ten years ago [1,2]. Comprehensive studies have focused on identifying the propionylated proteins in *Thermus thermophilus* [3], *Mycobacterium tuberculosis* [4], yeast [5], mammalian cells [1,6], and mouse liver mitochondria [7], as well as the enzymes that are responsible for adding and removing this modification, including p300 [1,2,6], CREB-binding protein [1,2], Sirt1 [1], Sirt2 [6,8], Sirt3 [8], Pat [8], AcuA [8], and CobB [8]. These studies have demonstrated that lysine propionylation occurs in a diverse range of proteins and exerts influence on a wide range of biological functions. In particular, lysine propionylation contributes to the overall metabolic regulatory network and cellular stress responses in mice [7]. It can regulate the enzyme activities of propionyl-CoA synthetase in *Salmonella enterica* [8] and isocitrate lyase (Icl) in *M. tuberculosis* [4]. These findings strongly suggest that lysine propionylation is likely to be one of the mechanisms that regulates cellular metabolism and responds to stress conditions in both bacteria and mammals [3,7].

Cyanobacteria are the most ancient group of Gram-negative bacteria capable of oxygenic photosynthesis that exhibit extraordinary diversity in terms of morphology and cell activity [9]. They are of global importance for CO_2_ assimilation, O_2_ production, and N_2_ fixation [10]. Cyanobacteria are ancient life forms believed to be the progenitors of higher plant chloroplasts [11]. The photosynthetic activity of cyanobacteria is responsible for approximately half of marine primary production and a quarter of global primary production on Earth [12]. Consequently, there are established model organisms for the study of their photosynthetic mechanisms. The unicellular cyanobacterium *Synechocystis* sp. PCC 6803 (henceforth referred to as *Synechocystis*) is the first phototrophic organism to undergo full genome sequencing, and has been universally used as a model cyanobacterium in studies about the photosynthetic process and biofuel production [13,14,15]. Photosynthesis and carbon metabolism are two interrelated processes in photosynthetic organisms [16,17,18], and complicated mechanisms have evolved to coordinate and regulate multiple pathways involved in the photosynthetic processes and cellular metabolism in cyanobacteria [18,19,20]. It is crucial to understand the regulatory mechanism between photosynthetic pathways and metabolic processes systematically. Lysine propionylation has been reported to be a functional PTM that contributes to the overall metabolic regulatory network and controls the activities of a set of metabolic enzymes in bacteria [1,3,8]; therefore, we hypothesized that lysine propionylation may be involved in the regulation of photosynthesis and carbon metabolism in cyanobacteria. However, to the best of our knowledge, no propionylated proteins have been reported previously in any photosynthetic organism, presenting a major obstacle to understanding the functions of lysine propionylation in cyanobacteria. 

To obtain a comprehensive understanding of lysine propionylation in cyanobacteria, a systematic study of propionylation events was performed in *Synechocystis*. Combining high-affinity anti-propionyllysine pan antibodies with high-accuracy mass spectrometry (MS) analysis and bioinformatics tools, we identified 111 unique lysine propionylation sites on 69 proteins in *Synechocystis*, out of which 23 propionylated proteins were involved in photosynthesis and respiration, including photosystem I/II subunits, phycocyanin subunits, allophycocyanin subunits, and carbon fixation pathways. Our results reveal the widest variety of propionylated proteins in photosynthetic organisms presently available, and offer insights into the functions related to propionylation in cyanobacteria. The functional significance of lysine propionylation on the enzymatic activity of fructose-1,6-bisphosphatase (Fbp) was determined by site-specific mutagenesis and biochemical studies. Further functional studies showed that the propionylation level of subunit II of photosystem I (PsaD) was significantly upregulated after high light (HL) treatment. Thus, our findings reveal novel insights into the regulatory functions of propionylation, suggesting that lysine propionylation is a functional modification with the potential to impact photosynthesis and carbon metabolism.

## 2. Results

### 2.1. Identification of Lysine Propionylated Proteins in Synechocystis

**Figure 1:**
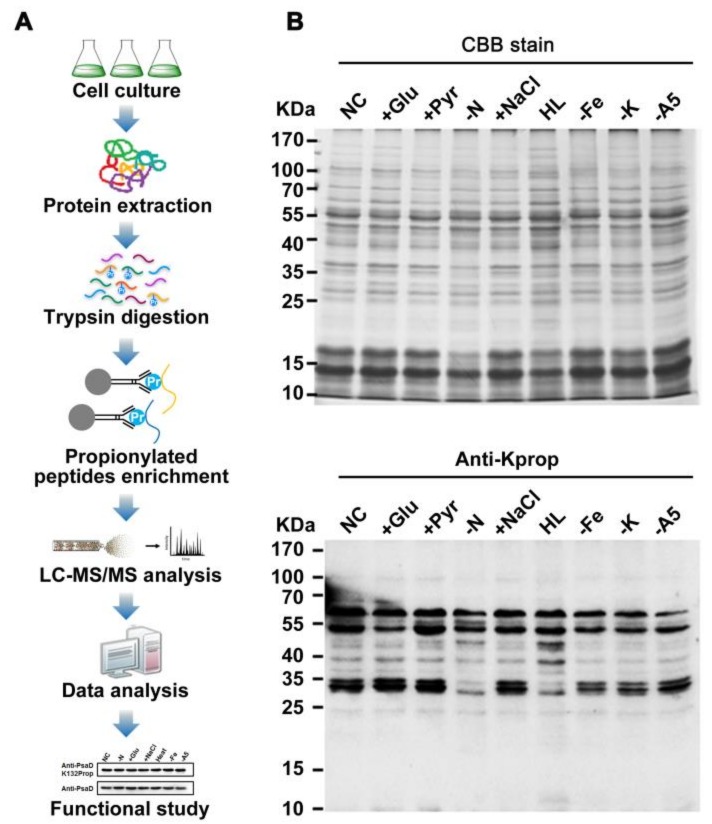
Profiling lysine propionylation in *Synechocystis* sp. PCC 6803. (**A**) Flowchart illustrating the experimental procedure for lysine propionylation analysis. (**B**) Profiling of lysine propionylation in *Synechocystis* under various stresses. Total proteins (20 µg) were extracted from the cells cultured under glucose treatment (+Glu), sodium pyruvate (+Pyr), nitrogen deficiency (−N), sodium chloride (+NaCl), high light (HL) conditions, iron deficiency (−Fe), potassium deficiency (−K), A5 deficiency (−A5), and normal conditions (NC).

To assess the possible role of protein propionylation in photosynthetic organisms, we surveyed whole cell lysates from *Synechocystis* for protein propionylation. As shown in Figure 1B, propionylation signals were abundant and likely to be changed for several protein bands in the 10–55 kDa mass range in *Synechocystis* under different stress conditions. These results implied that lysine propionylation is a widespread protein modification and is likely to be involved in stress responses in *Synechocystis*.

On the basis of these observations, we characterized the in vivo propionylation event in the model cyanobacterium by combining immunoaffinity enrichment and an MS-based high-throughput proteomic approach (Figure 1A). A total of 115 unique propionylated peptides encompassing 111 propionylation sites from 68 proteins were identified with a false discovery rate (FDR) below 1% for modified peptide in this study. All spectra containing propionylation were manually inspected as previously described [21,22]. Detailed information about all the identified propionylated peptides is listed in Table S1. All the raw data files were submitted to the public PeptideAtlas repository and can be accessed with the identifier PASS00818 (http://www.peptideatlas.org/PASS/PASS00818). The annotated spectra of all propionylated peptides were also deposited in PeptideAtlas and are supplied as the Supplementary Materials. In addition, the overall absolute peptide mass accuracy was 0.5249 parts per million (ppm) (standard deviation = 0.4501 ppm) and the average peptide score was 95.79, indicating the high accuracy and reliability of modified peptide data from MS (Figure S1A). Moreover, we also calculated the number of identified modification sites per protein and approximately 39% of identified proteins were modified by multiple propionylation sites (Figure S1B).

### 2.2. Functional Annotation of Propionylated Proteins

To address the functional distribution of identified proteins, we performed a Gene Ontology (GO) functional annotation of all identified proteins according to their biological processes, molecular functions, and cellular compartments (Figure S2A and Table S2). Among the identified proteins, the major biological processes of the propionylated proteins included cellular biosynthetic process (21%), organic substance biosynthetic process (20.99%), and photosynthesis (17.28%), indicating that propionylation may play an important role in cellular metabolism. Accordingly, most propionylated proteins were assigned to cytoplasm (31 proteins), followed by the cytoplasmic membrane (14 proteins), periplasm (2 proteins) and extracellular matrix (1 protein). However, the subcellular location of remaining propionylated proteins (30.4%) was not predicted. From a molecular function perspective, the propionylated proteins were mostly involved in anion binding (38.24%), oxidoreductase activity (20.59%), hydrolase activity (20.59%), and ribonucleotide binding (20.59%). We also predicted lipoproteins and integral membrane proteins, as previous described [23,24,25]. Among the identified propionylated proteins, nine proteins were detected as integral membrane proteins, with five of the integral membrane proteins having only one transmembrane helix. For the 60 non-integral membrane proteins with propionylation sites, two were grouped to lipoproteins.

**Figure 2:**
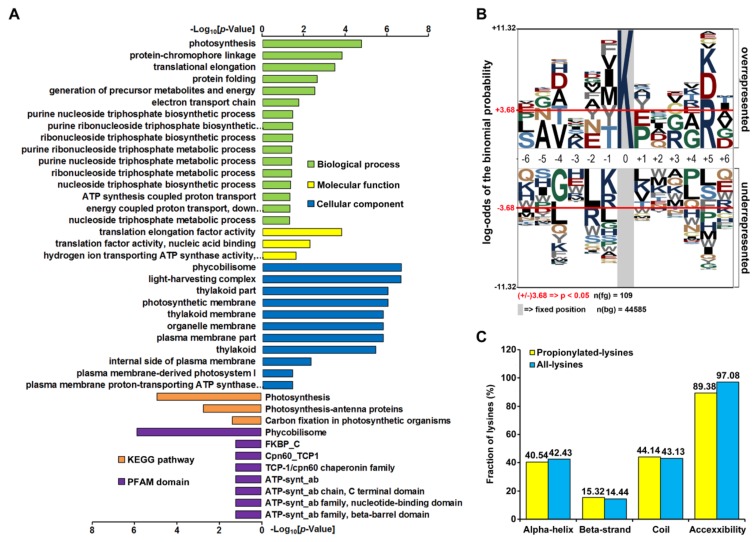
Enrichment analysis of propionylated proteins and bioinformatics analysis of propionylation sites. (**A**) Histogram representations of the enrichment of propionylated proteins according to their biological processes, molecular functions, cellular components, pathways, and protein family (PFAM) domains. The enrichment of GO categories, pathway, and domain were performed using DAVID bioinformatics tools (p < 0.05). (**B**) Sequence properties of lysine propionylation sites. Benjamini-corrected statistical significance values (adjusted *p* < 0.05) were used to control the family-wide false discovery rate. (**C**) Probabilities of localization for different secondary structures (α-helix, beta-strand, and coil) and protein surface of identified propionylation sites.

To gain further insight into the biological functions of propionylated proteins, we conducted GO, Kyoto Encyclopedia of Genes and Genomes (KEGG) pathway, and protein domain enrichment analyses (Figure 2A and Table S3). The GO enrichment analysis of biological processes showed that the propionylated proteins were mostly enriched in photosynthesis (*p* = 6.50 × 10^−6^). The major molecular functions for propionylated proteins were translation elongation factor activity (*p* = 6.97 × 10^−5^), translation factor activity and nucleic acid binding (p = 3.30 × 10^−3^), and hydrogen ion-transporting ATP synthase activity and rotational mechanisms (*p* = 1.77 × 10^−2^). In cyanobacterial cells, translation of the psbA2 gene, which encodes D1 protein of photosystem II (PSII), is regulated at the elongation step [26,27] and is activated when reducing equivalents are derived from photosystem I [28]. Thus, photosynthesis and protein translation activity are functioned in an interactive and cooperative way [29]. In this scenario, lysine propionylation may be one of the mechanisms to coordinate both processes in cyanobacteria. Future studies should be directed toward a better understanding of the mechanism and physiological significance of propionylation in the regulation of photosynthesis and protein translation.

### 2.3. Analysis of Lysine Propionylation Sites

We further assessed the position-specific amino acid frequencies of the flanking sequences of propionylation sites. The 12 amino acid residues surrounding the lysine propionylation sites were compared with the residues around nonpropionylated lysines from the *Synechocystis* proteome (Figure 2B). We observed a significant preference of the polar basic residue (arginine) at the +5 position, while propionylation rarely occurred in the nonpolar hydrophobic residue leucine at the −2 position. As the other two amino acid residues (K and D) also showed a relatively high frequency at the +5 position, it was suggested that there may exist three possible site-specific propionylation motifs in *Synechocystis*, and KxxxxR is most likely to be the one. Further functional experiments are needed to confirm these possible site-specific motifs in *Synechocystis*.

Next, to elucidate the specific structural properties of propionylation sites, the local secondary structures surrounding propionylation sites were analyzed using NetSurfP software. Consistent with other lysine PTMs [30,31], the propionylated lysine residue was more frequently observed in secondary structure regions. In the present study, more than 55.8% of propionylated lysine residues were localized in the α-helix and β-strand in comparison to 43.1% in the coil region. However, the average relative side chain solvent accessibility of propionylated lysines (89.38%) was lower than that of all lysines (97.08%), as opposed to other lysine PTMs [30,31], indicating that lysine propionylation may have a different effect on modified proteins (Figure 2C and Table S4).

### 2.4. Functional Interaction Networks of Propionylated Proteins in Photosynthesis

**Figure 3:**
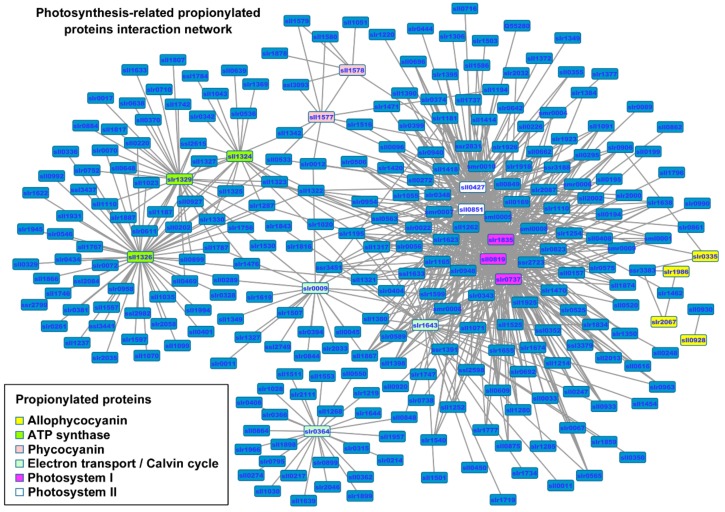
The complete interaction network of identified propionylated proteins associated with photosynthesis in *Synechocystis*. The propionylated proteins involved in photosynthesis are grouped using associated subcellular localizations and highlighted in different colors.

We next generated a protein interaction network of all identified proteins using the *Synechocystis* protein-protein interaction (PPI) database [32] to assess the physical and functional interactions within our datasets (Figure S3 and Table S5A). Based on this network, protein groups associated with propionylated proteins were characterized, and a large sub-interaction network was further constructed and visualized by Cytoscape. This interaction network consisted of 331 proteins, among which existed 17 propionylated photosynthetic proteins such as allophycocyanin (slr0335, ApcE; slr1986, ApcB; slr2067, ApcA; sll0928, ApcD), phycocyanin (sll1578, CpcA; sll1577, CpcB), ATP synthase (sll1324, AtpF; slr1329, AtpB; sll1326, AtpA), electron transport/Calvin cycle (slr0009, RbcL; slr1643, PetH; slr0364, Pgk), photosystem I (slr1835, PsaB; sll0819, PsaF; slr0737, PsaD), and photosystem II (sll0427, PsbO; sll0851, PsbC) (Figure 3 and Table S5B). As there is little functional information about these interactions, further studies were needed to verify the potential interactions. It is expected that such bioinformatic analysis will contribute to formulating testable hypotheses to explore the function roles of lysine propionylation in *Synechocystis*, and will be useful in selecting key proteins and possible mechanisms from these proteins.

### 2.5. Effects of Propionylation on Metabolic Enzyme Activity

**Figure 4:**
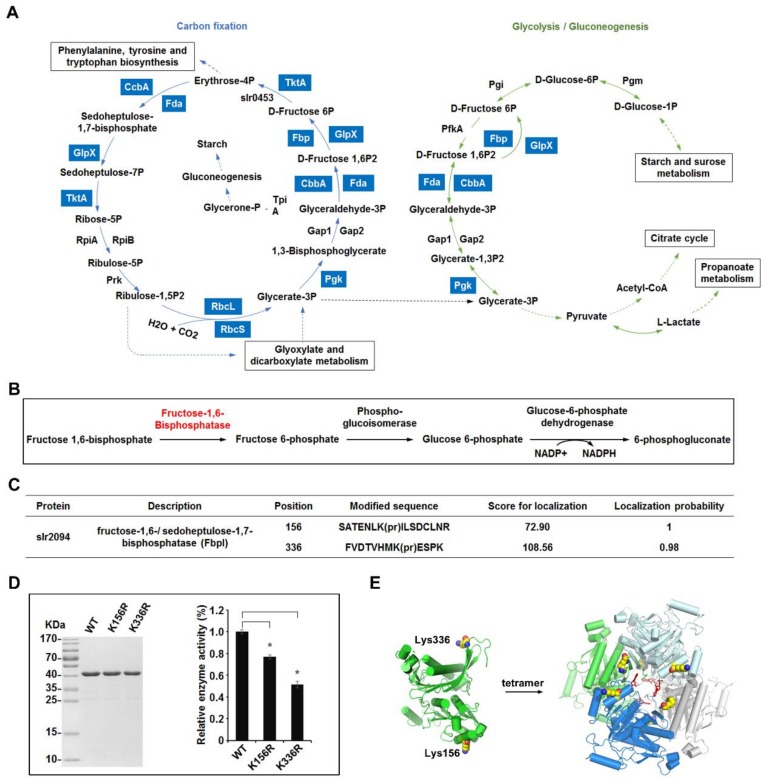
Overview of the lysine propionylation events involved in cellular metabolism in *Synechocystis*. (**A**) Working scheme to delineate the lysine propionylation events in carbon metabolism of *Synechocystis*. The identified propionylated proteins were highlighted in light blue. Solid arrows represent direct reactions and dotted arrows represent reactions with multiple steps. (**B**) Schematic illustration of the metabolic pathway catalyzed by fructose-1,6/sedoheptulose-1,7-bisphosphatase (FbpI). (**C**) Identification of the propionylation sites of FbpI. (**D**) Effects of propionylation sites on the enzyme activity of FbpI. The FbpI and its mutants were expressed and the relative activities were determined. Data are means ± SD from three independent assays. (**E**) The crystal structure of FbpI. Two propionylation sites and the structure of the stable tetramer are shown.

To determine the molecular pathways in which the propionylated proteins participate, all identified proteins were analyzed and mapped to KEGG pathways of *Synechocystis*. In accordance with previous studies on lysine propionylation [3], many enzymes involved in metabolic pathways were found to be propionylated in this work, such as enzymes involved in carbon fixation and glycolysis/gluconeogenesis pathways (Table S6 and Figure 4A). 

One protein of interest is bifunctional enzyme fructose-1,6/sedoheptulose-1,7-bisphosphatase (FbpI), encoded by *slr2094* (Figure 4B). FbpI is the only recognized enzyme in *Synechocystis* that converts fructose-1,6-biphosphate to fructose-6-biphosphate, a process that is connected with the Calvin cycle, the oxidative photosephosphate cycle, and gluconeogenesis in oxygenic photosynthetic organisms [33]. In this study, two reliable propionylation sites (Lys156 and Lys336) were observed in FbpI (Figure 4C) and the MS/MS spectra of these peptides, indicating the exact sites of their propionylation, are shown in Figure S4A. Multiple sequence alignment analyses revealed that the identified propionylation sites of FbpI were highly conserved in cyanobacteria, suggesting that these residues may be important for an evolutionarily conserved function of FbpI (Figure S4B). To further assess whether lysine propionylation at these positions (Lys156 and Lys336) would affect the enzymatic activity of FbpI, we cloned the *slr2094* gene and converted the modified residues at the two positions (Lys156 and Lys336) to arginine (R) to lock protein into a non-propionylated state, as previously described [30]. All mutations were verified by DNA sequencing and MS analysis (Figure S5). The enzymatic activity of FbpI (wild type) and its mutants were then measured. As shown in Figure 4D, in contrast to WT, the mutation of Lys156, Lys336 to R gave rise to a decrease of the enzymatic activity (*p* < 0.01), suggesting that propionylation may alter the enzymatic activity of FbpI. Additionally, based on the crystal structure of FbpI, we found that FbpI could form a stable tetramer and Lys156, Lys336 located in the functional α-helix domain and structured loop of FbpI, respectively [34,35,36,37,38] (Figure 4E). Because propionylation changes the charge status of lysine from +1 to 0 and adds a propionyl group to lysine, it is conceivable that propionylation at Lys156 and/or Lys336 is likely to alter the conformation and enzymatic activity of FbpI and further affect the cellular metabolic process in *Synechocystis*. Overall, our results suggest the involvement of lysine propionylation in the regulation of cellular metabolism in *Synechocystis*, though further in vivo studies are needed to confirm the effects of propionylation on the process of cellular metabolism.

### 2.6. Validation of Propionylated Proteins Involved in Photosynthesis

**Figure 5:**
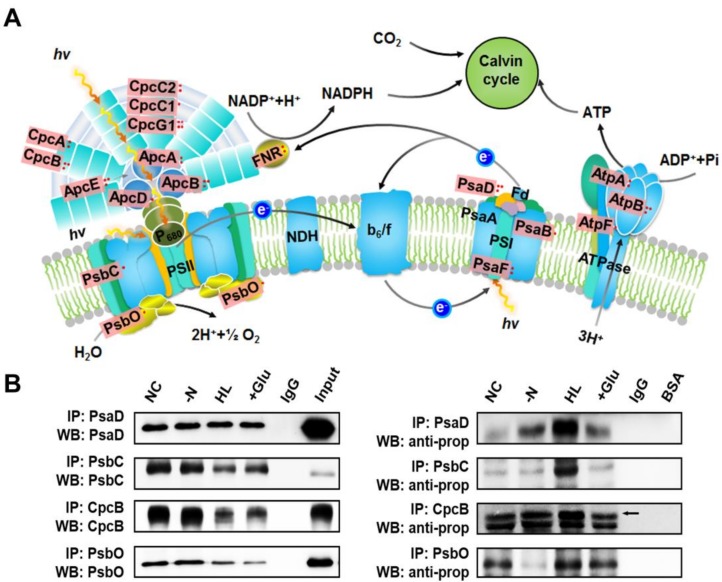
Overview of the lysine propionylation events involved in photosynthesis in *Synechocystis*. (**A**) Illustrations of propionylated proteins involved in the photosynthesis process. The identified propionylated proteins are highlighted in light red. (**B**) Verification of propionylated proteins associated with photosynthesis using immunoprecipitation and western blotting. NC: normal condition, -N: nitrogen deficiency, HL: high light condition, +Glu: glucose condition, Input: total cell lysate.

Unique to the photosynthetic organisms, a large proportion of the identified propionylated proteins were involved in photosynthesis. A total of 19 photosynthetic proteins were found to be propionylated (approximately 28% of the total) according to the mapped KEGG pathways, including a large proportion of the subunits of phycocyanin (CpcA, CpcB, CpcC1, CpcC2, and CpcG1), allophycocyanin (ApcA, ApcB, ApcD, and ApcE), PSII components (PsbC and PsbO), PSI components (PsaB, PsaD, and PsaF), and ATPase complex (AtpA, AtpB, and AtpF) (Figure 5A). Based on this observation, four identified photosynthetic proteins (PsaD, PsbC, CpcB and PsbO) were selected to further validate our MS findings. The presence of four propionylated proteins identified through our proteomic approach was validated by immunoprecipitation and western blotting (Figure 5B). The right part of Figure 5B shows the propionylation level of proteins under different conditions, and the corresponding protein levels of PsaD, PsbC, CpcB, and PsbO are displayed in the left part of Figure 5B. We found that the propionylation level of PsaD was increased in nitrogen deficiency, HL, and glucose conditions, especially under HL. For PsbC, the propionylation status was evidently elevated under HL. For CpcB, the propionylation level was significantly increased under HL and glucose conditions. For PsbO, the propionylation level was evidently affected under nitrogen deficiency and glucose conditions, and moderately changed under HL. The changes in propionylation statuses of these proteins indicate that propionylation may play regulatory role in response to different environmental stimuli. Further studies are required to reveal how reversible propionylation contributes to the response of *Synechocystis* to various environmental conditions.

### 2.7. Increased Propionylation Level of PsaD in High Light Condition

**Figure 6:**
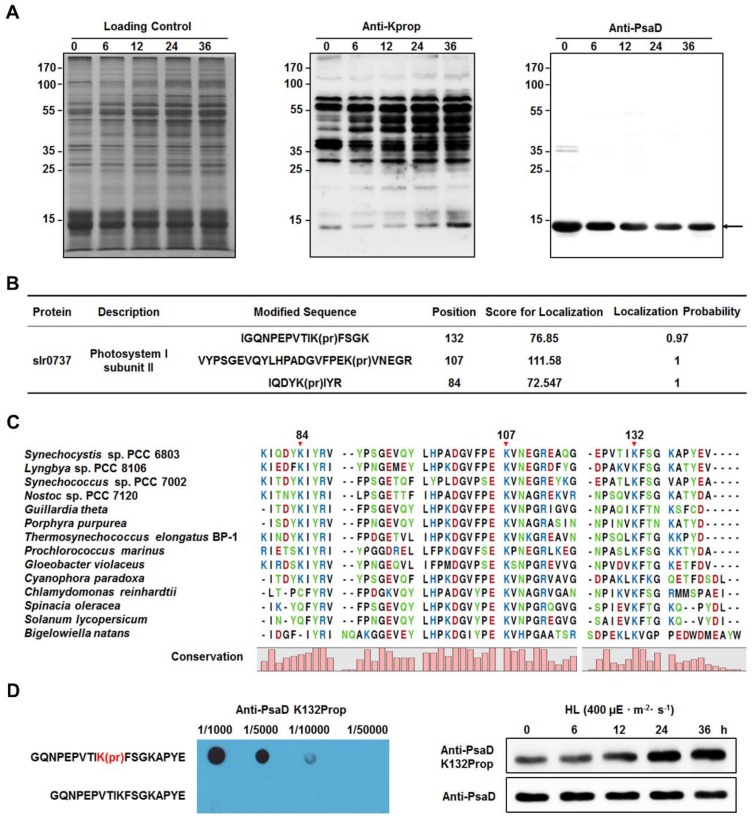
Identification of lysine propionylation in subunit II of photosystem I (PsaD). (**A**) Immunoblotting analysis of propionylation level of proteins from *Synechocystis* after exposure to high light intensity for 0, 6, 12, 24, and 36 h. Coomassie blue staining was used for the loading control and western blotting analysis was performed using anti-propionyllysine antibody and anti-PsaD antibody. (**B**) List of identified propionylation sites of PsaD. (**C**) Conservation analysis of PsaD from different species (cyanobacteria, algae, and plants). The conserved propionylation sites were marked by the red arrow. (**D**) Immunoblotting analysis of Lys132 propionylation level of PsaD in *Synechocystis* after exposure to high light intensity for 0, 6, 12, 24, and 36 h. The generated specific anti-Lys132 propionylation antibody was validated by dot blot analysis.

According to the results of immunoprecipitation and western blotting (Figure 5B), we further assessed the propionylation status of PsaD under different light intensities. By performing western blotting analysis on whole cell lysates with pan anti-propionyllysine antibody, we found that there was only one band (the bottom band in the middle part of Figure 6A) at the position corresponding to the molecular weight of PsaD. Considering that the propionylation level of PsaD was significantly increased under HL (Figure 5B), the band around 14 kDa may represent the signals from the propionated PsaD. By comparing with the corresponding immunoblot signals of PsaD, we can see that the propionylation levels of PsaD were likely to be increased after exposure to HL conditions for 0, 6, 12, 24, and 36 h. In this study, we identified three reliable propionylation sites (Lys84, Lys107, and Lys132) in PsaD (Figure 6B) and the MS/MS spectra of these peptides, which indicate their exact propionylation sites, are shown in Figure S6. Further analysis revealed that the lysines at positions 84 (in β-sheet), 107 (in coil), and 132 (in coil) in PsaD were highly conserved from cyanobacteria to plants (Figure 6C). To explore in more detail whether HL treatment might affect the propionylation levels of *Synechocystis* proteins in vivo, we further evaluated the propionylation status of Lys132 in PsaD with the generated site-specific propionylation antibody. As shown in Figure 6D, the result of dot blot analysis confirmed the specificity of the antibody and the propionylation level at Lys132 in PsaD was significantly increased during the long-term exposure to HL, suggesting that propionylation at this residue may be a novel regulatory mechanism in response to HL stress in *Synechocystis*. Moreover, western blotting analyses also showed that the propionylation level of PsaD at Lys132 were not affected by other stresses such as nitrogen deficiency, glucose addition, NaCl addition, heat treatment, iron deficiency, or copper deficiency (Figure S6D). As Lys132 in PsaD is important for the stable association of PsaD to the PSI core [39], our findings suggest that propionylation at this residue may play a role in the regulation of photosynthesis in *Synechocystis*.

## 3. Discussion

Recently, we analyzed the lysine acetylome of *Synechocystis* and revealed previously unappreciated roles of lysine acetylation in the regulation of photosynthesis [31]. In this study, we report the first systematic study of lysine propionylation in a cyanobacterium model. This is, to the best of our knowledge, the first global survey of lysine propionylation in a photosynthetic organism. The identified propionylaton sites were present in proteins involved in various pathways and processes in *Synechocystis.* In particular, proteins in major metabolic and photosynthetic pathways are modified by propionylation. Since lysine propionylation has emerged as an essential regulatory post-translational modification and may affect multiple metabolic processes, our study provides a large number of putative regulatory modification sites in *Synechocystis*. Through western blot analysis using an anti-propionyllysine antibody, we found that propionylation occurred in diverse photosynthetic organisms, and that the levels of this modification changed in response to different stress treatments in *Synechocystis*, suggesting propionylation is a prevalent and regulatory PTM in photosynthetic organisms. Therefore, understanding the physiological functions of these propionylation sites would certainly be useful to elucidate the regulation of photosynthetic processes and metabolic networks in cyanobacteria and other photosynthetic organisms.

In the present study, a large number of proteins involved in carbon metabolism were propionylated. For example, *slr2094* (FbpI), which encodes the fructose-1,6-biphosphatase (FBPase)/sedoheptulose-1,7-biphosphatase (SBPase) bifunctional enzyme, was found to be propionylated. FbpI can hydrolyze both fructose-1,6-bisphosphate (FBP) and sedoheptulose-1,7-bisphosphate (SBP) simultaneously in cyanobacteria [37]. It has been reported that FbpI is essential to sustain growth and has a vital role in photosynthetic carbon fixation and gluconeogenesis in cyanobacteria [35,36,40,41]. In addition, previous structure and biochemical analyses show that regulation of FbpI activity occurs at the structural level [37], and FbpI may adopt a unique regulatory mechanism that enables the enzyme to hydrolyze both FBP and SBP simultaneously [37]. It is conceivable that propionylation could have a more profound impact on protein structure and function compared to lysine acetylation, which regulates the activity of enzymes of central metabolic pathways in various eukaryotes and bacteria [31,42,43]. Consistent with this notion, our functional results suggest that lysine propionylation is likely to regulate FbpI activity. Thus, lysine propionylation could provide an additional layer to regulate the enzymatic activity of FbpI. 

**Figure 7:**
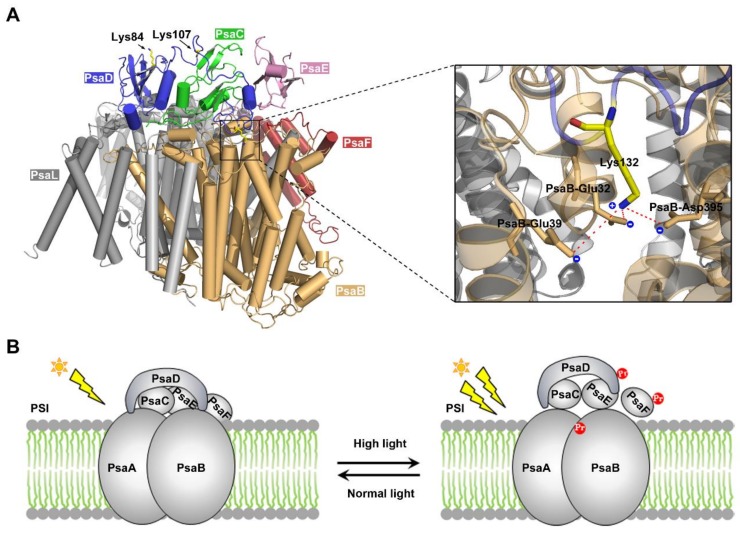
Analysis of the functional roles of PsaD propionylation. (**A**) The crystal structure of PsaD and other PSI subunits. The interactions between Lys132 side chain and the negatively charged amino acids are shown in detail. Lysine amine nitrogen of PsaD is within 5 Å of the Glu32, Gly39, and Glu395 of PsaB. (**B**) Schematic summary of the proposed model of the role of PsaD propionylation during HL acclimation in *Synechocystis*. The Pr highlighted in red represents propionylation.

Increasing evidence shows that diverse PTM events are involved in the regulation of photosynthesis in cyanobacteria [31,44,45,46,47]. According to the dataset presented in this work, there exist many propionylated proteins in the photosynthetic pathway (Figure 5A), and the propionylation level of four photosynthetic proteins (PsaD, PsbC, PsbO, and CpcB) were found to increase in response to HL conditions (Figure 5B). Notably, among the identified photosynthetic proteins, the propionylation level of PsaD (which is likely to interact with many different PSI subunits [48,49,50,51,52,53]), was suggested to be elevated under HL intensity (Figure 6A). PSI is a membrane chlorophyll–protein complex capable of light-induced electron transfer to soluble electron acceptors, resulting in the reduction of low-potential electron carriers (either ferredoxin (Fd) or flavodoxin [54]). PsaD, a subunit of *Synechocystis* PSI, is a conserved peripheral protein on the reducing side of PS I [55], and plays a critical role in the stability of PSI [39,56]. Specifically, the mutation of Lys132 decreases the stability of PSI and is harmful to the cell growth above 30 °C [39]. Lys132 is highly conserved, and plays an important role in maintaining an electrostatic stabilization of the C terminus of PsaD [39]. To assess how modulation of protein propionylation affects its function, we examined the crystal structure of PSI complex from *Synechocystis* [57]. It showed that Lys132 in PsaD is adjacent to the acidic amino acids Glu32, Glu39, and Asp395 of PsaB within 5 Å, and may form intra-molecular electrostatic interactions with these acidic amino acids (Figure 7A). Thus, it is conceivable that propionylation in Lys132 may disrupt specific intra-molecular electrostatic interactions and decrease the structural stability of PsaD, thereby impairing the overall stability of PSI. 

It is well-known that all photosynthetic organisms are often exposed to strong fluctuations in light intensity in their growth environments [58]. To cope with light fluctuations, cyanobacteria as well as plants have developed complex regulatory machinery to prevent their photosynthetic apparatus from high light conditions. For example, cyanobacteria acclimate to HL conditions by adjusting photosystem stoichiometry through a decrease of PSI abundance in thylakoid membranes [59,60], and this decrease in PSI content is essential for *Synechocystis* to accommodate the continuous HL conditions in *Synechocystis* [61]. As PsaD has been proven to be a key protein in stabilizing the reducing side of PSI [56], and its integration considerably decreases the turnover of PSI [62], it is conceivable that the mechanism controlling PsaD level/modification is tightly associated with PSI abundance and HL response in *Synechocystis*. In light of our results and the published data, we propose a molecular model of propionylation during HL acclimation in *Synechocystis* (Figure 7B). Under HL intensity, multiple photosynthetic proteins were propionylated, such as the PSI-D subunit PsaD. Propionylation of PsaD reduced the electrostatic stabilization of its C terminus and disrupted the electrostatic interaction between PsaD and other PSI subunits, which may lead to the decreased PSI stability and abundance. These coordinated responses can enable cyanobacteria to avoid absorbing excess light energy and escape from the photoinhibition and photodamage caused by HL intensity. Consistent with this model, down-regulation of the PSI abundance during the acclimation to HL has been observed in *Synechocystis* [59,63,64], although the mechanism remains unclear. Therefore, reversible propionylation may be an important and previously unknown regulatory mechanism involved in the regulation of PSI abundance and HL acclimation in *Synechocystis*. 

As one of the diverse types of PTM, protein propionylation competes with other forms of acylation—including acetylation, malonylation, crotonylation, succinylation, and butyrylation—for the same ɛ-amino group of lysine residue [65]. Thus, it is likely that there exists a complicated interplay and crosstalk between lysine propionylation and other PTMs, which ultimately regulate protein functions and photosynthetic pathways. Revealing the combinatorial and hierarchical patterns of propionylation and other PTMs will be indispensable for us to understand metabolic and photosynthetic pathways in cyanobactria.

Overall, our results indicate that lysine propionylation is widespread in cyanobacteria and that it is likely to be an important regulatory mechanism in carbon metabolism and photosynthetic pathways in cyanobacteria. Future studies should be directed toward a better understanding of the underlying molecular mechanisms.

## 4. Materials and Methods 

### 4.1. Cell Culture and Protein Extraction

The wild-type strain of *Synechocystis* was grown in BG-11 liquid medium bubbling with filtrated air at 30 °C under continuous illumination at 40 µmol photons m^−2^ s^−1^ [66]. Cells were grown under several conditions and harvested at different growth stages. For stress treatments, the cultures were grown to the exponential phase (OD_730_ = 0.7~0.8) and immediately resuspended in medium deficient of nitrogen [67], iron [68], potassium [69], or A5 (trace metal elements). Briefly, cells in the exponential phase of growth were harvested by centrifugation, washed twice with nitrogen deficiency medium BG11 (lacking the specified nutrient), and immediately resuspended in medium BG11 without the specified nutrient: in Fe-free medium BG11 that added an Fe-binding chelator to the growth medium; in potassium-free medium BG11 that contained Na_2_HPO_4_ instead of K_2_HPO_4_; in nitrogen-free medium without nitrate (NaNO_3_); or in A5-free medium BG11 (without the trace elements H_3_BO_3_, MnCl_2_•4H2O, ZnSO_4_•7H_2_O, Na_2_MoO_4_•2H_2_O, CuSO_4_•5H_2_O, and Co(NO_3_)_2_•6H_2_O). For HL treatment, cells in the exponential growth phase (OD_730_ ≈ 0.7~0.8) were harvested and adjusted to an OD_730_ of ~0.3 with fresh medium in tubes (φ40 × 200 mm) containing 100-mL cultures that were exposed to HL (400 µmol photons m^−2^ s^−1^) for designated times. For high salt stress and heterotrophic conditions, cells grown to the exponential phase were transferred to fresh medium supplemented with 0.685 M NaCl or 5 mM glucose. Cells with different treatments were harvested by centrifugation at 6000 g for 5 min at 4 °C, washed twice with ice cold PBS buffer, and resuspended in ice-cold lysis buffer (20 mM Tris-Cl, pH 7.5, 150 mM NaCl, 1% Triton X-100, 50mM Nicotinamide, pH 7.5 and 1 × protease inhibitor cocktail). The mixture was then disrupted by sonication (2s on, 2s off) for 30 min on ice with an output of 135 W by a JY92-IIN sonicator (Ningbo Scientz Biotechnology Co., Ltd., Ningbo, China), and centrifuged at 5000× *g* for 30 min at 4 °C. The supernatants were stored in aliquots at −80 °C until further use. Protein concentrations were measured using a BCA Protein Assay Kit (Beyotime, Jiangsu, China).

### 4.2. In-Solution Trypsin Digestion and Immunoaffinity Enrichment of Propionylated Peptides

Cell lysates were precipitated by using five volumes of ice-cold acetone, then washed twice with 80% (v/v) ice-cold acetone. The precipitated proteins were redissolved in 50 mM ammonium bicarbonate, then in-solution digested by trypsin, as previously described [44,70]. The propionylated peptides were enriched using anti-propionyl lysine antibody (PTM Biolabs Inc., Chicago, IL, USA), as previous described [30]. Briefly, tryptic peptides were resuspended in NETN buffer containing 50 mM Tris-HCl (pH 8.0), 100 mM NaCl, 1 mM EDTA, and 0.5% Nonidet P-40, and centrifuged at 5000 g for 10 minutes. The supernatant was incubated with agarose beads conjugated with anti-propionyllysine antibody and the mixture was gently rotated for 6 h at 4 °C. The beads were washed sequentially with NETN buffer (three times), ETN buffer (50 mM Tris-Cl, pH 8.0, 100 mM NaCl, 1 mM EDTA, twice), and water (once). The propionylated peptides were eluted by three washes with 1% trifluoroacetic acid. The resulting propionylated peptides were condensed in a vacuum centrifuge and desalted by self-packed C_18_ STAGE tips according to the manufacturer’s instructions, prior to nano-HPLC-MS/MS analysis.

### 4.3. Nano-HPLC–MS/MS Analysis

The enriched peptides were dissolved in the HPLC buffer A (0.1% (v/v) formic acid in water), and analyzed by online nanoflow LC−MS/MS using an easy nLC-1000 system (Thermo Fisher Scientific, Waltham, MA, USA) connected to a Q-Exactive (Thermo Fisher Scientific, Waltham, MA, USA) mass spectrometer. Briefly, samples were injected onto the analytical C18-nanocapillary LC column (5 µm particle size, 100 Å pore diameter) and eluted at a flow rate of 300 nL/min with a 40-min linear gradient of 6–90% solvent B (98% ACN/0.1% formic acid, v/v). The peptides were then directly ionized and sprayed into a Q-Exactive mass spectrometer via a nanospray ion source. Mass spectrometer analysis was carried out in a data-dependent mode with full scans (350–1800 m/z) acquired using an Orbitrap mass analyzer at a mass resolution of 70,000 at m/z = 200. Following every survey scan, up to 20 of the most intense precursor ions were picked for MS/MS fragmentation by higher-energy C-trap dissociation (HCD) with a normalized collision energy of 28%. The dynamic exclusion duration was set to be 10 s with a repeat count of 1 and ±10 ppm exclusion window. The electrospray voltage applied was 2.0 kV. The automatic gain control (AGC) for full MS was set to 3 × 10^6^ ions, with 5 × 10^4^ ions for MS/MS, using ion injection times of 50 and 200 ms, respectively. Internal calibration was carried out using lock mass at m/z 445.120025.

### 4.4. Experimental Design and Statistical Rationale

To identify as many propionylation events as possible in this work, we performed a series of preliminary experiments using small amount of *Synechocystis* proteins to find the best protocol, such as: the optimum amount of starting protein; the ratio of antibody to agarose and peptides; the incubation time of antibody, agarose, and peptides; and the optimum operating parameters of mass spectrometers. Based on the results of these preliminary experiments, we performed a global lysine propionylation analysis of *Synechocystis* using optimized experimental procedures as described in this manuscript. For the enrichment and motif analysis, a Benjamini-corrected statistical significance value (adjusted *p* value) cutoff of 0.05 was used to control the family-wide false discovery rate.

### 4.5. Data Analysis and Peptide Identification

The MS/MS spectra were searched against the *Synechocystis* protein database from the Cyanobase database (http://genome.microbedb.jp/cyanobase/*Synechocystis*/, including 3672 protein sequences, released 2012) concatenated with a reverse decoy database and common contaminants by using MaxQuant software (version 1.3.0.5) [71]. The cleavage enzyme was specified as trypsin, and the maximum missed cleavage sites were set as 2. The precursor and fragmented ion mass tolerances were 10 ppm and 0.02 Da, respectively. Carbamidomethylation (Cys) was specified as a fixed modification, while oxidation (Met), deamidation (Asn/Gln), propionylation (Lys), and acetylation (Protein N-terminal) were set as variable modifications. Minimum peptide length was set at six. The maximum false discovery rate (FDR) for modification site, peptide, and protein were specified as 1%. All MS/MS spectra for propionylated peptides were manually inspected using criteria previously reported [24]. The identified peptides with C-terminal propionylation were discarded prior to bioinformatics analysis to improve the data quality.

### 4.6. Bioinformatics Analysis

All identified propionylated proteins were classified into biological process and molecular function class based on the Gene Ontology (GO) terms by Blast2GO software [72]. The subcellular localization of identified proteins was performed by the PSORTb program [73]. Functional enrichment analyses were carried out by DAVID for the GO term, KEGG (Kyoto Encyclopedia of Genes and Genomes) pathways, and protein family (PFAM) domains [74,75]. The protein–protein interaction (PPI) network of propionylated proteins were visualized by Cytoscape [76] using the interaction data from the *Synechocystis* PPI database (http://bioportal.kobic.kr/SynechoNET) [31,77]. Amino acid sequence motifs were analyzed using the pLogo web tool (version 1.2.0) [78]. The secondary structures around the propionylated lysines were predicted by using the NetSurfP tool [79]. The structure models were prepared from the available crystal structure via structural modeling using a SWISS-MODEL Server (http://swissmodel.expasy.org//SWISS-MODEL.html). The PyMOL Molecular Graphics System (version 1.7.2, http://www.pymol.org) was employed to present the structural results of this study. In our data, the corresponding *p* value < 0.05 was considered statistically significant. The lipoproteins and integral membrane proteins were analyzed by the LipoP 1.0 server [25] (http://www.cbs.dtu.dk/services/LipoP/) and THHMM server 2.0 [24] (http://www.cbs.dtu.dk/services/TMHMM/) as previously described [23].

### 4.7. Production of Specific Antibodies against Synechocystis Proteins

The generation of polyclonal antibodies against *Synechocystis* proteins was carried out by ABclonal Inc. (Wuhan, China). Briefly, to produce the polyclonal antibodies against PsbC (photosystem II CP43 protein), CpcB (phycocyanin beta subunit), and PsaD, full-length cDNA of these genes were amplified and cloned into the pGEX-4T expression vector (Pharmacia). The resulting plasmids were transformed into *Escherichia coli* strain BL21 (DE3) for overexpression. Cells growing logarithmically were treated with 1-mM isopropyl-β-D-thiogalactopyranoside (IPTG) for 4 h at 30 °C. The fusion proteins were then purified by performing His-tag affinity chromatography. For the polyclonal antibody against propionylated PsaD at lysine 132, a synthetically modified peptide of GQNPEPVTIK(pr)FSGKAPYE was used to generate the site-specific propionyllysine antibody, whereas the unmodified peptide of GQNPEPVTIKFSGKAPYE was used as control. Following purification of these antigens, immunization and sampling of the antisera from rabbit were performed by ABclonal according to standard operating procedures. The specificity of the generated antibodies was determined by the manufacturer using ELISA and western blotting.

### 4.8. Immunoprecipitation and Western Blotting

To further validate propionylated proteins, we performed immunoprecipitation and western blotting to analyze the *Synechocystis* proteins in vivo using the generated specific protein antibodies as previously described [30,31]. Briefly, Dynabeads Protein G (Invitrogen AS, Oslo, Norway) with specific protein antibody was incubated with whole cell lysates in PBS containing 0.02% Tween 20 by gentle rotation overnight at 4 °C. The beads were then washed three times to remove the unbound proteins. Bound proteins were boiled in SDS loading buffer for 5 min then subjected to 12% SDS-PAGE and transferred to a polyvinylidene difluoride (PVDF) membrane for further western blotting analysis. The membrane was incubated with generated *Synechocystis* protein antibody or anti-propionyl lysine antibody (PTM Biolabs Inc., Chicago, IL) (1:2,000, in TBST/5% BSA). The membrane was then washed three times with TBST buffer (25 mM Tris-HCl, pH 8.0, 150 mM NaCl, 0.1% Tween 20) and incubated with horseradish peroxidase-conjugated goat antirabbit antibody (1:5000 dilutions) for 1 h at 37 °C. After being washed with TBST buffer, the membrane was visualized with enhanced chemiluminescence (ECL) immune-blotting detection reagents (Advansta, CA, USA). The density of each band was determined with a fluorescence scanner (ImageQuant TL, GE Healthcare). Densitometry analysis was performed via the ImageJ suite (http://rsbweb.nih.gov/ij/).

### 4.9. Site-directed Mutagenesis and Purification of fructose-1,6-/sedoheptulose-1,7-bisphosphatase (FbpI)

The full-length *fbpI* gene (*slr2094*) was amplified by using *Synechocystis* genomic DNA as a template with the following primers: FbpI-sense (5′-GCTAGCGTGGACAGCACCCTCGGTT-3′) and FbpI-antisense (5′-CTCGAGATGCAGTTGGATTACTTTGGGGCT-3′). The PCR amplicon was inserted into pMD18-T (Takara). The site-directed mutation of the *fbpI* gene was introduced into the selected sites by PCR reaction and, subsequently, the mutagenesis was completely sequenced to confirm the presence of the site-directed mutation. Mutagenic primers were given below with the mutated base triplets underlined: FbpI-K156R-sense (5′-CCGAAAACCTGCGGATCCTCTCCGATTGCCTCAACCG-3′); FbpI-K156R-antisense (5′-TCCGCAGGTTTTCGGTGGCCGAT-3′); FbpI-K336R-sense (5′-TGCGGGAAAGCCCCAAAGTAATCCAAC-3′); FbpI-K336R-antisense (5′-GGCTTTCCCGCATATGGACAGTGTCTACAAAGCGGGCGG-3′). Next, the plasmids containing the wild-type or point-mutated fbpI genes were digested with NheI and XhoI, and the fragments were inserted into pET21b (Novagen), followed by transformation into *E. coli* BL21 (DE3) for protein expression.

The wild-type *E. coli* BL21 (DE3)/pET21b-fbpI and the site-directed mutants were cultured in 5 mL Luria-Bertani (LB) medium supplemented with ampicillin (50 g/mL) overnight at 37 °C. The cultures were then transformed into fresh LB medium with ampicillin (50 g/mL) at 37 °C in shaking flasks to optical density at 600 nm of 0.4–0.6. Cells were induced with 0.1 mM isopropyl-β-D-1-thiogalactopyranoside (IPTG) at 16 °C for 12 h. The cultures were harvested by centrifugation at 6000 rpm for 5 min at 4 °C. After being washed twice with ice-cold PBS, the pellets were resuspended in binding buffer (20 mM Tris-HCl (pH 8.0), 500 mM NaCl) and disrupted by high-pressure homogenizer with an output of 1500 W (1500 bar, JN-02C, JNBIO, Guangzhou, China). Cellular debris were removed by centrifugation at 8000× *g* for 30 min at 4 °C, and the supernatants were loaded onto an affinity Ni^2+^ column (Qiagen Inc., Chatsworth, CA) pre-equilibrated with the binding buffer. After the column was washed with washing buffer (20 mM Tris-HCl, pH 8.0, 500 mM NaCl, and 30 mM imidazole or 60 mM imidazole), the target protein was then eluted with elution buffer (20 mM Tris-HCl, pH 8.0, 500 mM NaCl, and 100 mM imidazole). The elution was desalted and concentrated using a 30,000 MWCO concentrator (Millipore) in storage buffer (20 mM Tris-HCl, pH 8.0, 50 mM NaCl). Protein concentration was determined by a BCA protein assay (Beyotime, Jiangsu, China) and the purified proteins were analyzed by SDS-PAGE.

### 4.10. Enzymatic Activity Assay

The activity of purified FbpI and its mutants were determined by monitoring dihydronicotinamide adenine dinuclectide phosphate (NADPH) formation from NADP+, resulting in an absorbance increase at 340 nm as previously described [35]. Briefly, the purified protein was incubated with reaction buffer (10 mM MgCl_2_, 100 mM Tris-HCl (pH 8.0), 0.5 mM EDTA, 0.4 mM NADP^+^, 0.5 U/mL yeast glucose-6-phosphate dehydrogenase, and 1.5 U/mL yeast phosphoglucoisomerase). Reactions were pre-incubated at 30 °C for 5 min, then the fructose 1,6-phosphate was immediately added to a final concentration of 0.2 mM. After 15 min incubation at 30 °C, reactions were measured by monitoring the absorbance at 340 nm using SpectraMax M5 (Molecular devices, USA). The reaction mixture without enzyme served as a blank. Enzymatic activity was given as µmol min^−1^ (mg protein)^−1^.

## Supplementary Materials

Supplementary materials can be found at https://www.mdpi.com/1422-0067/20/19/4792/s1.

## Abbreviations

PTM	post-translational modification
MS/MS	high-accuracy tandem mass spectrometry
FDR	false discovery rate
GO	Gene Ontology
KEGG	Kyoto Encyclopedia of Genes and Genomes
PPI	protein-protein interaction network
HL	high light
PSI	photosystem I
PSII	photosystem II
PBS	phycobilisome
LHCII	light harvesting complex II
FbpI	fructose-1,6-/sedoheptulose-1,7-bisphosphatase
PsaD	subunit II of photosystem I
