# Supplementary material for: Lysine Propionylation is a Widespread Post-Translational Modification Involved in Regulation of Photosynthesis and Metabolism in Cyanobacteria
Source: Int J Mol Sci. 2019 Sep 26;20(19):4792. doi: 10.3390/ijms20194792 (PMC6801645; doi:10.3390/ijms20194792)
Supplement: Supplementary file 1 [file ijms-20-04792-s001.zip › SI/TableS/Supplementary Dataset.pdf]

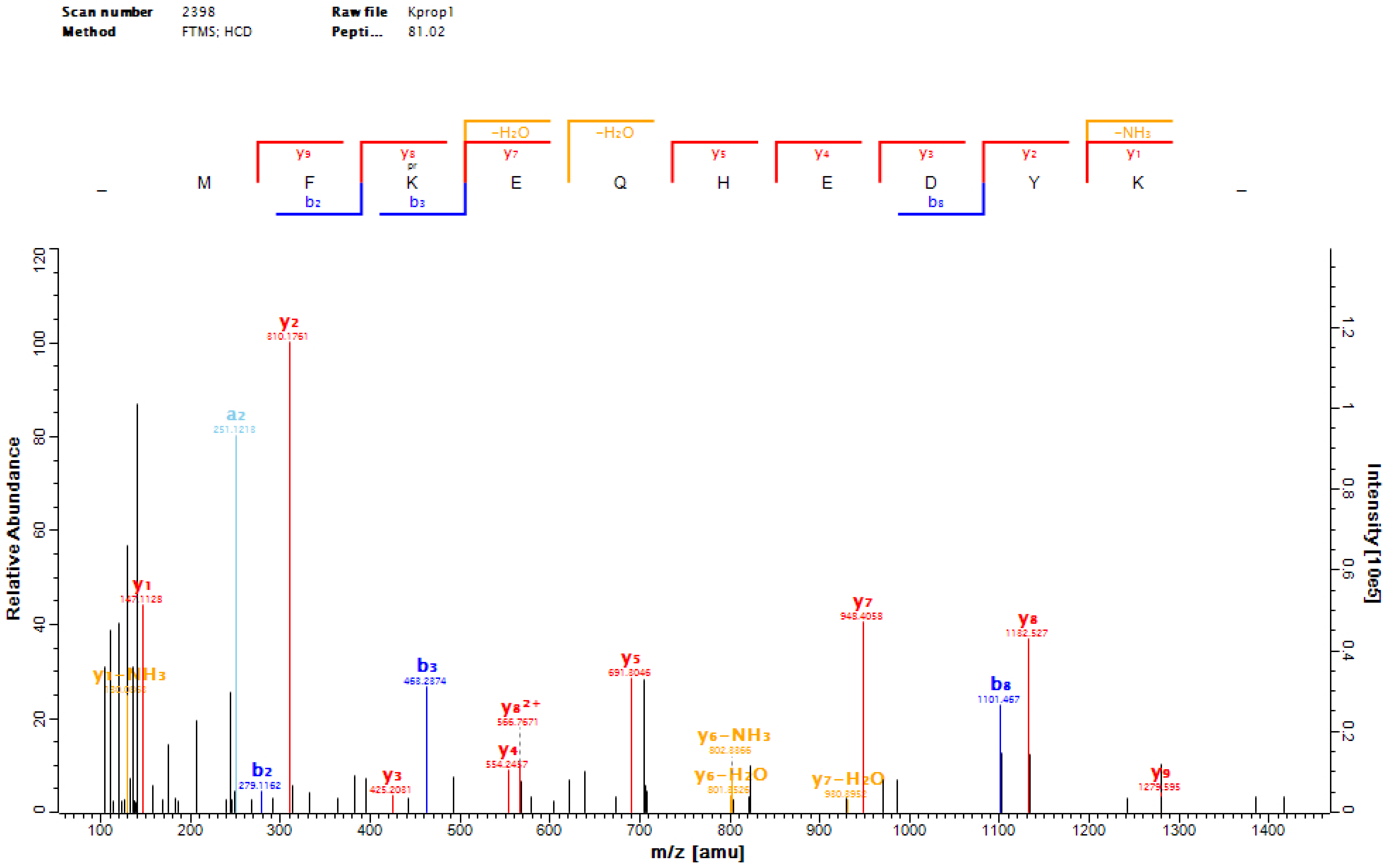

Scan number 2398
Raw file Kprop1
Method FTMS; HCD
Pepti... 81.02
_ M F K E Q H E D Y K _
y9 y8 y7 y5 y4 y3 y2 y1
-H2O -H2O -NH3
b2 b3 b8
y2 810.1761
a2 251.1218
y1 147.1128
y1-NH3
b3 463.2374
y5 691.3046
y7 948.4058
y8 1132.527
b8 1101.467
y8 2+ 566.7671
y4 554.2457
y3 425.2081
b2 279.1162
y6-NH3 802.3366
y6-H2O 801.3526
y7-H2O 930.3952
y9 1279.595
Relative Abundance
Intensity [10e5]
m/z [amu]

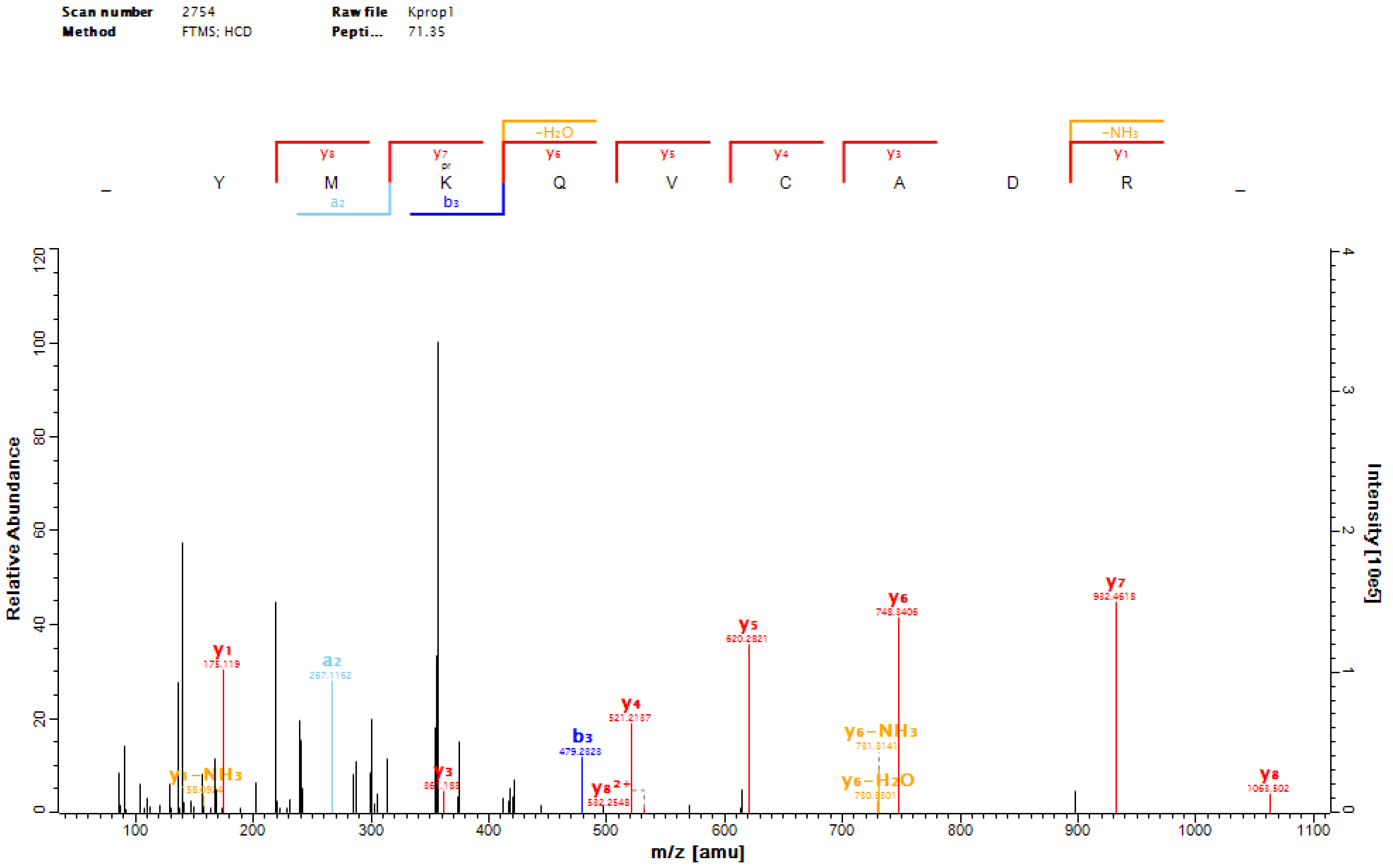

Scan number 2754
Method FTMS; HCD
Raw file Kprop1
Pepti... 71.35
Y M K Q V C A D R
y8 y7 y6 y5 y4 y3 y1
-H2O
-NH3
a2 b3
Relative Abundance
Intensity [10e5]
m/z [amu]
y1 175.119
a2 267.1162
y3
y1-NH3
b3 479.2828
y4 521.2187
y8 2+ 532.2548
y5 620.2821
y6 748.3406
y6-NH3 731.3141
y6-H2O 730.3301
y7 932.4618
y8 1063.502

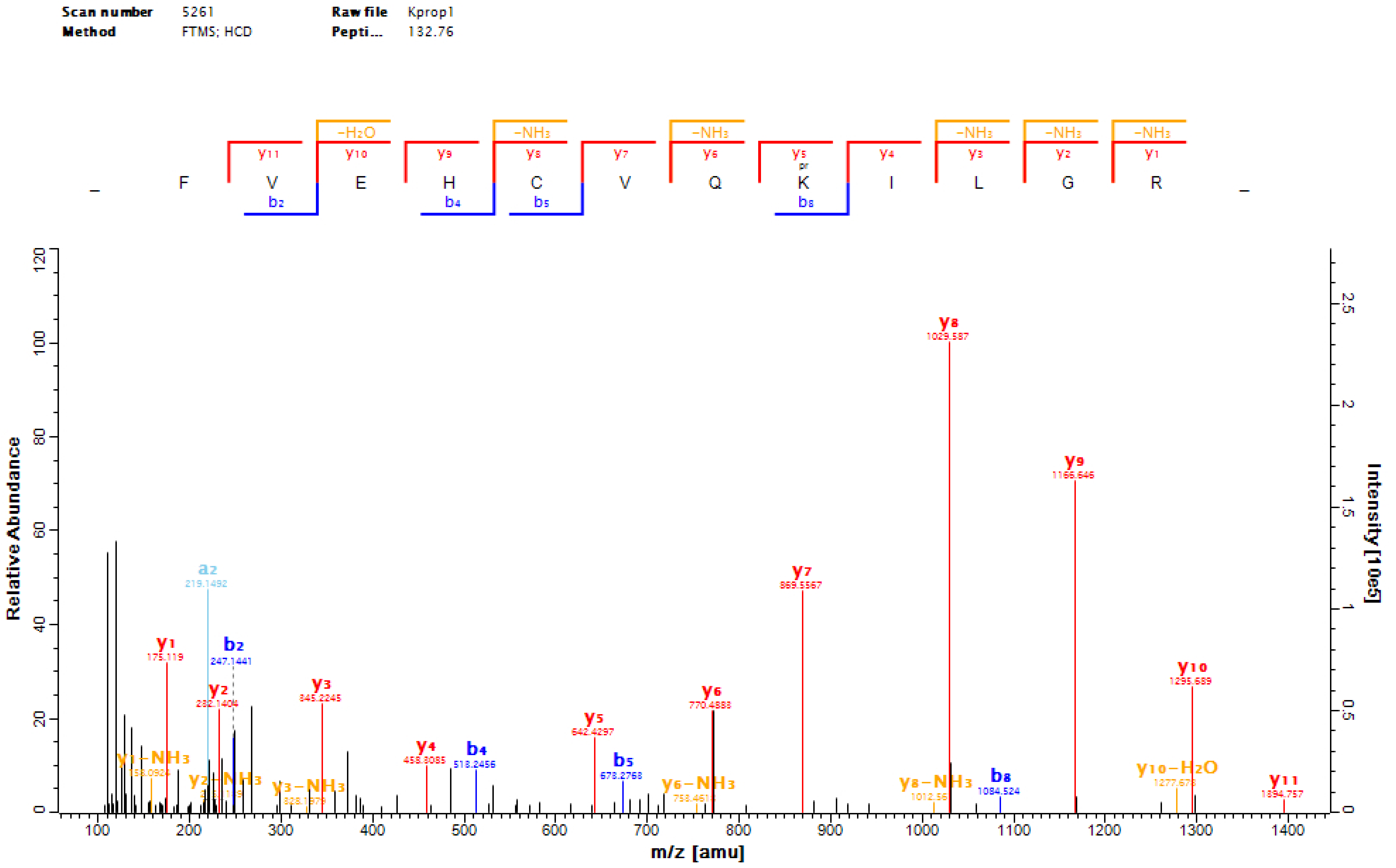

Scan number 5261
Method FTMS; HCD
Raw file Kprop1
Pepti... 132.76
_ F V E H C V Q K I L G R _
-H2O
-NH3
pr
y11 y10 y9 y8 y7 y6 y5 y4 y3 y2 y1
b2 b4 b5 b8
a2 219.1492
y1 175.119
b2 247.1441
y2 232.1404
y3 345.2245
y4 458.3085
b4 513.2456
y5 642.4297
b5 673.2763
y6 770.4883
y7 869.5567
y8 1029.587
b8 1084.524
y9 1166.646
y10 1295.689
y11 1394.757
y1-NH3
y2-NH3
y3-NH3 328.1979
y6-NH3 753.4614
y8-NH3 1012.56
y10-H2O 1277.678
Relative Abundance
Intensity [10e5]
m/z [amu]

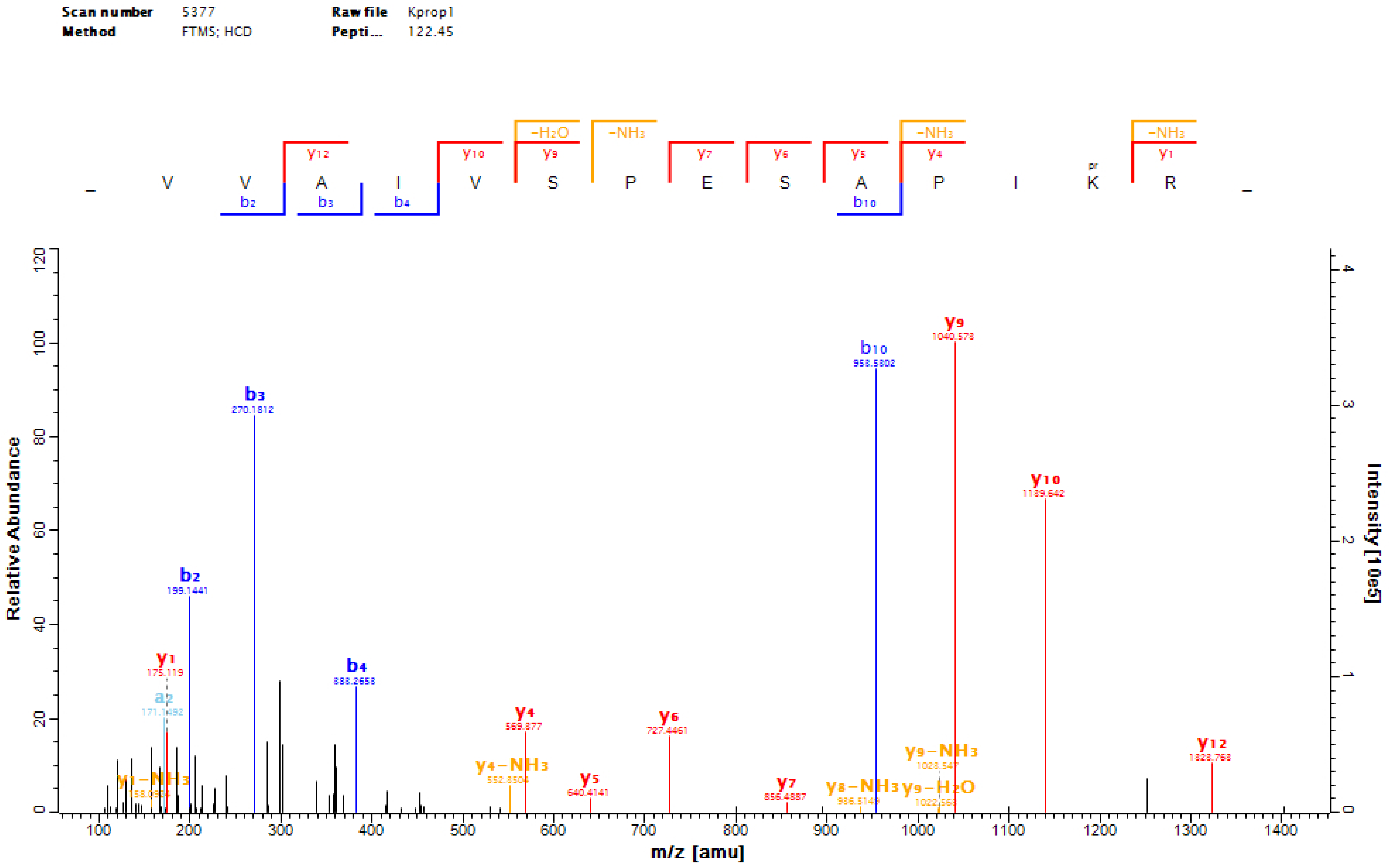

Scan number 5377
Method FTMS; HCD
Raw file Kprop1
Pepti... 122.45
_ V V A I V S P E S A P I K R _
pr
y12
y10
y9
-H2O
-NH3
y7
y6
y5
y4
-NH3
y1
-NH3
b2
b3
b4
b10
Relative Abundance
Intensity [10e5]
m/z [amu]
y9
1040.578
b10
958.5802
b3
270.1812
y10
1139.642
b2
199.1441
y1
175.119
a2
171.1492
b4
383.2653
y4
569.377
y6
727.4461
y12
1323.758
y9-NH3
1023.547
y4-NH3
552.3504
y1-NH3
158.0924
y5
640.4141
y7
856.4887
y8-NH3
936.5149
y9-H2O
1022.563

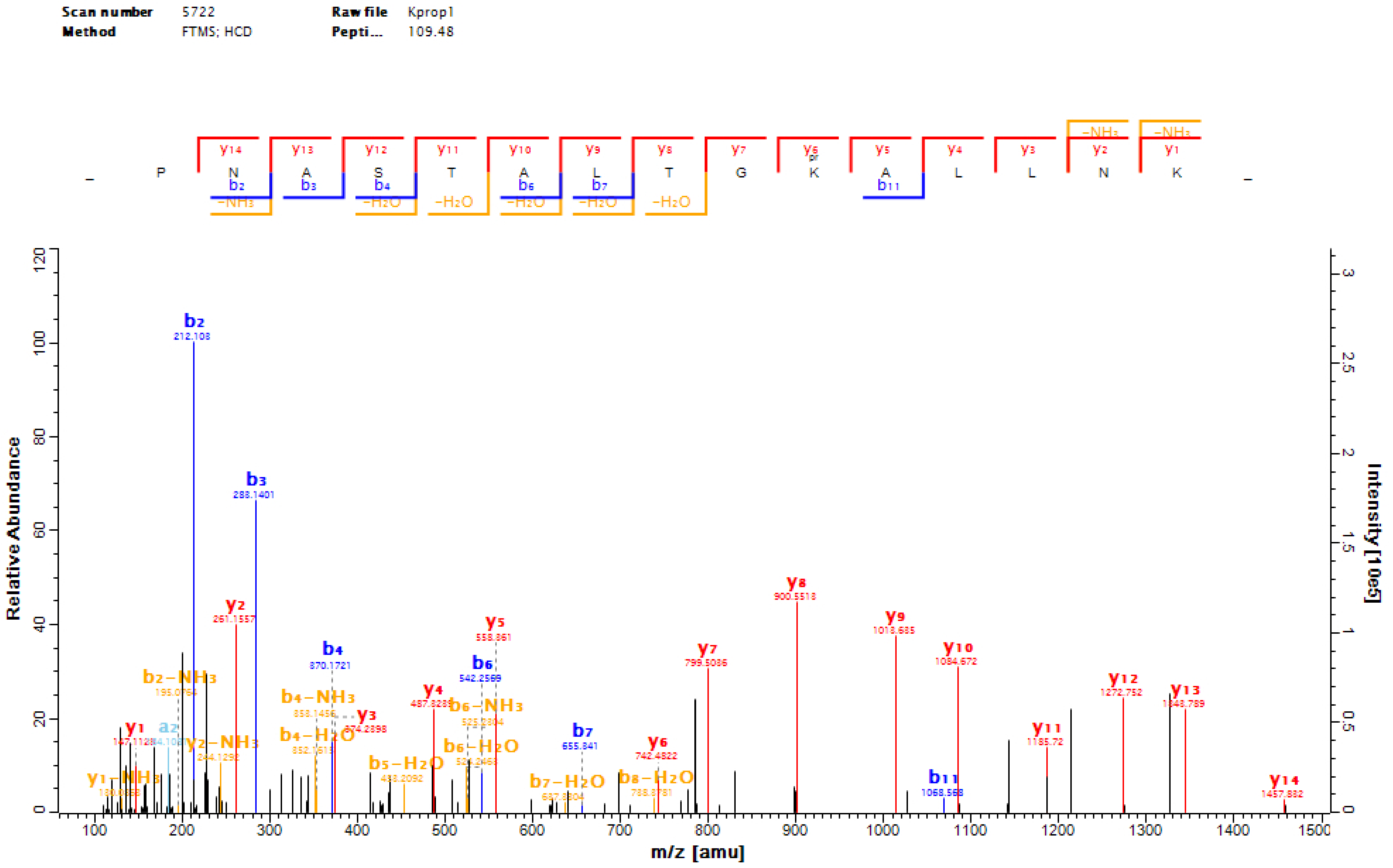

Scan number 5722
Raw file Kprop1
Method FTMS; HCD
Pepti... 109.48
_ P N A S T A L T G K A L L N K _
Relative Abundance
Intensity [10e5]
m/z [amu]
b2 212.108
b3 288.1401
y2 261.1557
b4 370.1721
y5 558.351
b6 542.2569
b2-NH3 195.0764
b4-NH3 353.1456
y3 374.2398
y4 487.3238
b6-NH3 525.2304
y1 147.1128
a2
y2-NH3 244.1292
b4-H2O 352.1613
b5-H2O 453.2092
b6-H2O 524.2463
y1-NH3 130.0863
b7 655.341
y6 742.4822
b7-H2O 637.3304
b8-H2O 738.3781
y7 799.5036
y8 900.5513
y9 1013.635
y10 1084.672
b11 1068.568
y11 1185.72
y12 1272.752
y13 1343.789
y14 1457.832

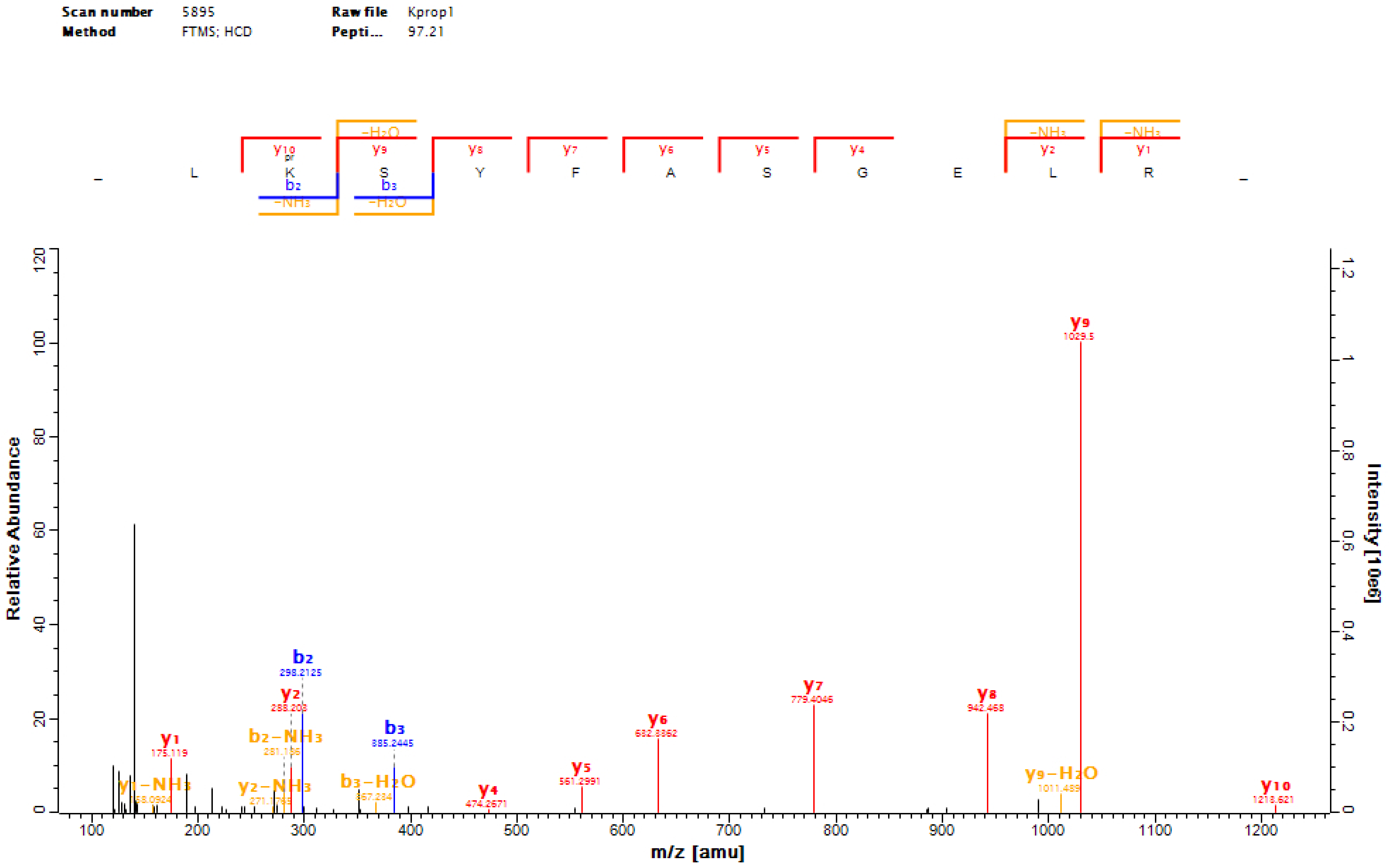
Scan number 5895
Raw file Kprop1
Method FTMS; HCD
Pepti... 97.21
L K S Y F A S G E L R
y10
y9
y8
y7
y6
y5
y4
y2
y1
b2
b3
-H2O
-NH3
Relative Abundance
Intensity [10e6]
m/z [amu]
y1 175.119
y1-NH3 158.0924
y2 288.203
y2-NH3 271.1765
b2 298.2125
b2-NH3 281.186
b3 385.2445
b3-H2O 367.234
y4 474.2571
y5 561.2991
y6 632.3362
y7 779.4046
y8 942.468
y9-H2O 1011.489
y9 1029.5
y10 1218.621

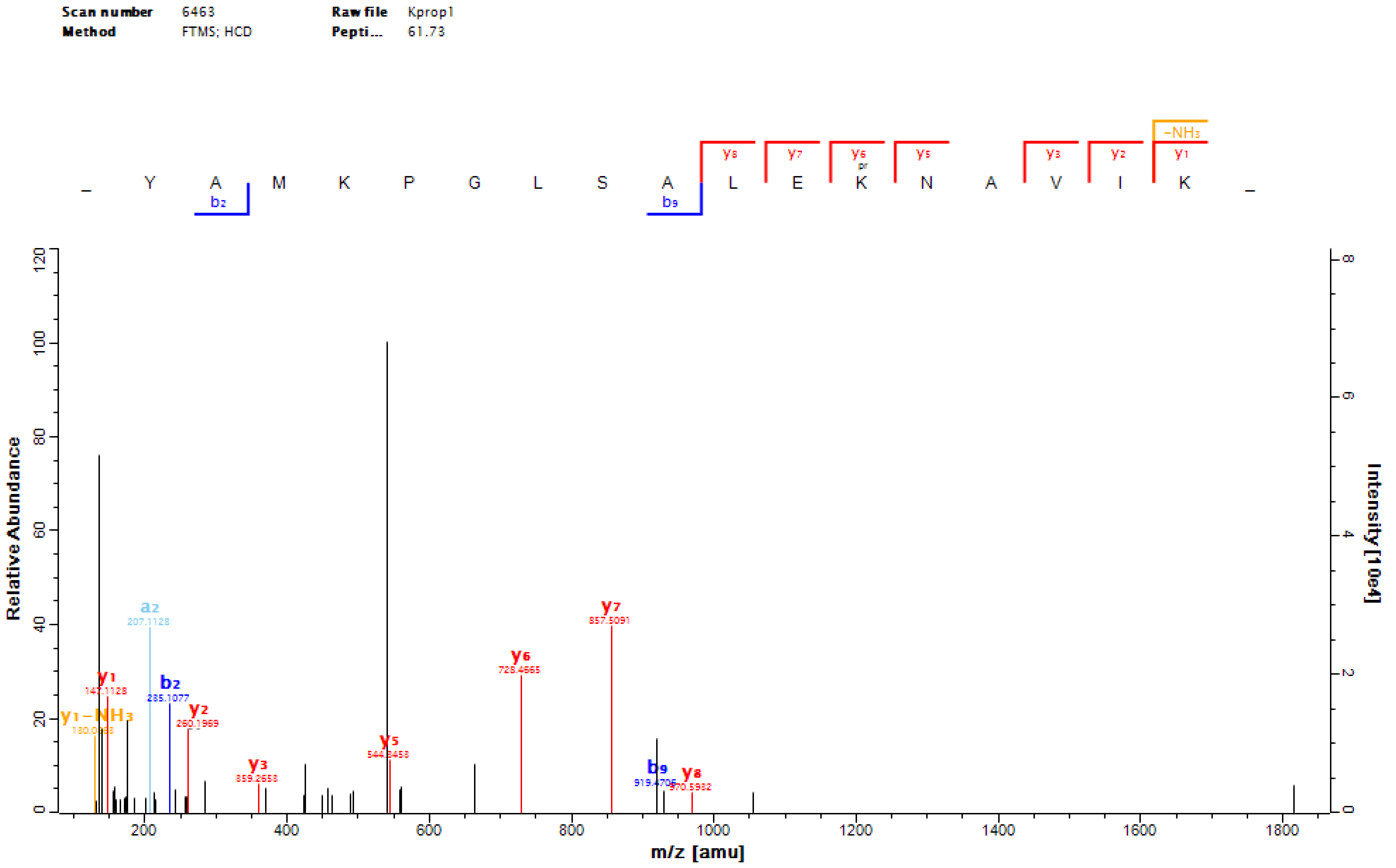

Scan number 6463
Method FTMS; HCD
Raw file Kprop1
Pepti... 61.73
_ Y A M K P G L S A L E K N A V I K _
b2 b9
y8 y7 y6 y5 y3 y2 y1
-NH3
pr
Relative Abundance
Intensity [10e4]
m/z [amu]
a2 207.1128
b2 235.1077
y1 147.1128
y1-NH3 130.0863
y2 260.1969
y3 359.2653
y5 544.3453
y6 728.4665
y7 857.5091
b9 919.4706
y8 970.5932

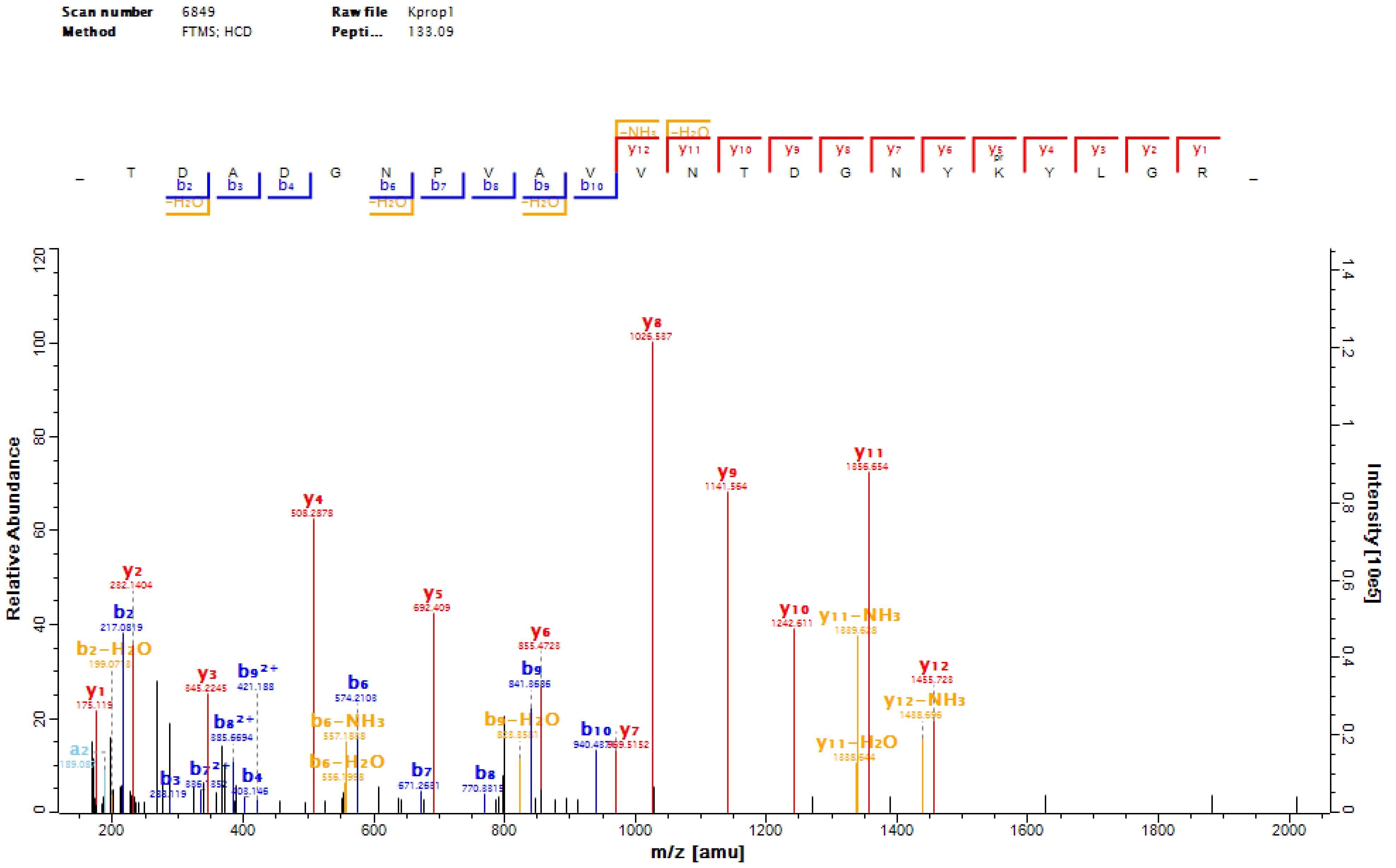

Scan number 6849
Method FTMS; HCD
Raw file Kprop1
Pepti... 133.09
T D A D G N P V A V V N T D G N Y K Y L G R
Relative Abundance
Intensity [10e5]
m/z [amu]
y1 175.119
a2 189.087
b2-H2O 199.0718
b2 217.0819
y2 232.1404
b3 288.119
y3 345.2245
b7 2+ 336.1352
b8 2+ 385.6694
b4 403.146
b9 2+ 421.188
y4 508.2878
b6-H2O 556.1998
b6-NH3 557.1838
b6 574.2103
b7 671.2661
y5 692.409
b8 770.3315
b9-H2O 823.3581
b9 841.3686
y6 855.4728
b10
y7
y8 1026.537
y9 1141.564
y10 1242.611
y11-H2O 1338.644
y11-NH3 1339.628
y11 1356.654
y12-NH3 1438.696
y12 1455.723

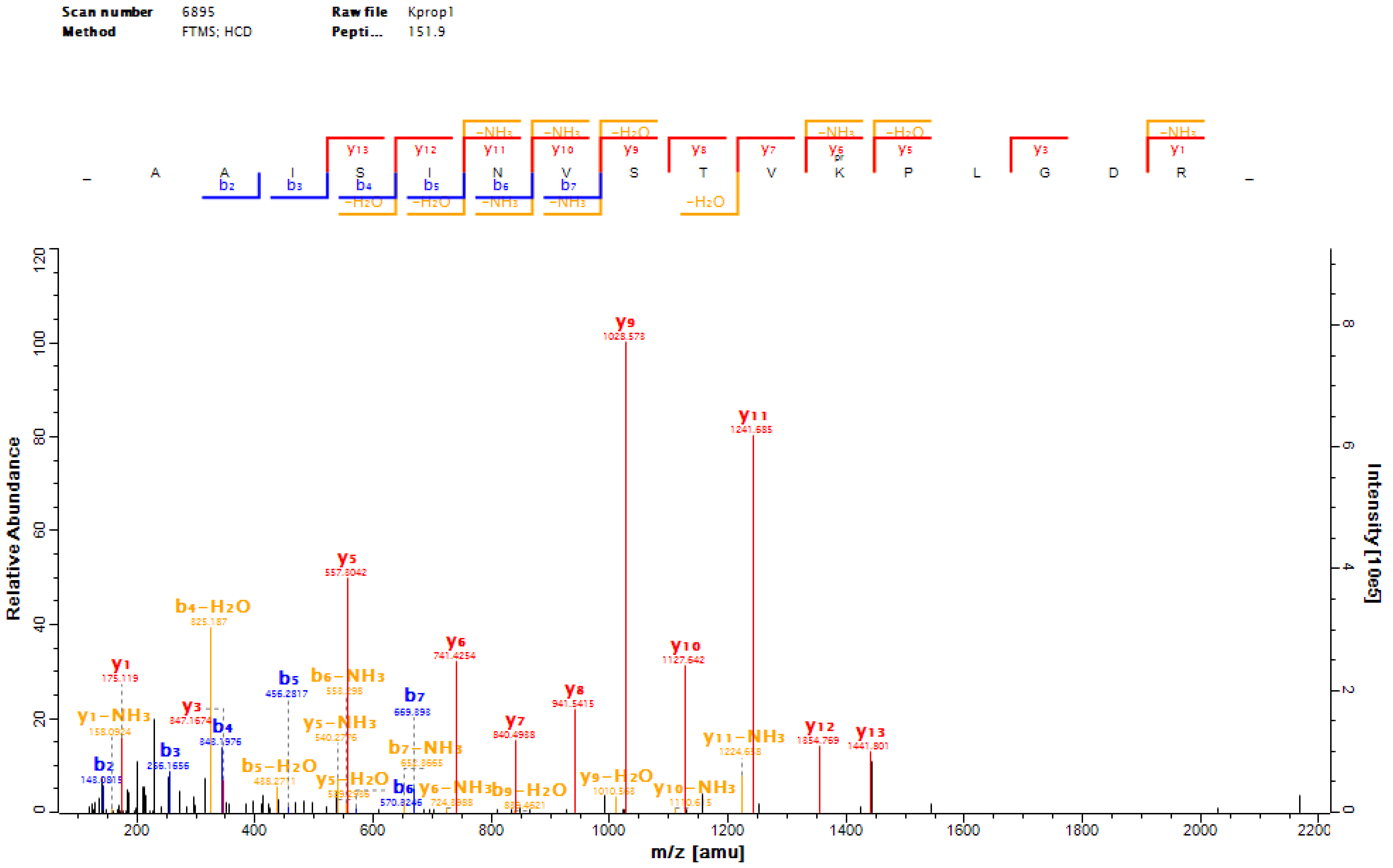

Scan number 6895
Raw file Kprop1
Method FTMS; HCD
Pepti... 151.9
_ A A I S I N V S T V K P L G D R _
y13 y12 y11 y10 y9 y8 y7 y6 y5 y3 y1
b2 b3 b4 b5 b6 b7
−NH3
−H2O
y9
1028.578
y11
1241.685
y5
557.3042
b4−H2O
325.187
y6
741.4254
y10
1127.542
y1
175.119
b5
456.2817
b6−NH3
b7
669.393
y8
941.5415
y3
y1−NH3
b4
y5−NH3
540.2776
y7
840.4938
y11−NH3
1224.658
y12
1354.769
y13
1441.801
b2
b3
b7−NH3
b5−H2O
y5−H2O
b6
570.3245
y6−NH3
724.3988
b9−H2O
y9−H2O
1010.568
y10−NH3
Relative Abundance
Intensity [10e5]
m/z [amu]

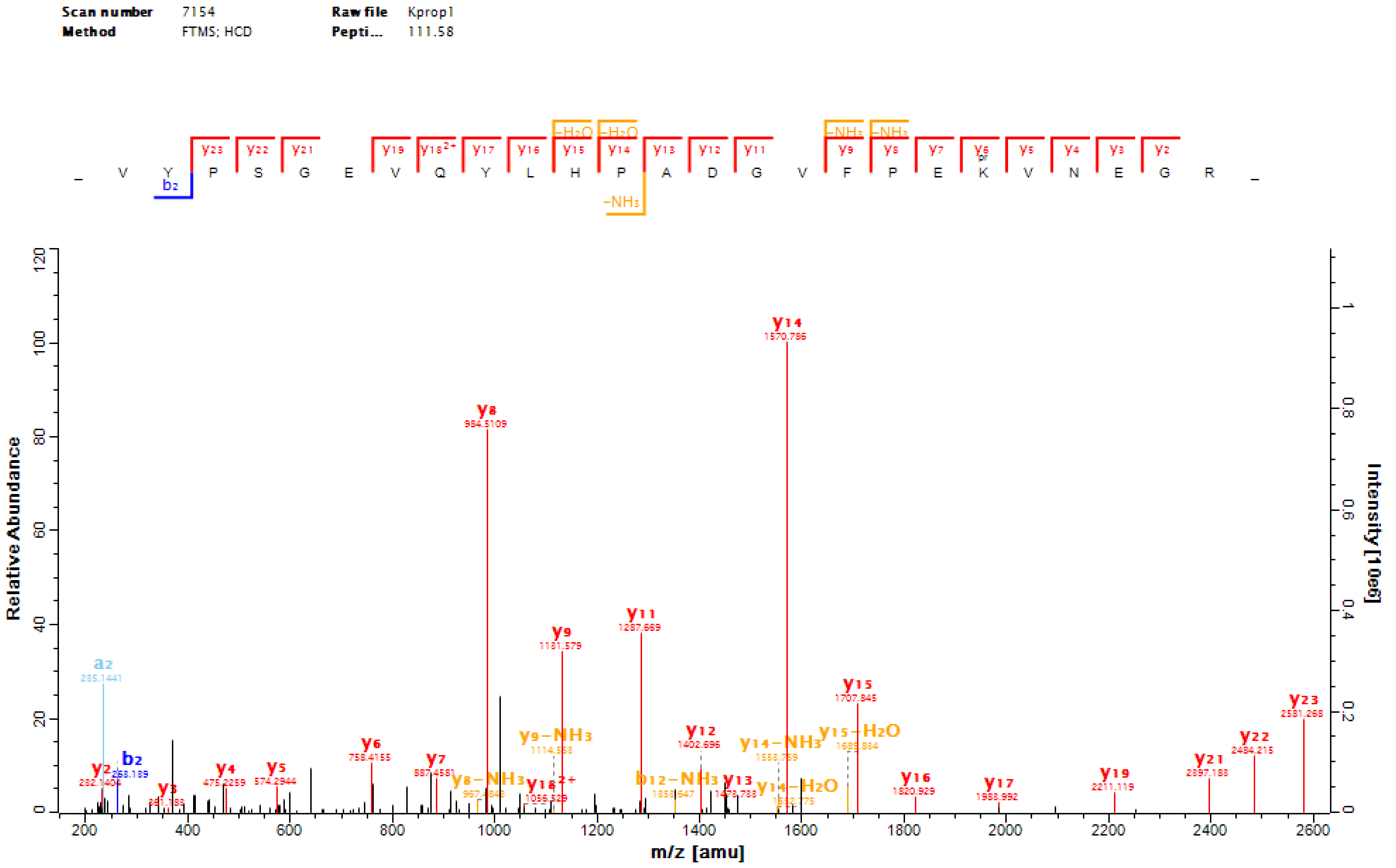

Scan number 7154
Raw file Kprop1
Method FTMS; HCD
Pepti... 111.58
_ V Y P S G E V Q Y L H P A D G V F P E K V N E G R _
y23 y22 y21 y19 y18²⁺ y17 y16 y15 y14 y13 y12 y11 y9 y8 y7 y6 y5 y4 y3 y2
b2
-H2O -H2O -NH3 -NH3 -NH3
Relative Abundance
Intensity [10e6]
m/z [amu]
a2 235.1441
b2 263.189
y2 232.1404
y3 361.183
y4 475.2259
y5 574.2944
y6 758.4155
y7 887.4581
y8 984.5109
y8-NH3 967.4843
y9 1131.579
y9-NH3 1114.553
y18 2+ 1056.529
y11 1287.669
b12-NH3 1358.647
y12 1402.696
y13 1473.733
y14 1570.786
y14-NH3 1553.759
y14-H2O 1552.775
y15 1707.845
y15-H2O 1689.834
y16 1820.929
y17 1983.992
y19 2211.119
y21 2397.183
y22 2484.215
y23 2581.268

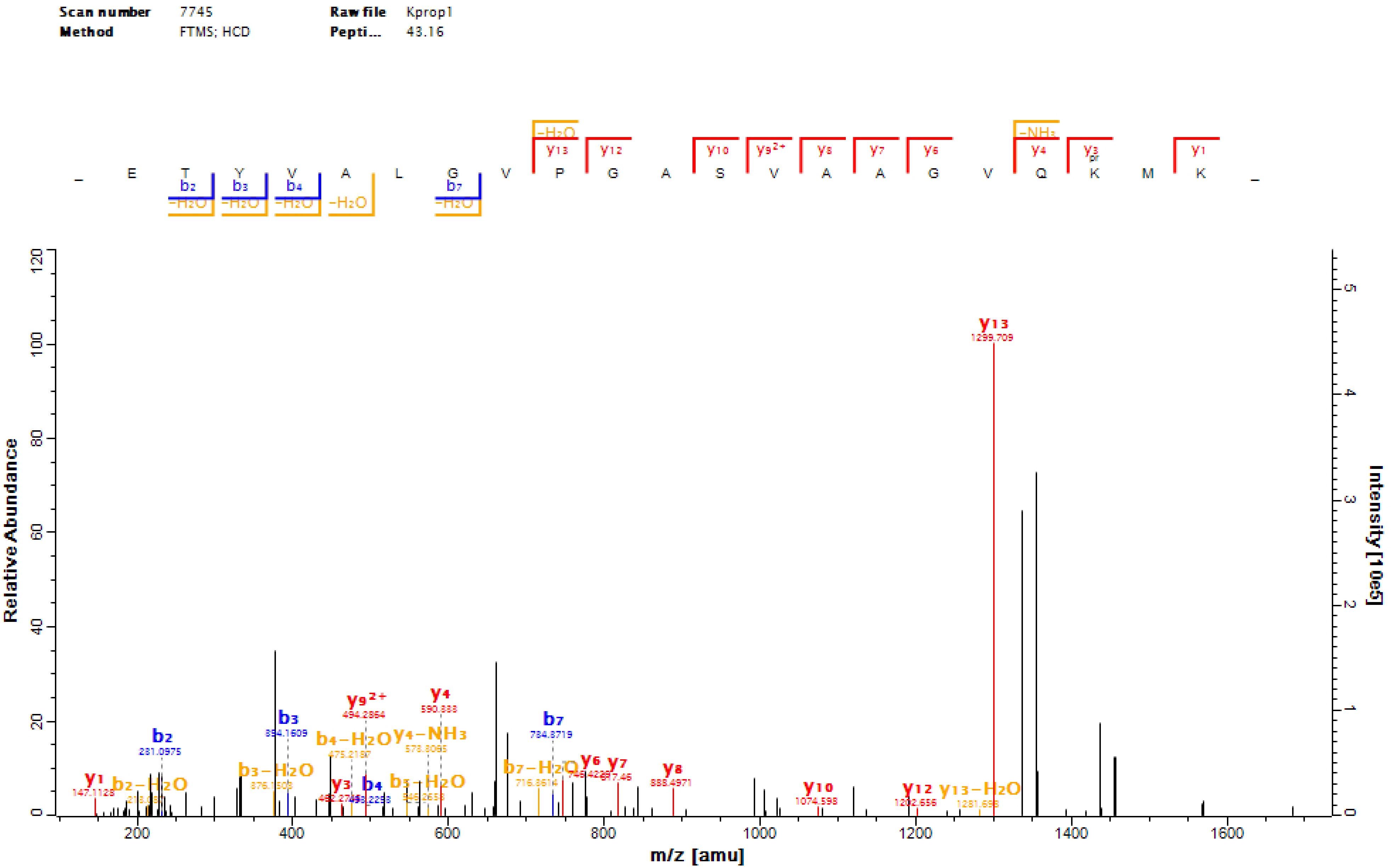
Scan number 7745
Method FTMS; HCD
Raw file Kprop1
Pepti... 43.16
_ E T Y V A L G V P G A S V A A G V Q K M K _
y1 147.1128
b2 231.0975
b2-H2O 213.087
b3 394.1609
b3-H2O 376.1503
y3
b4
b4-H2O 475.2187
y9 2+ 494.2864
y4 590.333
y4-NH3 573.3065
b5-H2O 545.2658
b7 734.3719
b7-H2O 716.3614
y6
y7 817.46
y8 888.4971
y10 1074.598
y12 1202.656
y13 1299.709
y13-H2O 1281.698
Relative Abundance
Intensity [10e5]
m/z [amu]

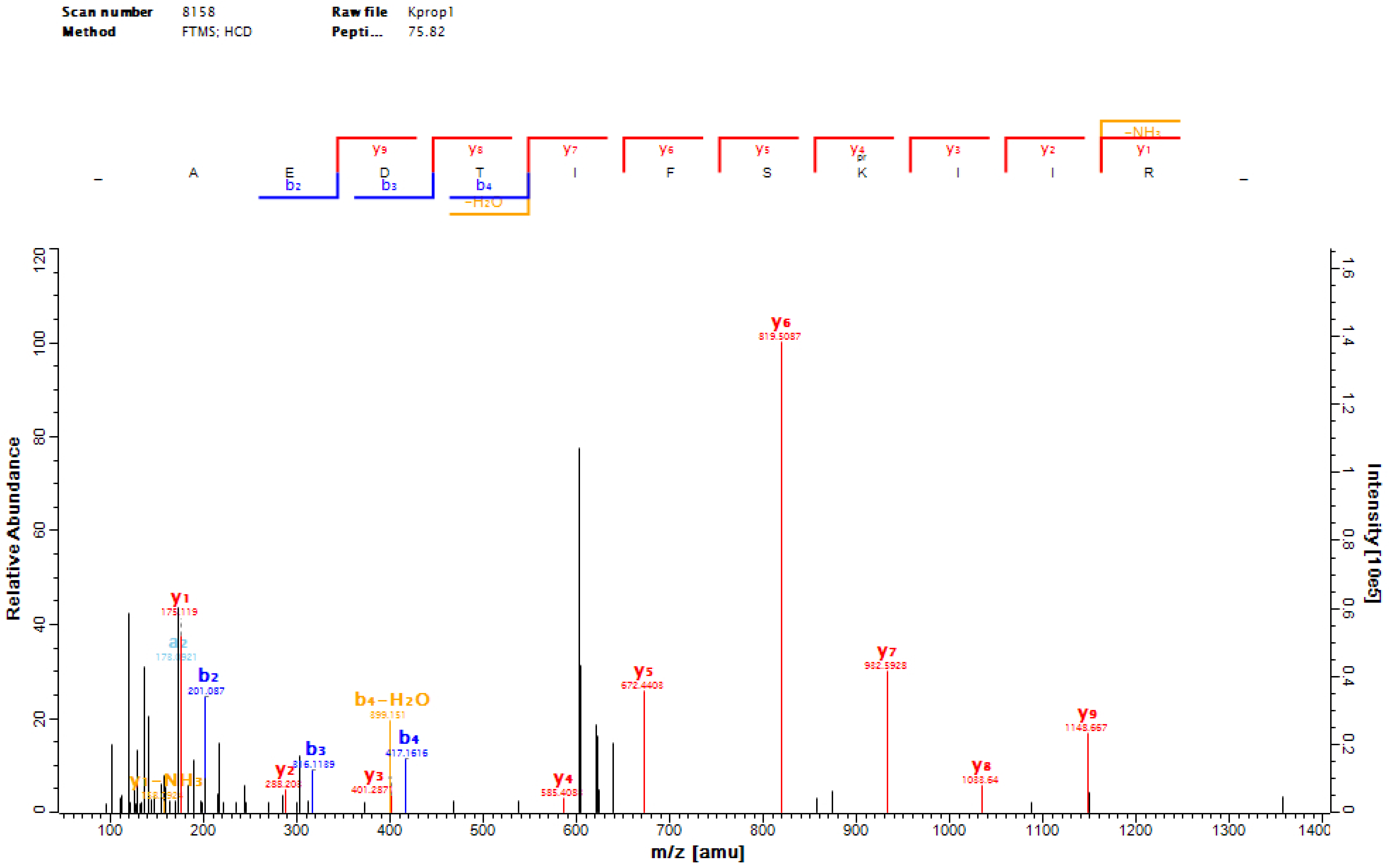

Scan number 8158
Raw file Kprop1
Method FTMS; HCD
Pepti... 75.82
A E D T I F S K I I R
b2 b3 b4 −H2O
y9 y8 y7 y6 y5 y4 y3 y2 y1 −NH3
pr
y1 175.119
a2
b2 201.087
y1−NH3
y2 288.203
b3 316.1189
y3 401.2871
b4−H2O 399.151
b4 417.1616
y4 585.4083
y5 672.4403
y6 819.5087
y7 932.5928
y8 1033.64
y9 1148.667
Relative Abundance
Intensity [10e5]
m/z [amu]
0 20 40 60 80 100 120
0 0.2 0.4 0.6 0.8 1 1.2 1.4 1.6
100 200 300 400 500 600 700 800 900 1000 1100 1200 1300 1400

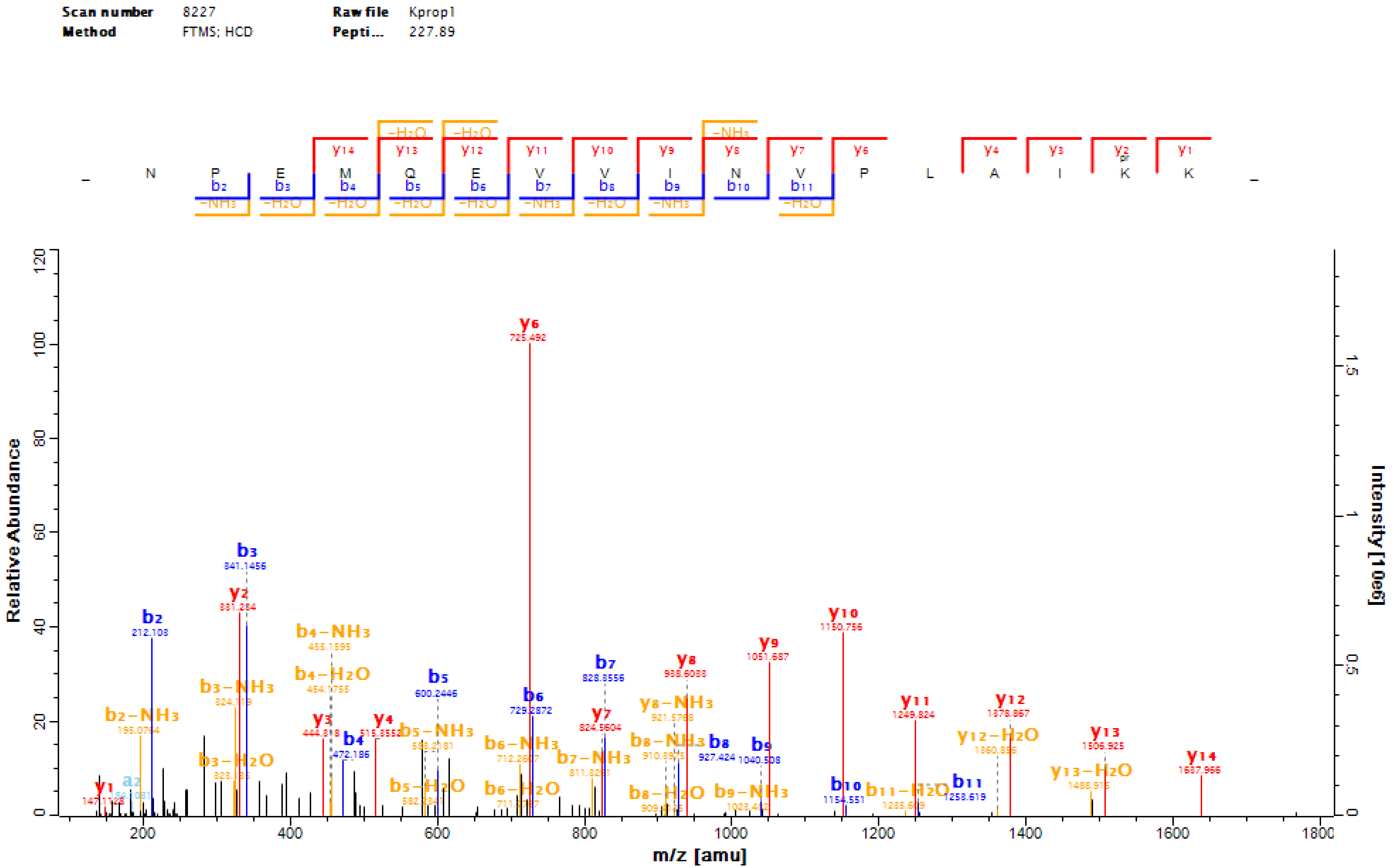

Scan number 8227
Raw file Kprop1
Method FTMS; HCD
Pepti... 227.89
_ N P E M Q E V V I N V P L A I K K _
Relative Abundance
Intensity [10e6]
m/z [amu]
y6 725.492
b3 341.1456
y2 331.284
b2 212.108
b4-NH3 455.1595
b4-H2O 454.1755
b5 600.2446
b3-NH3 324.119
b2-NH3 195.0784
b6 729.2872
b7 828.3556
y7 824.5604
y8 938.6083
y8-NH3 921.5768
y9 1051.687
y10 1150.756
y11 1249.824
y12 1378.867
y12-H2O 1360.856
y13 1506.925
y13-H2O 1488.915
y14 1637.966
y3 444.318
y4 515.3552
b4 472.186
b5-NH3 583.2181
b5-H2O
b6-NH3 712.2607
b6-H2O
b7-NH3 811.3291
b8-NH3
b8 927.424
b9 1040.508
b8-H2O
b9-NH3
b10 1154.551
b11-H2O
b11 1253.619
b3-H2O
y1 147.1128
a2

Scan number 8915 **Raw file** Kprop1
Method FTMS; HCD **Pepti...** 131.56

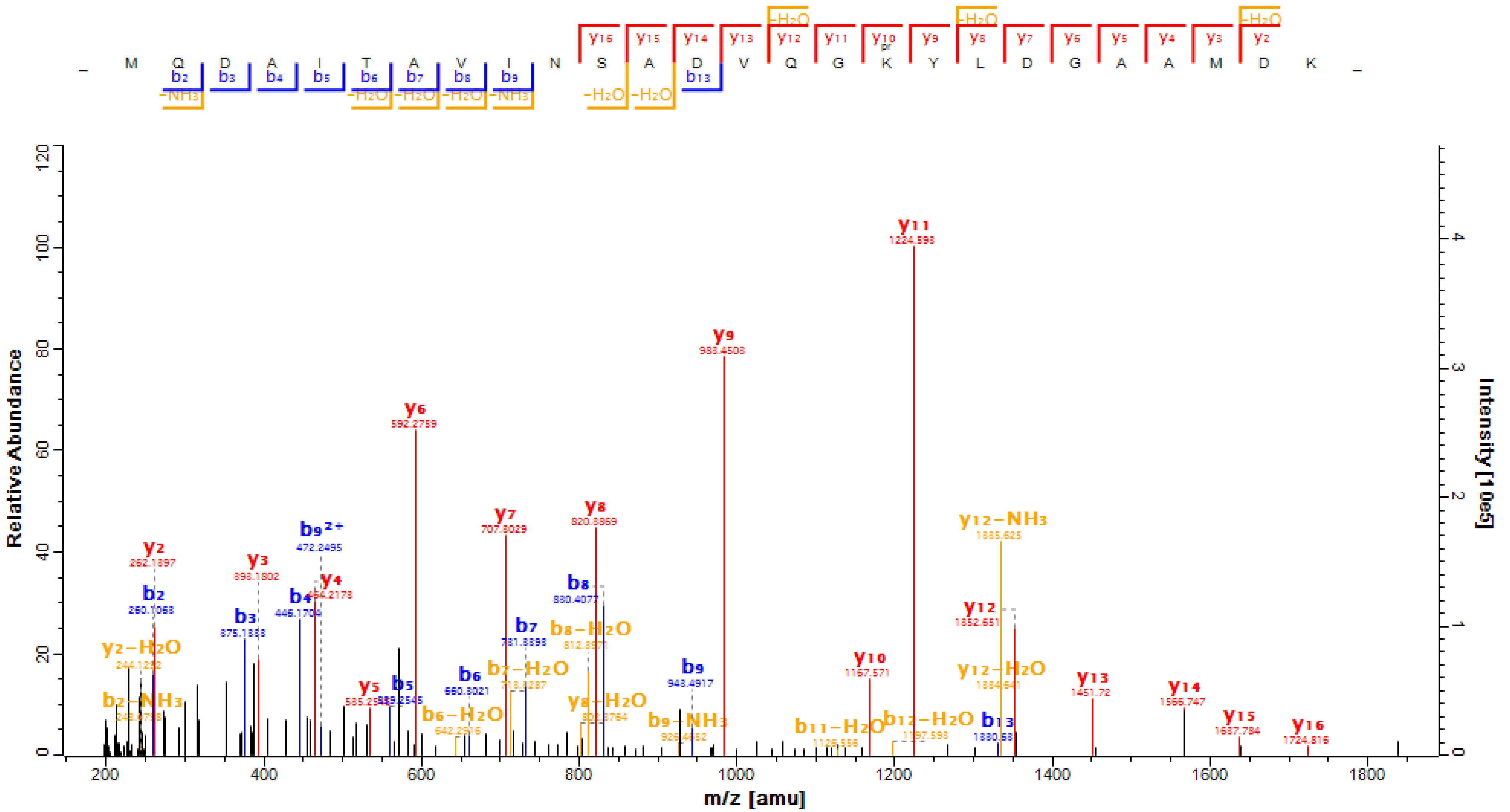

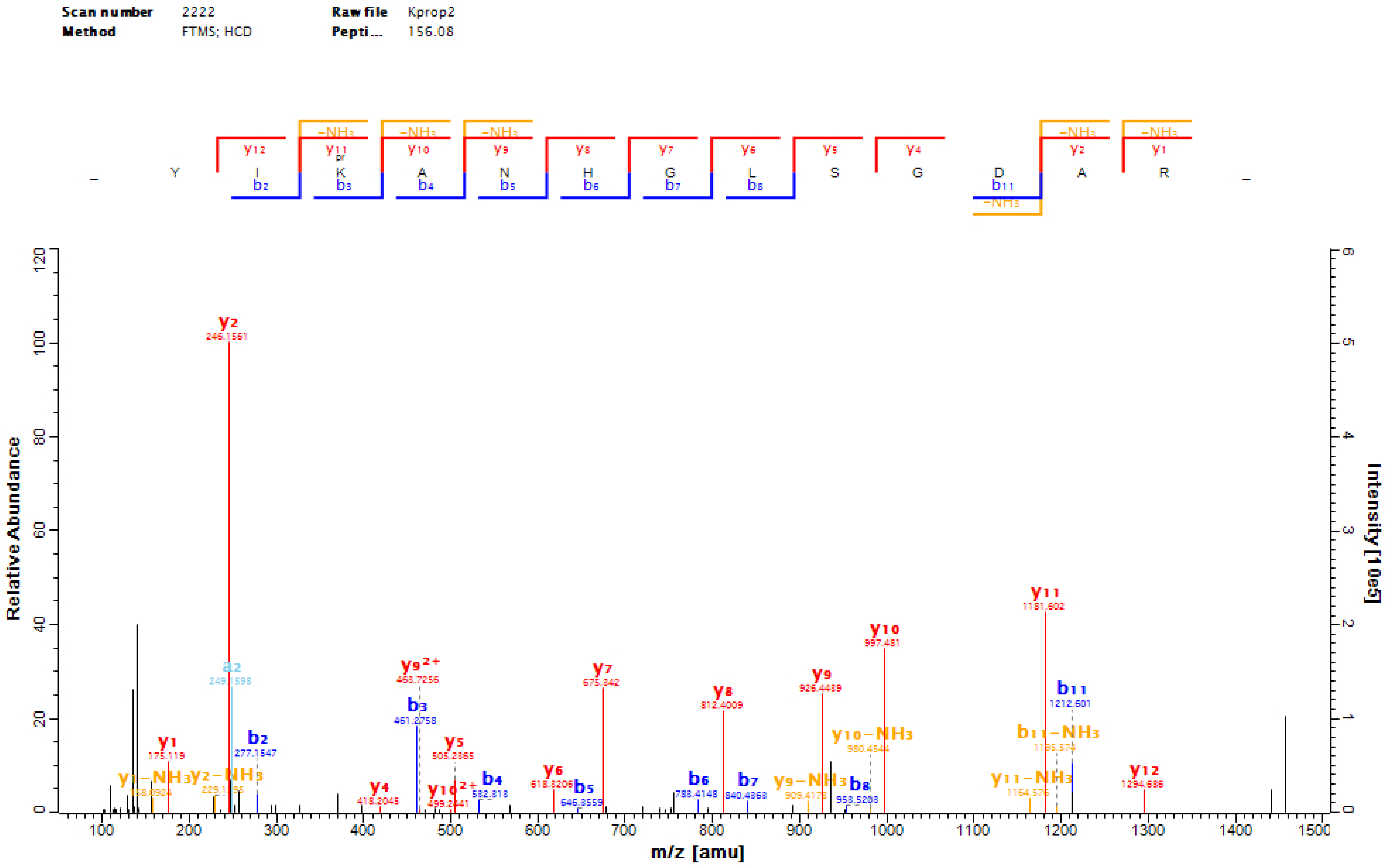

Scan number 2222
Raw file Kprop2
Method FTMS; HCD
Pepti... 156.08
_ Y I K A N H G L S G D A R _
pr
y12 y11 y10 y9 y8 y7 y6 y5 y4 y2 y1
b2 b3 b4 b5 b6 b7 b8 b11
-NH3
y2 246.1561
a2 249.1598
y1 175.119
y1-NH3 158.0924
y2-NH3 229.1296
b2 277.1547
y9 2+ 463.7256
b3 461.2758
y5 505.2865
y4 418.2045
y10 2+ 499.2441
b4 532.313
y6 618.3206
b5 646.3559
y7 675.342
b6 783.4148
b7 840.4863
y8 812.4009
y9-NH3 909.4173
y9 926.4439
b8 953.5208
y10-NH3 980.4544
y10 997.481
y11-NH3 1164.576
y11 1181.602
b11-NH3 1195.574
b11 1212.601
y12 1294.686
Relative Abundance
Intensity [10e5]
m/z [amu]

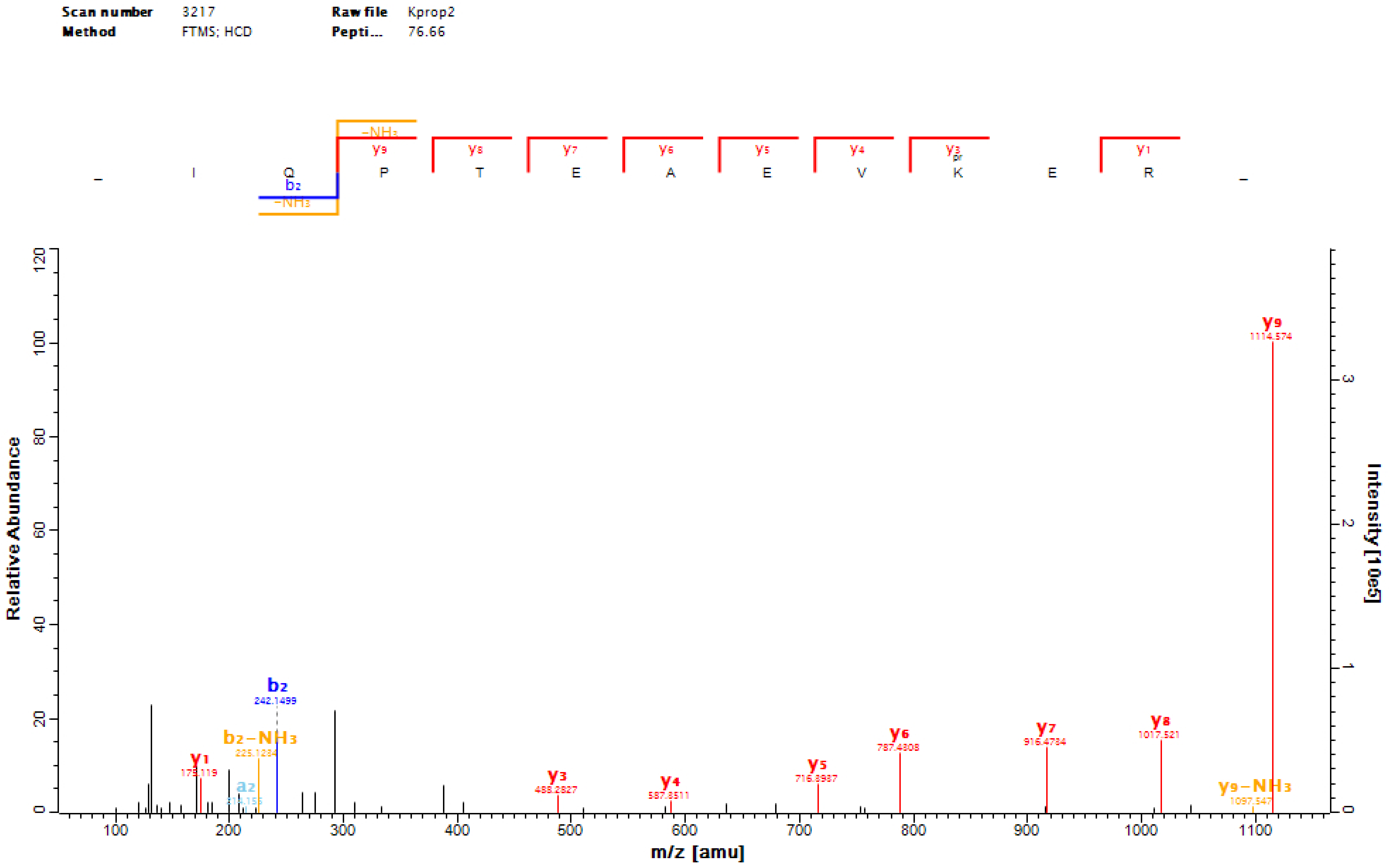
Scan number 3217
Raw file Kprop2
Method FTMS; HCD
Pepti... 76.66
_ I Q P T E A E V K E R _
y9 y8 y7 y6 y5 y4 y3 y1
b2
−NH3
Relative Abundance
Intensity [10e5]
m/z [amu]
y9 1114.574
b2 242.1499
b2−NH3 225.1234
y1 175.119
a2
y3 488.2827
y4 587.3511
y5 716.3937
y6 787.4308
y7 916.4734
y8 1017.521
y9−NH3 1097.547

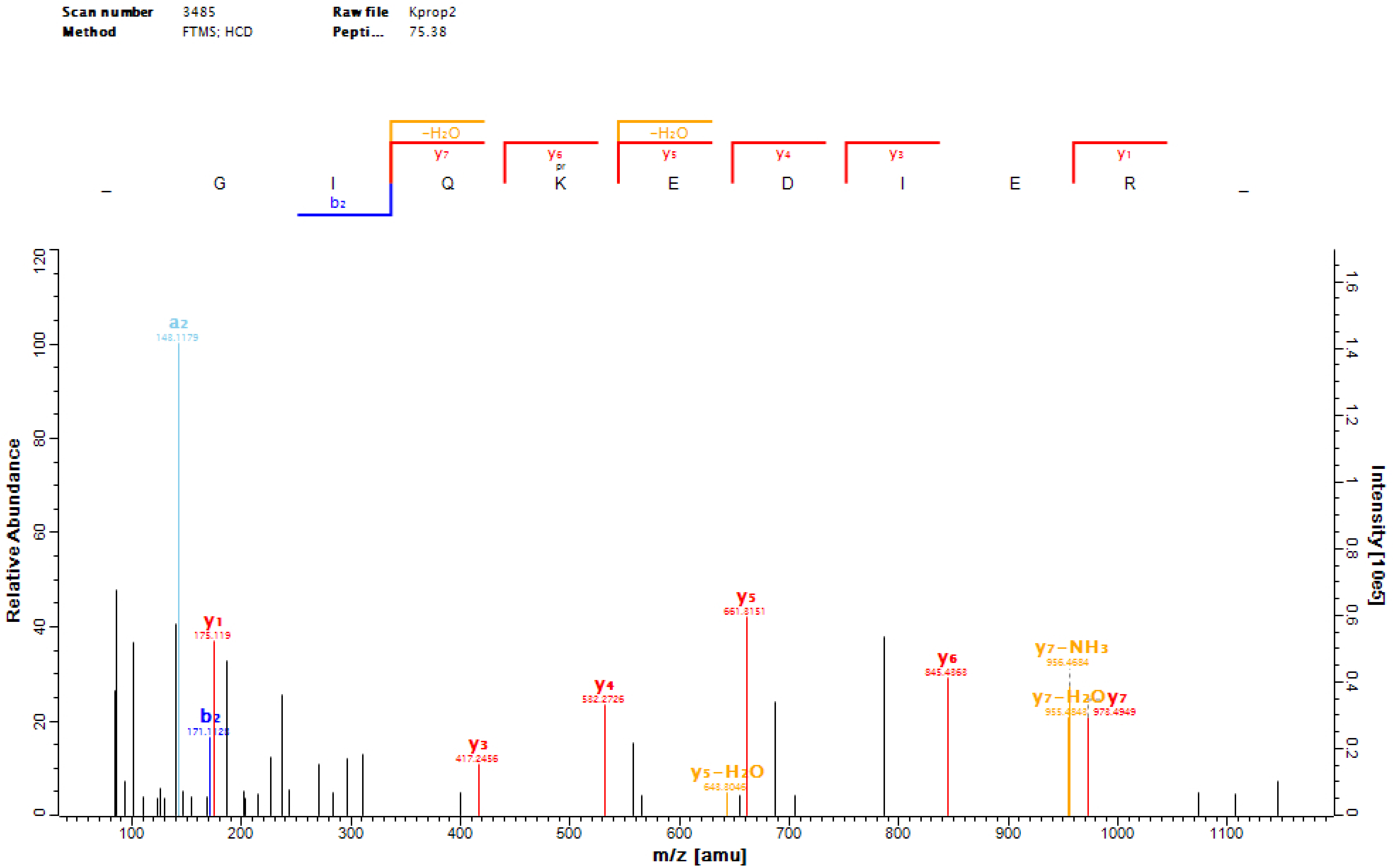
Scan number 3485
Method FTMS; HCD
Raw file Kprop2
Pepti... 75.38
_ G I Q K E D I E R _
a2 143.1179
y1 175.119
b2 171.1128
y3 417.2456
y4 532.2726
y5 661.3151
y5-H2O 643.3046
y6 845.4363
y7-NH3 956.4684
y7-H2O 955.4843
y7 973.4949
Relative Abundance
Intensity [10e5]
m/z [amu]

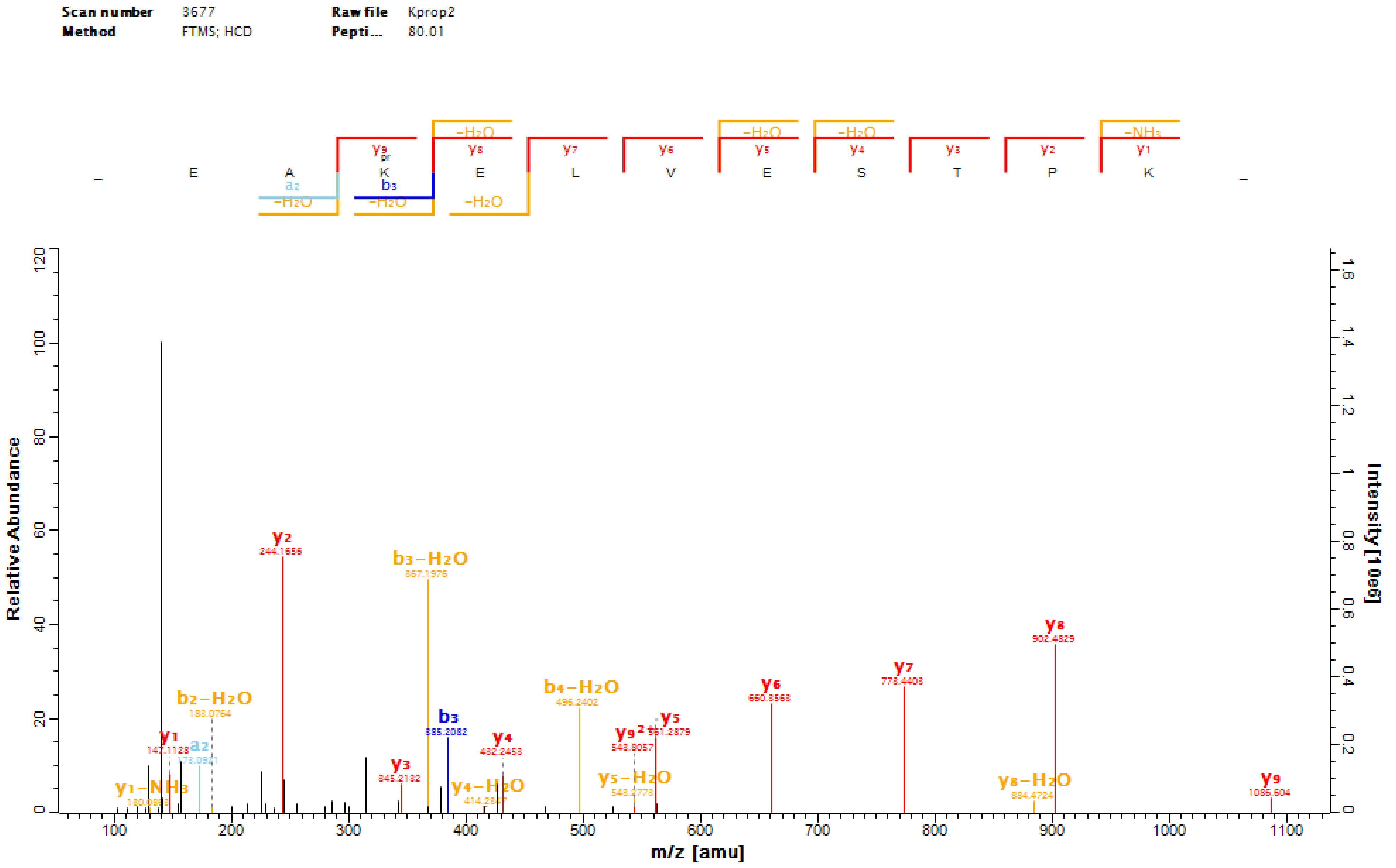

Scan number 3677
Raw file Kprop2
Method FTMS; HCD
Pepti... 80.01
_ E A K E L V E S T P K _
y2
244.1656
b3−H2O
367.1976
y8
902.4829
y7
773.4403
y6
660.3563
b4−H2O
496.2402
b2−H2O
183.0764
b3
385.2082
y5
561.2879
y4
432.2453
y1
y3
345.2182
y9
y4−H2O
y5−H2O
y8−H2O
y1−NH3
Relative Abundance
Intensity [10e6]
m/z [amu]

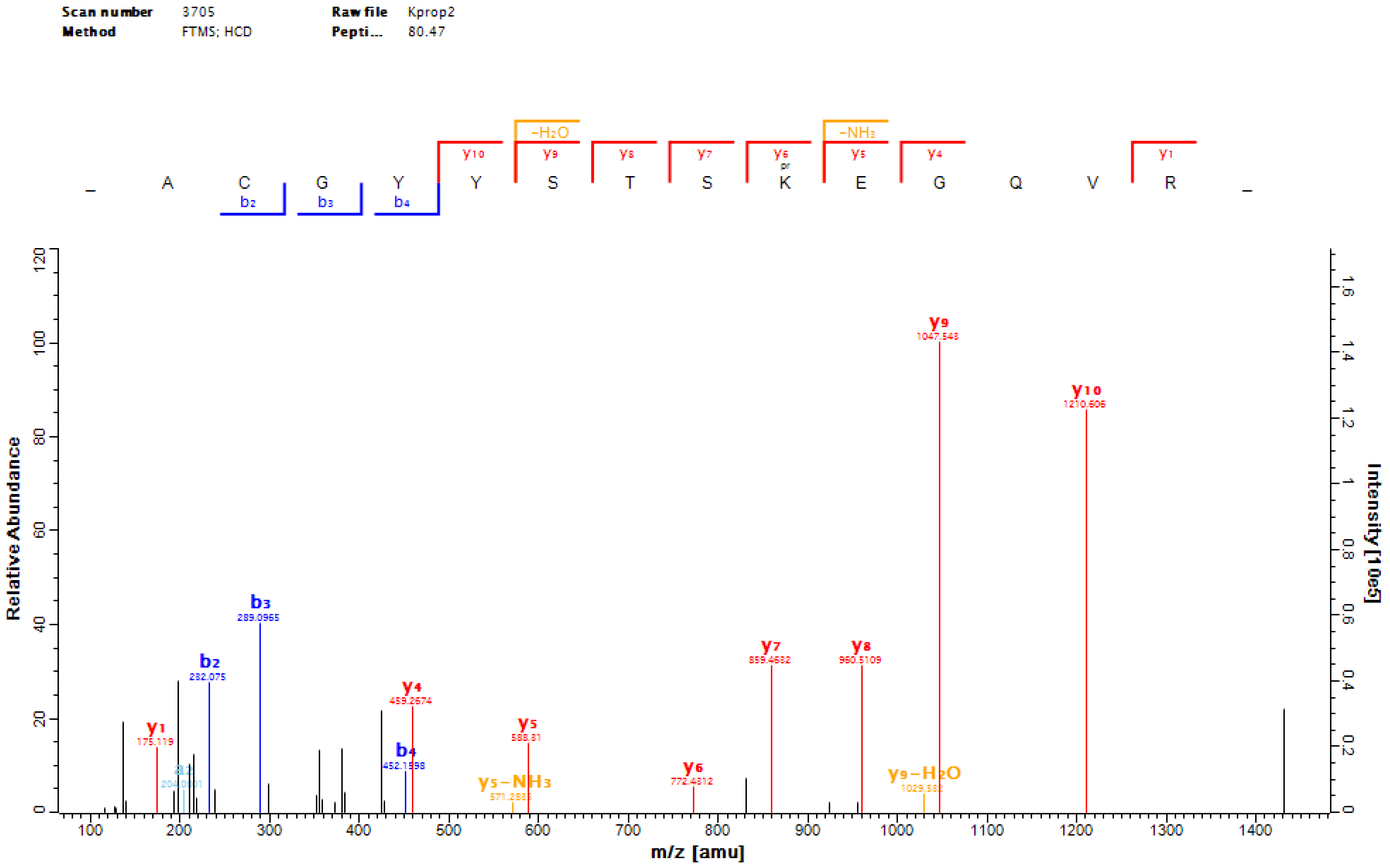
Scan number 3705
Method FTMS; HCD
Raw file Kprop2
Pepti... 80.47
_ A C G Y Y S T S K E G Q V R _
b2 b3 b4
y10 y9 y8 y7 y6 y5 y4 y1
-H2O
-NH3
Relative Abundance
Intensity [10e5]
m/z [amu]
y1 175.119
b2 232.075
b3 289.0965
y4 459.2674
b4 452.1598
y5 588.31
y5-NH3 571.2836
y6 772.4312
y7 859.4632
y8 960.5109
y9 1047.548
y9-H2O 1029.532
y10 1210.606

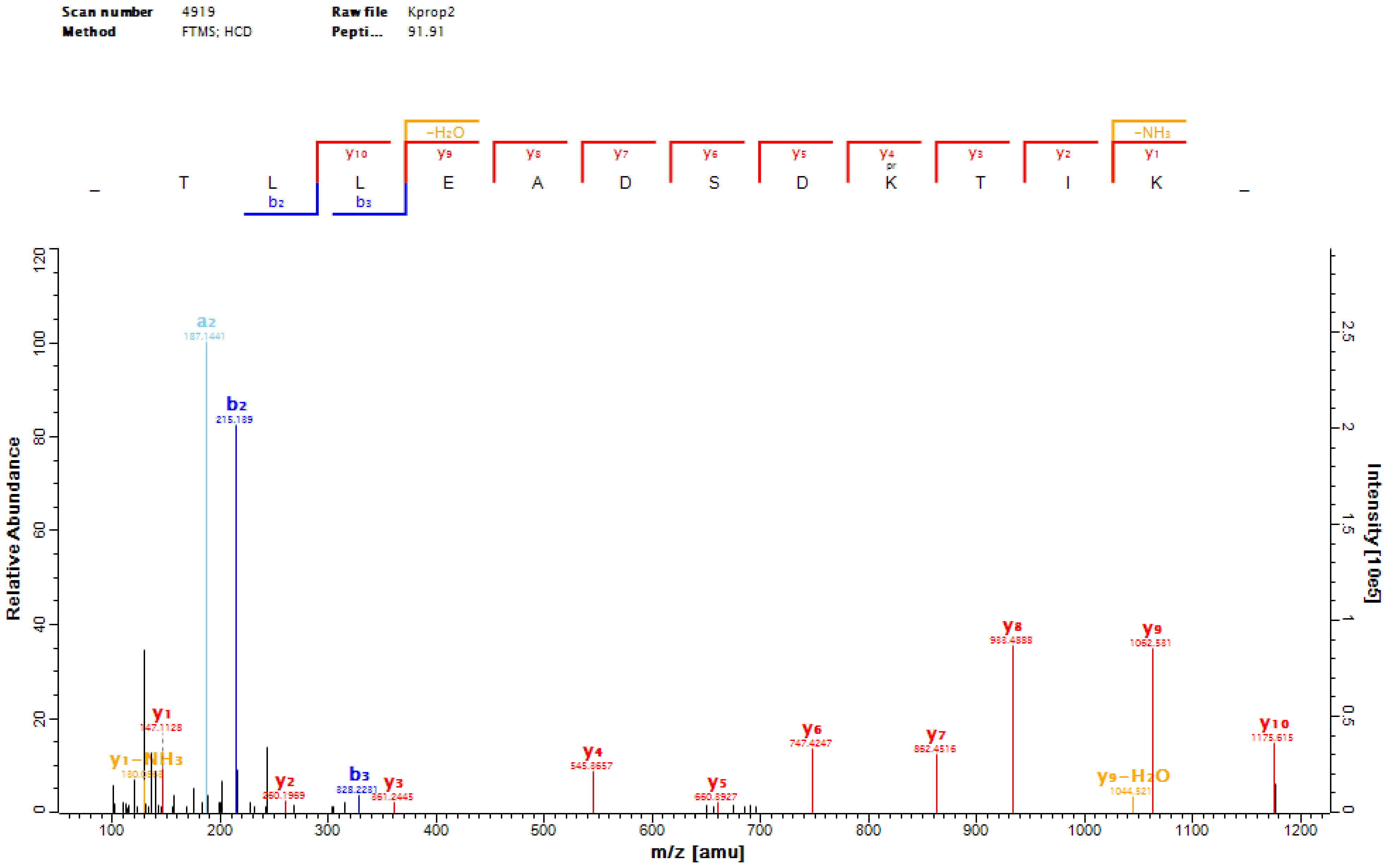

Scan number 4919
Raw file Kprop2
Method FTMS; HCD
Pepti... 91.91
T L L E A D S D K T I K
-H2O
-NH3
y10 y9 y8 y7 y6 y5 y4 y3 y2 y1
b2 b3
a2
187.1441
b2
215.189
y1
147.1128
y1-NH3
y2
y3
b3
y4
y5
y6
747.4247
y7
y8
y9
1062.531
y9-H2O
1044.521
y10
1175.615
Relative Abundance
Intensity [10e5]
m/z [amu]

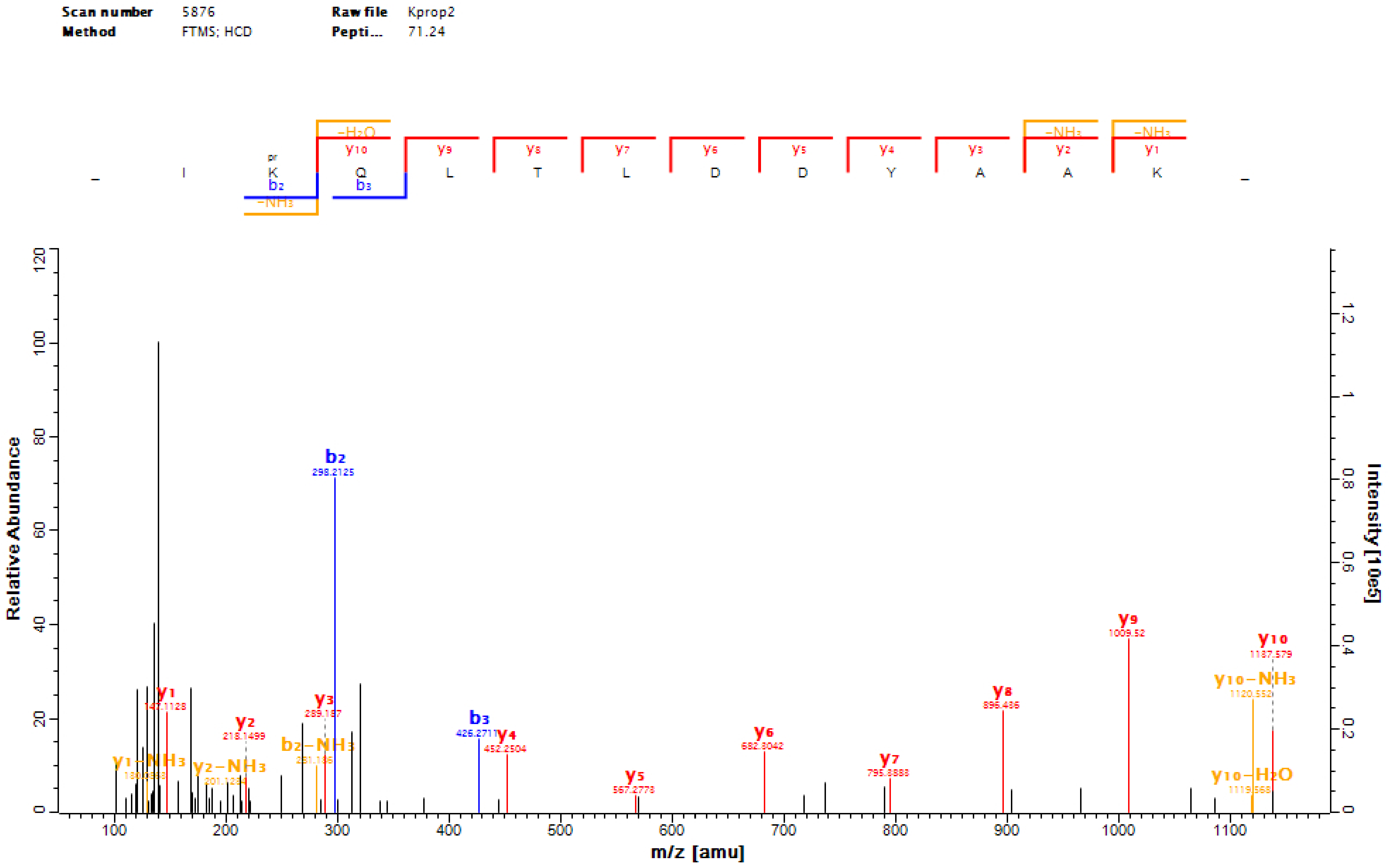

Scan number 5876
Raw file Kprop2
Method FTMS; HCD
Pepti... 71.24
_ I K Q L T L D D Y A A K _
pr
b2 b3
-NH3
-H2O
y10 y9 y8 y7 y6 y5 y4 y3 y2 y1
-NH3 -NH3
Relative Abundance
Intensity [10e5]
m/z [amu]
b2 298.2125
b3 426.2711
y1 147.1128
y2 218.1499
y3 289.187
y4 452.2504
y5 567.2773
y6 682.3042
y7 795.3883
y8 896.436
y9 1009.52
y10 1137.579
y1-NH3 130.0863
y2-NH3 201.1234
b2-NH3 281.186
y10-NH3 1120.552
y10-H2O 1119.568

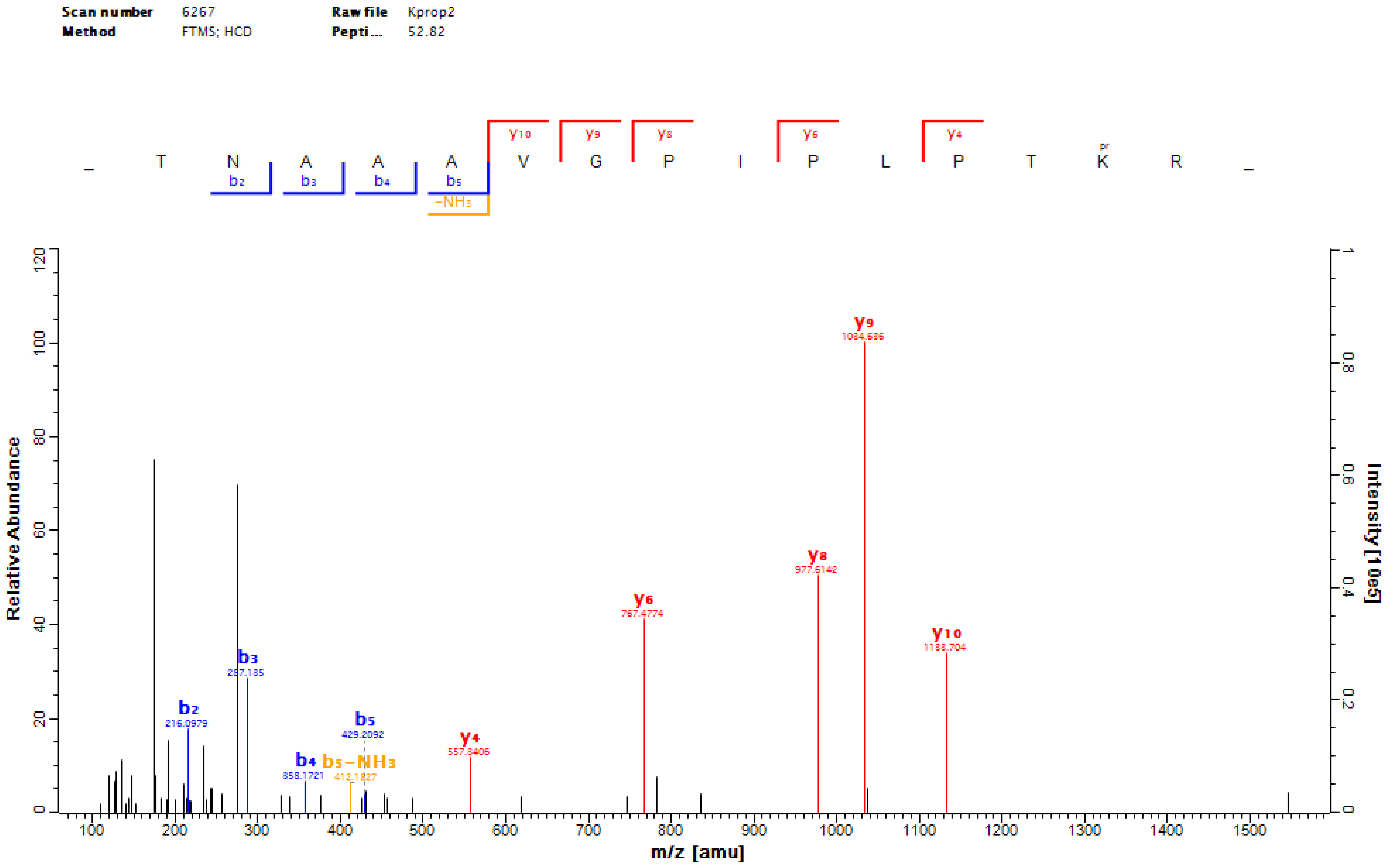

Scan number 6267
Method FTMS; HCD
Raw file Kprop2
Pepti... 52.82
_ T N A A A V G P I P L P T K R _
pr
b2 b3 b4 b5
−NH3
y10 y9 y8 y6 y4
Relative Abundance
Intensity [10e5]
m/z [amu]
b2 216.0979
b3 287.135
b4 358.1721
b5−NH3 412.1827
b5 429.2092
y4 557.3406
y6 767.4774
y8 977.6142
y9 1034.636
y10 1133.704

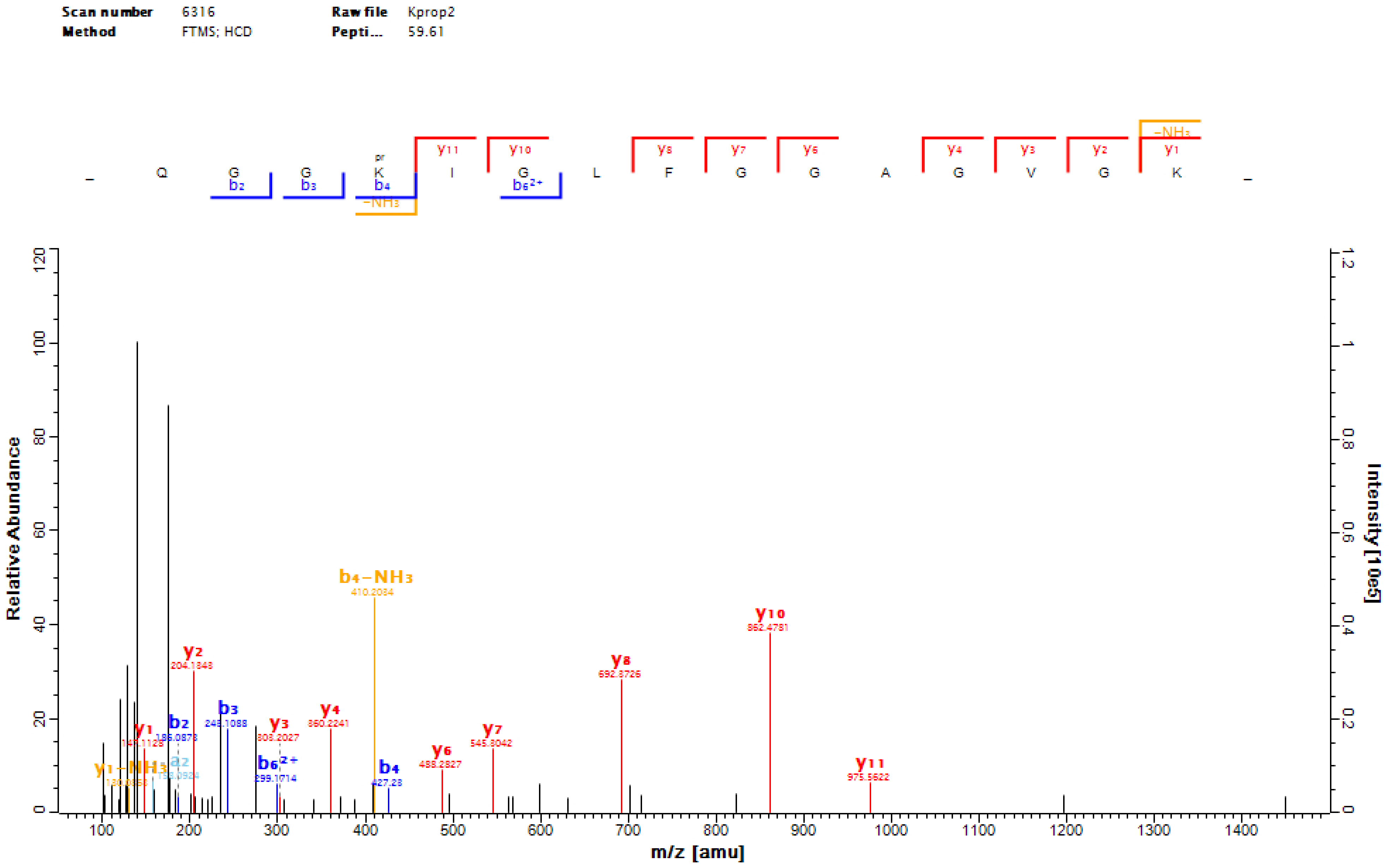

Scan number 6316
Raw file Kprop2
Method FTMS; HCD
Pepti... 59.61
Q G G K I G L F G G A G V G K
y11 y10 y8 y7 y6 y4 y3 y2 y1
b2 b3 b4 b6²⁺
−NH3
b4−NH3 410.2084
y10 862.4781
y8 692.3726
y2 204.1343
b3 243.1088
y4 360.2241
y3 303.2027
y7 545.3042
y1 147.1128
b2 186.0873
y6 488.2827
y11 975.5622
b6²⁺ 299.1714
b4 427.23
y1−NH3 130.0863
a2 158.0924
Relative Abundance
Intensity [10e5]
m/z [amu]

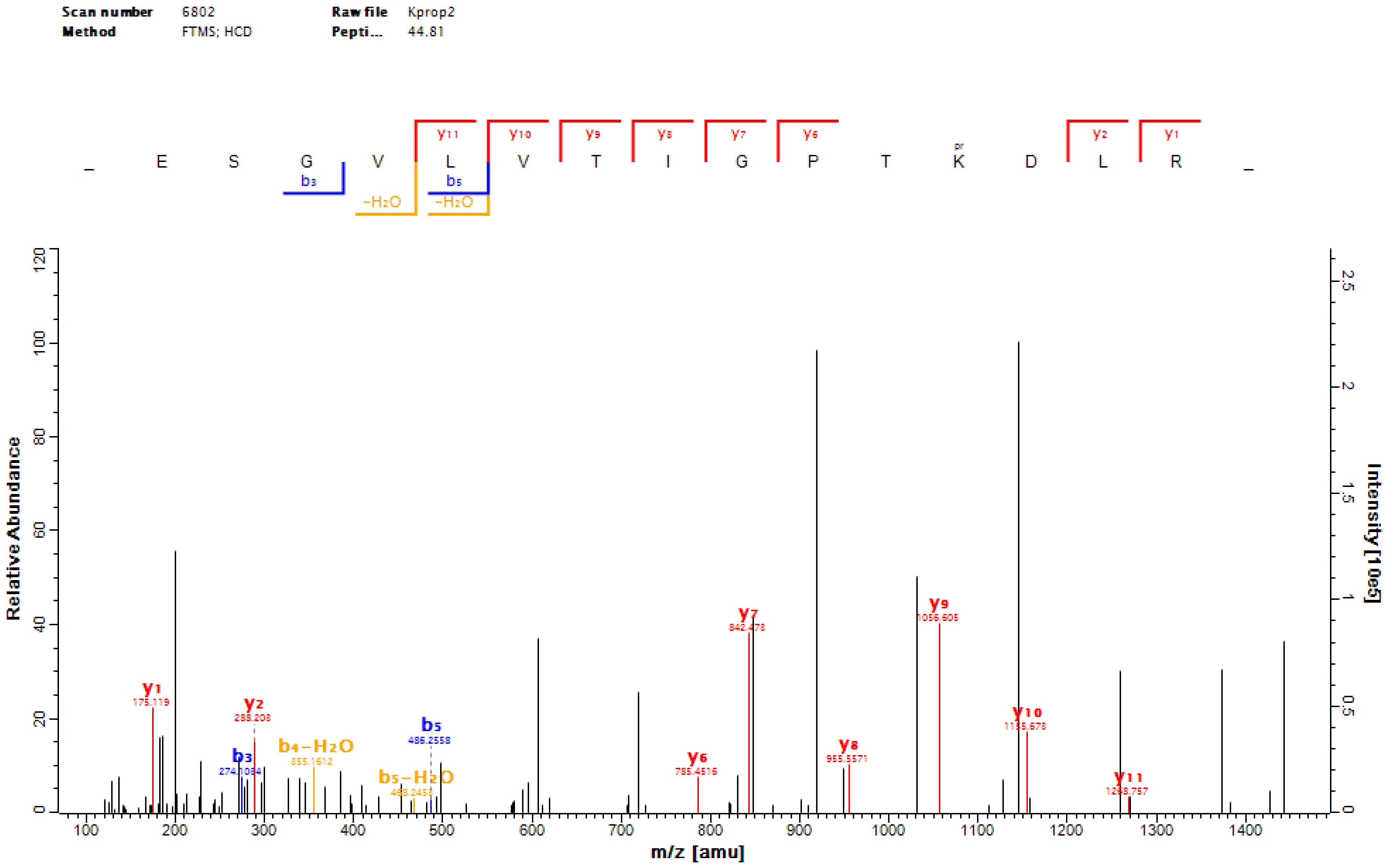
Scan number 6802
Method FTMS; HCD
Raw file Kprop2
Pepti... 44.81
_ E S G V L V T I G P T K D L R _
pr
y11 y10 y9 y8 y7 y6 y2 y1
b3 b5
-H2O -H2O
y1 175.119
y2 288.203
b3 274.1084
b4-H2O 355.1612
b5-H2O 468.2453
b5 486.2558
y6 785.4516
y7 842.473
y8 955.5571
y9 1056.605
y10 1155.673
y11 1268.757
Relative Abundance
Intensity [10e5]
m/z [amu]

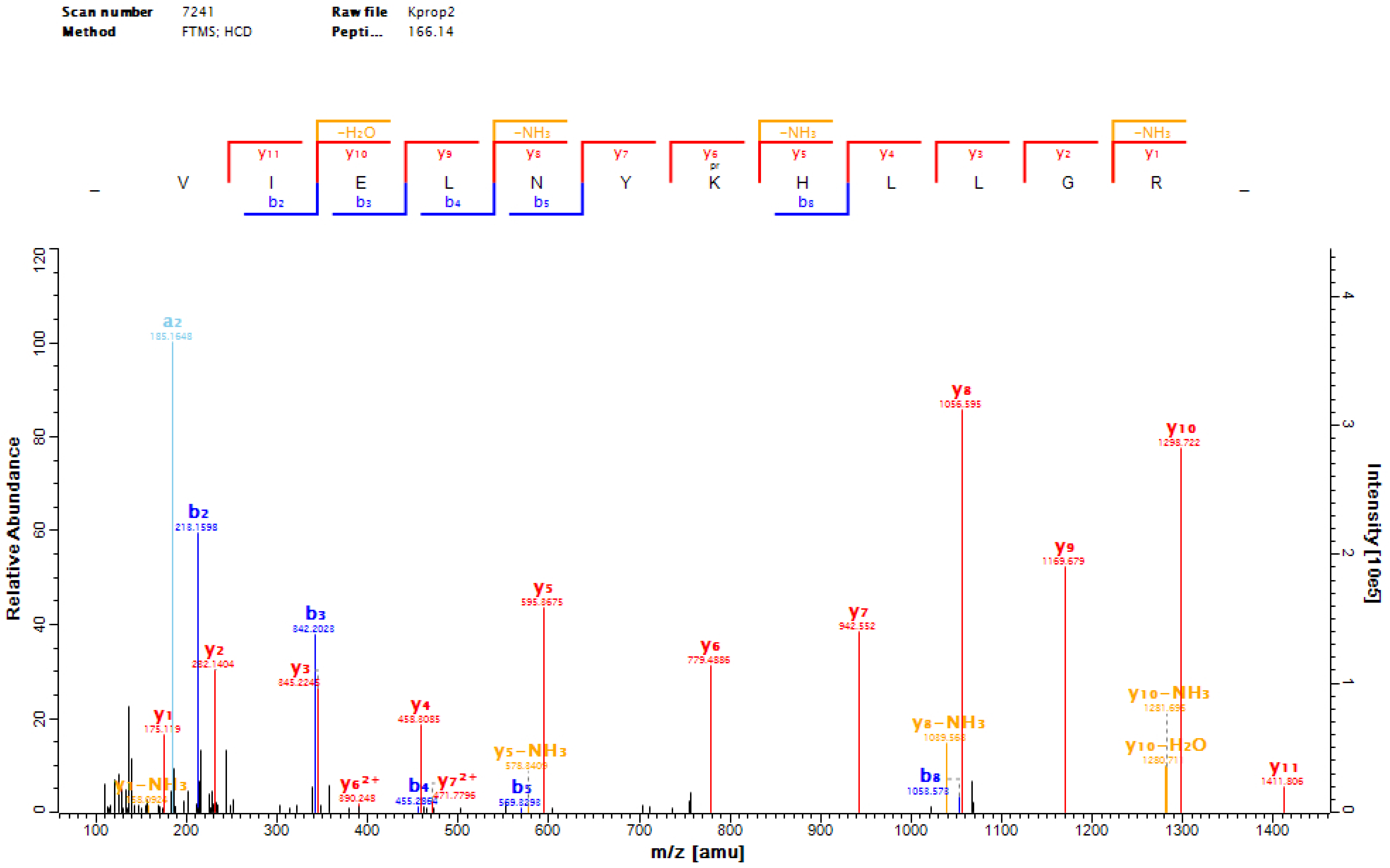

Scan number 7241
Raw file Kprop2
Method FTMS; HCD
Pepti... 166.14
_ V I E L N Y K H L L G R _
Relative Abundance
Intensity [10e5]
m/z [amu]
a2 185.1648
b2 213.1598
b3 342.2023
y1 175.119
y2 232.1404
y3 345.2245
y4 458.3085
y5 595.3675
y6 779.4886
y7 942.552
y8 1056.595
y9 1169.679
y10 1298.722
y11 1411.806
y5−NH3 578.3409
y8−NH3 1039.568
y10−NH3 1281.695
y10−H2O 1280.711
b8 1053.573

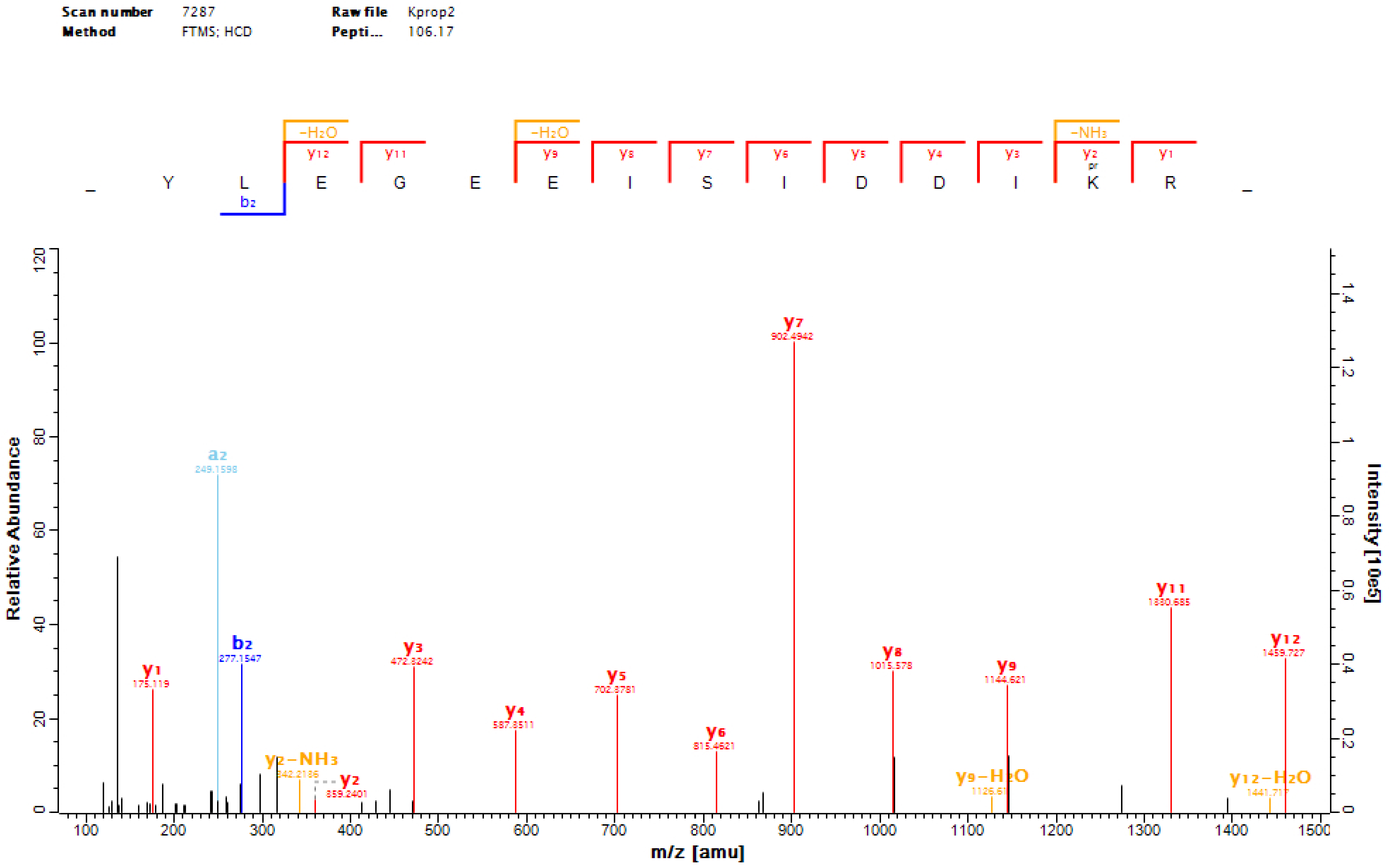
Scan number 7287
Method FTMS; HCD
Raw file Kprop2
Pepti... 106.17
_ Y L E G E E I S I D D I K R _
b2
y12 y11 y9 y8 y7 y6 y5 y4 y3 y2 y1
-H2O -H2O -NH3
Relative Abundance
Intensity [10e5]
m/z [amu]
y7 902.4942
a2 249.1598
y11 1330.685
y12 1459.727
b2 277.1547
y1 175.119
y3 472.3242
y8 1015.578
y9 1144.621
y5 702.3781
y4 587.3511
y6 815.4621
y2-NH3 342.2136
y2 359.2401
y9-H2O 1126.61
y12-H2O 1441.717

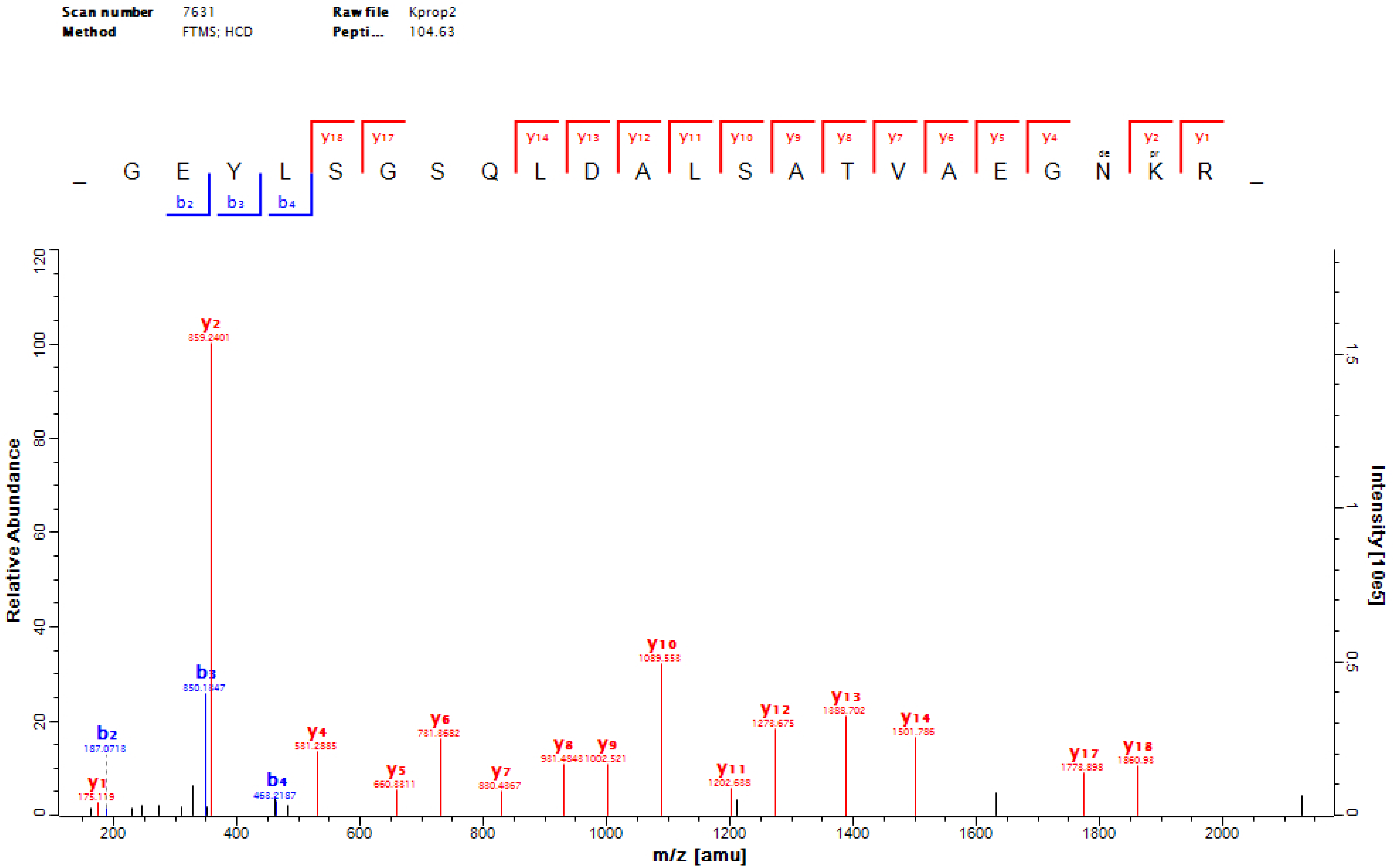

Scan number 7631
Method FTMS; HCD
Raw file Kprop2
Pepti... 104.63
_ G E Y L S G S Q L D A L S A T V A E G N K R _
Relative Abundance
Intensity [10e5]
m/z [amu]
y1
b2
b3
b4
y2
y4
y5
y6
y7
y8
y9
y10
y11
y12
y13
y14
y17
y18

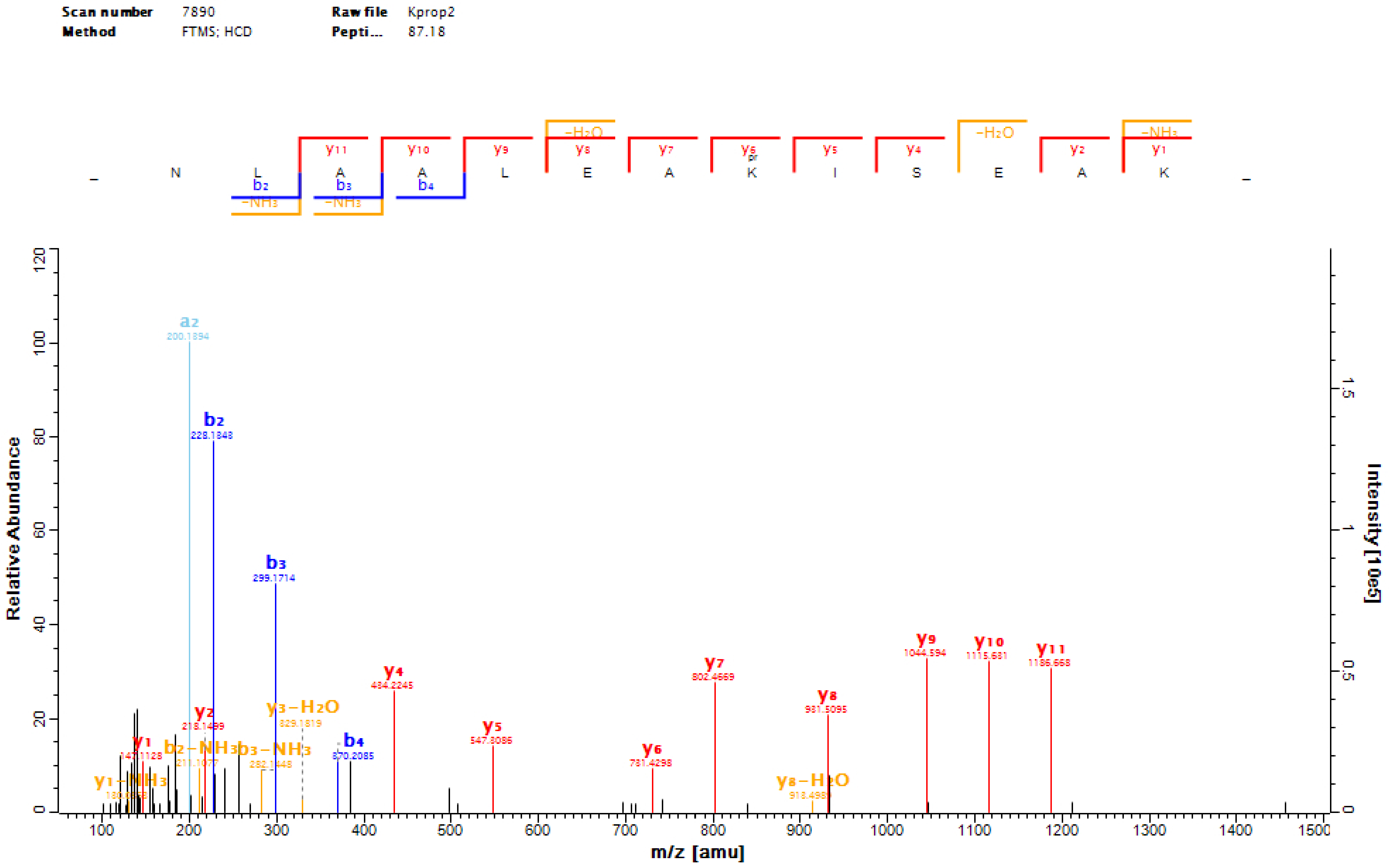
Scan number 7890
Method FTMS; HCD
Raw file Kprop2
Pepti... 87.18
_ N L A A L E A K I S E A K _
Relative Abundance
Intensity [10e5]
m/z [amu]
a2 200.1394
b2 228.1343
b3 299.1714
b4 370.2085
y1 147.1128
y2 218.1499
y4 434.2245
y5 547.3086
y6 731.4298
y7 802.4669
y8 931.5095
y9 1044.594
y10 1115.631
y11 1186.668
y3−H2O 329.1819
y8−H2O 913.4989
b2−NH3 211.1077
b3−NH3 282.1448
y1−NH3

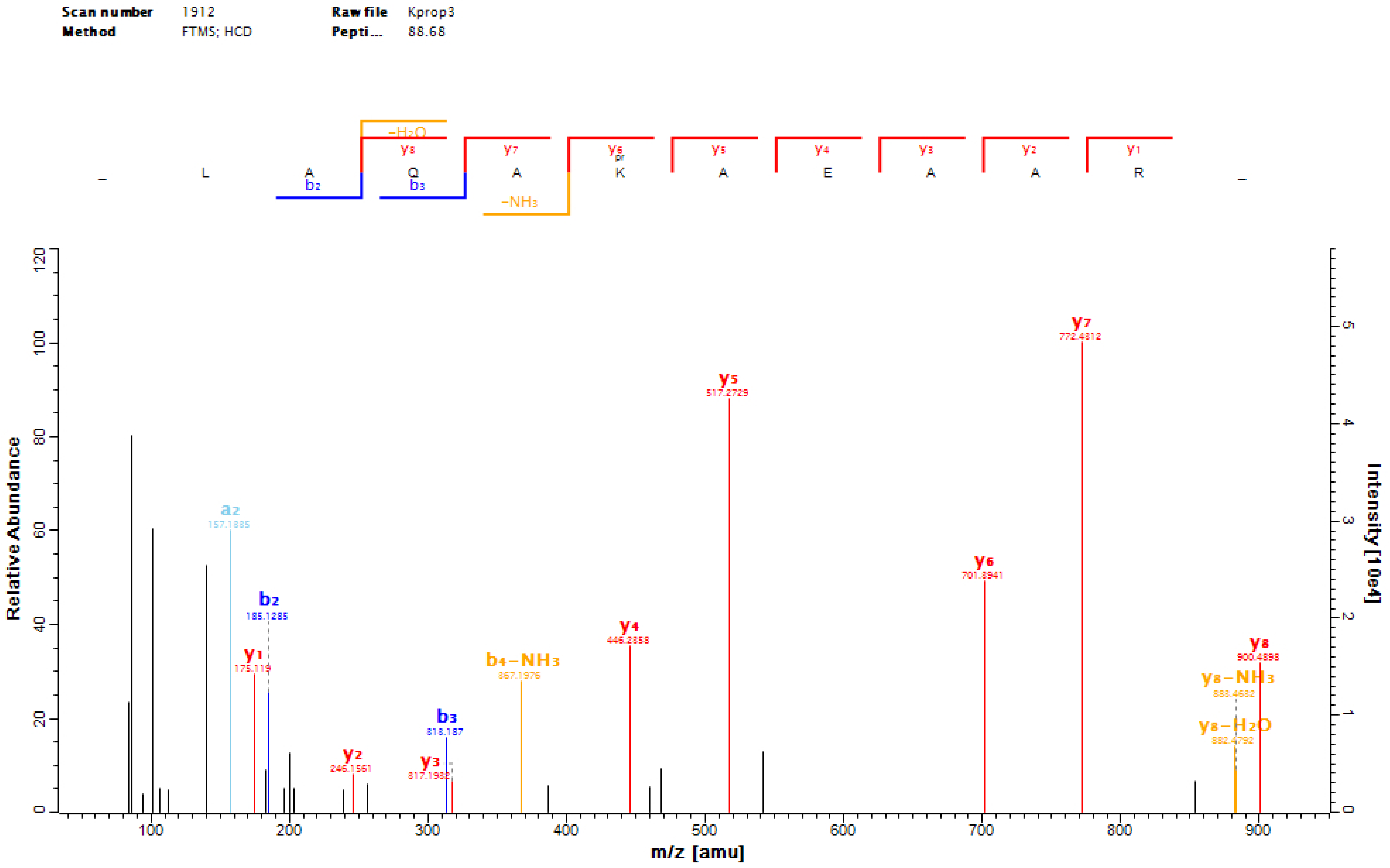

Scan number 1912
Method FTMS; HCD
Raw file Kprop3
Pepti... 88.68
_ L A Q A K A E A A R _
b2 b3
y8 y7 y6 y5 y4 y3 y2 y1
−H2O
−NH3
a2 157.1885
b2 185.1285
y1 175.119
y2 246.1561
y3 317.1932
b3 313.187
b4−NH3 367.1976
y4 446.2358
y5 517.2729
y6 701.3941
y7 772.4312
y8 900.4898
y8−NH3 883.4632
y8−H2O 882.4792
Relative Abundance
Intensity [10e4]
m/z [amu]

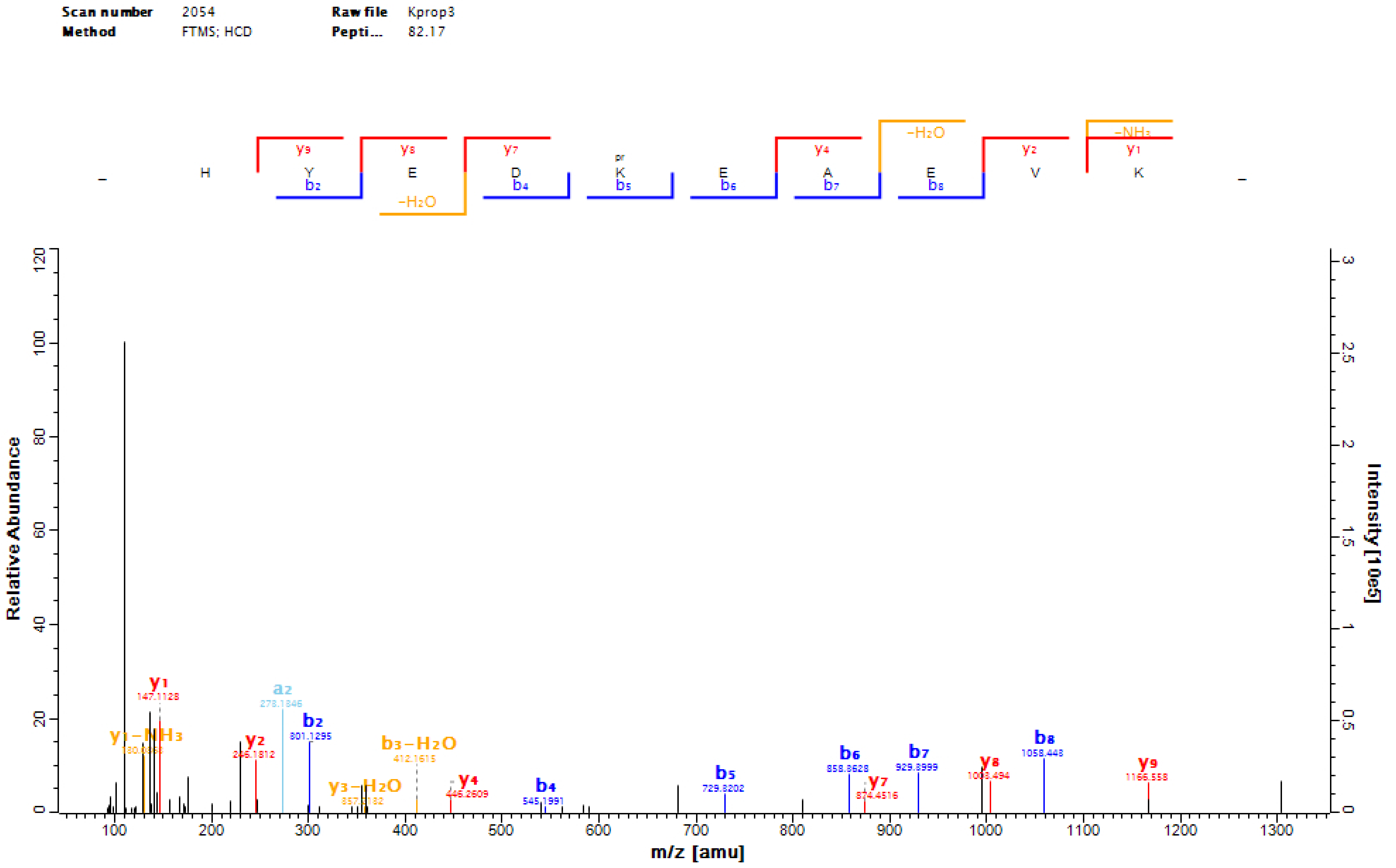
Scan number 2054
Method FTMS; HCD
Raw file Kprop3
Pepti... 82.17
y9 y8 y7 y4 y2 y1
-H2O -NH3
H Y E D K E A E V K
pr
b2 b4 b5 b6 b7 b8
Relative Abundance
Intensity [10e5]
m/z [amu]
y1 147.1128
y1-NH3 130.0863
y2 246.1812
a2 273.1346
b2 301.1295
b3-H2O 412.1615
y3-H2O 357.2132
y4 446.2609
b4 545.1991
b5 729.3202
b6 858.3628
y7 874.4516
b7 929.3999
y8 1003.494
b8 1058.443
y9 1166.558

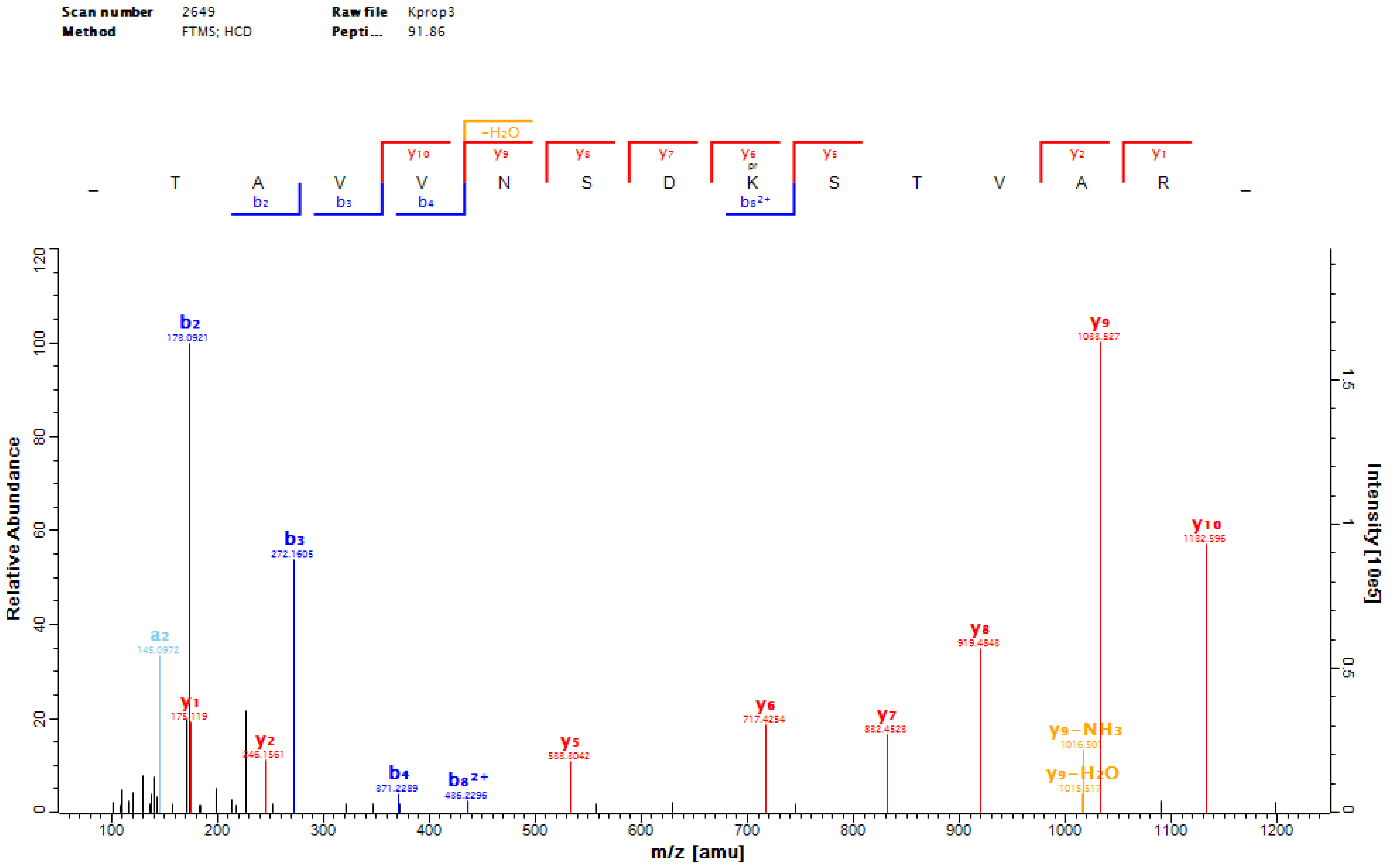

Scan number 2649
Raw file Kprop3
Method FTMS; HCD
Pepti... 91.86
_ T A V V N S D K S T V A R _
b2 b3 b4 b8 2+
y10 y9 y8 y7 y6 y5 y2 y1
-H2O
pr
b2 178.0921
b3 272.1605
a2 145.0972
y1 175.119
y2 246.1561
b4 371.2289
b8 2+ 436.2296
y5 533.3042
y6 717.4254
y7 832.4523
y8 919.4843
y9 1033.527
y10 1132.595
y9−NH3 1016.501
y9−H2O 1015.517
Relative Abundance
Intensity [10e5]
m/z [amu]

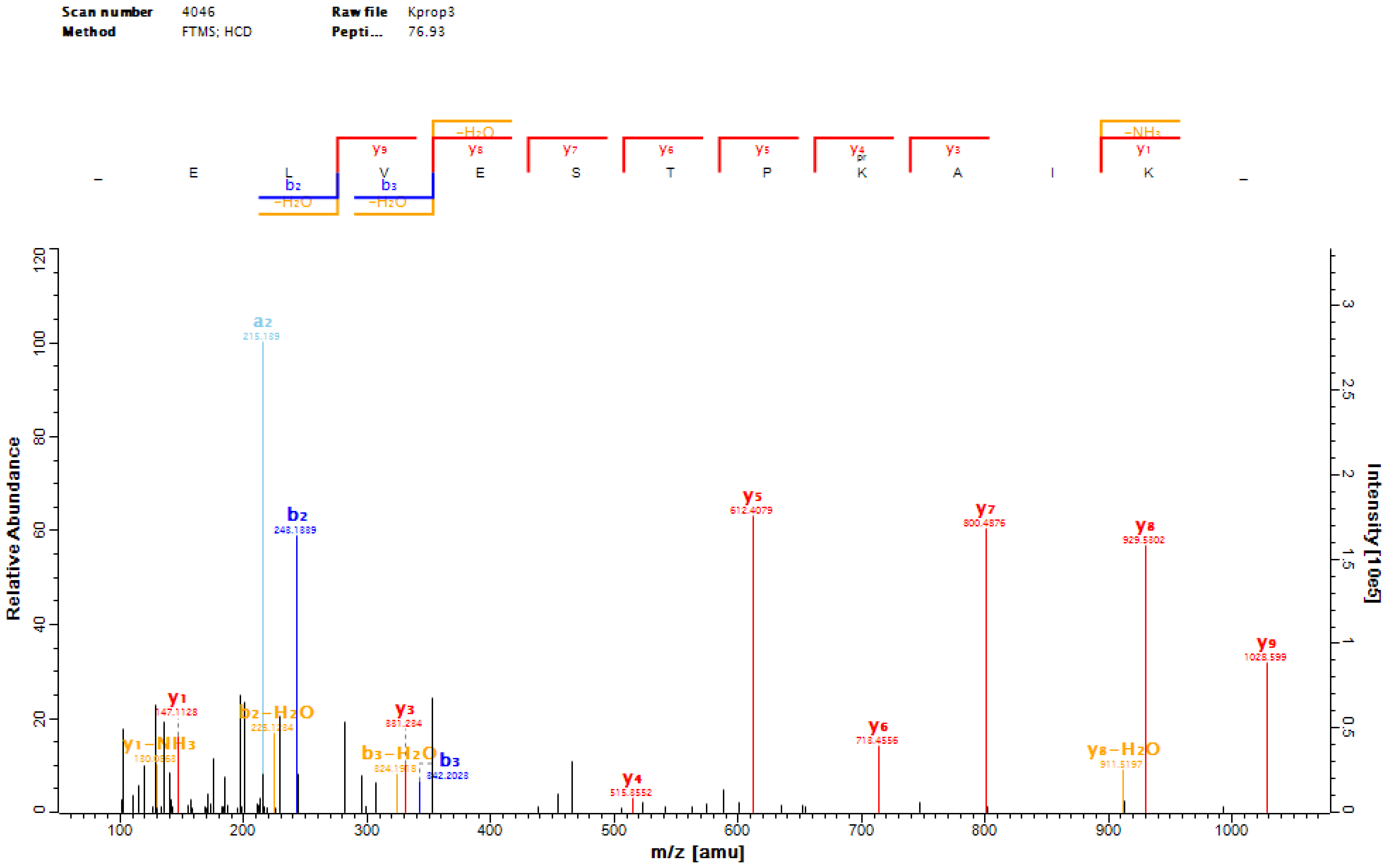

Scan number 4046
Raw file Kprop3
Method FTMS; HCD
Pepti... 76.93
_ E L V E S T P K A I K _
y9 y8 y7 y6 y5 y4 y3 y1
pr
−H2O
−NH3
b2 b3
−H2O −H2O
a2 215.189
b2 243.1889
y5 612.4079
y7 800.4876
y8 929.5302
y9 1028.599
y1 147.1128
b2−H2O 225.1284
y3 331.234
y1−NH3 130.0863
b3−H2O 324.1918
b3 342.2023
y6 713.4556
y8−H2O 911.5197
y4 515.3552
Relative Abundance
Intensity [10e5]
m/z [amu]

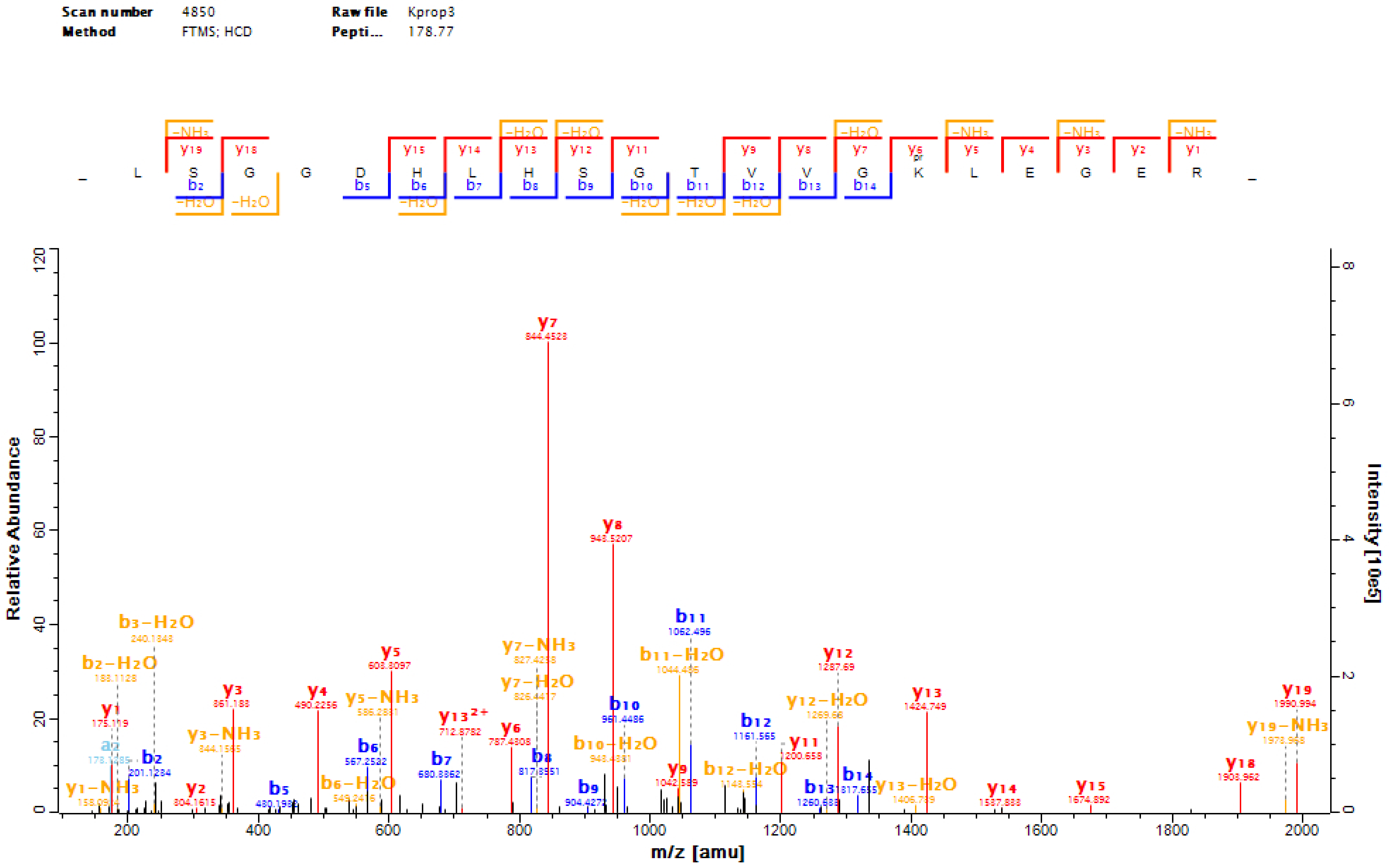

Scan number 4850
Raw file Kprop3
Method FTMS; HCD
Pepti... 178.77
_ L S G G D H L H S G T V V G K L E G E R _
y7 844.4523
y8 945.5207
b11 1062.496
b3−H2O 240.1343
b2−H2O 183.1128
y1 175.119
y3 361.183
y4 490.2256
y5 603.3097
y5−NH3 586.2831
y7−NH3 827.4258
y7−H2O 826.4417
b11−H2O 1044.486
y12 1287.69
y12−H2O 1269.68
y13 1424.749
y19 1990.994
y19−NH3 1973.968
y13 2+ 712.8782
y6 787.4808
b10 961.4486
b10−H2O 943.4381
b12 1161.565
y11 1200.658
b12−H2O 1143.554
y9 1042.589
b6 567.2522
b7 680.3362
b8 817.3951
b9 904.4272
b13 1260.633
b14 1317.655
y13−H2O 1406.739
y14 1537.833
y15 1674.892
y18 1903.952
a2 173.1285
b2 201.1284
y3−NH3 344.1565
y2 304.1615
b5 430.1986
b6−H2O 549.2416
y1−NH3 158.0924
Relative Abundance
Intensity [10e5]
m/z [amu]

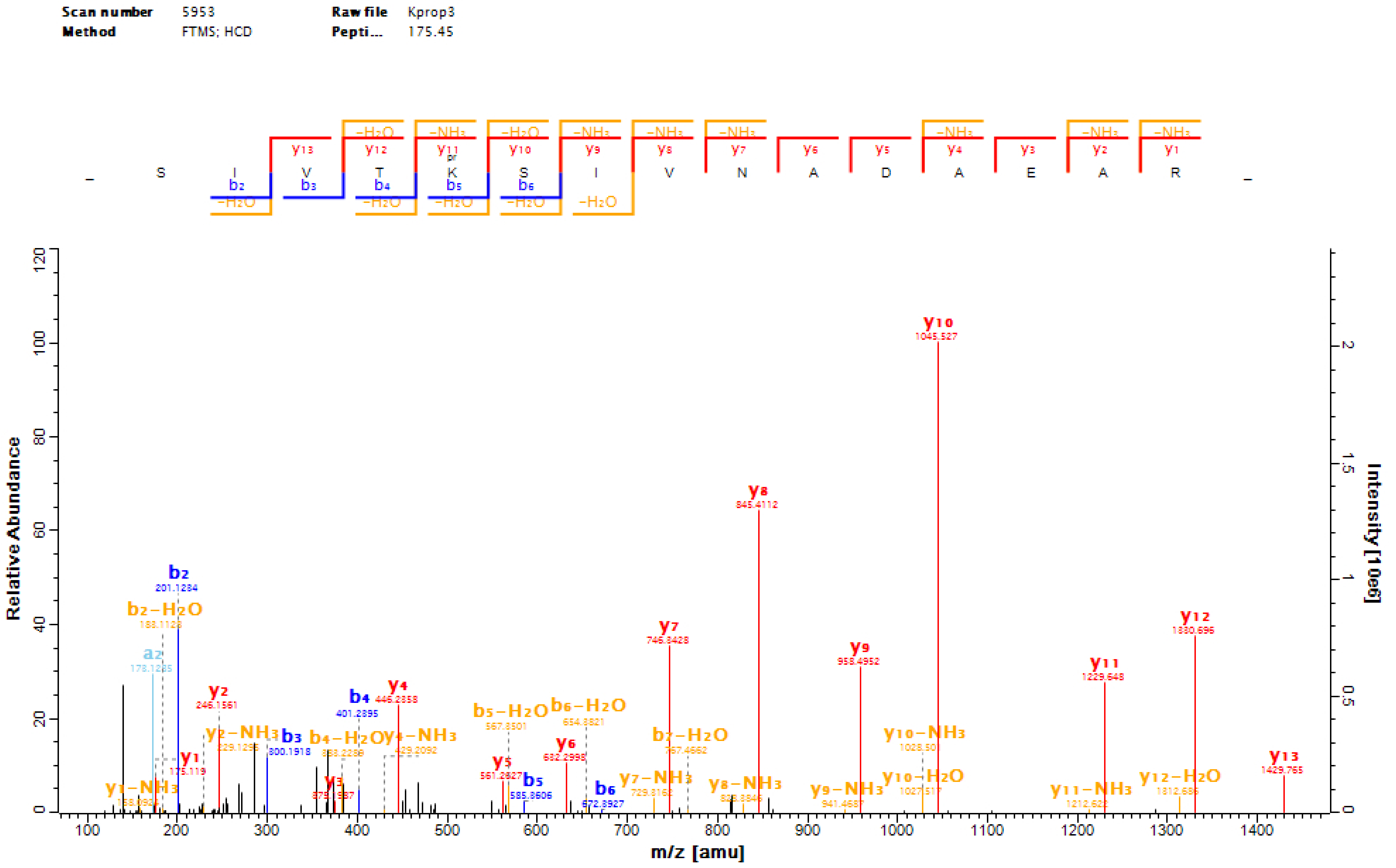

Scan number 5953
Method FTMS; HCD
Raw file Kprop3
Pepti... 175.45
_ S I V T K S I V N A D A E A R _
y13 y12 y11 y10 y9 y8 y7 y6 y5 y4 y3 y2 y1
b2 b3 b4 b5 b6
-H2O
-NH3
pr
Relative Abundance
Intensity [10e6]
m/z [amu]
y10
1045.527
y8
845.4112
y7
746.3428
y12
1330.696
y9
958.4952
y11
1229.648
y13
1429.765
b2
201.1234
b2-H2O
183.1128
a2
173.1285
y2
246.1561
y2-NH3
229.1295
y1
175.119
y1-NH3
b3
300.1918
b4
401.2395
y4
446.2358
b4-H2O
y4-NH3
429.2092
y3
b5-H2O
567.3501
b6-H2O
654.3821
y6
632.2998
y5
561.2627
b5
585.3606
b6
672.3927
b7-H2O
767.4662
y7-NH3
729.3162
y8-NH3
y9-NH3
y10-NH3
1028.50
y10-H2O
1027.517
y11-NH3
1212.622
y12-H2O
1312.686

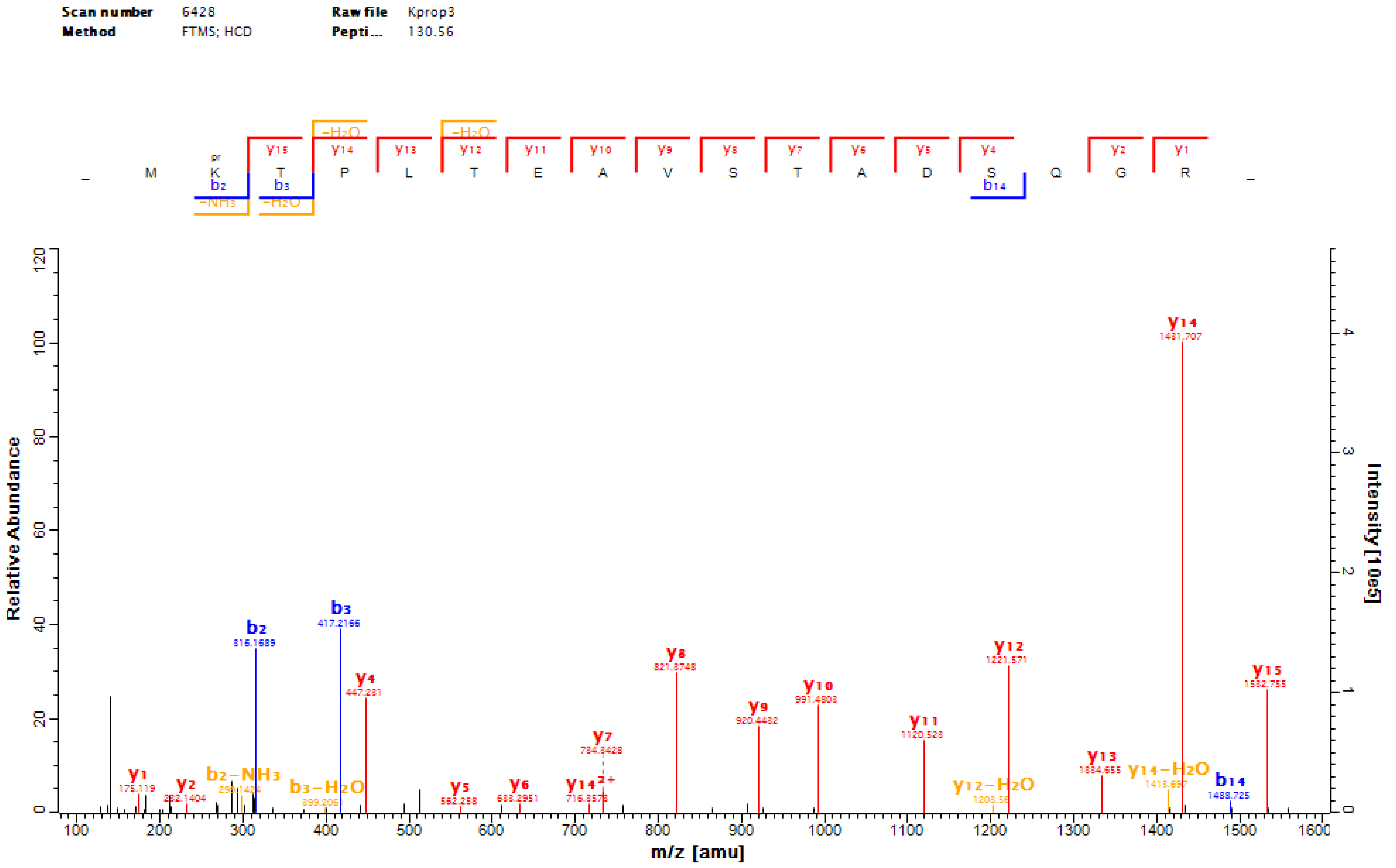
Scan number 6428
Raw file Kprop3
Method FTMS; HCD
Pepti... 130.56
_ M K T P L T E A V S T A D S Q G R _
pr
y15 y14 y13 y12 y11 y10 y9 y8 y7 y6 y5 y4 y2 y1
-H2O
b2 b3 b14
-NH3
y14
y12
y15
b3
b2
y4
y8
y10
y9
y11
y7
y13
y14-H2O
b14
y12-H2O
y1
y2
b2-NH3
b3-H2O
y5
y6
y14 2+
Relative Abundance
Intensity [10e5]
m/z [amu]

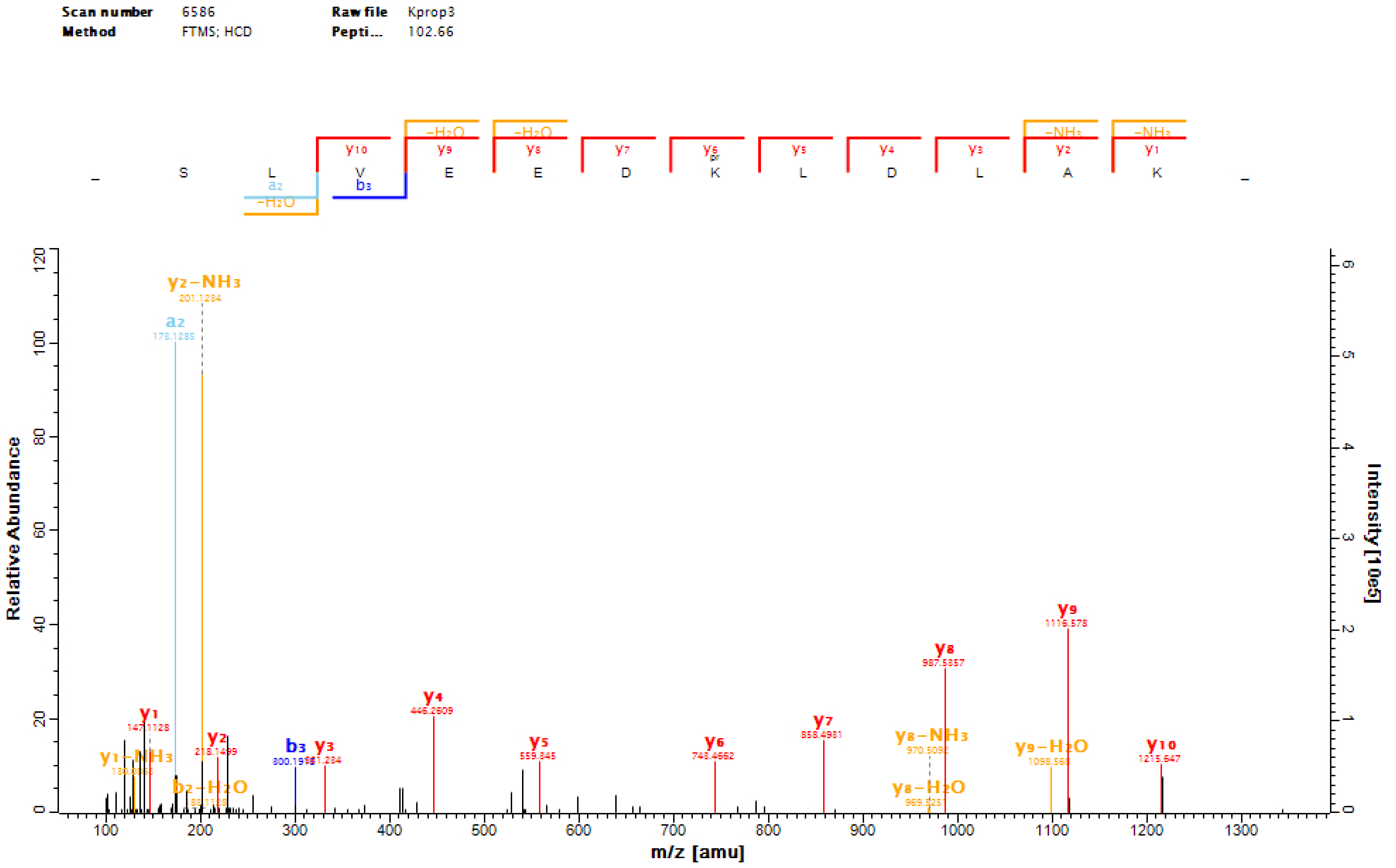
Scan number 6586
Raw file Kprop3
Method FTMS; HCD
Pepti... 102.66
_ S L V E E D K L D L A K _
a2
-H2O
b3
y10 y9 y8 y7 y6 y5 y4 y3 y2 y1
-H2O -H2O -NH3 -NH3
y2-NH3
201.1284
a2
178.1285
y1
147.1128
y1-NH3
y2
218.1499
b3
300.1918
y3
b2-H2O
y4
446.2609
y5
559.345
y6
743.4662
y7
858.4931
y8-NH3
970.5092
y8-H2O
y8
987.5357
y9-H2O
y9
1116.578
y10
1215.647
Relative Abundance
Intensity [10e5]
m/z [amu]

**Scan number**	6668	**Raw file**	Kprop3
**Method**	FTMS; HCD	**Pepti...**	105.16

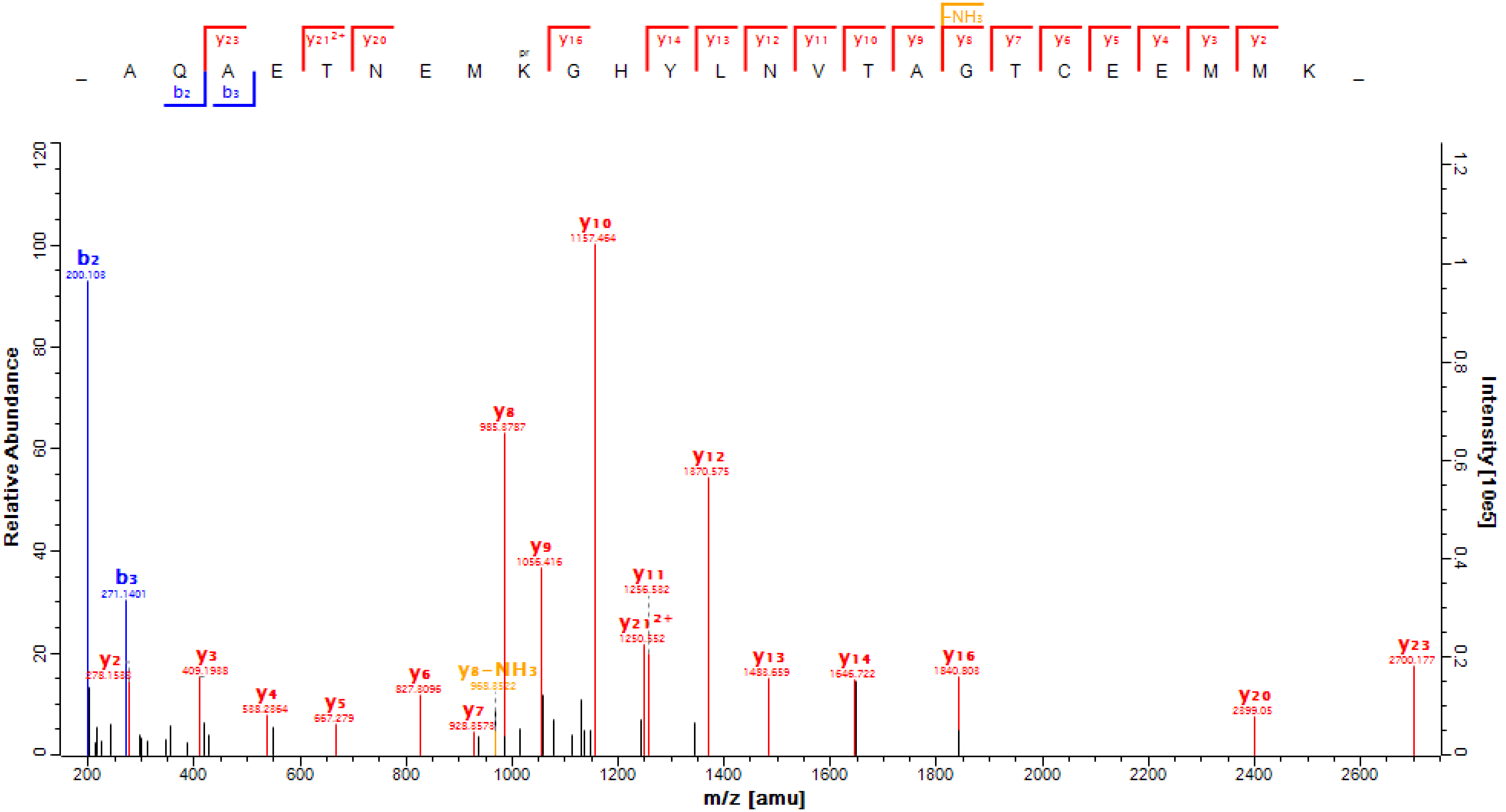

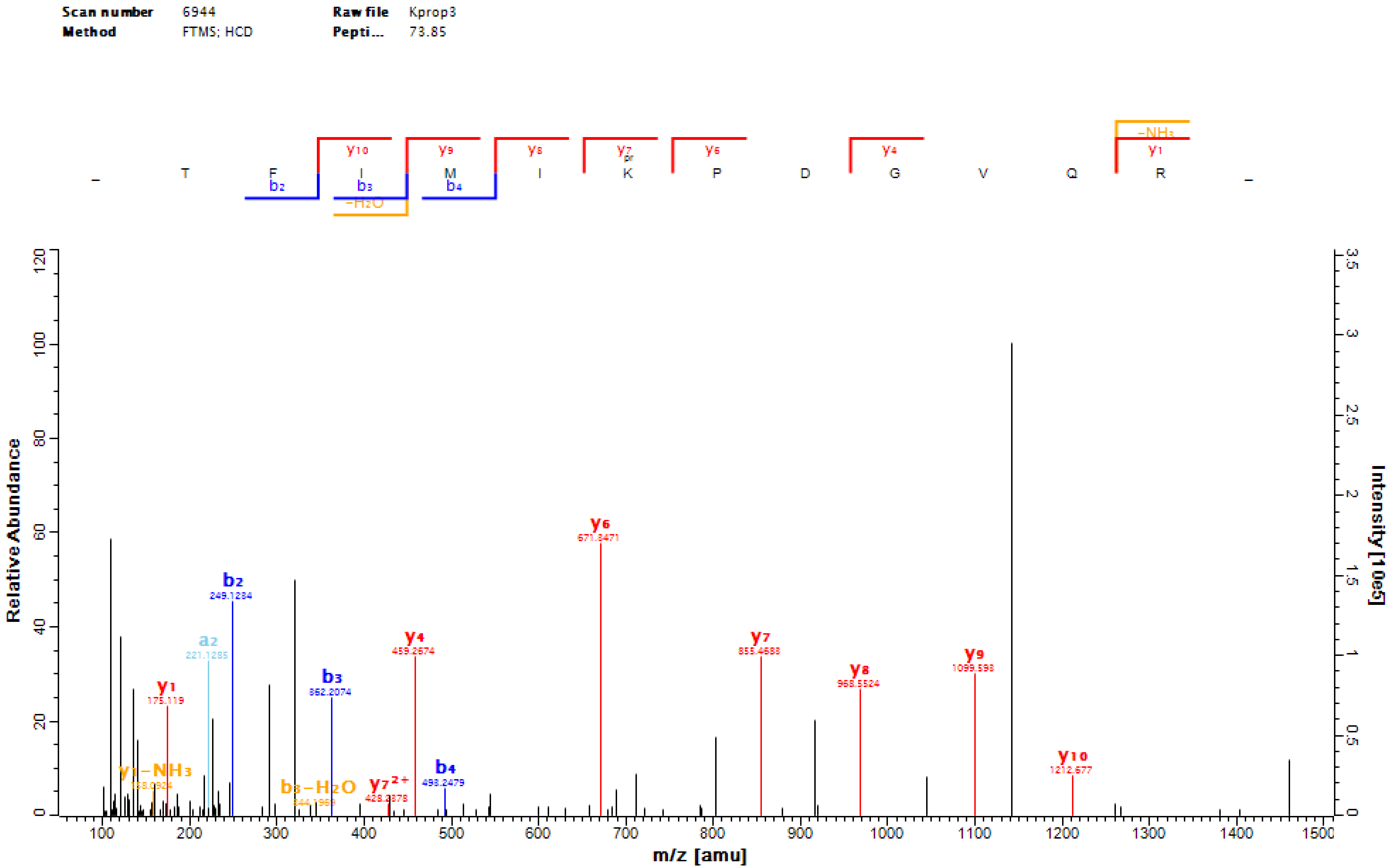
Scan number 6944
Method FTMS; HCD
Raw file Kprop3
Pepti... 73.85
_ T F I M I K P D G V Q R _
y10 y9 y8 y7pr y6 y4 y1
-NH3
b2 b3 b4
-H2O
Relative Abundance
Intensity [10e5]
m/z [amu]
y1 175.119
y1-NH3 158.0924
a2 221.1285
b2 249.1234
b3 362.2074
b3-H2O 344.1969
y7 2+ 428.2378
y4 459.2674
b4 493.2479
y6 671.3471
y7 855.4683
y8 968.5524
y9 1099.593
y10 1212.677

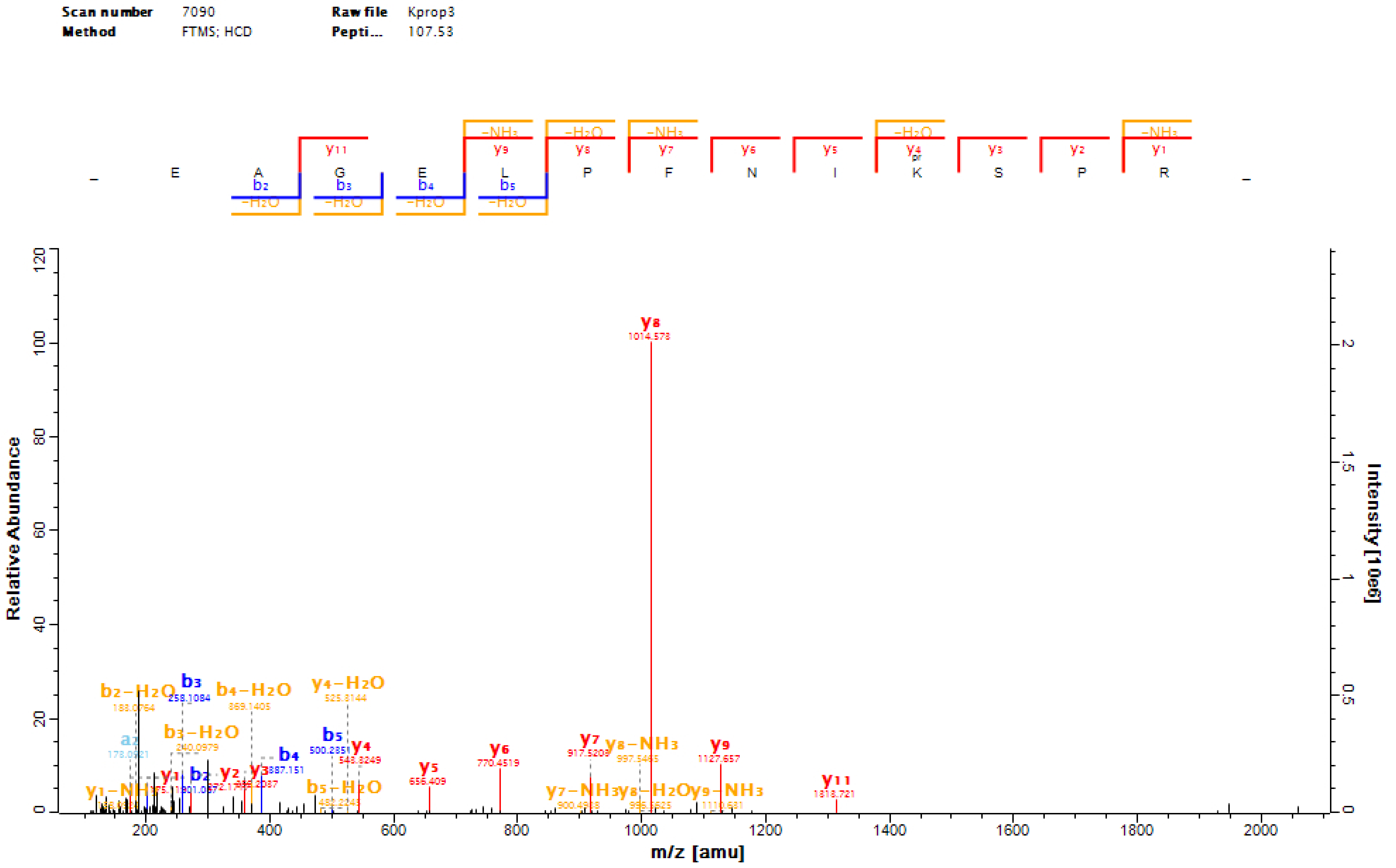

Scan number 7090
Raw file Kprop3
Method FTMS; HCD
Pepti... 107.53
_ E A G E L P F N I K S P R _
y11 y9 y8 y7 y6 y5 y4 y3 y2 y1
b2 b3 b4 b5
-H2O
-NH3
y8 1014.578
b2-H2O 183.0764
b3 258.1084
b4-H2O 369.1405
y4-H2O 525.3144
a2 173.0921
b3-H2O 240.0979
b5 500.2351
y4 543.3249
b4 387.151
y6 770.4519
y7 917.5209
y8-NH3 997.5465
y9 1127.657
y5 656.409
y11 1318.721
y1-NH3
y1 175.1
b2 201.087
y2 272.17
y3
b5-H2O 482.2245
y7-NH3 900.4938
y8-H2O 996.5625
y9-NH3 1110.631
Relative Abundance
Intensity [10e6]
m/z [amu]

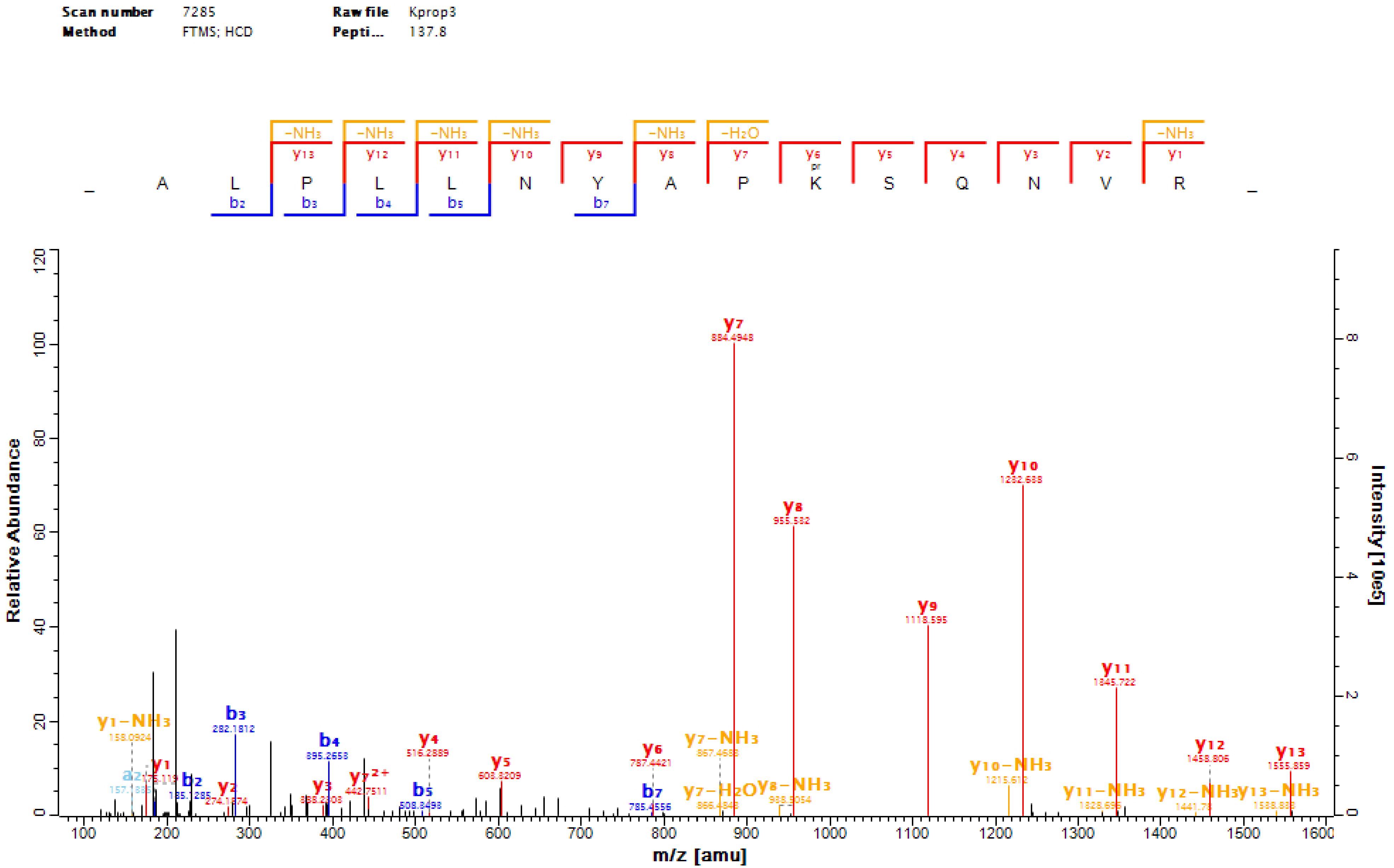
Scan number 7285
Method FTMS; HCD
Raw file Kprop3
Pepti... 137.8
_ A L P L L N Y A P K S Q N V R _
y13 y12 y11 y10 y9 y8 y7 y6 y5 y4 y3 y2 y1
-NH3 -H2O
b2 b3 b4 b5 b7
Relative Abundance
Intensity [10e5]
m/z [amu]
y7 884.4948
y10 1232.638
y8 955.532
y9 1118.595
y11 1345.722
y12 1458.806
y13 1555.859
y1-NH3 158.0924
b3 282.1812
b4 395.2653
y4 516.2889
y5 603.3209
y6 787.4421
y7-NH3 867.4683
y10-NH3 1215.612
y7-H2O
y8-NH3
y11-NH3
y12-NH3
y13-NH3
b5 508.3498
b7 785.4556
y7 2+ 442.7511
y3
y2 274.1874
b2
y1
a2

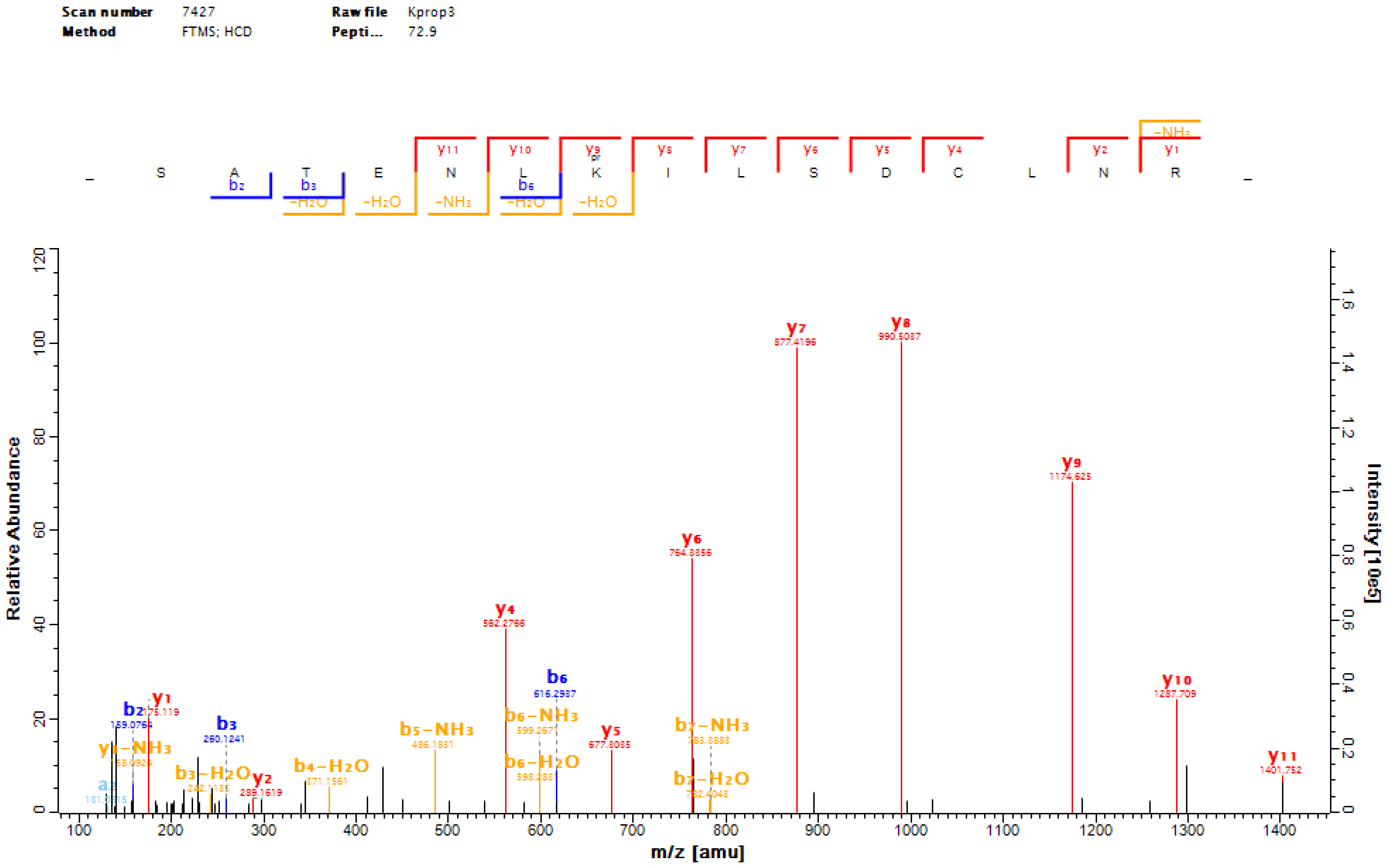

Scan number 7427
Raw file Kprop3
Method FTMS; HCD
Pepti... 72.9
_ S A T E N L K I L S D C L N R _
Relative Abundance
Intensity [10e5]
m/z [amu]
y7 877.4196
y8 990.5087
y9 1174.625
y6 764.3356
y4 562.2766
b6 616.2987
y10 1287.709
y11 1401.752
y5 677.3085
b5−NH3 486.1881
b6−NH3 599.2671
b6−H2O
b7−NH3
b7−H2O
b4−H2O 371.1561
b3−H2O 242.1135
b3 260.1241
b2 159.0764
y1 175.119
y2 289.1619
y1−NH3 158.0924

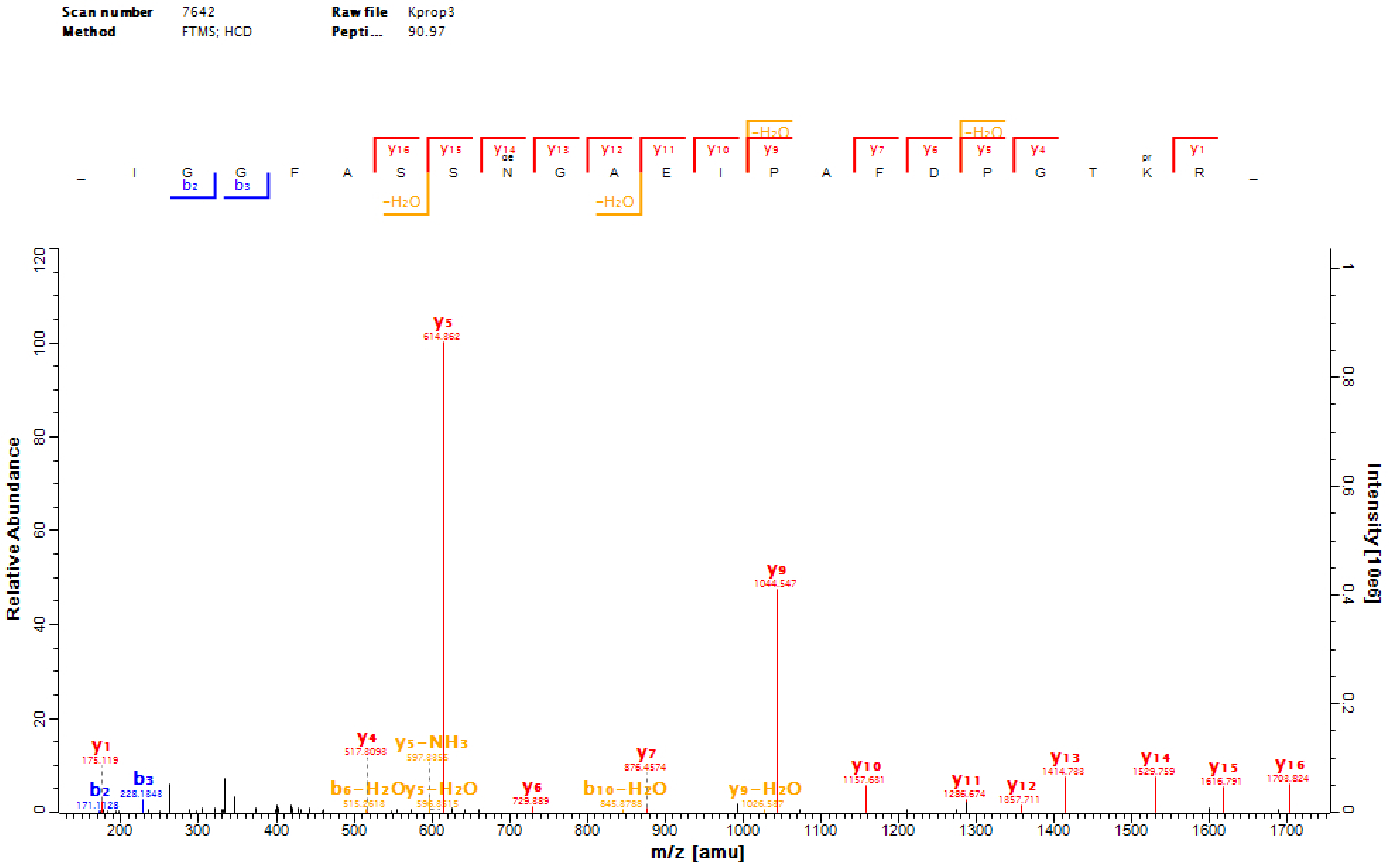
Scan number 7642
Raw file Kprop3
Method FTMS; HCD
Pepti... 90.97
_ I G G F A S S N G A E I P A F D P G T K R _
b2 b3
y16 y15 y14 y13 y12 y11 y10 y9 y7 y6 y5 y4 y1
-H2O
de
pr
y5 614.862
y9 1044.547
y1 175.119
b2 171.1128
b3 228.1348
y4 517.3098
b6-H2O 515.2618
y5-NH3 597.8356
y5-H2O
y6 729.389
y7 876.4574
b10-H2O 845.3788
y9-H2O 1026.537
y10 1157.631
y11 1286.674
y12 1357.711
y13 1414.733
y14 1529.759
y15 1616.791
y16 1703.824
Relative Abundance
Intensity [10e6]
m/z [amu]

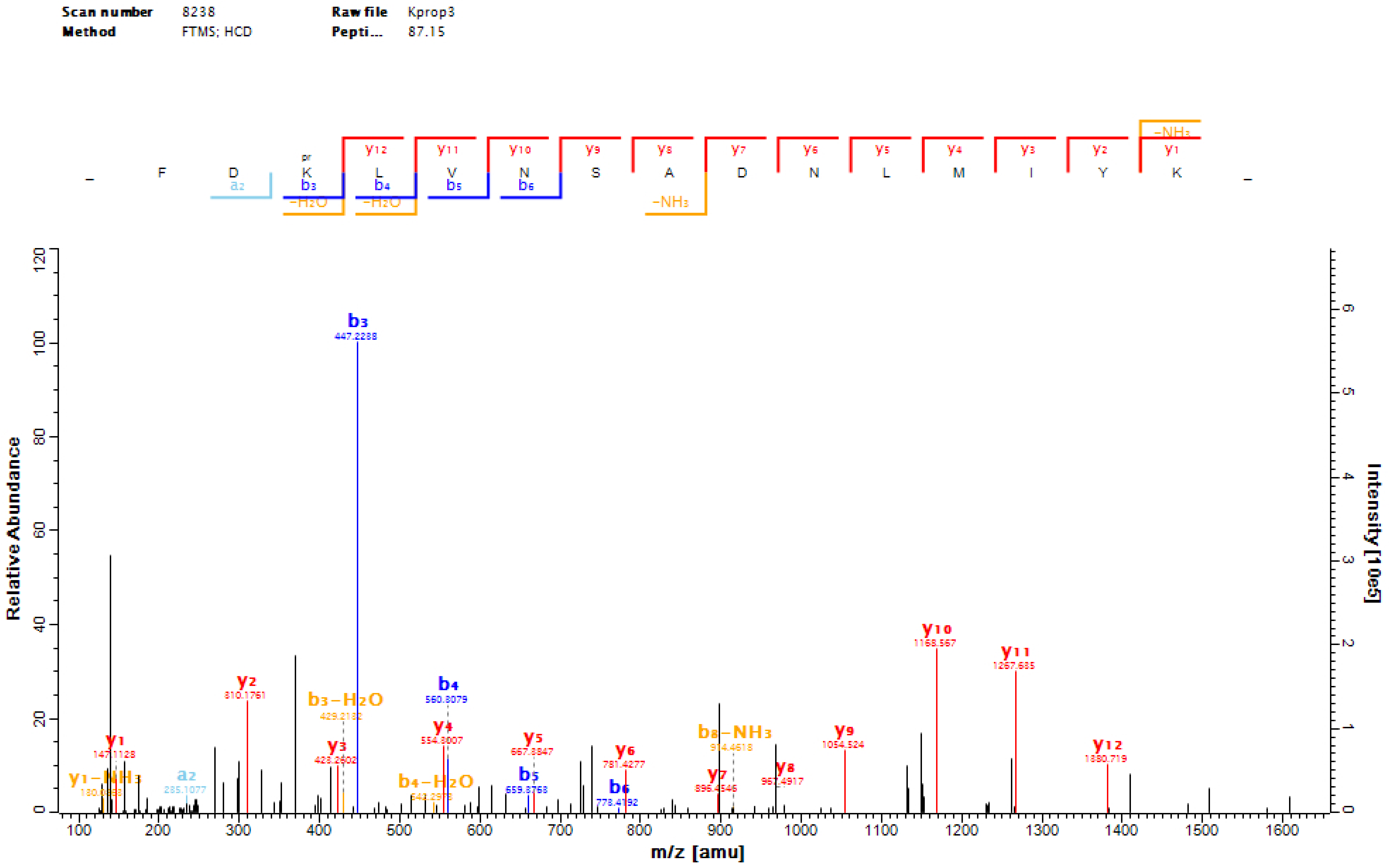

Scan number 8238
Method FTMS; HCD
Raw file Kprop3
Pepti... 87.15
F D K L V N S A D N L M I Y K
pr
y12 y11 y10 y9 y8 y7 y6 y5 y4 y3 y2 y1
-NH3
a2 b3 b4 b5 b6
-H2O -H2O -NH3
b3 447.2288
y10 1168.567
y11 1267.635
y2 310.1761
b3-H2O 429.2182
b4 560.3079
y4 554.3007
y1 147.1128
y3 423.2602
y5 667.3847
y6 781.4277
b8-NH3 914.4618
y9 1054.524
y12 1380.719
a2 235.1077
b4-H2O
b5 659.3768
b6 773.4192
y7 896.4546
y8 967.4917
Relative Abundance
Intensity [10e5]
m/z [amu]

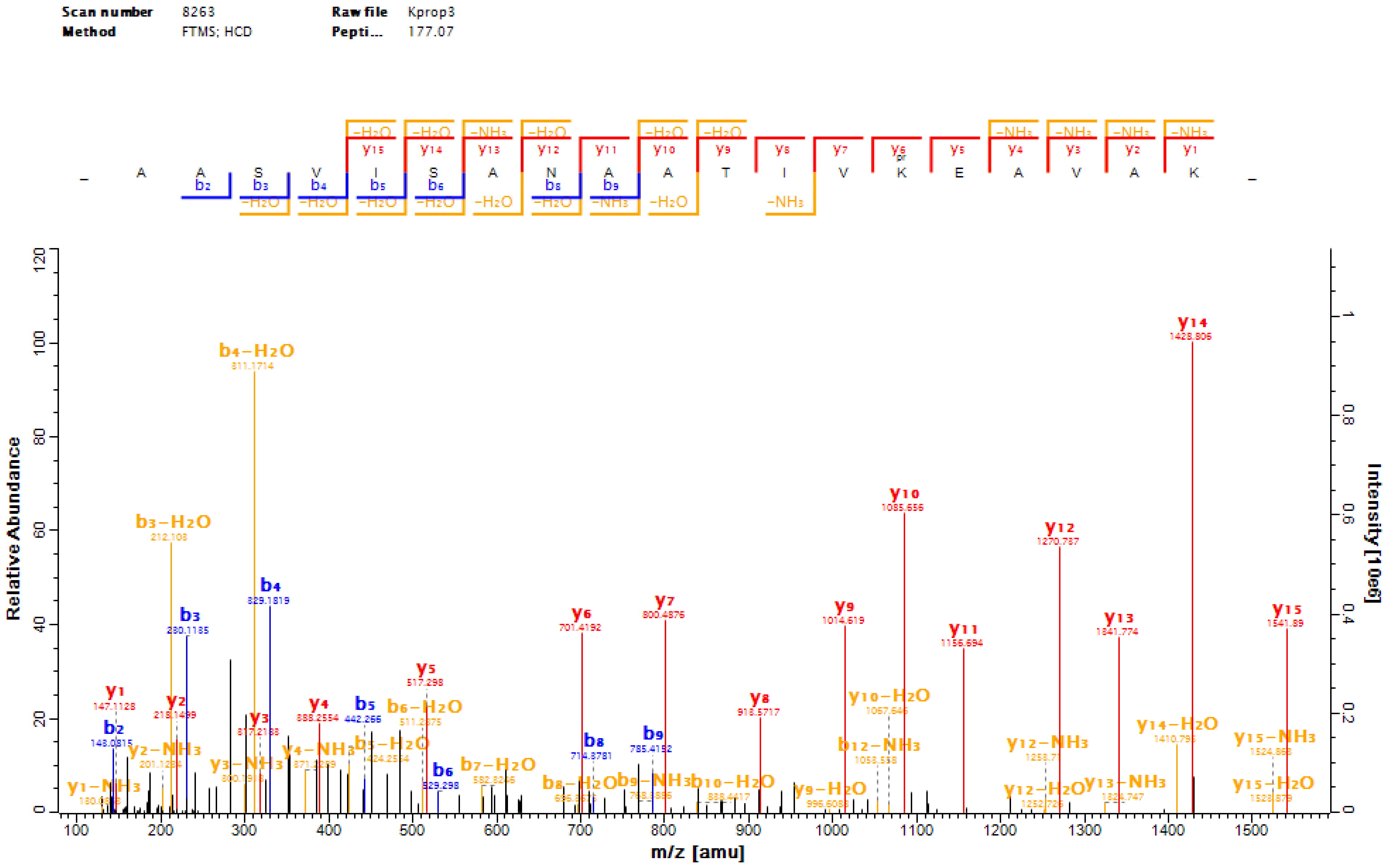

Scan number 8263
Raw file Kprop3
Method FTMS; HCD
Pepti... 177.07
A A S V I S A N A A T I V K E A V A K
Relative Abundance
Intensity [10e6]
m/z [amu]
y14 1428.806
b4−H2O 311.1714
y10 1085.656
b3−H2O 212.108
y12 1270.787
b4 329.1819
b3 230.1185
y6 701.4192
y7 800.4876
y9 1014.619
y11 1156.694
y13 1341.774
y15 1541.89
y5 517.298
y1 147.1128
y2 218.1499
b2 143.0815
y3 317.2188
y4 388.2554
b5 442.266
b6−H2O 511.2875
b5−H2O 424.2554
b6 529.298
b7−H2O 582.3246
y8 913.5717
b8 714.3781
b9 785.4152
y10−H2O 1067.646
b12−NH3 1053.558
y14−H2O 1410.795
y15−NH3 1524.863
y15−H2O 1523.879
y12−NH3 1253.71
y12−H2O 1252.726
y13−NH3 1324.747
y9−H2O 996.6088
b10−H2O 838.4417
b9−NH3
b8−H2O
y1−NH3
y2−NH3 201.1234
y3−NH3 300.1918
y4−NH3 371.2289

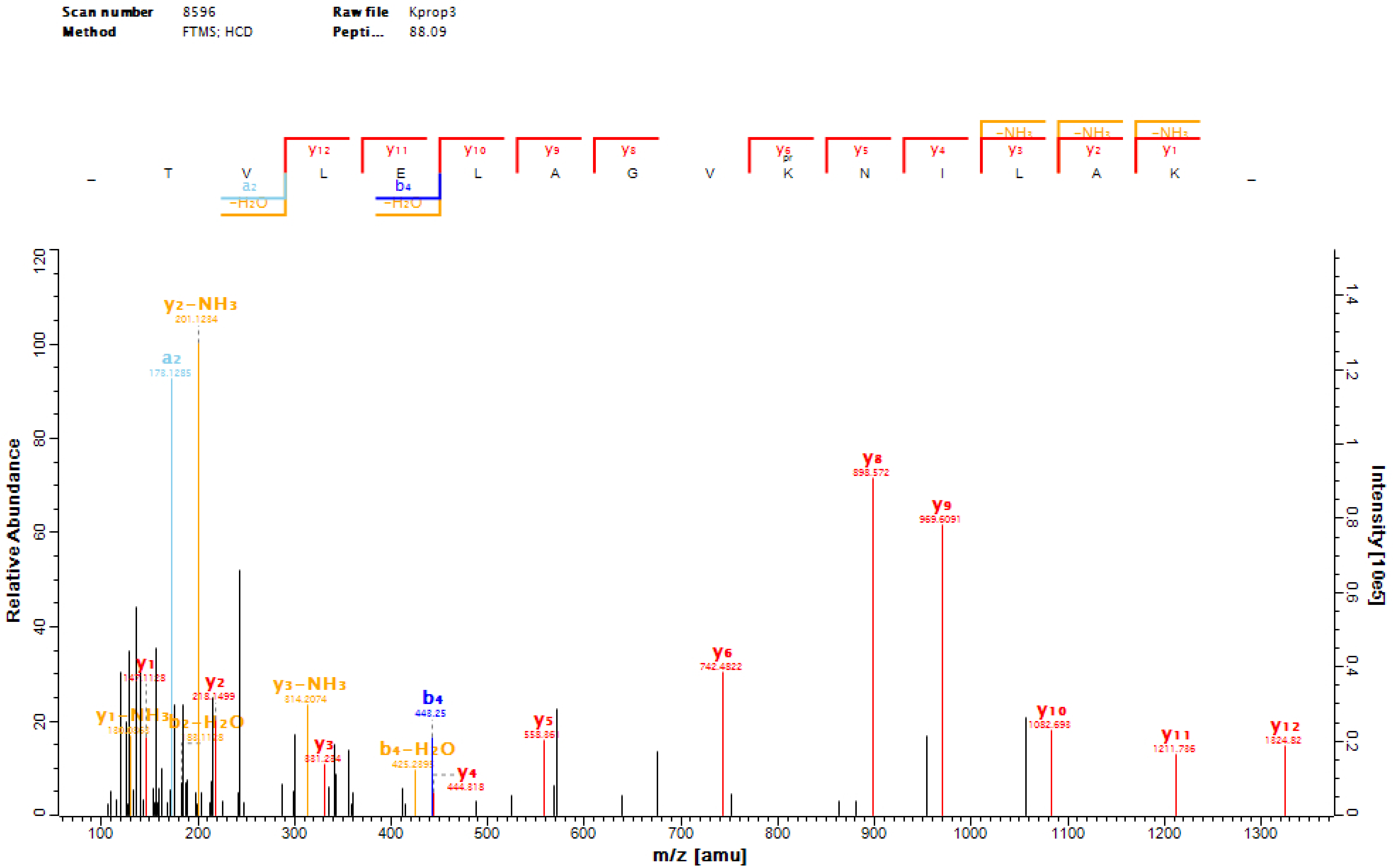

Scan number 8596
Method FTMS; HCD
Raw file Kprop3
Pepti... 88.09
_ T V L E L A G V K N I L A K _
y12 y11 y10 y9 y8 y6 y5 y4 y3 y2 y1
a2 b4 -H2O -NH3
Relative Abundance
Intensity [10e5]
m/z [amu]
y2-NH3 201.1284
a2 173.1285
y8 898.572
y9 969.6091
y6 742.4822
y1 147.1128
y2 218.1499
y3-NH3 314.2074
b4 443.25
y1-NH3
b2-H2O
y5 558.361
y10 1082.693
y11 1211.736
y12 1324.82
y3 331.234
b4-H2O 425.2395
y4 444.318

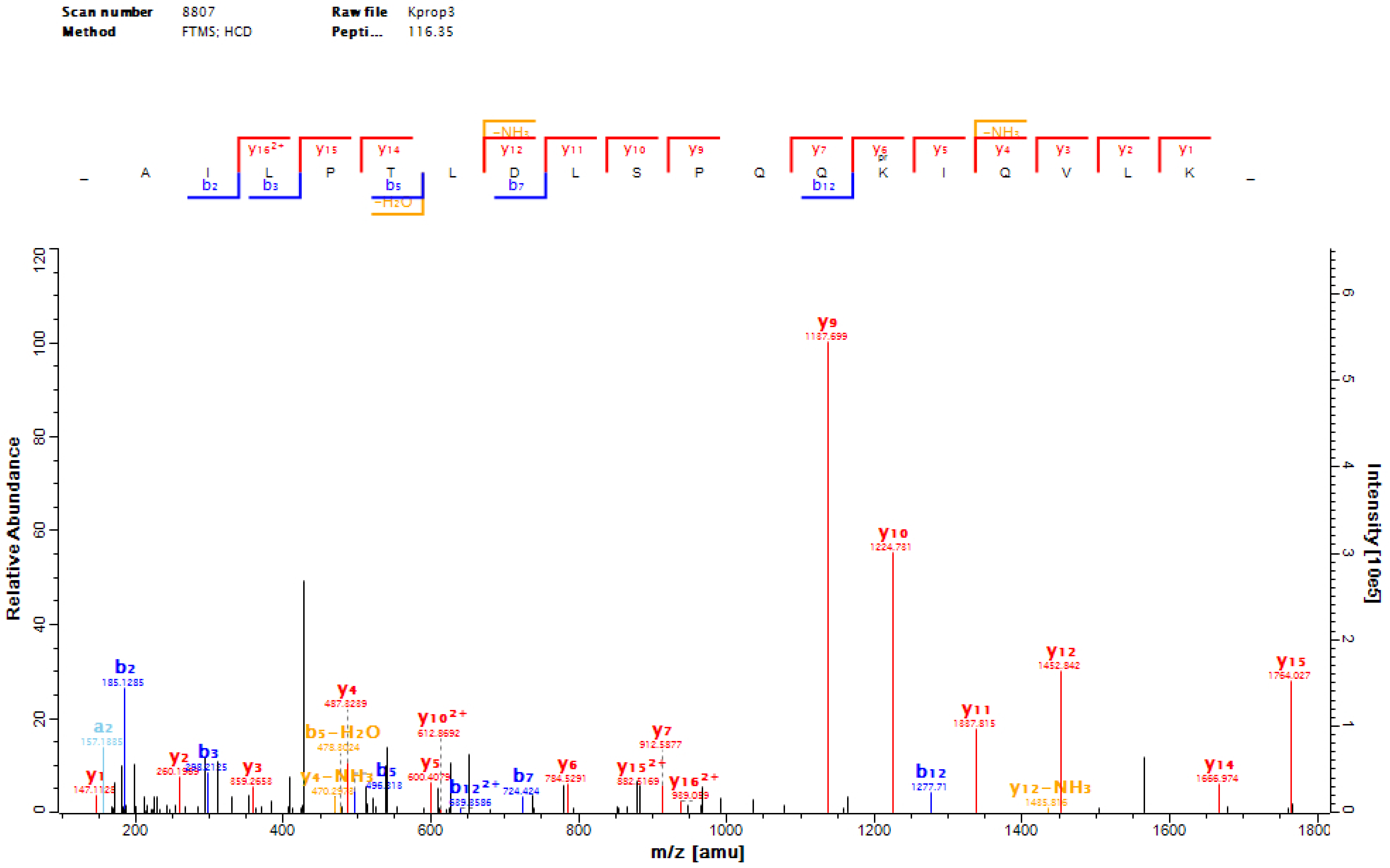

Scan number 8807
Raw file Kprop3
Method FTMS; HCD
Pepti... 116.35
_ A I L P T L D L S P Q Q K I Q V L K _
Relative Abundance
Intensity [10e5]
m/z [amu]

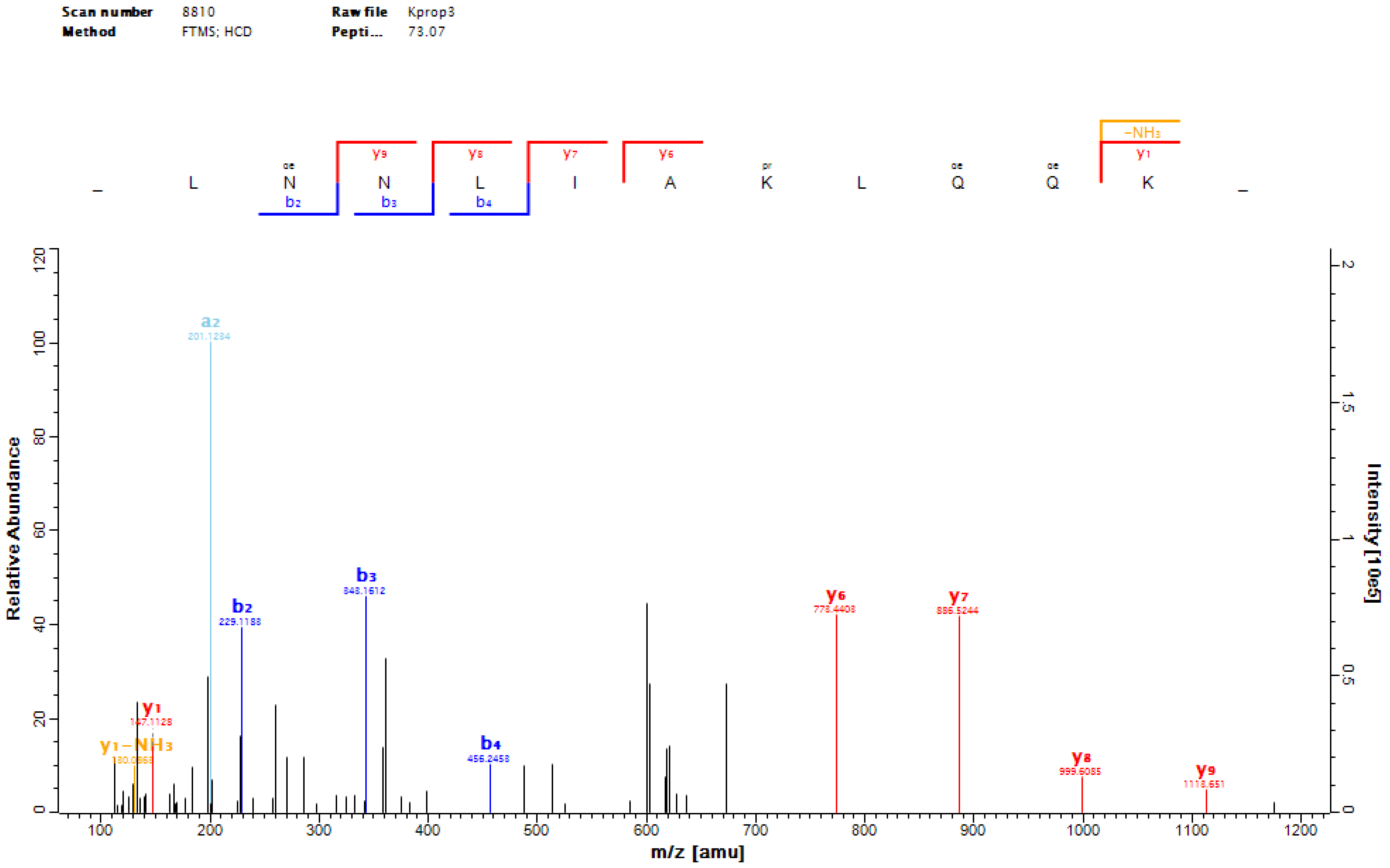

Scan number 8810
Method FTMS; HCD
Raw file Kprop3
Pepti... 73.07
_ L N N L I A K L Q Q K _
oe
pr
-NH3
y9 y8 y7 y6 y1
b2 b3 b4
a2
y1
y1-NH3
b2
b3
b4
y6
y7
y8
y9
Relative Abundance
Intensity [10e5]
m/z [amu]

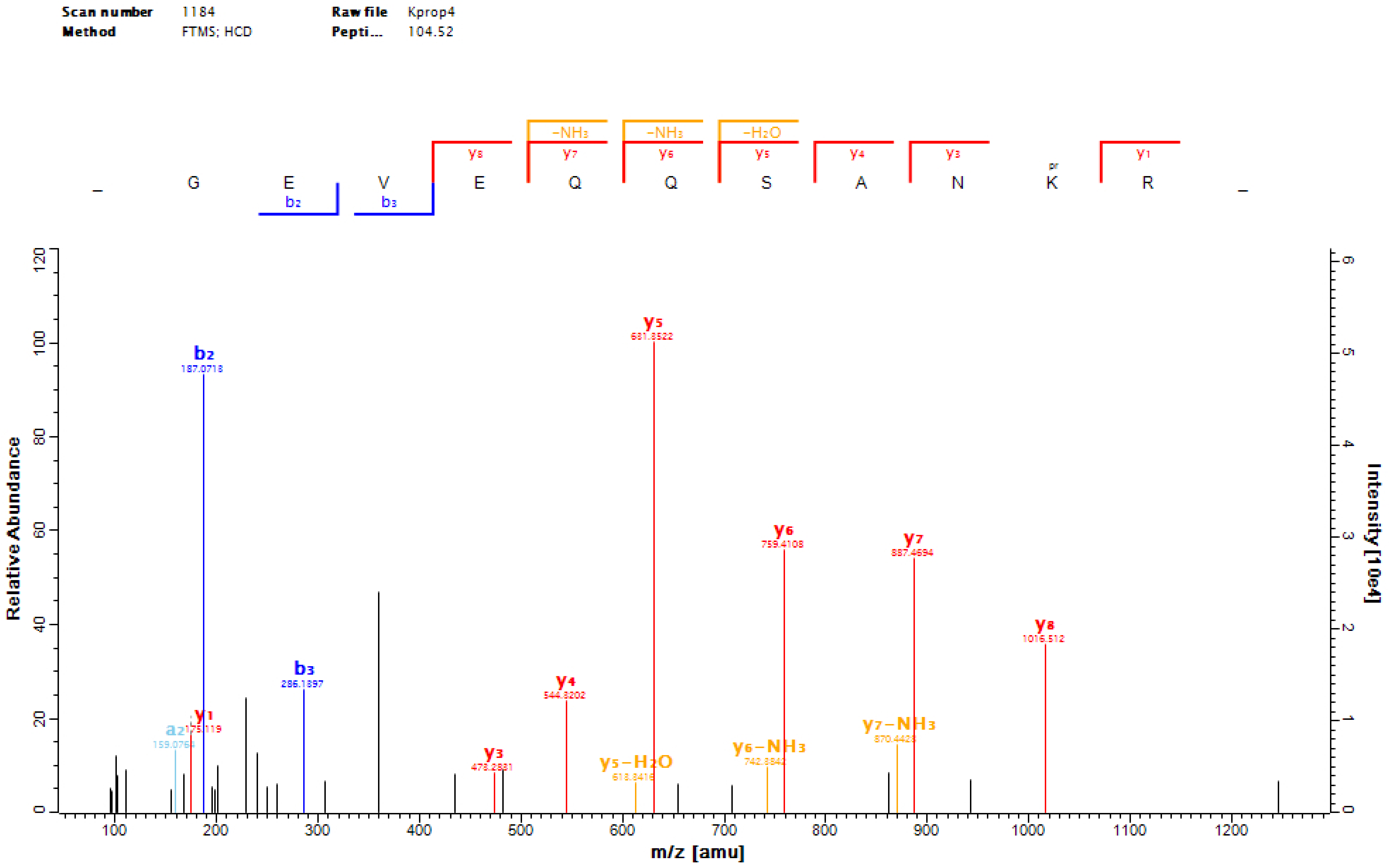

Scan number 1184
Raw file Kprop4
Method FTMS; HCD
Pepti... 104.52
_ G E V E Q Q S A N K R _
pr
b2
b3
y8
y7
y6
y5
y4
y3
y1
-NH3
-NH3
-H2O
Relative Abundance
Intensity [10e4]
m/z [amu]
b2 187.0718
b3 286.1897
a2 159.0764
y1 175.119
y3 473.2831
y4 544.3202
y5 631.3522
y5-H2O 613.3416
y6 759.4108
y6-NH3 742.3842
y7 887.4694
y7-NH3 870.4428
y8 1016.512

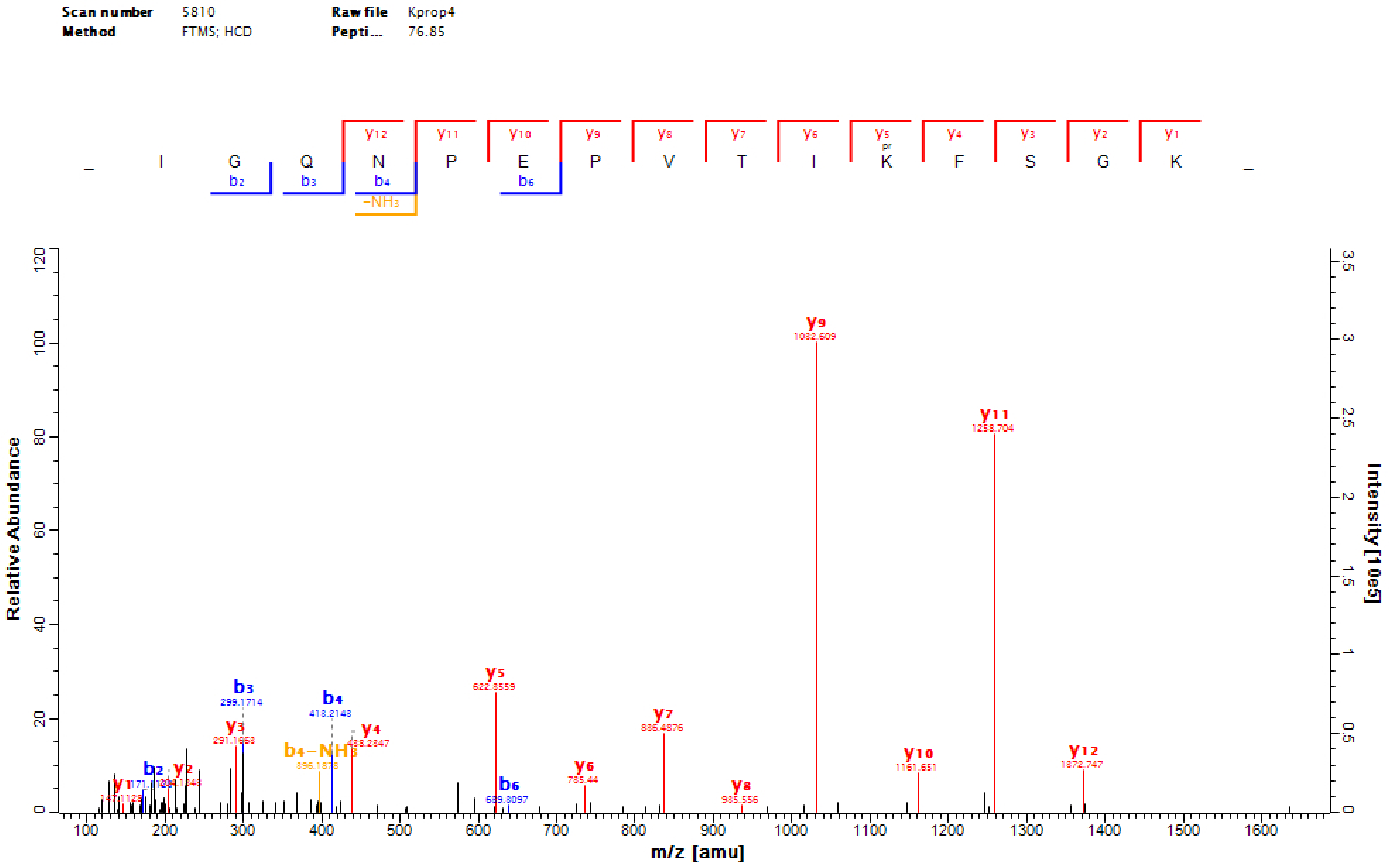

Scan number 5810
Method FTMS; HCD
Raw file Kprop4
Pepti... 76.85
_ I G Q N P E P V T I K F S G K _
y12 y11 y10 y9 y8 y7 y6 y5 y4 y3 y2 y1
b2 b3 b4 b6
-NH3
y9 1032.609
y11 1258.704
y5 622.3559
b3 299.1714
b4 418.2148
y3 291.1668
y4 438.2347
b4-NH3
y7 836.4876
y10 1161.651
y12 1372.747
y6 735.44
y8 935.556
b6
y1
b2
y2
Relative Abundance
Intensity [10e5]
m/z [amu]

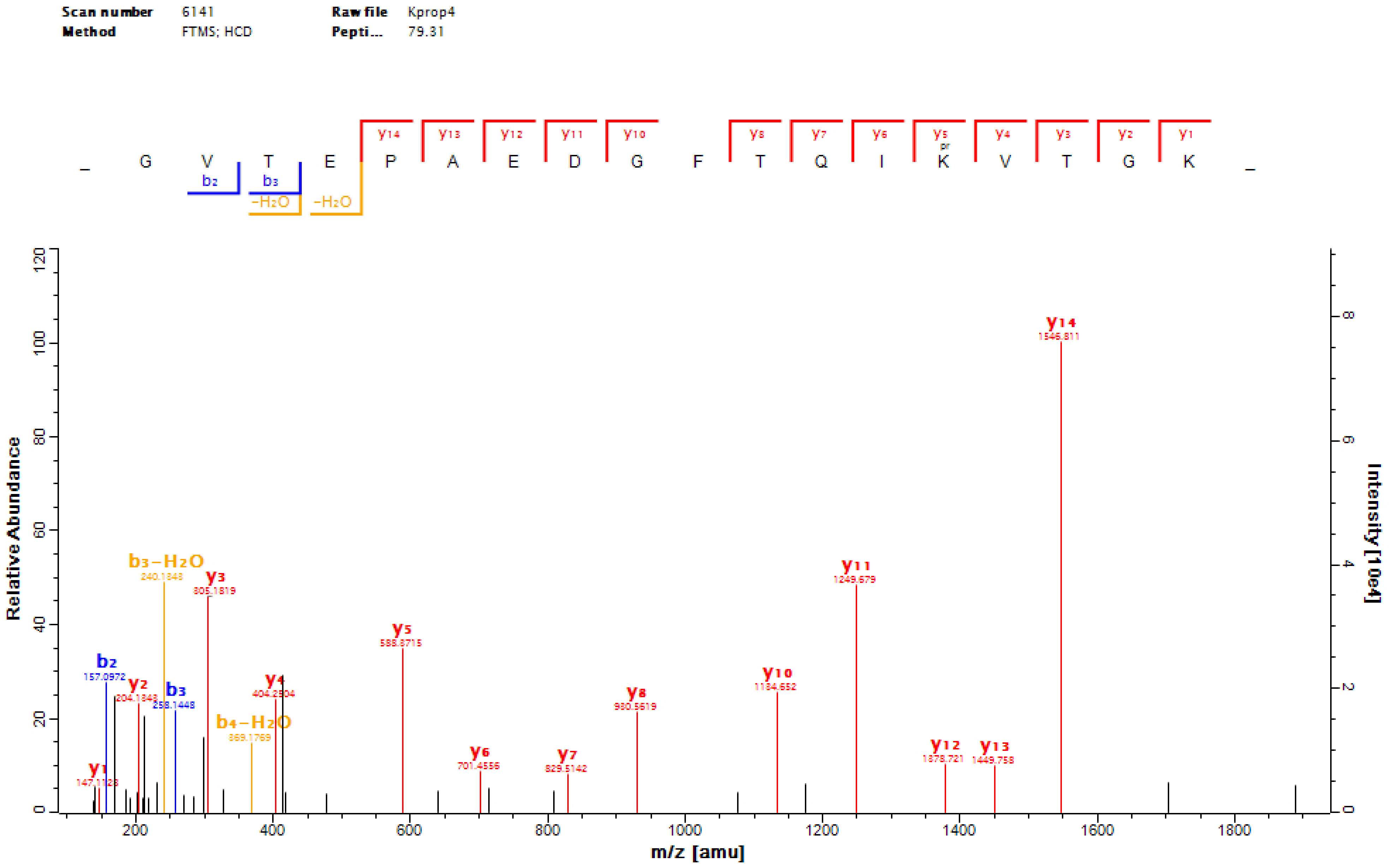

Scan number 6141
Raw file Kprop4
Method FTMS; HCD
Pepti... 79.31
_ G V T E P A E D G F T Q I K V T G K _
b2 b3
-H2O -H2O
y14 y13 y12 y11 y10 y8 y7 y6 y5 y4 y3 y2 y1
pr
Relative Abundance
Intensity [10e4]
m/z [amu]
y14 1546.811
y11 1249.679
y10 1134.652
y8 930.5619
y5 588.8715
y3 305.1819
b3-H2O 240.1343
b2 157.0972
y2 204.1343
b3 258.1448
y4 404.2504
b4-H2O 369.1769
y1 147.1123
y6 701.4556
y7 829.5142
y12 1378.721
y13 1449.758

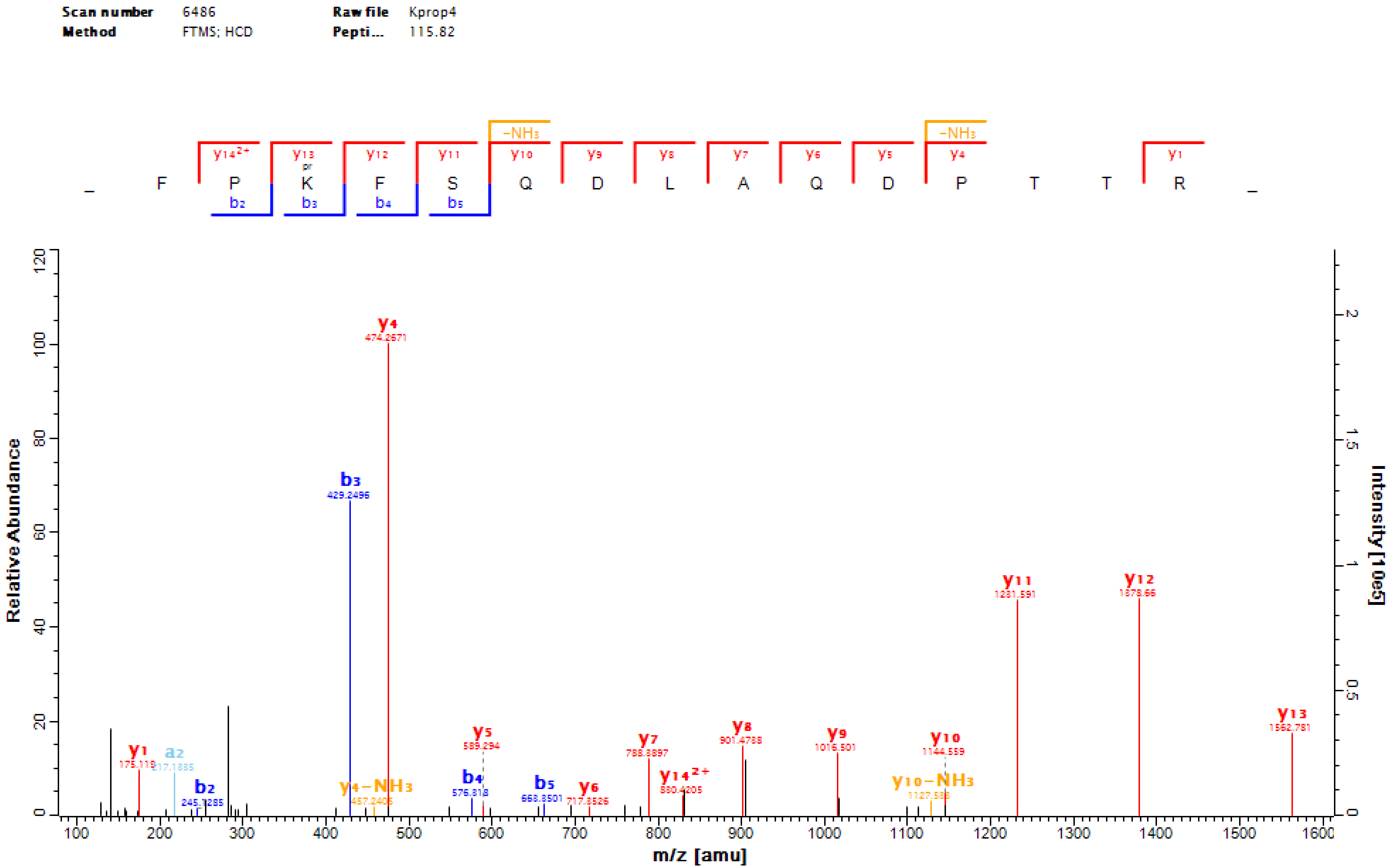

Scan number 6486
Method FTMS; HCD
Raw file Kprop4
Pepti... 115.82
_ F P K F S Q D L A Q D P T T R _
y14 2+ y13 y12 y11 y10 y9 y8 y7 y6 y5 y4 y1
-NH3 -NH3
b2 b3 b4 b5
y4 474.2671
b3 429.2496
y11 1231.591
y12 1378.66
y13 1562.781
y1 175.119
a2 217.1385
b2 245.1285
y4-NH3 457.2406
y5 589.294
b4 576.318
b5 663.3501
y6 717.3526
y7 788.3897
y14 2+ 830.4205
y8 901.4788
y9 1016.501
y10 1144.559
y10-NH3 1127.533
Relative Abundance
Intensity [10e5]
m/z [amu]

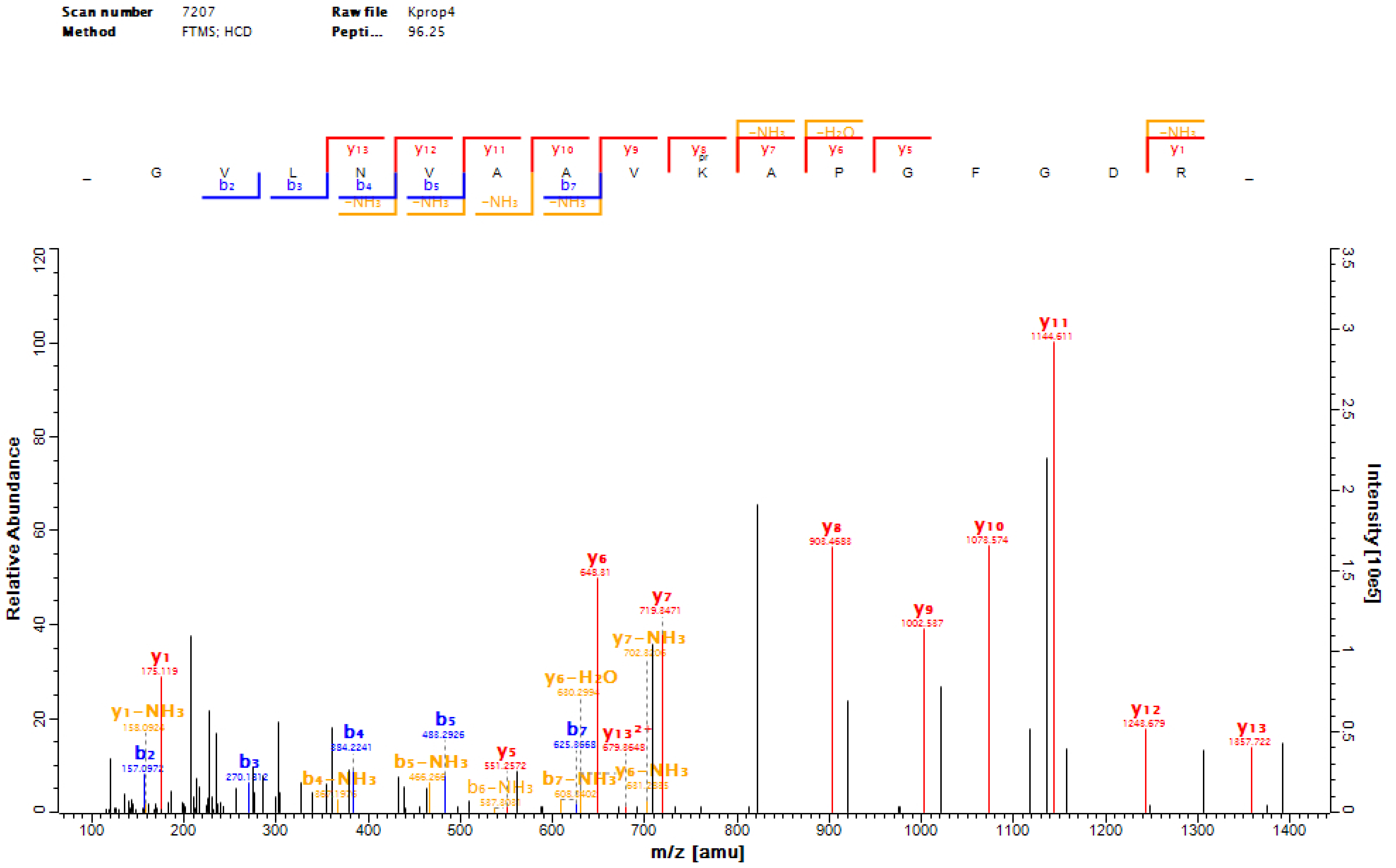
Scan number 7207
Raw file Kprop4
Method FTMS; HCD
Pepti... 96.25
_ G V L N V A A V K A P G F G D R _
b2 b3 b4 b5 b7
-NH3 -NH3 -NH3 -NH3
y13 y12 y11 y10 y9 y8 y7 y6 y5 y1
-NH3 -H2O -NH3
Relative Abundance
Intensity [10e5]
m/z [amu]
y1 175.119
y1-NH3 158.0924
b2 157.0972
b3 270.1812
b4 384.2241
b4-NH3 367.1976
b5 483.2926
b5-NH3 466.266
y5 551.2572
b6-NH3 537.3031
b7 625.3668
b7-NH3 608.3402
y6 648.31
y6-H2O 630.2994
y13 2+ 679.3648
y6-NH3 631.2835
y7 719.3471
y7-NH3 702.3206
y8 903.4683
y9 1002.537
y10 1073.574
y11 1144.611
y12 1243.679
y13 1357.722

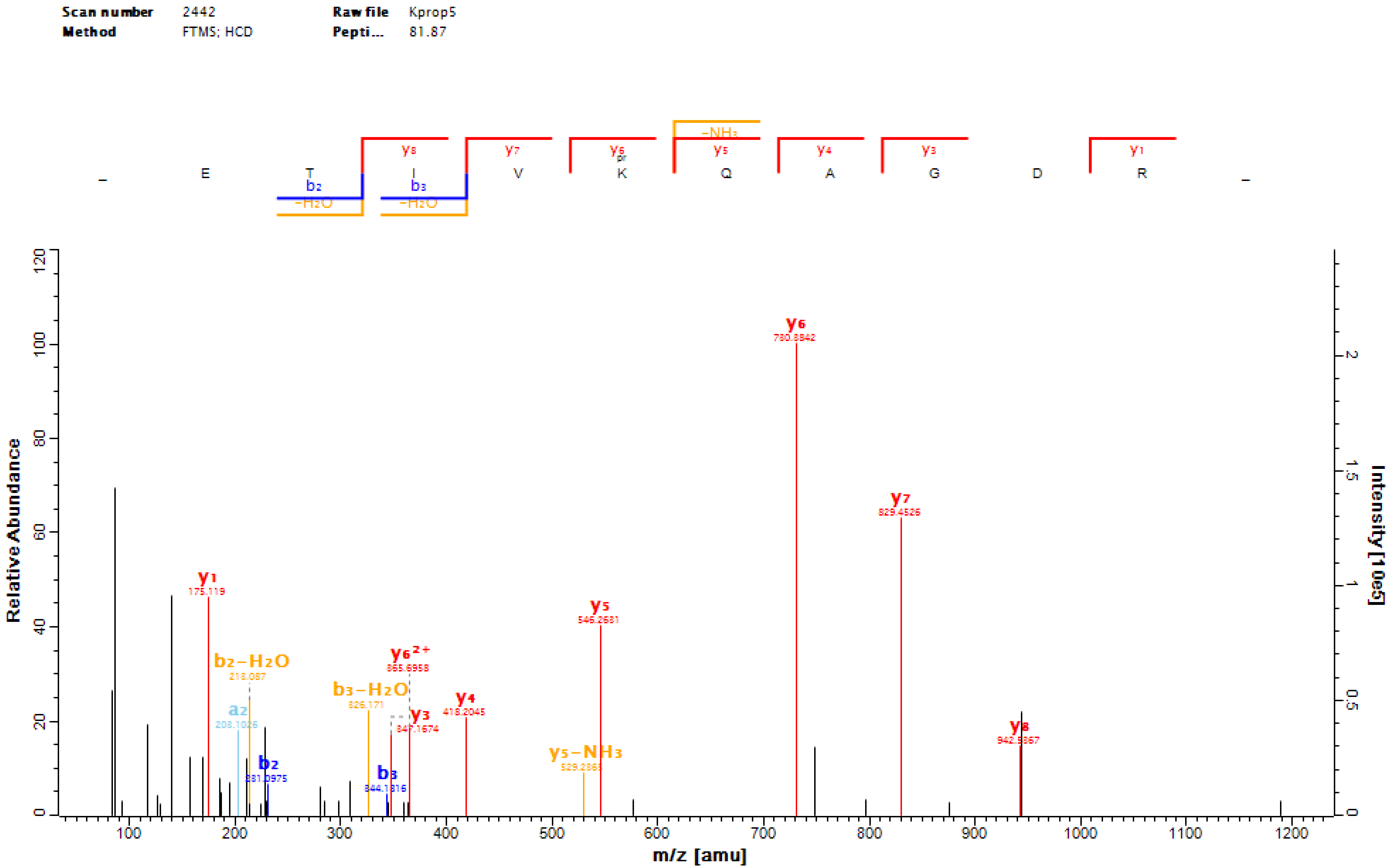

Scan number 2442
Raw file Kprop5
Method FTMS; HCD
Pepti... 81.87
E T I V K Q A G D R
y1 175.119
b2-H2O 213.087
a2 203.1026
b2 231.0975
b3-H2O 326.171
b3 344.1316
y6 2+ 365.6958
y3 347.1674
y4 418.2045
y5-NH3 529.2365
y5 546.2631
y6 730.3842
y7 829.4526
y8 942.5367
Relative Abundance
Intensity [10e5]
m/z [amu]

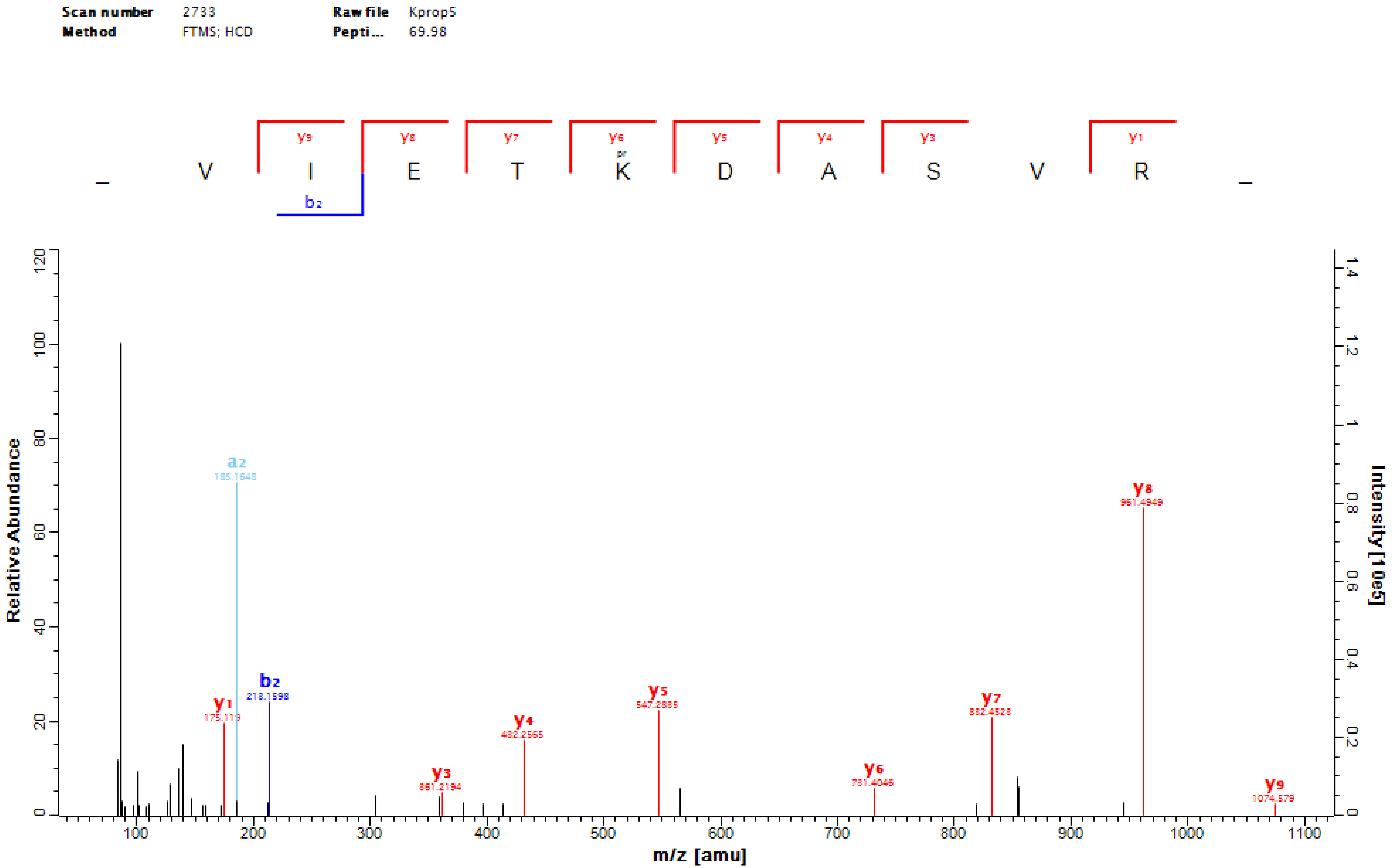

Scan number 2733
Method FTMS; HCD
Raw file Kprop5
Pepti... 69.98
_ V I E T K D A S V R _
y9 y8 y7 y6 y5 y4 y3 y1
pr
b2
a2
185.1648
y8
961.4949
b2
218.1598
y1
175.119
y5
547.2835
y7
832.4523
y4
432.2565
y3
361.2194
y6
731.4046
y9
1074.579
Relative Abundance
Intensity [10e5]
m/z [amu]

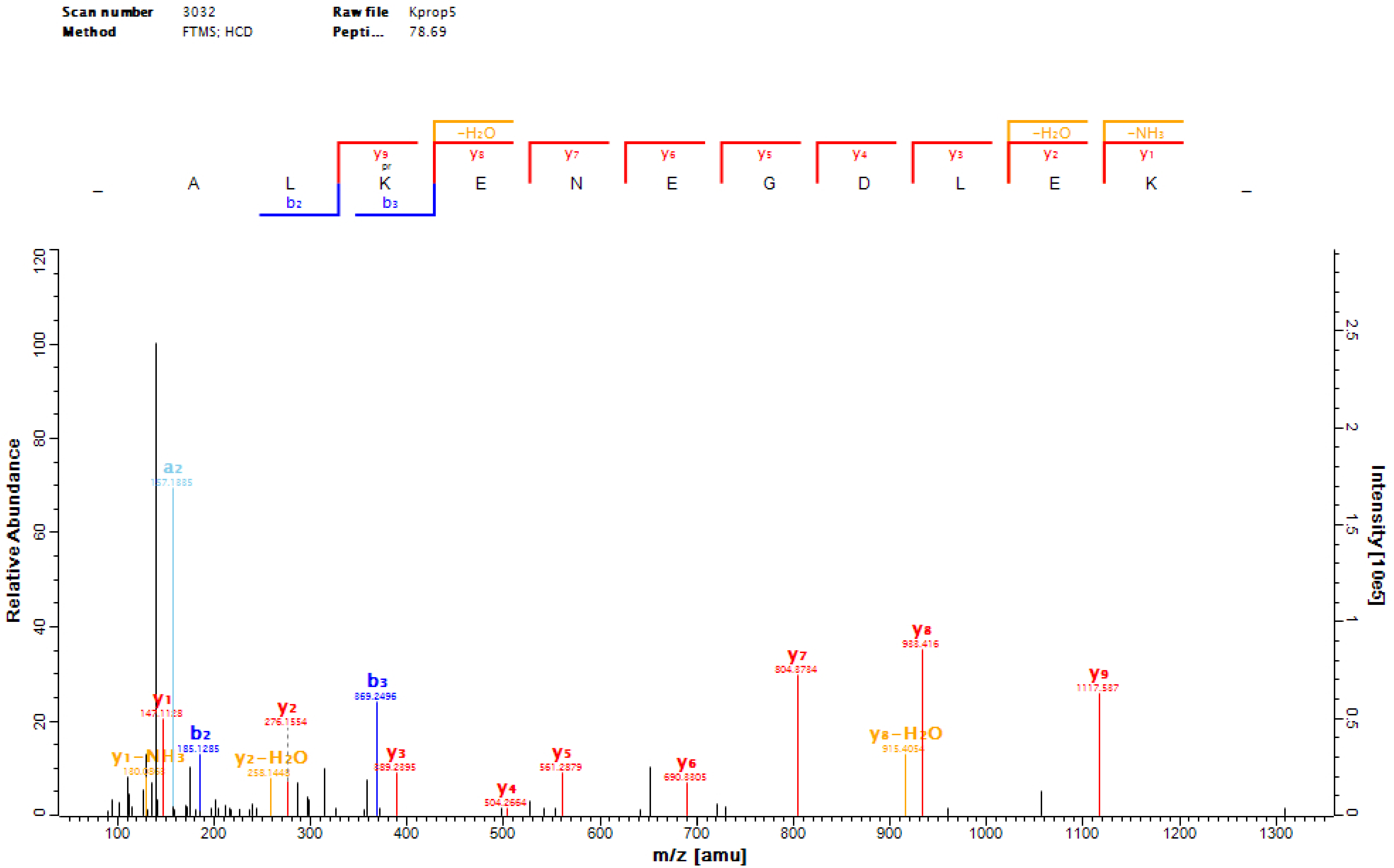

Scan number 3032
Method FTMS; HCD
Raw file Kprop5
Pepti... 78.69
_ A L K E N E G D L E K _
b2 b3
y9 y8 y7 y6 y5 y4 y3 y2 y1
-H2O -H2O -NH3
a2
y1
y1-NH3
b2 185.1285
y2 276.1554
y2-H2O 258.1448
b3
y3
y4 504.2664
y5 561.2879
y6
y7 804.3784
y8 933.416
y8-H2O 915.4054
y9 1117.537
Relative Abundance
Intensity [10e5]
m/z [amu]

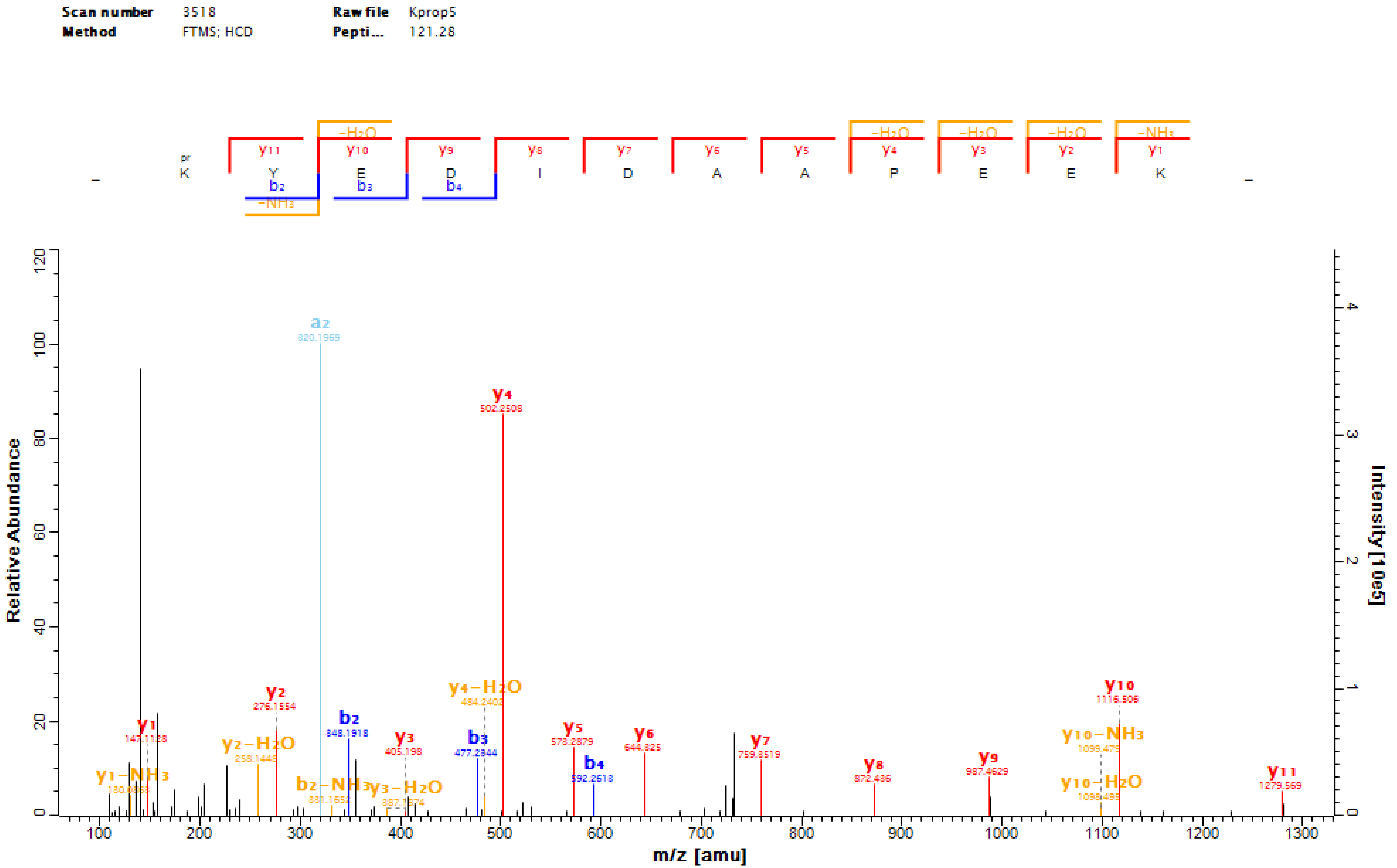

Scan number 3518
Method FTMS; HCD
Raw file Kprop5
Pepti... 121.28
K Y E D I D A A P E E K
y11 y10 y9 y8 y7 y6 y5 y4 y3 y2 y1
b2 b3 b4
-H2O
-NH3
Relative Abundance
Intensity [10e5]
m/z [amu]
a2
320.1969
y4
502.2508
y1
147.1128
y2
276.1554
b2
348.1918
y3
405.198
b3
477.2344
y4-H2O
484.2402
y5
573.2879
b4
592.2618
y6
644.325
y7
759.3519
y8
872.436
y9
987.4629
y10
1116.506
y10-NH3
y10-H2O
y11
1279.569
y1-NH3
y2-H2O
258.1448
b2-NH3
y3-H2O

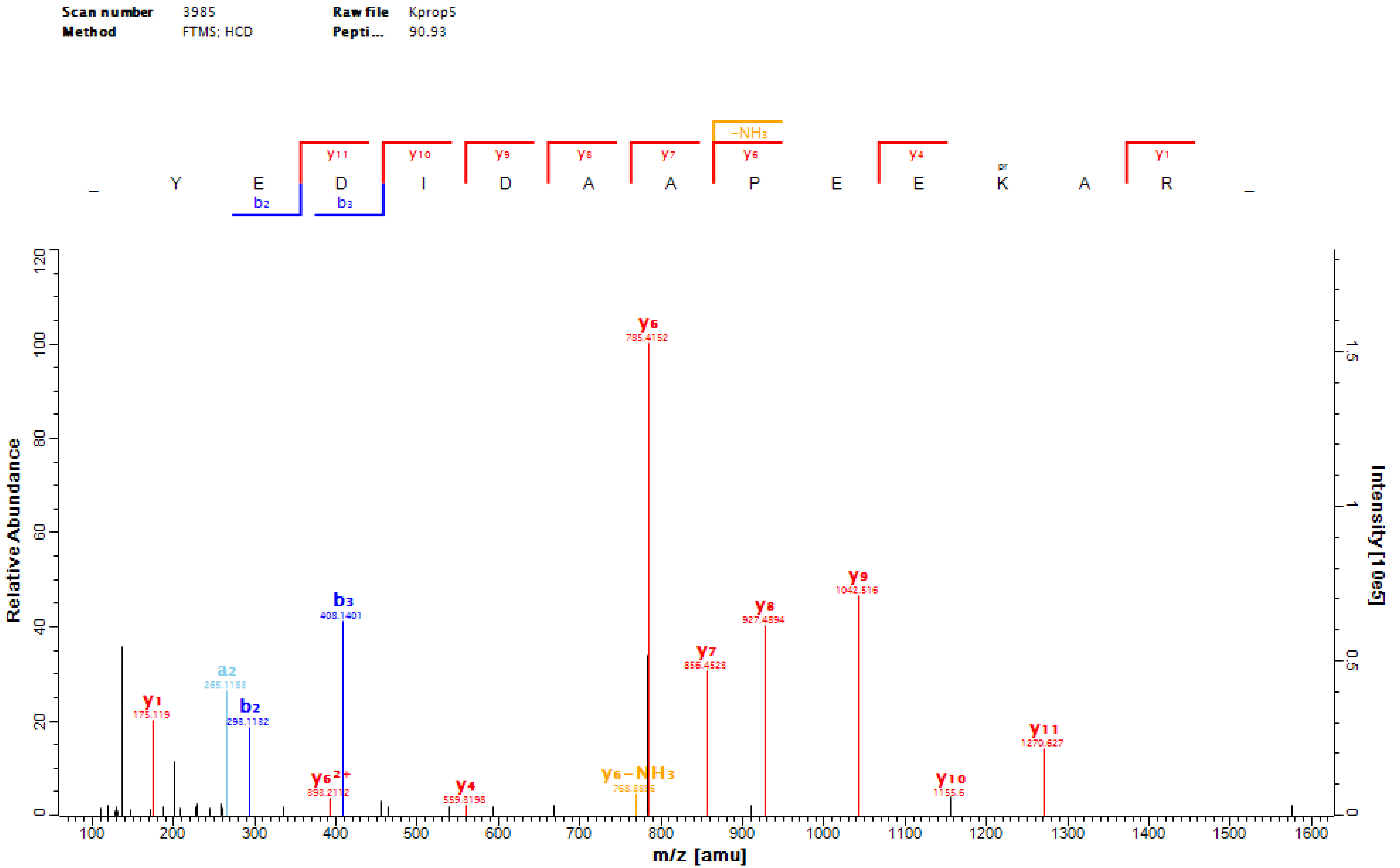

Scan number 3985
Raw file Kprop5
Method FTMS; HCD
Pepti... 90.93
_ Y E D I D A A P E E K A R _
pr
y11 y10 y9 y8 y7 y6 y4 y1
-NH3
b2 b3
Relative Abundance
Intensity [10e5]
m/z [amu]
y6 785.4152
b3 408.1401
y9 1042.516
y8 927.4894
y7 856.4523
a2 265.1188
y1 175.119
b2 293.1182
y11 1270.627
y10 1155.6
y6 2+ 393.2112
y4 559.3198

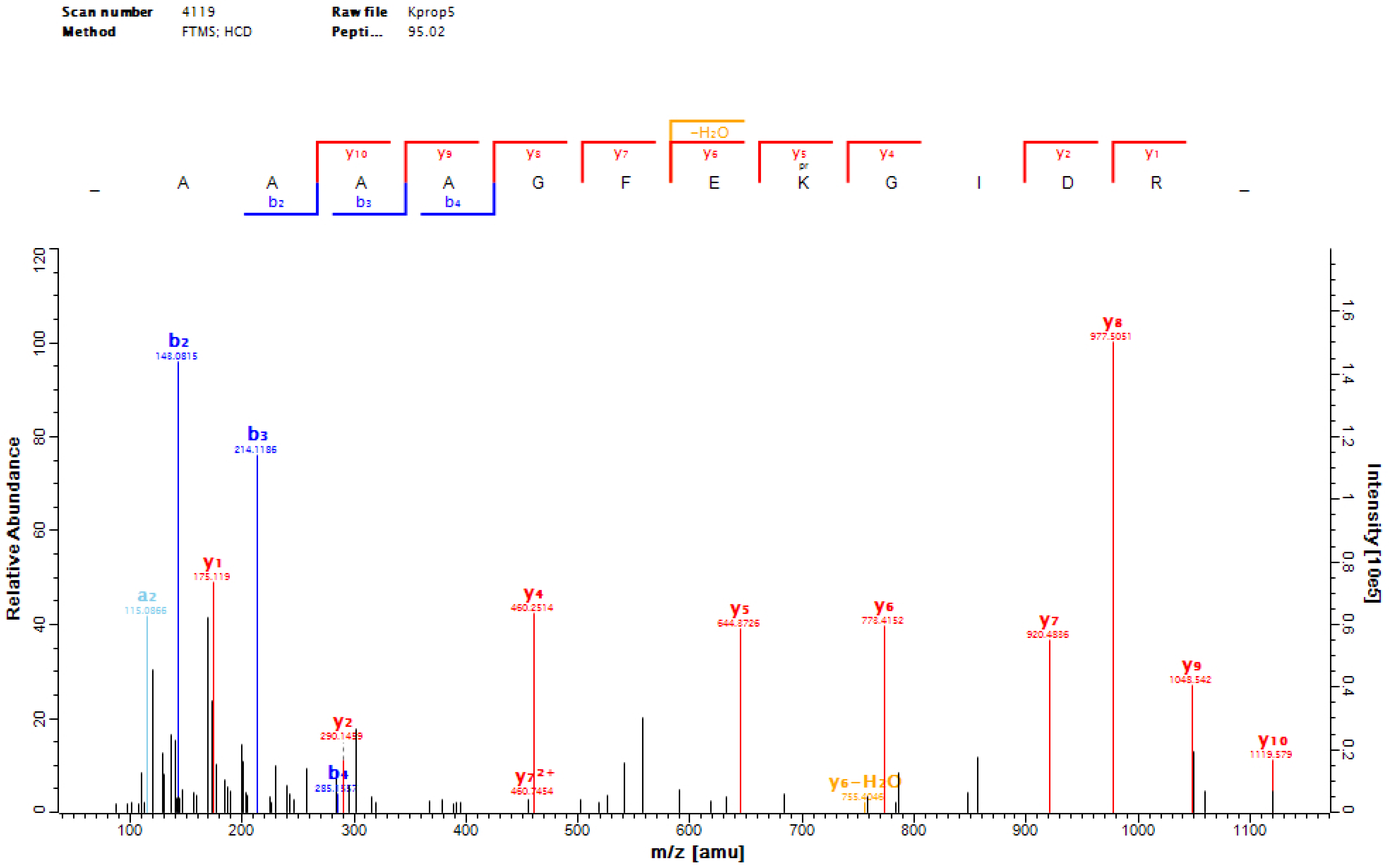
Scan number 4119
Raw file Kprop5
Method FTMS; HCD
Pepti... 95.02
_ A A A A G F E K G I D R _
-H2O
y10 y9 y8 y7 y6 y5 y4 y2 y1
b2 b3 b4
Relative Abundance
Intensity [10e5]
m/z [amu]
b2 143.0815
b3 214.1186
a2 115.0866
y1 175.119
y2 290.1459
b4 285.1557
y4 460.2514
y7 2+ 460.7454
y5 644.3726
y6 773.4152
y6-H2O 755.4046
y7 920.4836
y8 977.5051
y9 1048.542
y10 1119.579

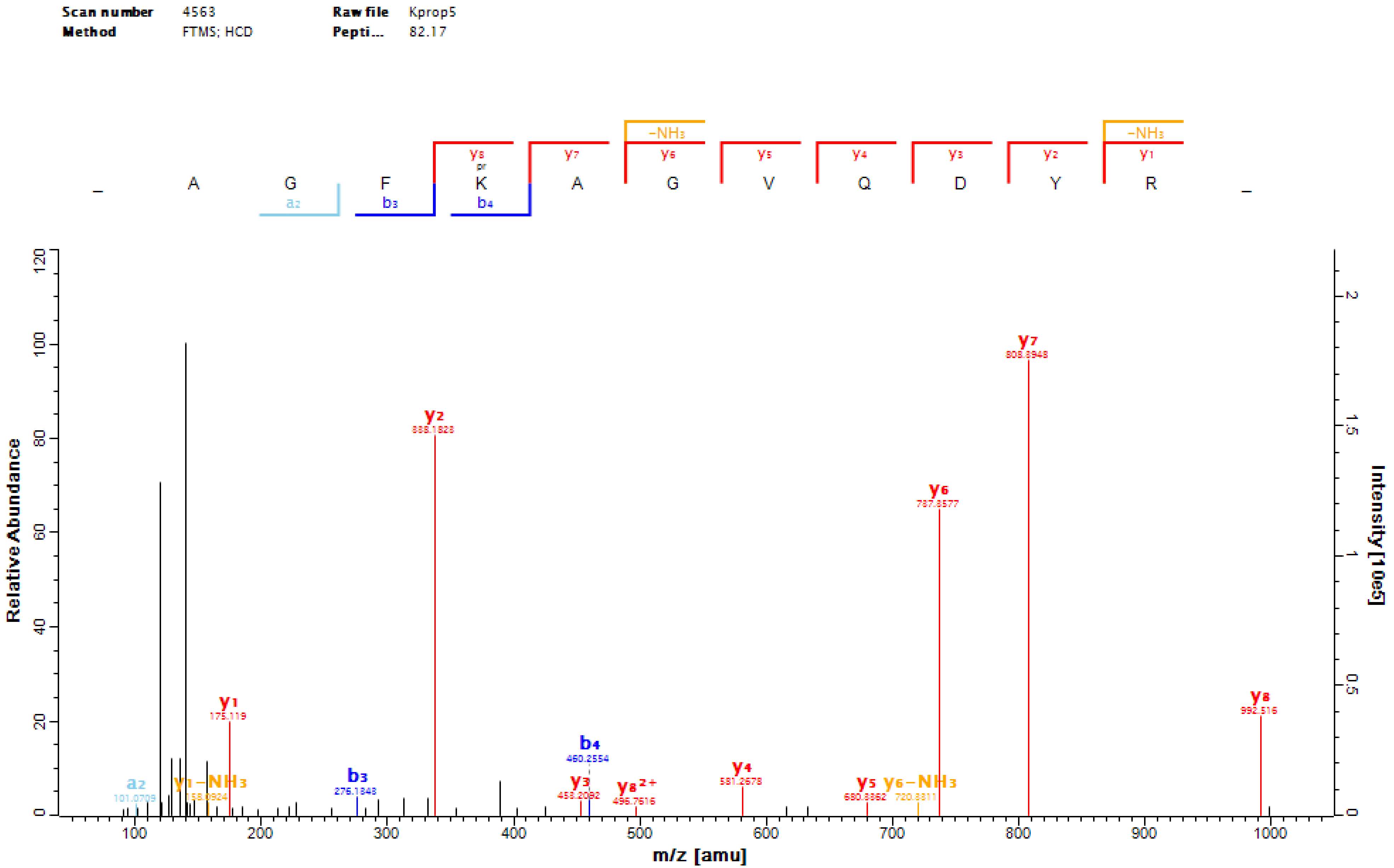
Scan number 4563
Method FTMS; HCD
Raw file Kprop5
Pepti... 82.17
A G F K A G V Q D Y R
a2 b3 b4
y8 y7 y6 y5 y4 y3 y2 y1
-NH3
Relative Abundance
Intensity [10e5]
m/z [amu]
a2
101.0709
y1-NH3
y1
175.119
b3
y2
b4
460.2554
y3
y8 2+
496.7616
y4
581.2678
y5
y6-NH3
y6
y7
y8
992.516

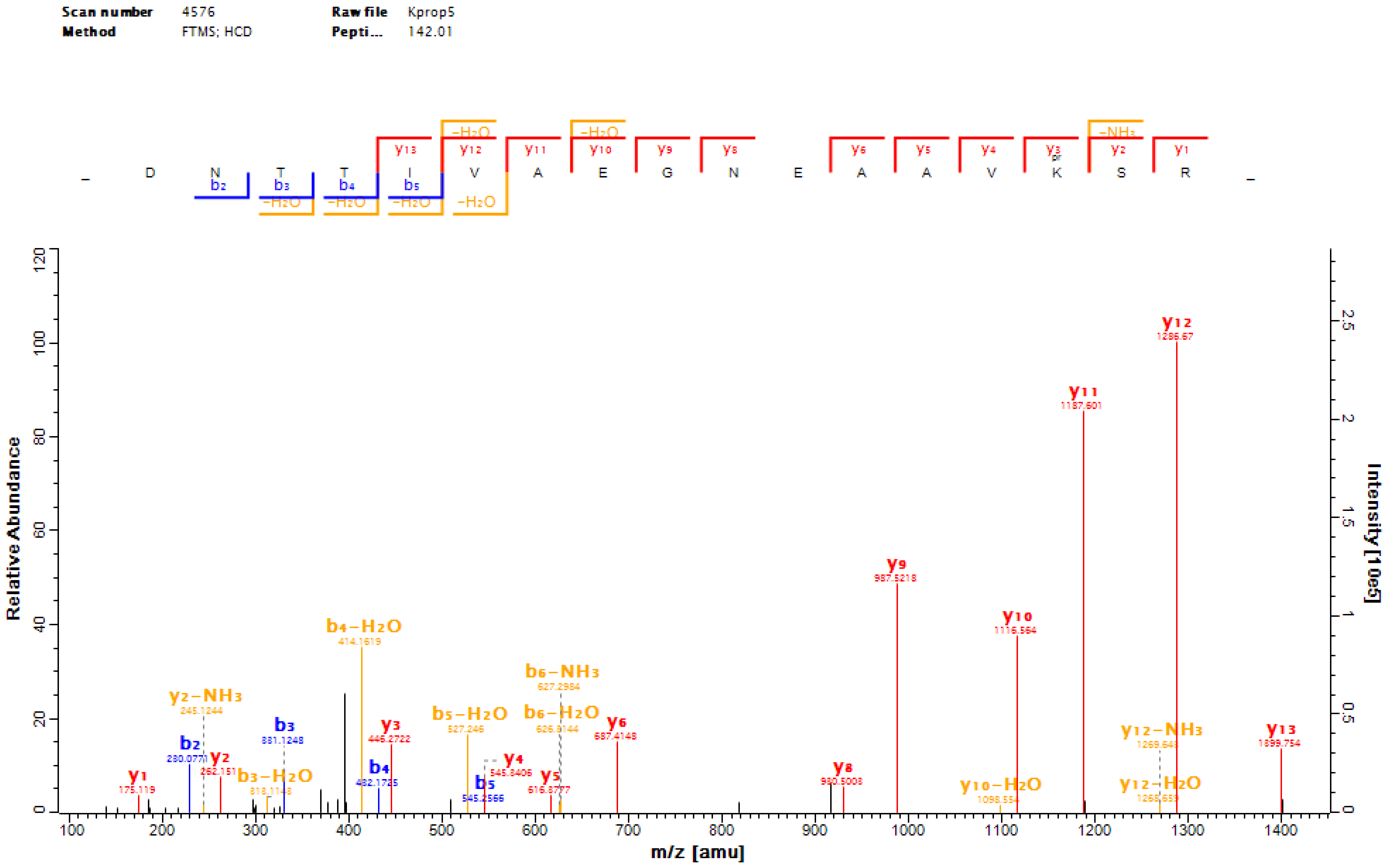

Scan number 4576
Method FTMS; HCD
Raw file Kprop5
Pepti... 142.01
_ D N T T I V A E G N E A A V K S R _
Relative Abundance
Intensity [10e5]
m/z [amu]
y1 175.119
b2 230.0771
y2-NH3 245.1244
y2 262.151
b3-H2O 313.1143
b3 331.1248
b4-H2O 414.1619
b4 432.1725
y3 446.2722
b5-H2O 527.246
b5 545.2566
y4 545.3406
y5 616.3777
b6-H2O 626.3144
b6-NH3 627.2984
y6 687.4148
y8 930.5008
y9 987.5218
y10-H2O 1098.554
y10 1116.564
y11 1187.601
y12-H2O 1268.659
y12-NH3 1269.643
y12 1286.67
y13 1399.754

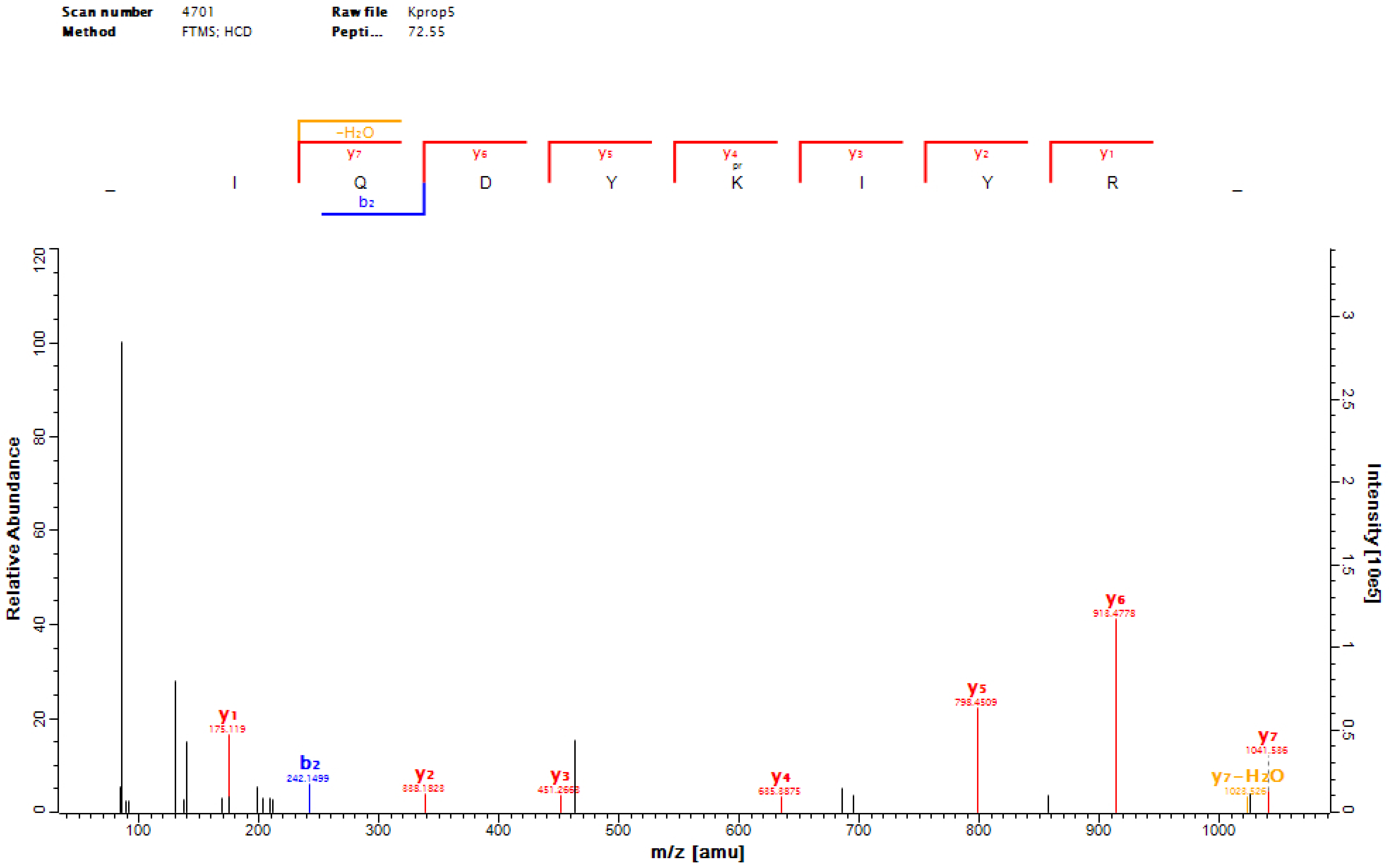

Scan number 4701
Method FTMS; HCD
Raw file Kprop5
Pepti... 72.55
_ I Q D Y K I Y R _
−H2O
y7 y6 y5 y4 y3 y2 y1
pr
b2
Relative Abundance
Intensity [10e5]
m/z [amu]
y1 175.119
b2 242.1499
y2 338.1823
y3 451.2663
y4 635.3875
y5 798.4509
y6 915.4778
y7 1041.536
y7−H2O 1023.526

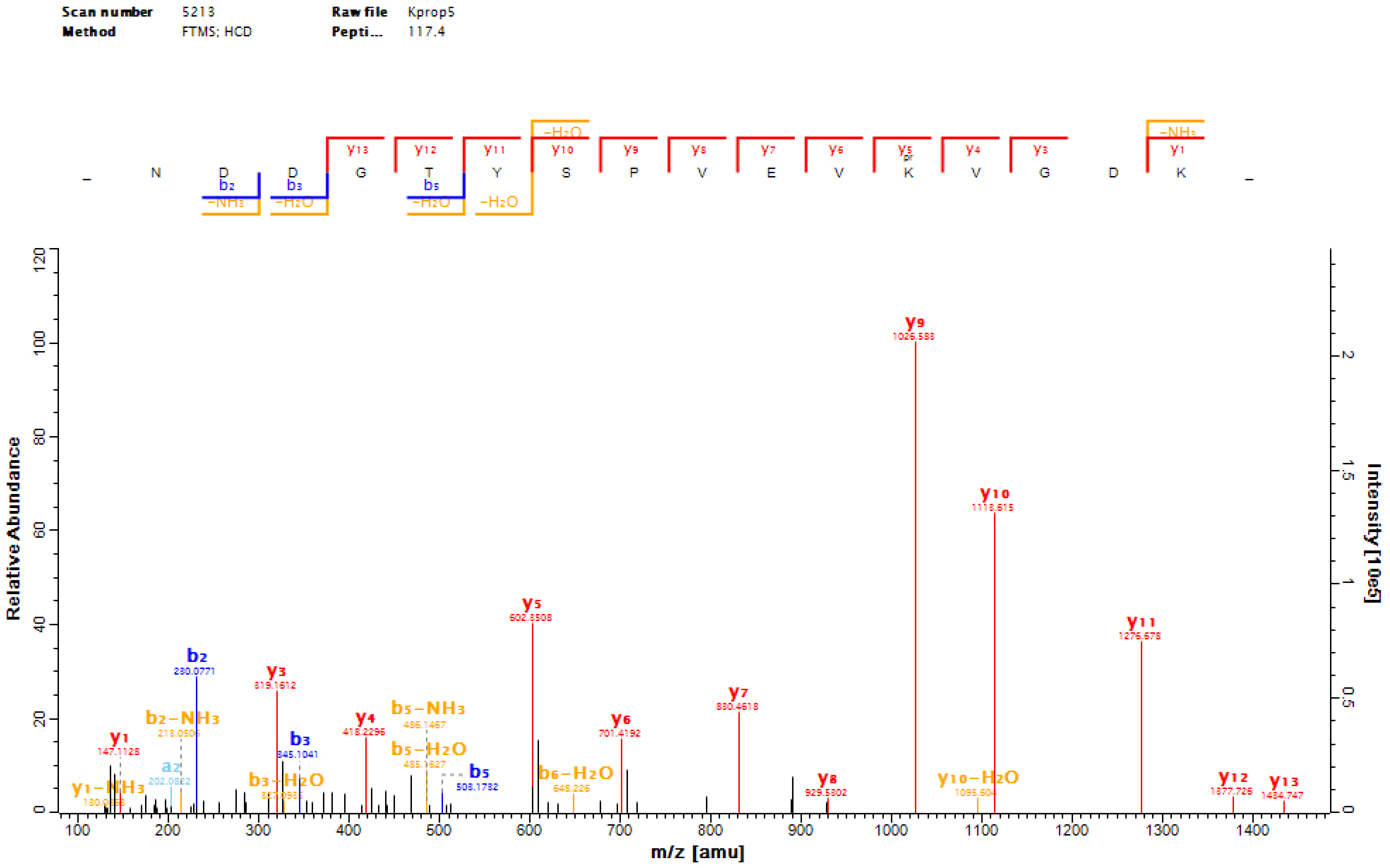

Scan number 5213
Method FTMS; HCD
Raw file Kprop5
Pepti... 117.4
_ N D D G T Y S P V E V K V G D K _
y13 y12 y11 y10 y9 y8 y7 y6 y5 y4 y3 y1
b2 b3 b5
−NH3 −H2O −H2O −H2O −H2O −NH3
Relative Abundance
Intensity [10e5]
m/z [amu]
y9 1026.583
y10 1113.615
y11 1276.678
y5 602.3508
y7 830.4618
y6 701.4192
y3 319.1612
b2 230.0771
y4 418.2296
b5−NH3 486.1467
b2−NH3 213.0506
b3 345.1041
b5−H2O 485.1627
y1 147.1128
a2 202.0822
b5 503.1782
b6−H2O 648.226
b3−H2O 327.0985
y1−NH3 130.0863
y8 929.5302
y10−H2O 1095.604
y12 1377.726
y13 1434.747

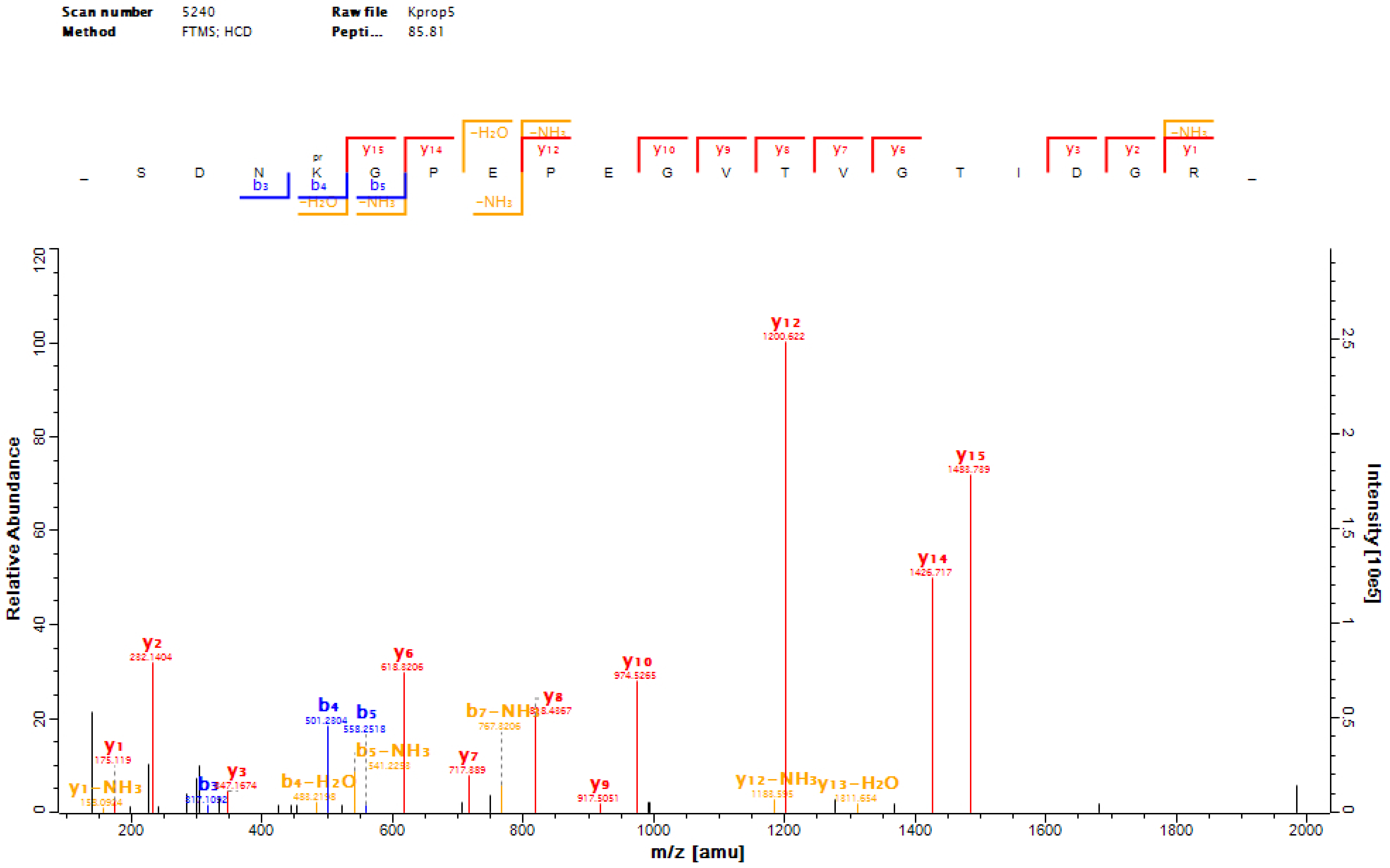

Scan number 5240
Method FTMS; HCD
Raw file Kprop5
Pepti... 85.81
_ S D N K G P E P E G V T V G T I D G R _
pr
y15 y14 y12 y10 y9 y8 y7 y6 y3 y2 y1
b3 b4 b5
-H2O -NH3
y12 1200.622
y15 1483.739
y14 1426.717
y10 974.5265
y2 232.1404
y6 618.3206
b4 501.2304
b5 558.2518
b7-NH3 767.3206
y8
b5-NH3 541.2253
y1 175.119
y7 717.389
y3 347.1674
b4-H2O 483.2198
y1-NH3
b3 317.1092
y9 917.5051
y12-NH3 1183.595
y13-H2O 1311.654
Relative Abundance
Intensity [10e5]
m/z [amu]

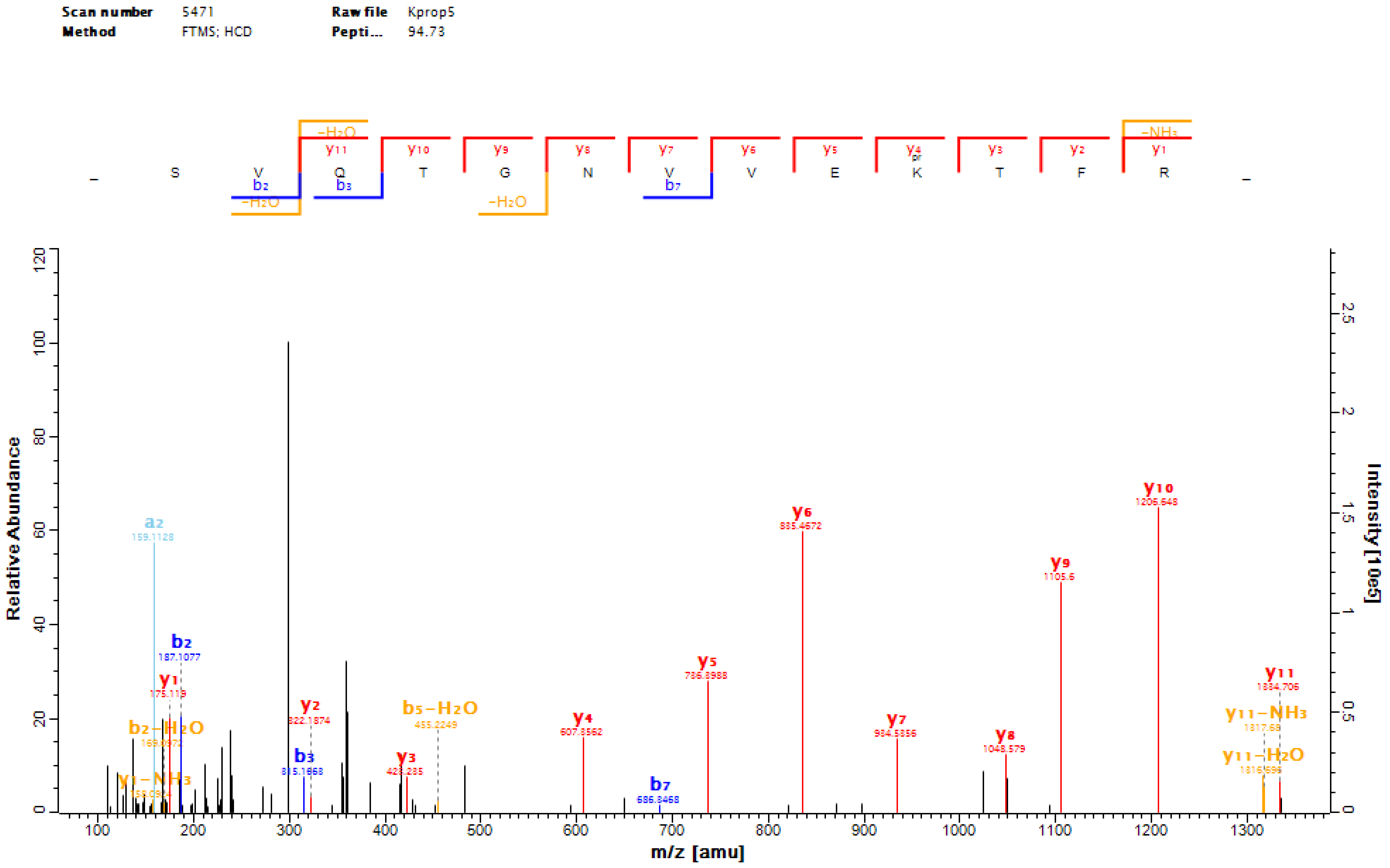

Scan number 5471
Raw file Kprop5
Method FTMS; HCD
Pepti... 94.73
_ S V Q T G N V V E K T F R _
y11 y10 y9 y8 y7 y6 y5 y4 y3 y2 y1
b2 b3 b7
-H2O
-NH3
Relative Abundance
Intensity [10e5]
m/z [amu]
a2 159.1128
b2 187.1077
y1 175.119
b2-H2O 169.0972
y1-NH3 158.0924
y2 322.1874
b3 315.1663
y3 423.235
b5-H2O 455.2249
y4 607.3562
b7 686.3468
y5 736.3988
y6 835.4672
y7 934.5356
y8 1048.579
y9 1105.6
y10 1206.648
y11 1334.706
y11-NH3 1317.68
y11-H2O 1316.696

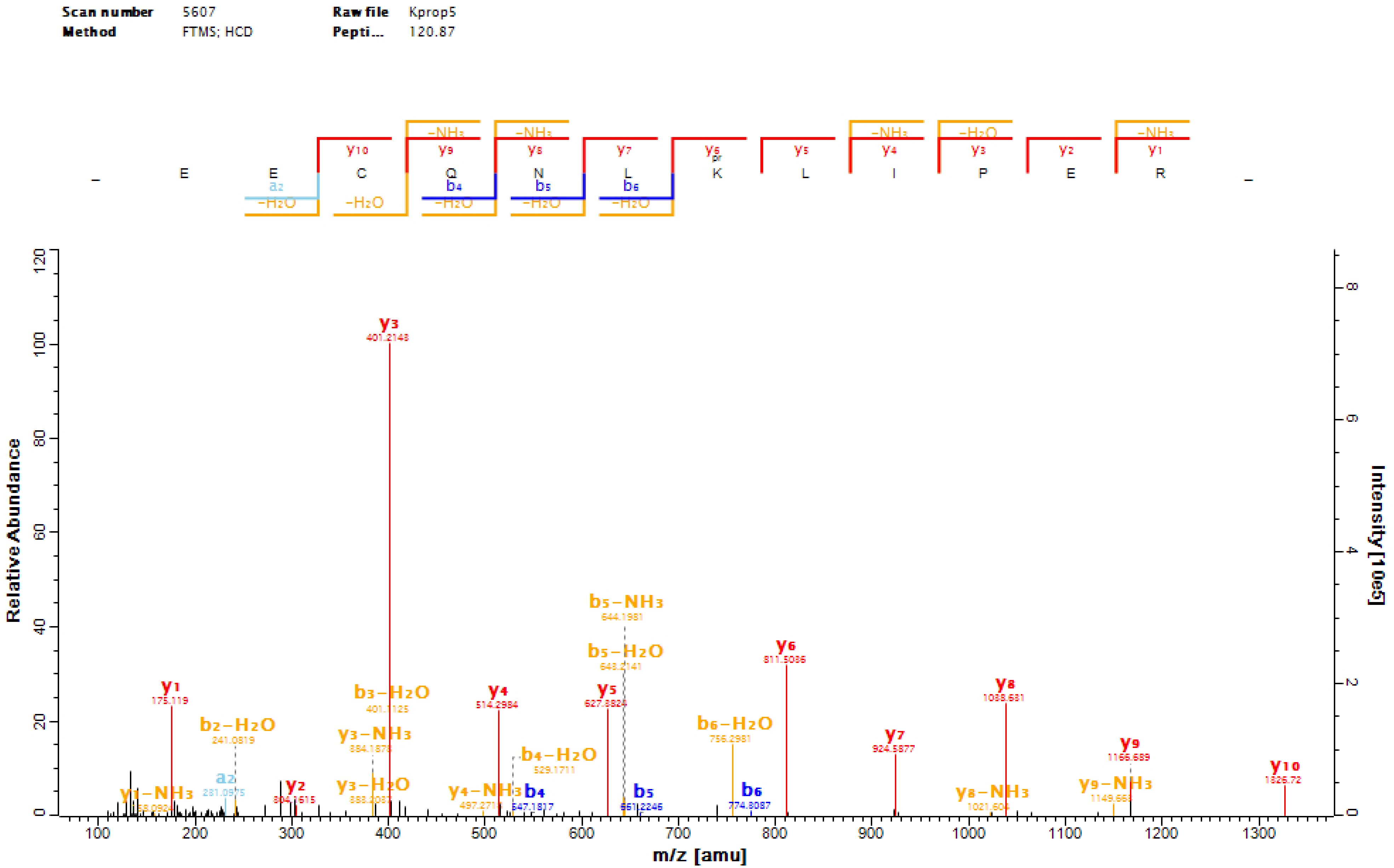

Scan number 5607
Method FTMS; HCD
Raw file Kprop5
Pepti... 120.87
_ E E C Q N L K L I P E R _
y10 y9 y8 y7 y6 y5 y4 y3 y2 y1
pr
-NH3 -NH3 -NH3 -H2O -NH3
a2 b4 b5 b6
-H2O -H2O -H2O -H2O -H2O
Relative Abundance
Intensity [10e5]
m/z [amu]
y1 175.119
y1-NH3 158.0924
a2 231.0975
b2-H2O 241.0819
y2 304.1615
y3-NH3 384.1878
y3-H2O 383.2037
y3 401.2148
b3-H2O 401.1125
y4 514.2984
y4-NH3 497.2719
b4 547.1817
b4-H2O 529.1711
y5 627.3824
b5-NH3 644.1981
b5-H2O 643.2141
b5 661.2245
b6-H2O 756.2981
b6 774.3087
y6 811.5036
y7 924.5877
y8 1038.681
y8-NH3 1021.604
y9 1166.689
y9-NH3 1149.663
y10 1326.72

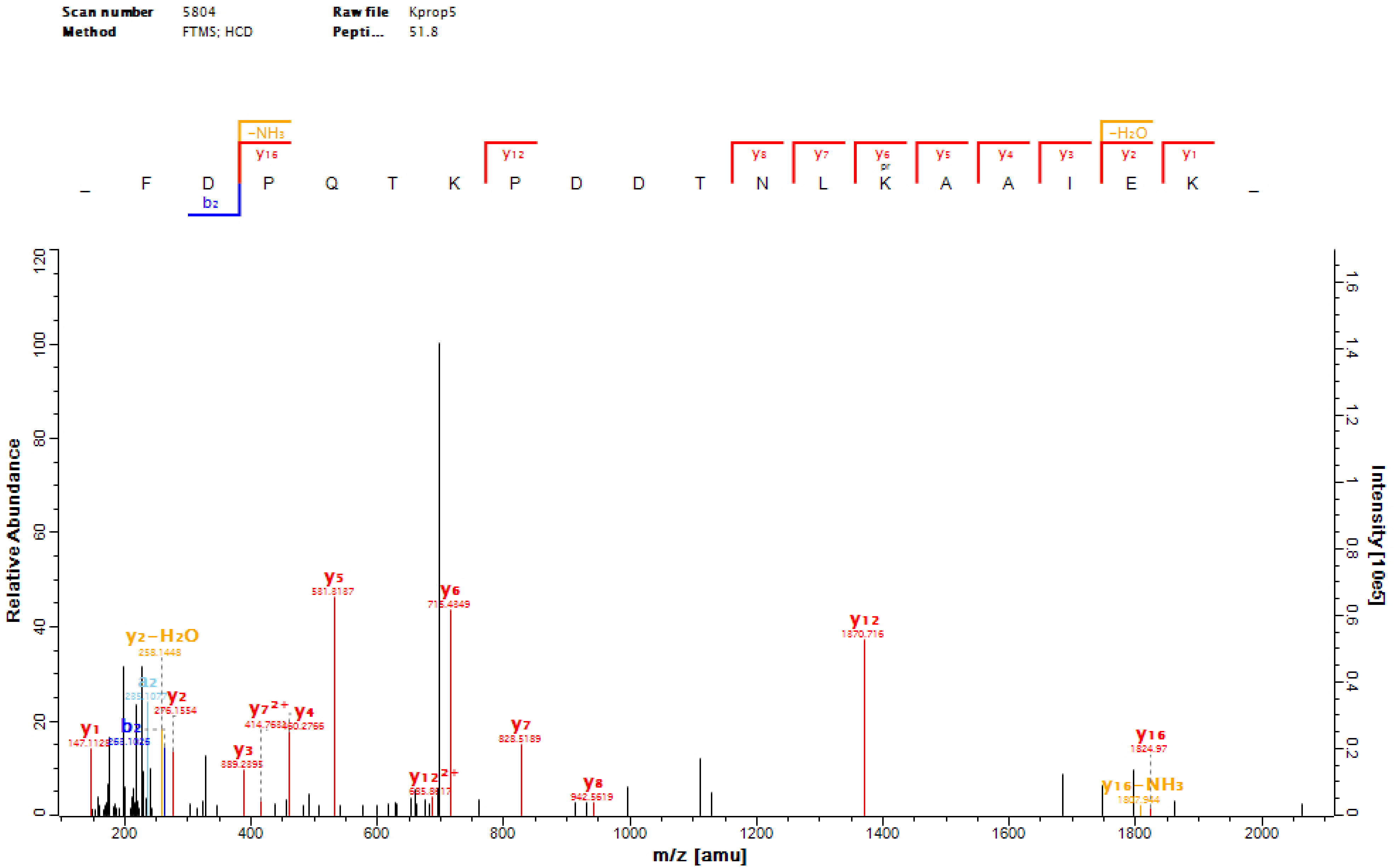
Scan number 5804
Raw file Kprop5
Method FTMS; HCD
Pepti... 51.8
_ F D P Q T K P D D T N L K A A I E K _
b2
y16
-NH3
y12
y8 y7 y6 y5 y4 y3 y2 y1
-H2O
pr
Relative Abundance
Intensity [10e5]
m/z [amu]
y1 147.1128
b2 263.1026
a2 235.1077
y2-H2O 258.1448
y2 276.1554
y3 389.2395
y7 2+ 414.7634
y4 460.2766
y5 531.3137
y6 715.4349
y12 2+ 685.8817
y7 828.5189
y8 942.5619
y12 1370.716
y16 1824.97
y16-NH3 1807.944

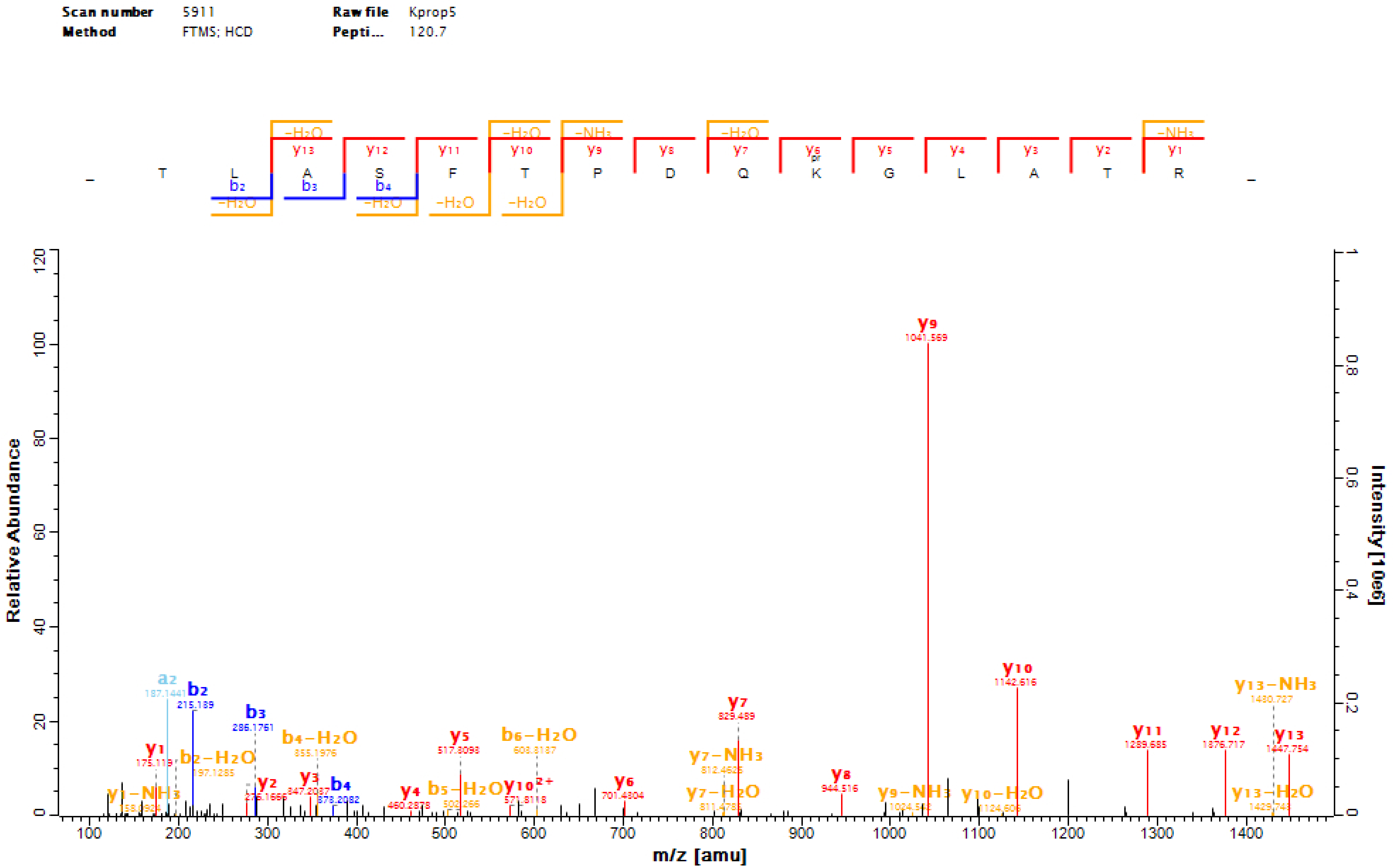

Scan number 5911
Method FTMS; HCD
Raw file Kprop5
Pepti... 120.7
T L A S F T P D Q K G L A T R
Relative Abundance
Intensity [10e6]
m/z [amu]
y9 1041.569
y10 1142.616
y13−NH3 1430.727
y7 829.489
y11 1289.685
y12 1376.717
y13 1447.754
a2 187.1441
b2 215.139
b3 286.1761
b4−H2O 355.1976
b6−H2O 603.3187
y5 517.3093
y1 175.119
b2−H2O 197.1285
y7−NH3 812.4626
y2 276.1666
y3 347.2037
b4 373.2082
y4 460.2878
b5−H2O 502.266
y10 2+ 571.8118
y6 701.4304
y7−H2O 811.4785
y8 944.516
y9−NH3 1024.542
y10−H2O 1124.605
y13−H2O 1429.743
y1−NH3 158.0924

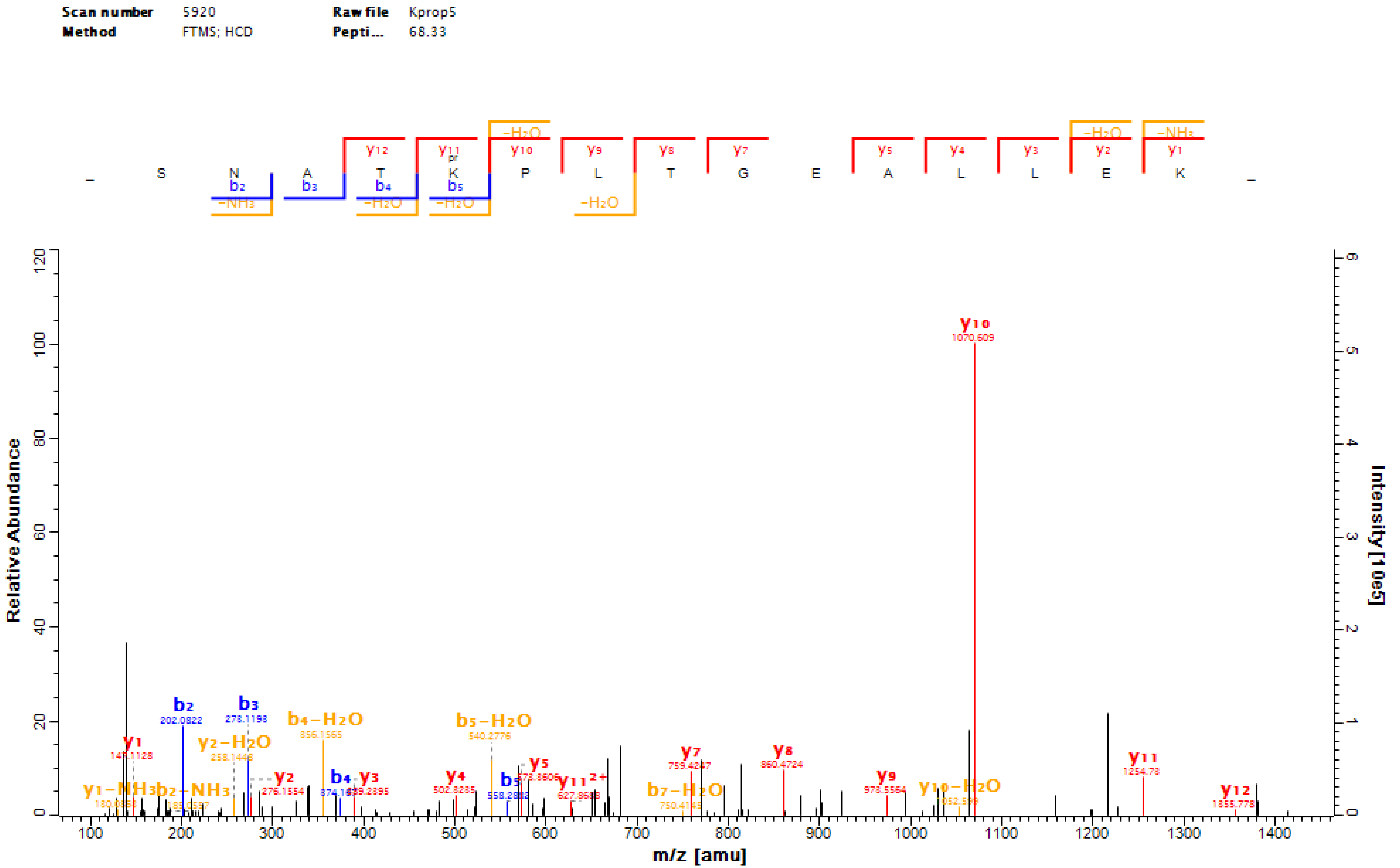
Scan number 5920
Raw file Kprop5
Method FTMS; HCD
Pepti... 68.33
_ S N A T K P L T G E A L L E K _
y12 y11 y10 y9 y8 y7 y5 y4 y3 y2 y1
b2 b3 b4 b5
-NH3 -H2O
y10 1070.609
b2 202.0822
b3 278.1198
b4-H2O 356.1565
b5-H2O 540.2776
y1
y2-H2O
y2 276.1554
y3
y4 502.3235
y5
y7 759.4247
y8 860.4724
y9 973.5564
y10-H2O
y11 1254.73
y12 1355.778
b7-H2O 750.4145
y1-NH3
b2-NH3
b4
b5
Relative Abundance
Intensity [10e5]
m/z [amu]

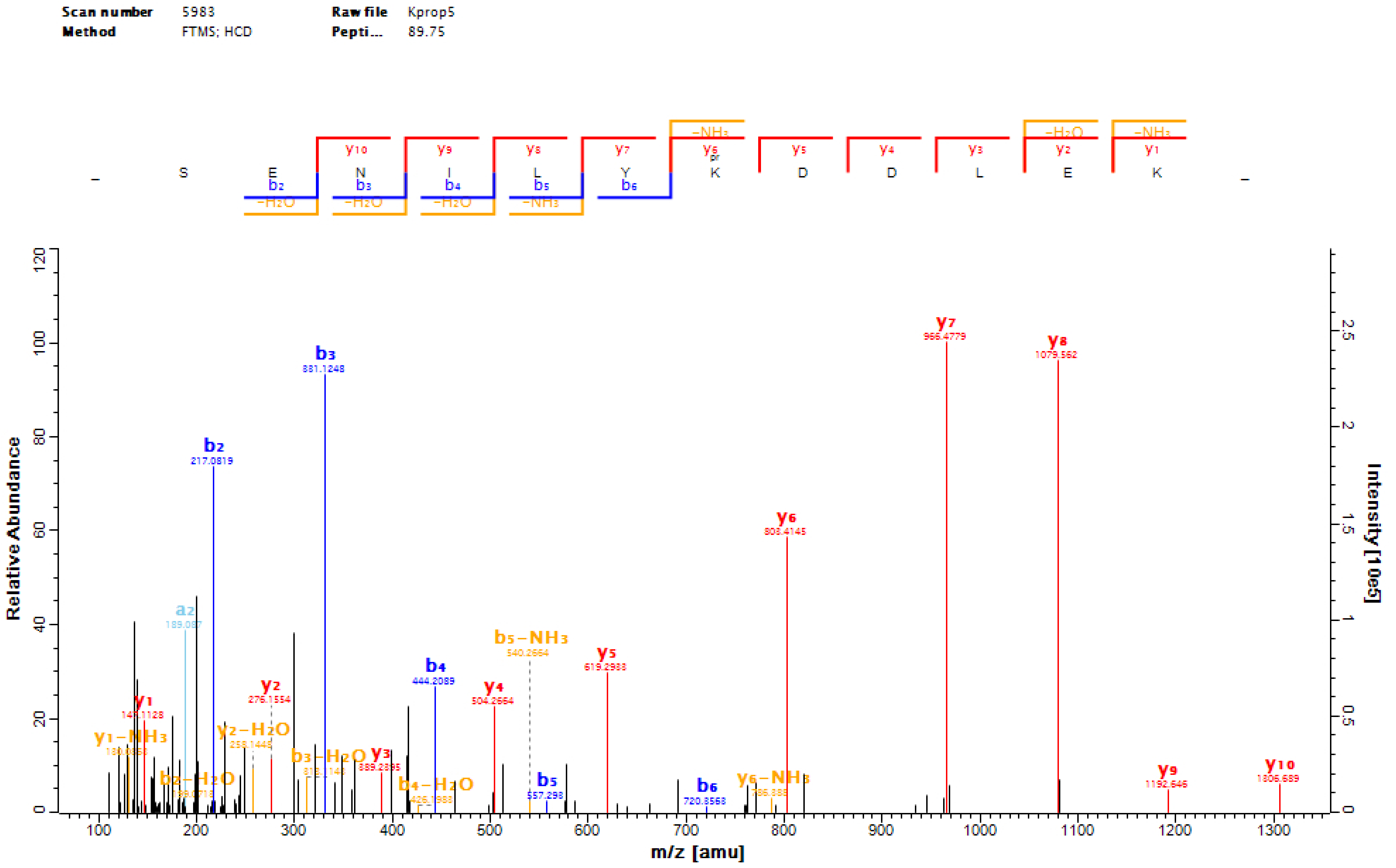
Scan number 5983
Raw file Kprop5
Method FTMS; HCD
Pepti... 89.75
_ S E N I L Y K D D L E K _
y10 y9 y8 y7 y6 y5 y4 y3 y2 y1
b2 b3 b4 b5 b6
–H2O –NH3
b3
331.1248
b2
217.0819
a2
189.087
y1
147.1128
y1–NH3
y2
276.1554
y2–H2O
258.1448
b2–H2O
y3
389.2395
b3–H2O
b4
444.2089
b4–H2O
y4
504.2664
b5–NH3
540.2664
b5
557.293
y5
619.2933
b6
720.3563
y6–NH3
y6
803.4145
y7
966.4779
y8
1079.562
y9
1192.646
y10
1306.689
Relative Abundance
Intensity [10e5]
m/z [amu]

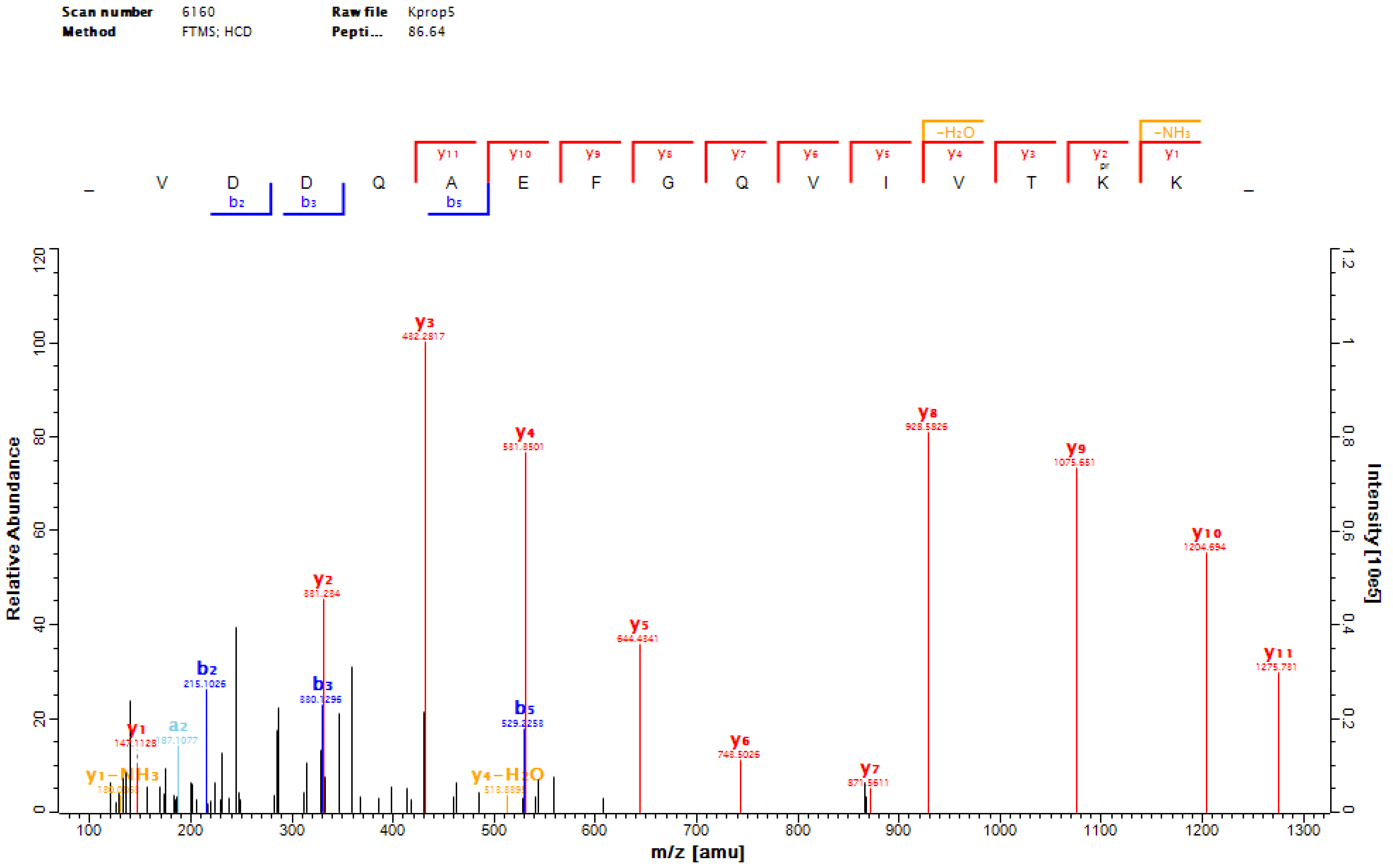

Scan number 6160
Method FTMS; HCD
Raw file Kprop5
Pepti... 86.64
_ V D D Q A E F G Q V I V T K K _
b2 b3 b5
y11 y10 y9 y8 y7 y6 y5 y4 y3 y2 y1
-H2O
-NH3
y3 432.2817
y8 928.5826
y4 531.3501
y9 1075.651
y10 1204.694
y2 331.234
y5 644.4341
y11 1275.731
b2 215.1026
b3 330.1296
b5 529.2258
a2 187.1077
y1 147.1128
y6 743.5026
y7 871.5611
y1-NH3
y4-H2O
Relative Abundance
Intensity [10e5]
m/z [amu]

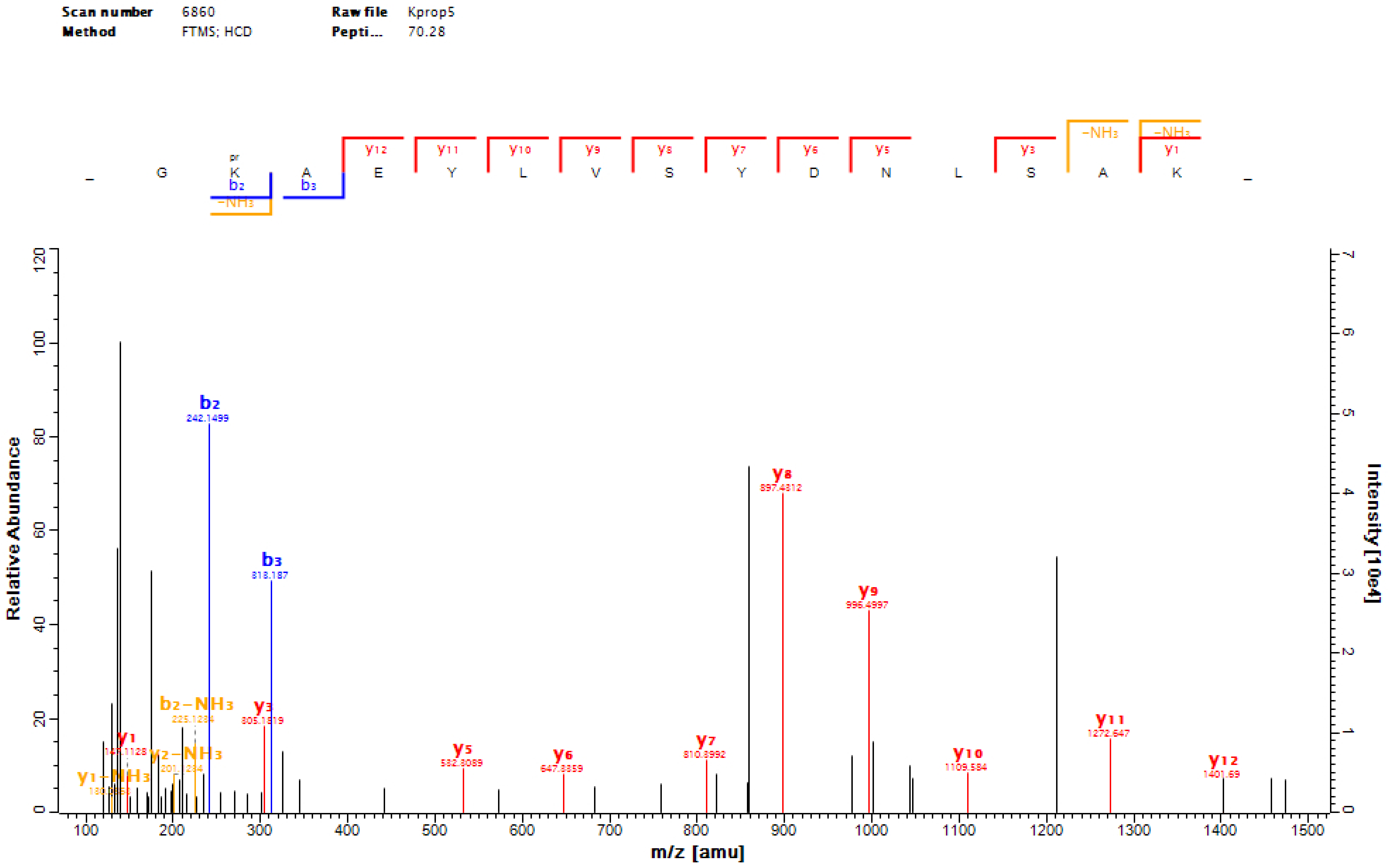
Scan number 6860
Method FTMS; HCD
Raw file Kprop5
Pepti... 70.28
_ G K A E Y L V S Y D N L S A K _
pr
b2
b3
-NH3
y12 y11 y10 y9 y8 y7 y6 y5 y3 y1
Relative Abundance
Intensity [10e4]
m/z [amu]
b2
242.1499
b3
313.187
y8
897.4312
y9
996.4997
b2-NH3
225.1234
y3
305.1819
y1
147.1128
y2-NH3
y1-NH3
y5
532.3089
y6
647.3359
y7
810.3992
y10
1109.584
y11
1272.647
y12
1401.69

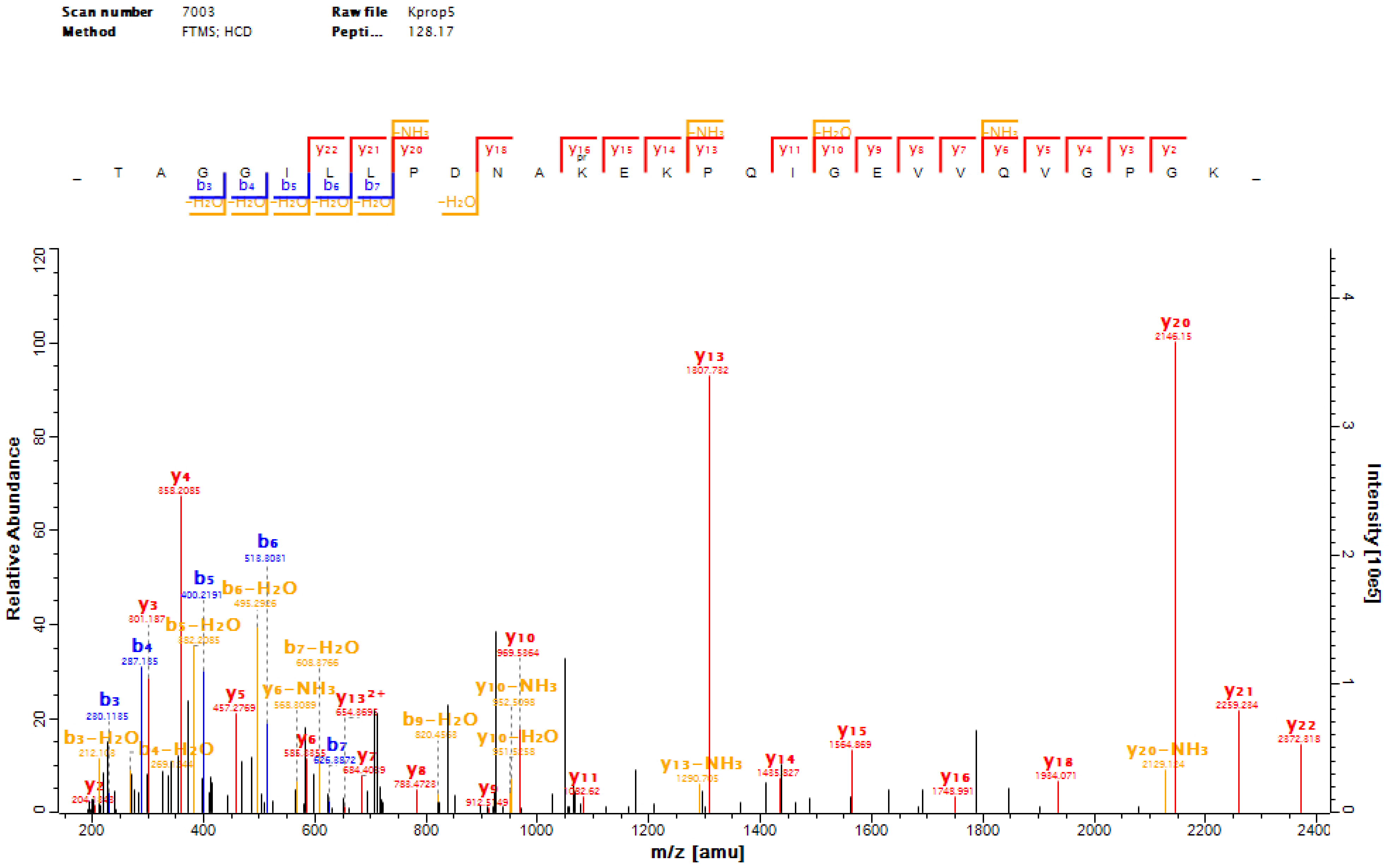

Scan number 7003
Method FTMS; HCD
Raw file Kprop5
Pepti... 128.17
_ T A G G I L L P D N A K E K P Q I G E V V Q V G P G K _
Relative Abundance
Intensity [10e5]
m/z [amu]
y20
2146.15
y13
1307.782
y4
b6
b5
y3
b4
b3
y2
y5
y6
b7
y7
y8
y9
y10
y11
y14
y15
y16
y18
y21
y22
b3−H2O
b4−H2O
b5−H2O
b6−H2O
b7−H2O
b9−H2O
y6−NH3
y10−NH3
y10−H2O
y13−NH3
y20−NH3
y13 2+

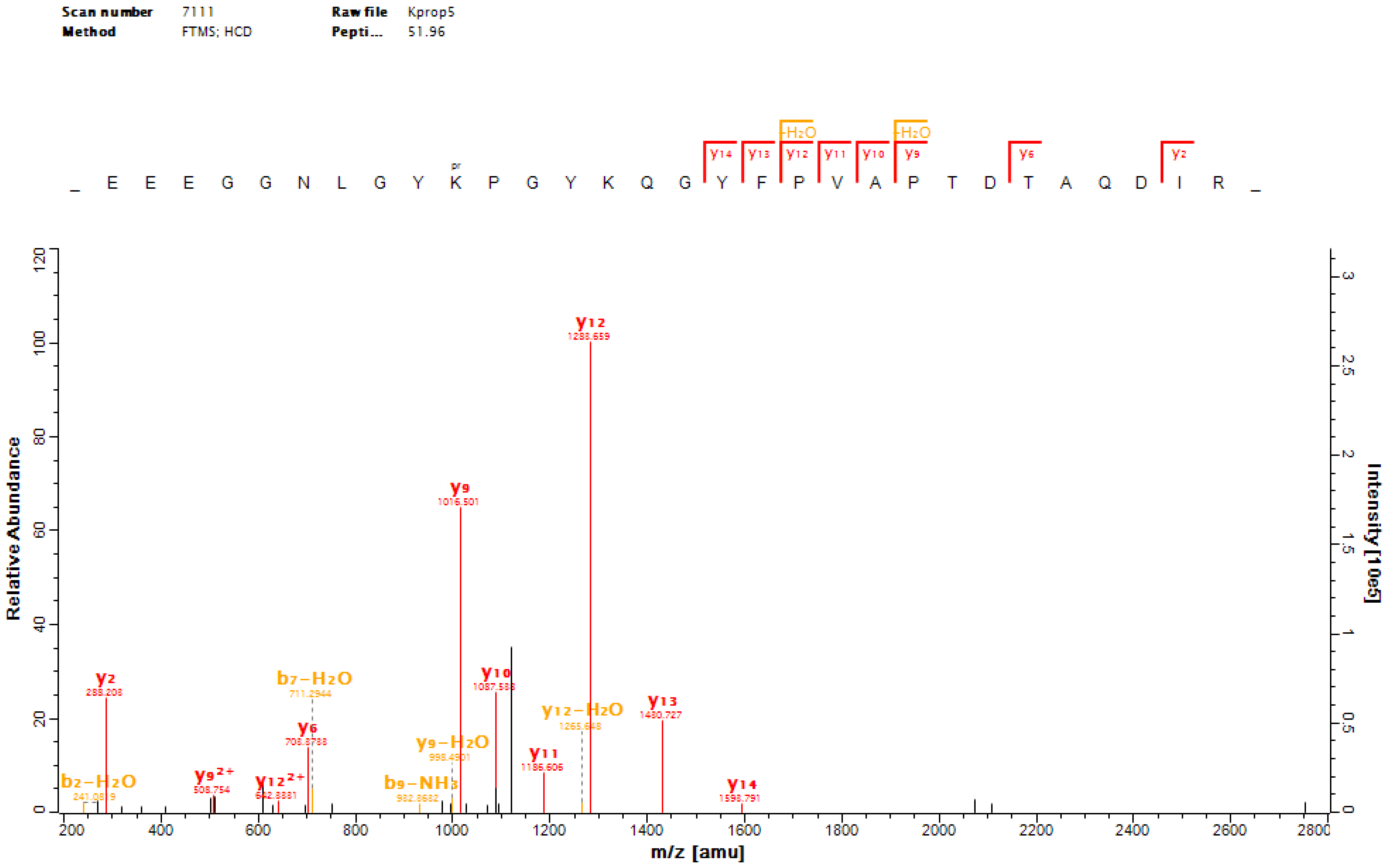

Scan number 7111
Method FTMS; HCD
Raw file Kprop5
Pepti... 51.96
_ E E E G G N L G Y K P G Y K Q G Y F P V A P T D T A Q D I R _
pr
y14 y13 y12 y11 y10 y9 y6 y2
-H2O -H2O
y12 1283.659
y9 1016.501
y2 288.203
b7-H2O 711.2944
y10 1087.538
y12-H2O 1265.648
y13 1430.727
y6 703.8783
y9-H2O 998.4901
y11 1186.606
y9 2+ 508.754
y12 2+ 642.3331
b2-H2O 241.0819
b9-NH3 932.3632
y14 1593.791
Relative Abundance
Intensity [10e5]
m/z [amu]

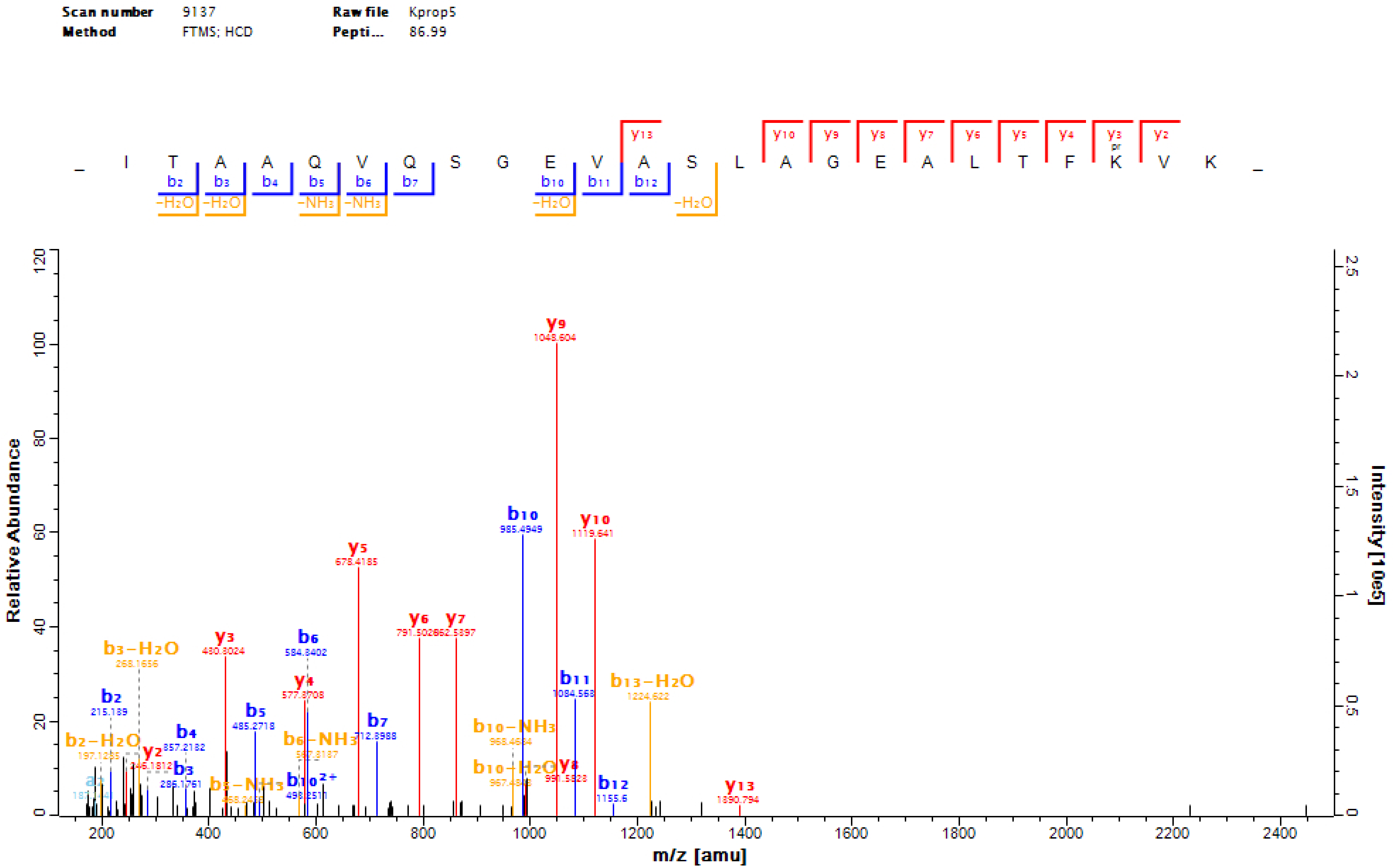
Scan number 9137
Raw file Kprop5
Method FTMS; HCD
Pepti... 86.99
_ I T A A Q V Q S G E V A S L A G E A L T F K V K _
Relative Abundance
Intensity [10e5]
m/z [amu]
y9 1048.604
b10 985.4949
y10 1119.641
y5 678.4185
y6 791.502
y7 862.5397
y3 430.3024
b6 584.3402
b3-H2O 268.1656
y4 577.3708
b11 1084.568
b13-H2O 1224.622
b2 215.139
b5 485.2718
b7 712.3988
b4 357.2182
b2-H2O 197.1285
y2 246.1812
b3 286.1761
b10-NH3 968.4684
b10-H2O 967.4843
y8 991.5823
b12 1155.6
y13 1390.794

Scan number 2098 **Raw file** Kprop6
Method FTMS; HCD **Pepti...** 39.62

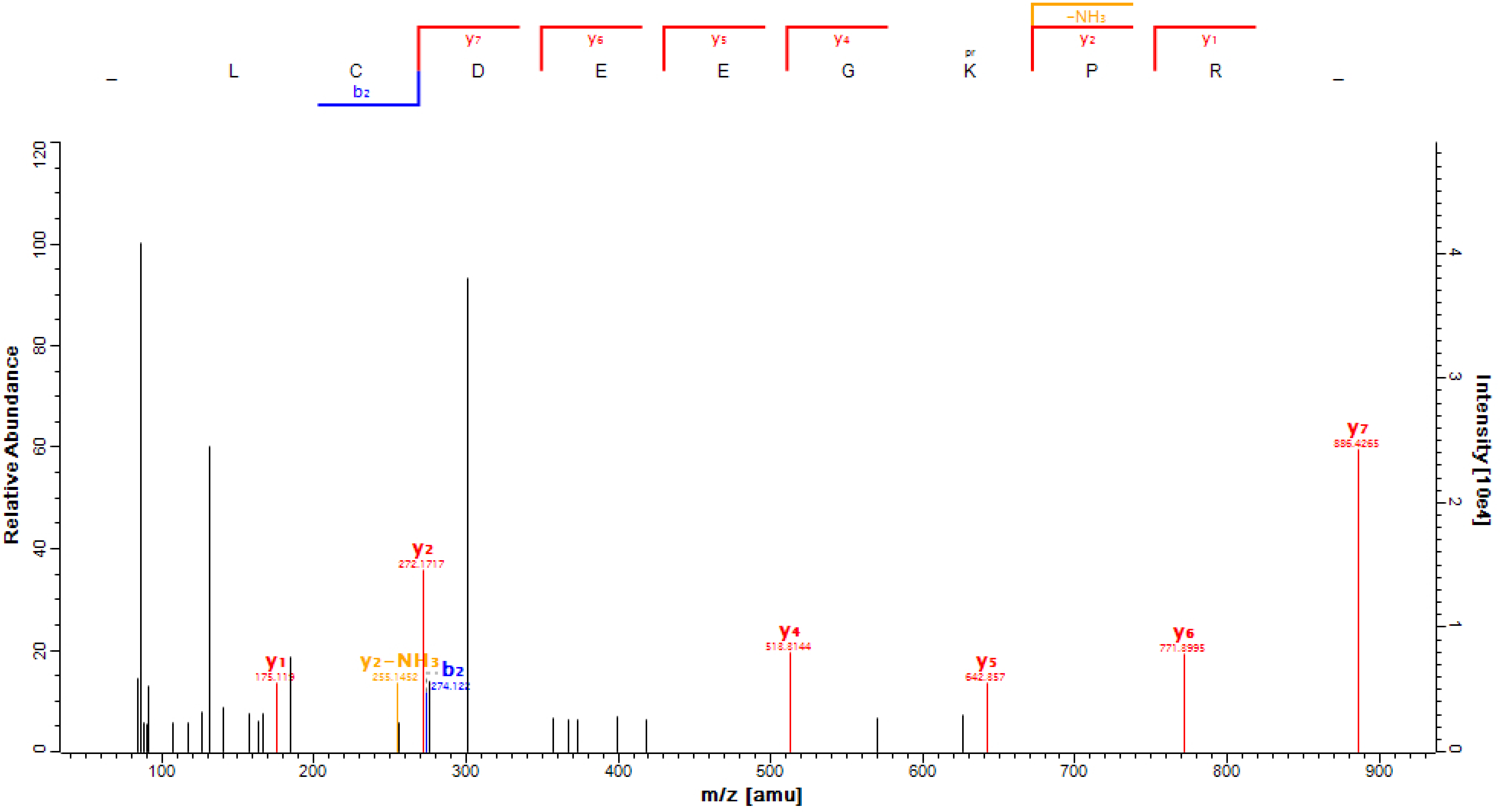

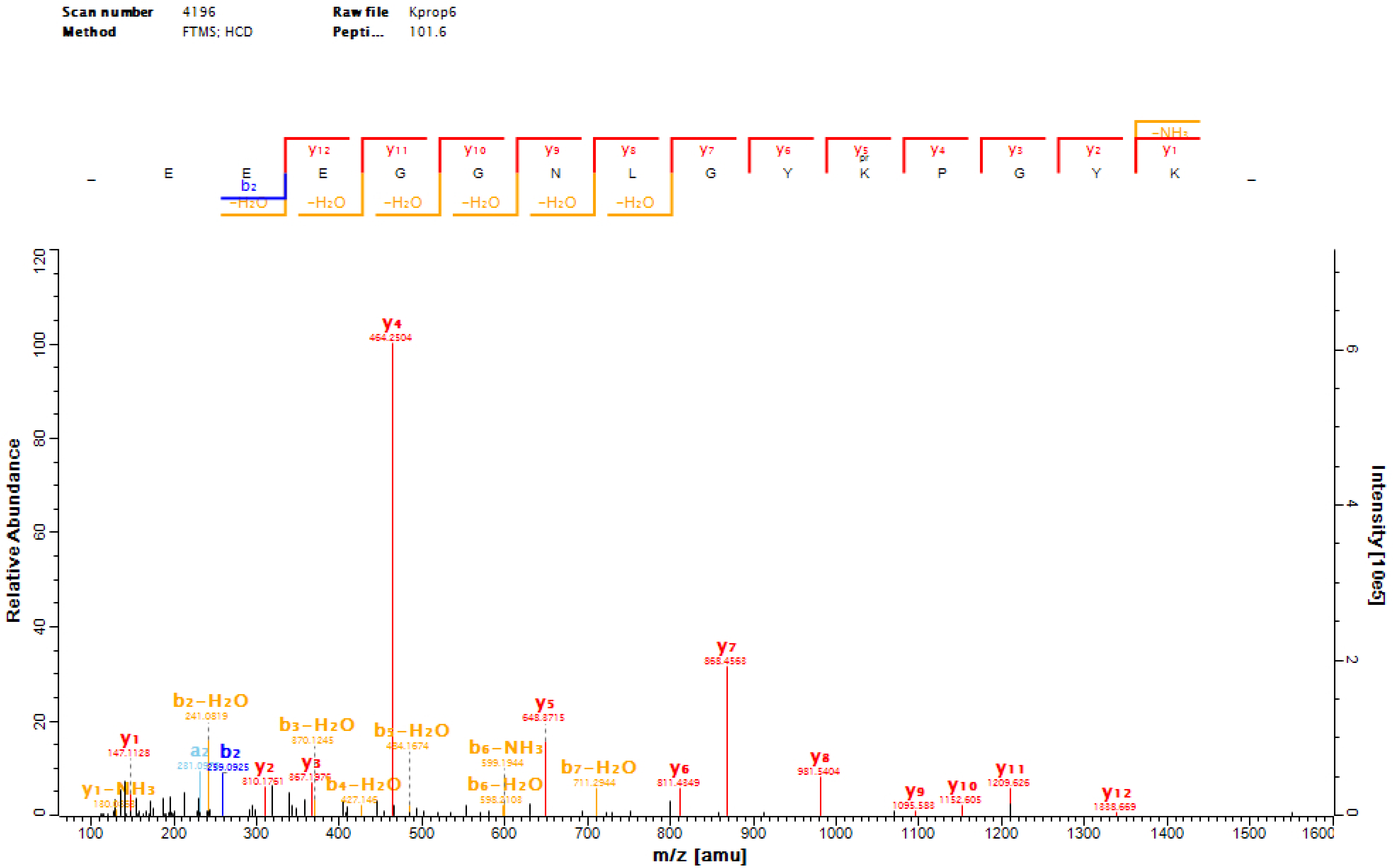

Scan number 4196
Raw file Kprop6
Method FTMS; HCD
Pepti... 101.6
_ E E E G G N L G Y K P G Y K _
Relative Abundance
Intensity [10e5]
m/z [amu]
y4 464.2504
y7 868.4563
y5 648.8715
y8 981.5404
y1 147.1128
b2−H2O 241.0819
b2 259.0925
y2 310.1751
y3 367.1975
b3−H2O 370.1245
b4−H2O 427.146
b5−H2O 484.1674
b6−NH3 599.1944
b6−H2O 598.2103
b7−H2O 711.2944
y6 811.4849
y9 1095.583
y10 1152.605
y11 1209.626
y12 1338.669
y1−NH3

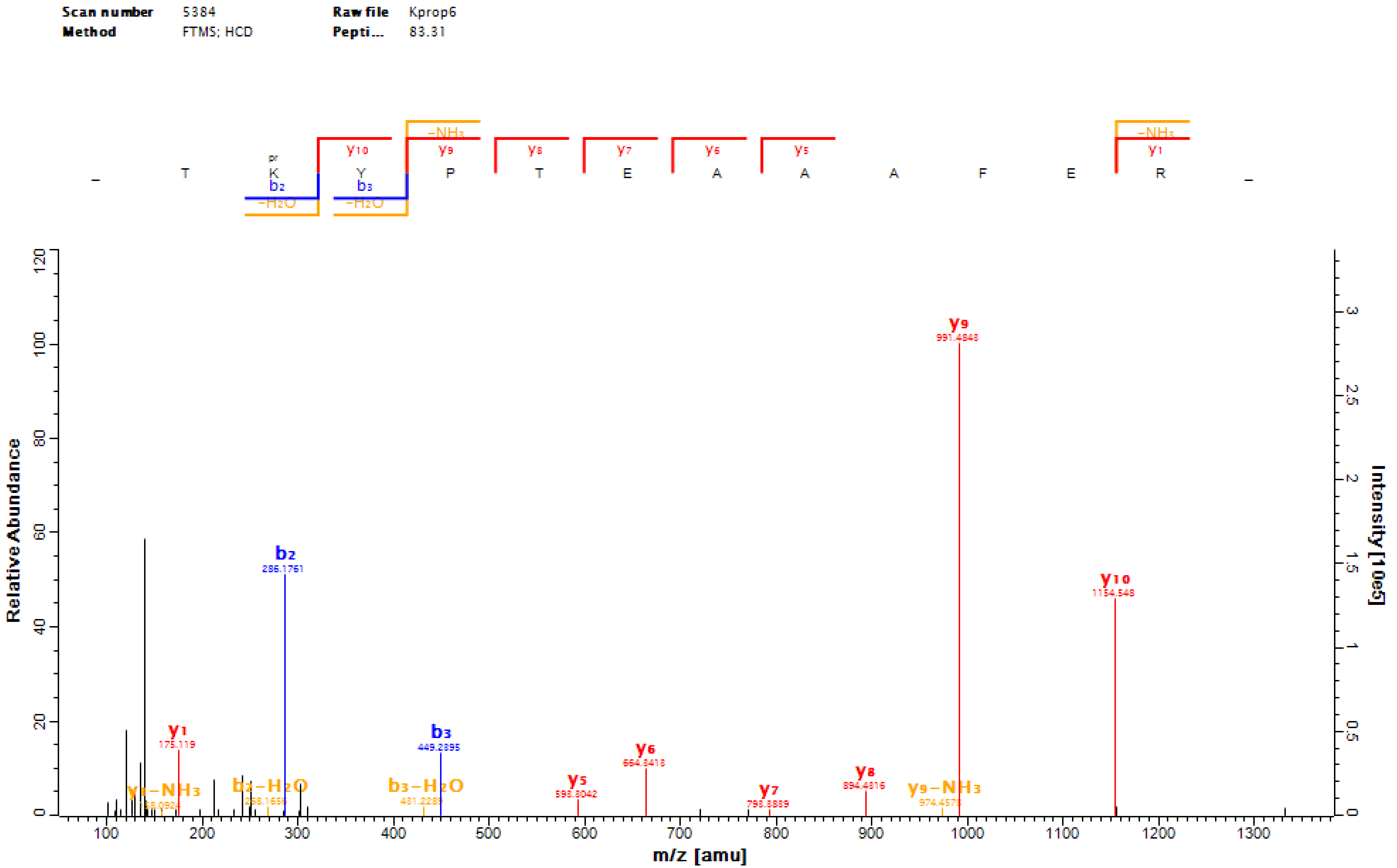

Scan number 5384
Method FTMS; HCD
Raw file Kprop6
Pepti... 83.31
_ T K Y P T E A A A F E R _
pr
y10 y9 y8 y7 y6 y5 y1
-NH3
b2 b3
-H2O
Relative Abundance
Intensity [10e5]
m/z [amu]
y1 175.119
b2 286.1761
b3 449.2395
y5 593.3042
y6 664.3418
y8 894.4316
y9 991.4843
y10 1154.548
b2-H2O
b3-H2O
431.2289
y9-NH3
974.4578

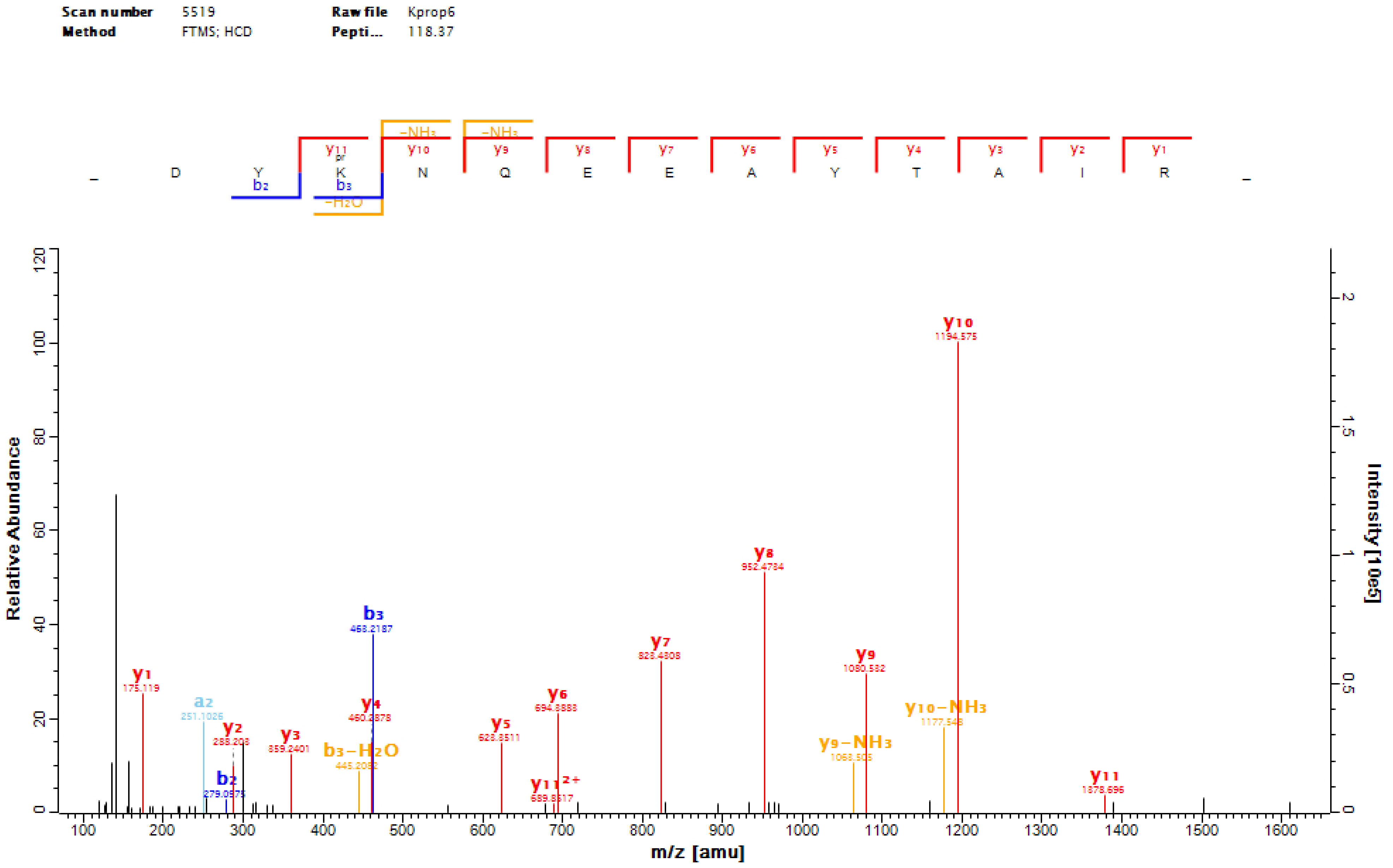

Scan number 5519
Method FTMS; HCD
Raw file Kprop6
Pepti... 118.37
_ D Y K N Q E E A Y T A I R _
b2 b3 -H2O
y11 y10 y9 y8 y7 y6 y5 y4 y3 y2 y1
-NH3 -NH3
y1 175.119
a2 251.1026
b2 279.0975
y2 288.203
y3 359.2401
b3-H2O 445.2082
y4 460.2878
b3 463.2187
y5 623.3511
y11 2+ 689.8817
y6 694.3883
y7 823.4308
y8 952.4734
y9-NH3 1063.505
y9 1080.532
y10-NH3 1177.548
y10 1194.575
y11 1378.696
Relative Abundance
Intensity [10e5]
m/z [amu]

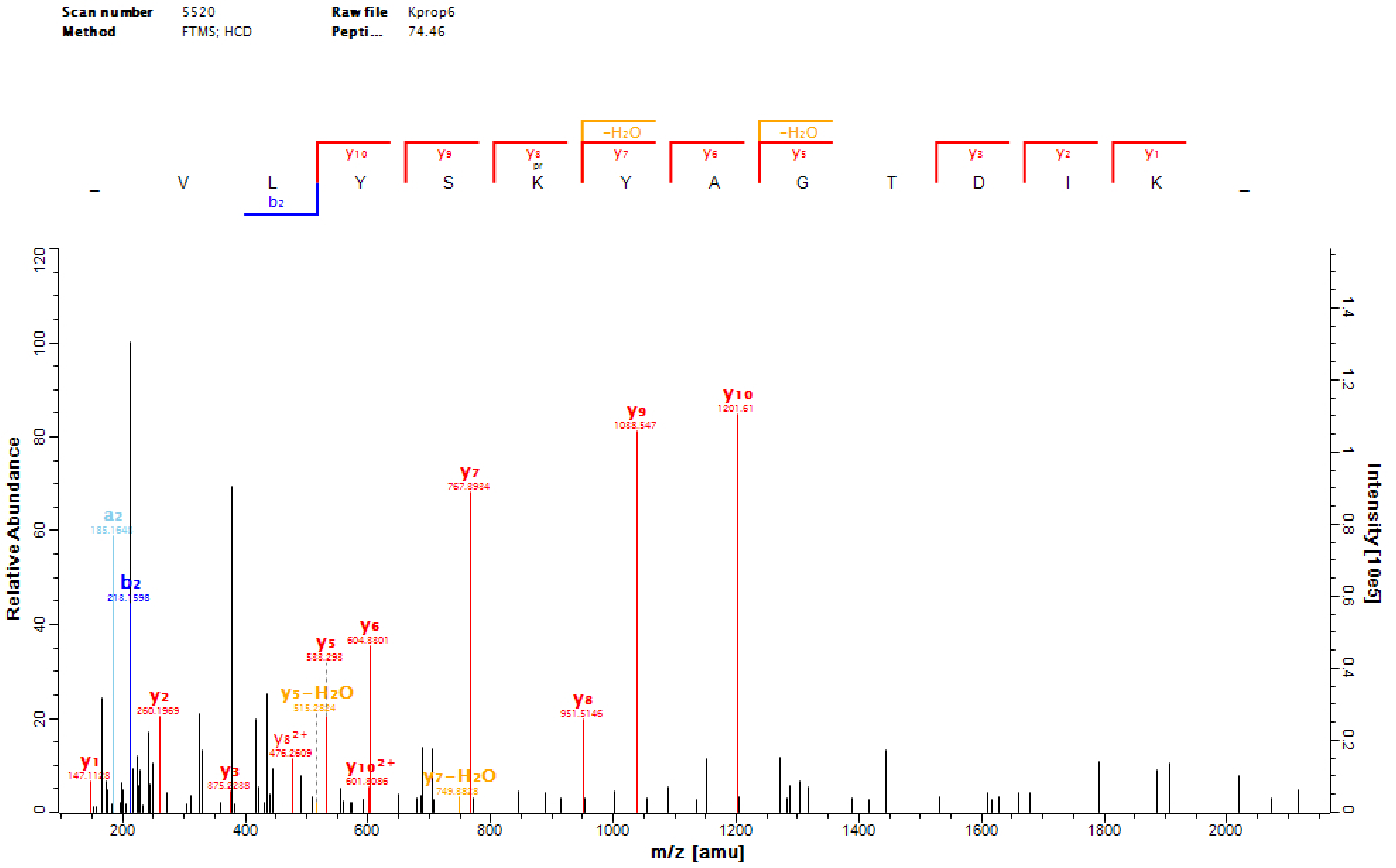
Scan number 5520
Method FTMS; HCD
Raw file Kprop6
Pepti... 74.46
_ V L Y S K Y A G T D I K _
y10 y9 y8 y7 y6 y5 y3 y2 y1
-H2O -H2O
b2
a2 185.1648
b2 213.1598
y1 147.1128
y2 260.1969
y3 375.2238
y8 2+ 476.2609
y5-H2O 515.2824
y5 533.293
y10 2+ 601.3086
y6 604.3301
y7-H2O 749.3828
y7 767.3984
y8 951.5146
y9 1038.547
y10 1201.61
Relative Abundance
Intensity [10e5]
m/z [amu]

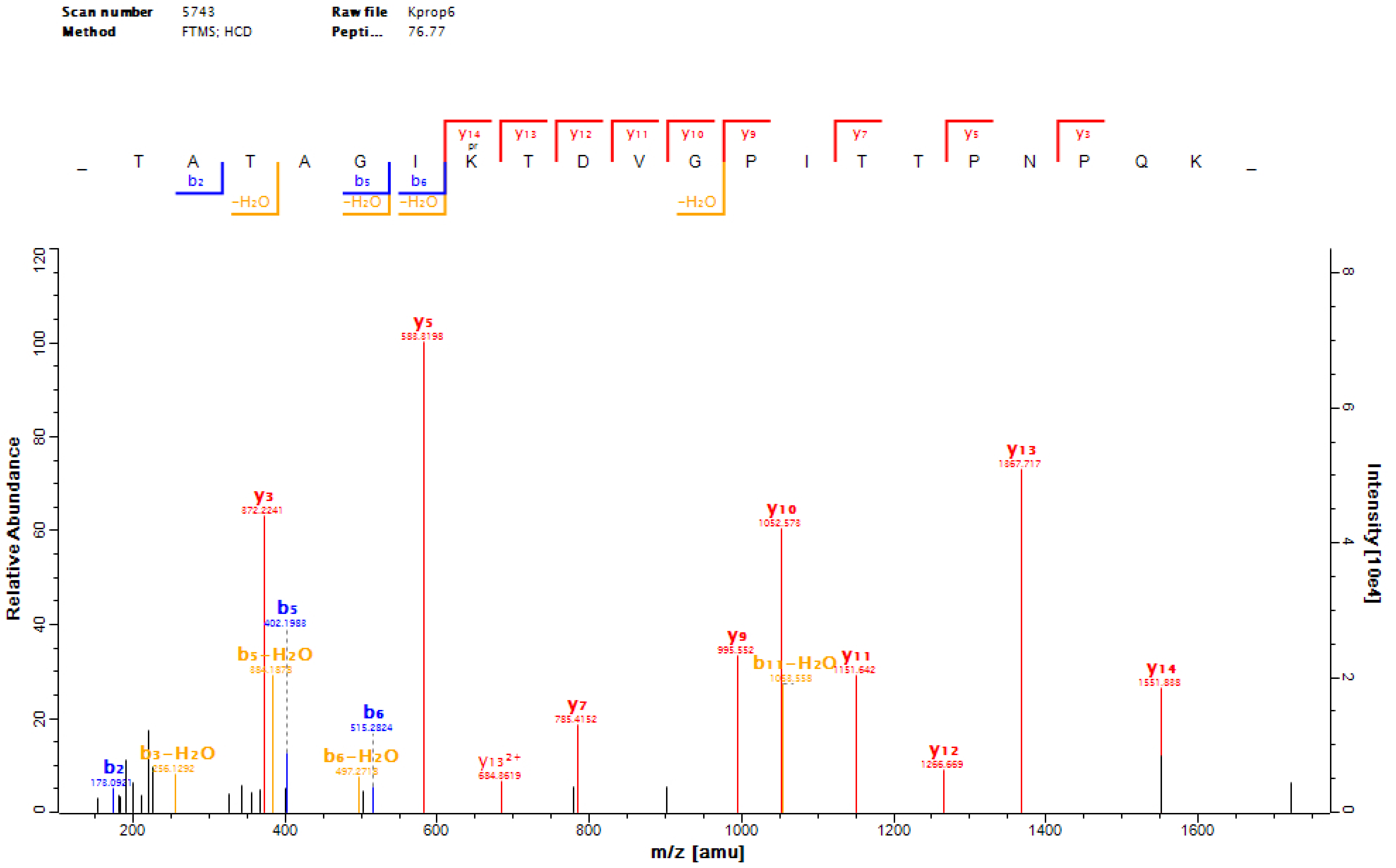

Scan number 5743
Raw file Kprop6
Method FTMS; HCD
Pepti... 76.77
_ T A T A G I K T D V G P I T T P N P Q K _
y14 y13 y12 y11 y10 y9 y7 y5 y3
b2 b5 b6
-H2O
y5
y3
y13
y10
b5
b5-H2O
y9
b11-H2O
y11
y14
b6
y7
b3-H2O
b6-H2O
b2
y13 2+
y12
Relative Abundance
Intensity [10e4]
m/z [amu]

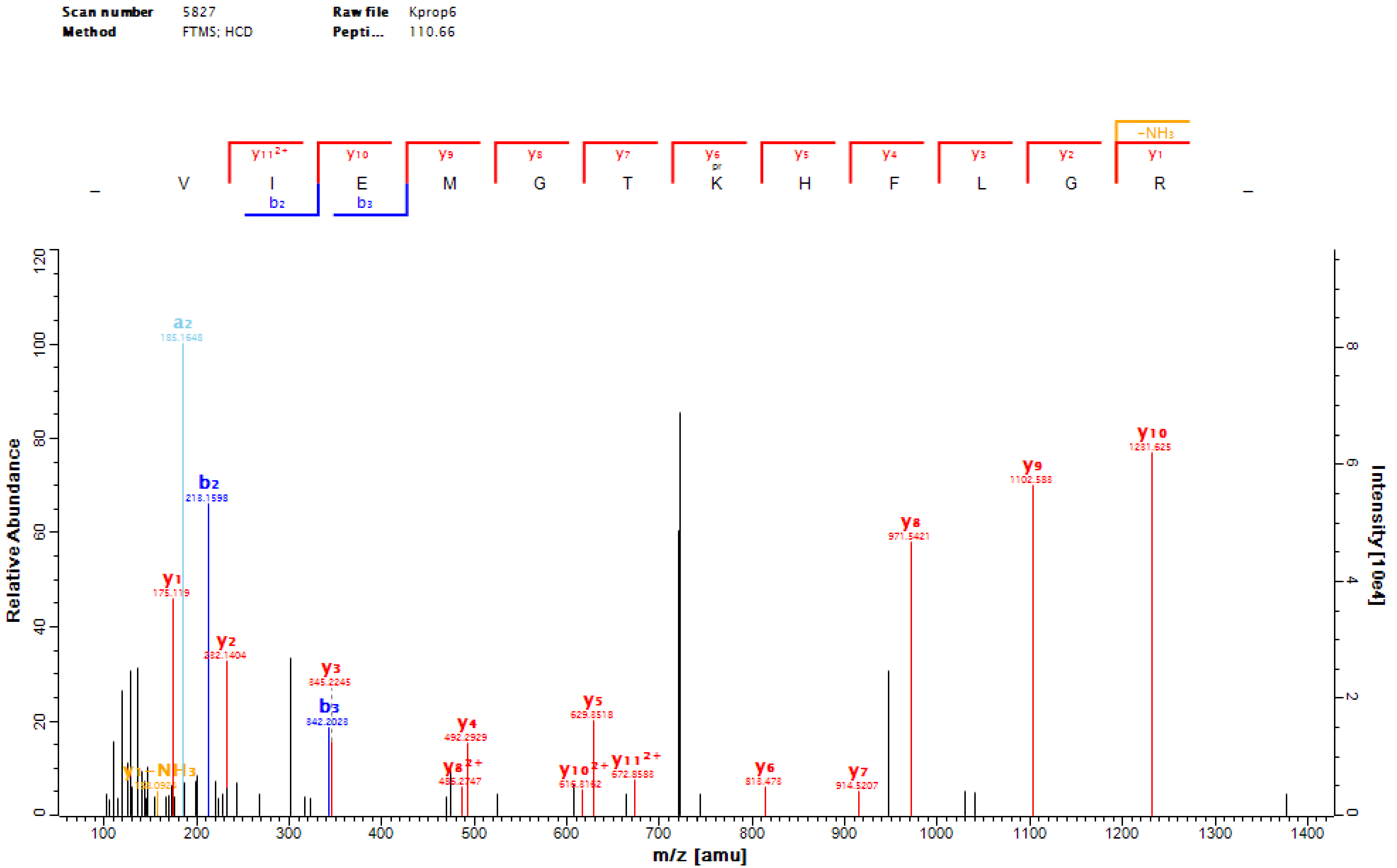

Scan number 5827
Method FTMS; HCD
Raw file Kprop6
Pepti... 110.66
_ V I E M G T K H F L G R _
pr
-NH3
a2 185.1648
b2 218.1598
b3 342.2028
y1 175.119
y1-NH3 158.0924
y2 232.1404
y3 345.2245
y4 492.2929
y8 2+ 486.2747
y5 629.3518
y10 2+ 616.3162
y11 2+ 672.8583
y6 813.473
y7 914.5207
y8 971.5421
y9 1102.583
y10 1231.625
Relative Abundance
Intensity [10e4]
m/z [amu]

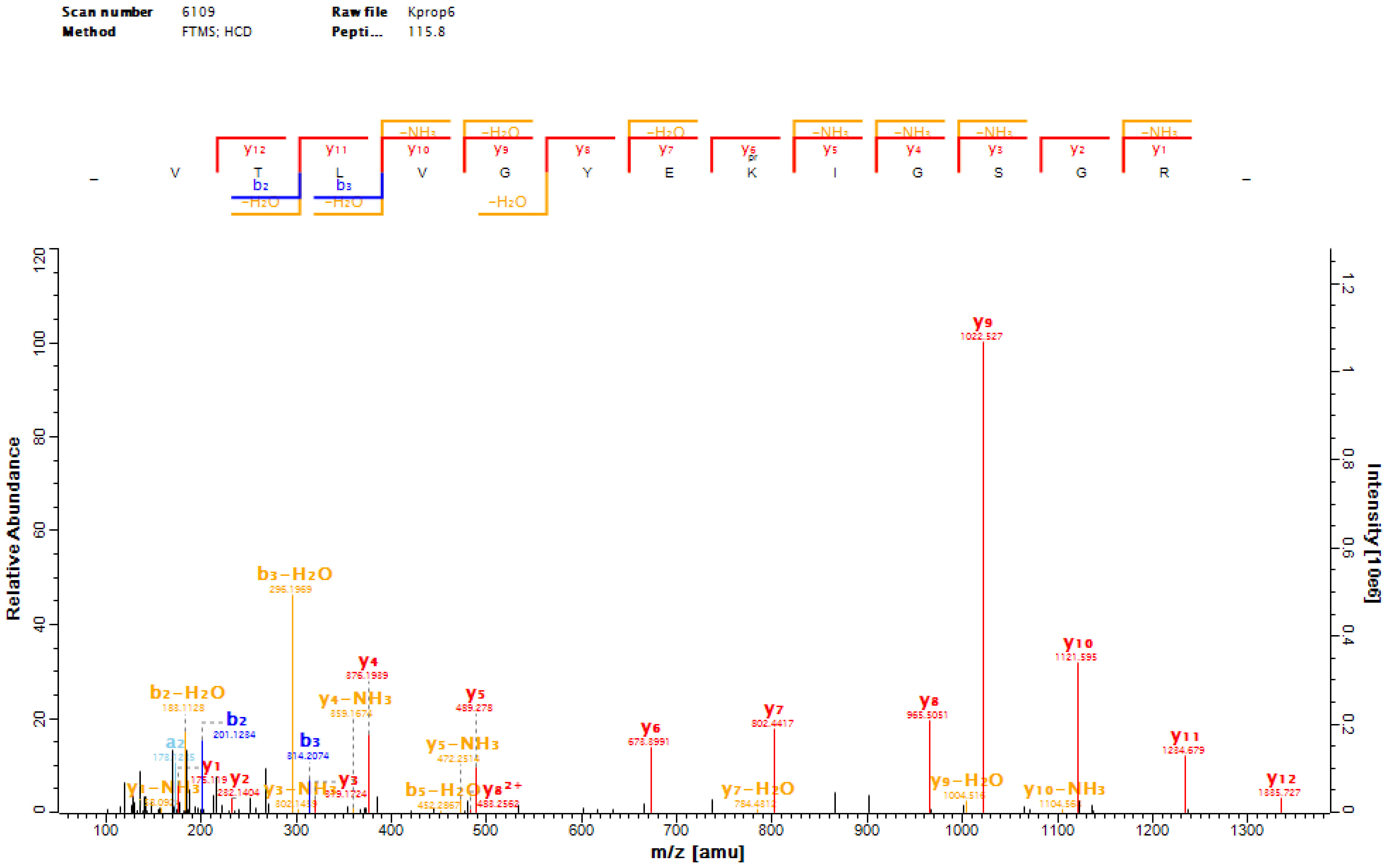

Scan number 6109
Raw file Kprop6
Method FTMS; HCD
Pepti... 115.8
V T L V G Y E K I G S G R
Relative Abundance
Intensity [10e6]
m/z [amu]
y9 1022.527
y10 1121.595
b3−H2O 296.1969
y4 376.1989
y4−NH3 359.1674
y5 489.278
y5−NH3 472.2514
y6 673.3991
y7 802.4417
y8 965.5051
y11 1234.679
y12 1335.727
b2−H2O 183.1128
b2 201.1234
b3 314.2074
y1−NH3
y1
y2 232.1404
y3−NH3
y3
b5−H2O 452.2867
y8 2+ 483.2562
y7−H2O 784.4312
y9−H2O 1004.516
y10−NH3 1104.568

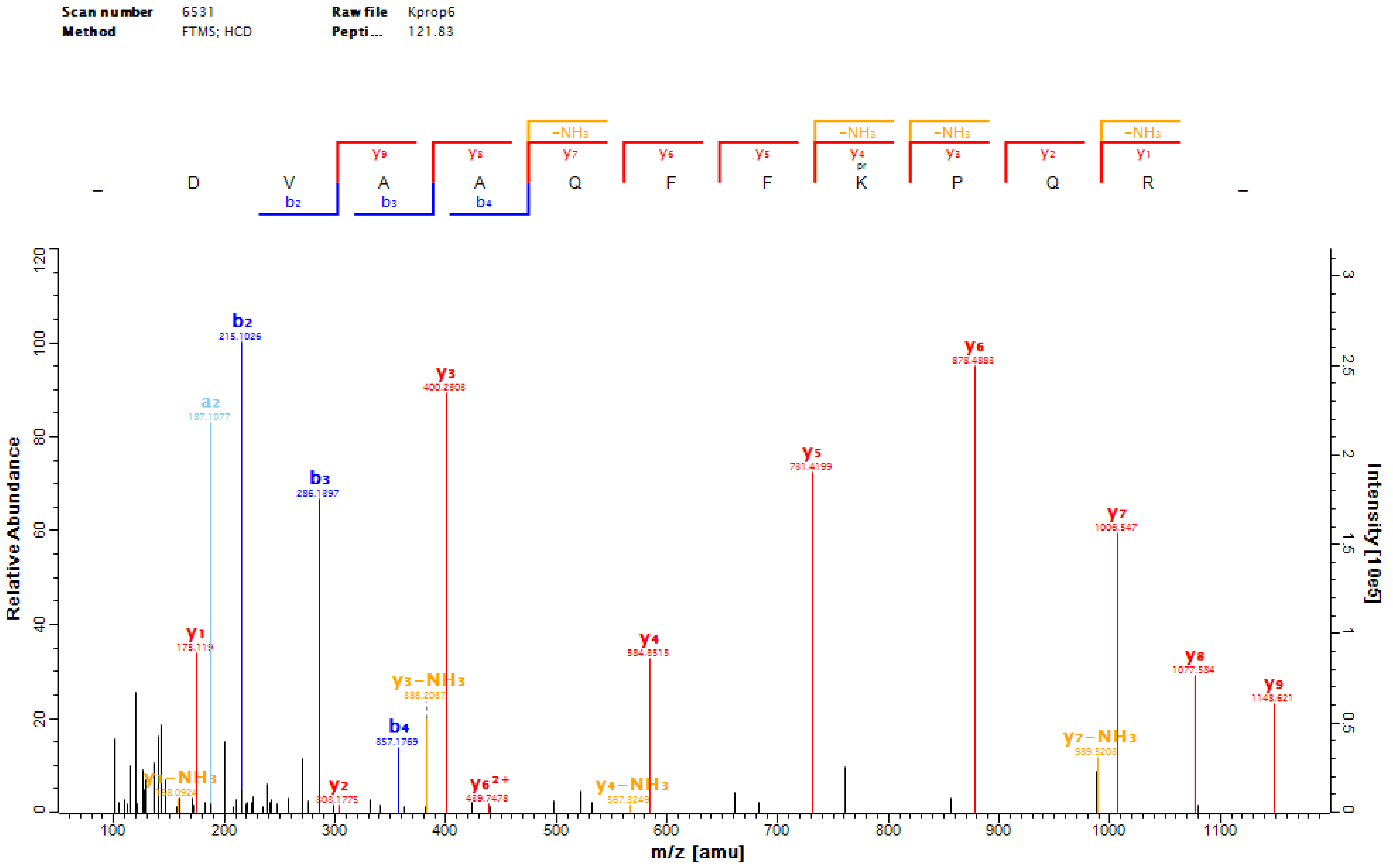

Scan number 6531
Method FTMS; HCD
Raw file Kprop6
Pepti... 121.83
_ D V A A Q F F K P Q R _
pr
-NH3
y9 y8 y7 y6 y5 y4 y3 y2 y1
b2 b3 b4
b2 215.1026
a2 187.1077
b3 286.1397
b4 357.1769
y1 175.119
y2
y3 400.2303
y3-NH3
y4 584.3515
y4-NH3
y5 731.4199
y6 878.4883
y6 2+
y7 1005.547
y7-NH3
y8 1077.584
y9 1148.621
Relative Abundance
Intensity [10e5]
m/z [amu]

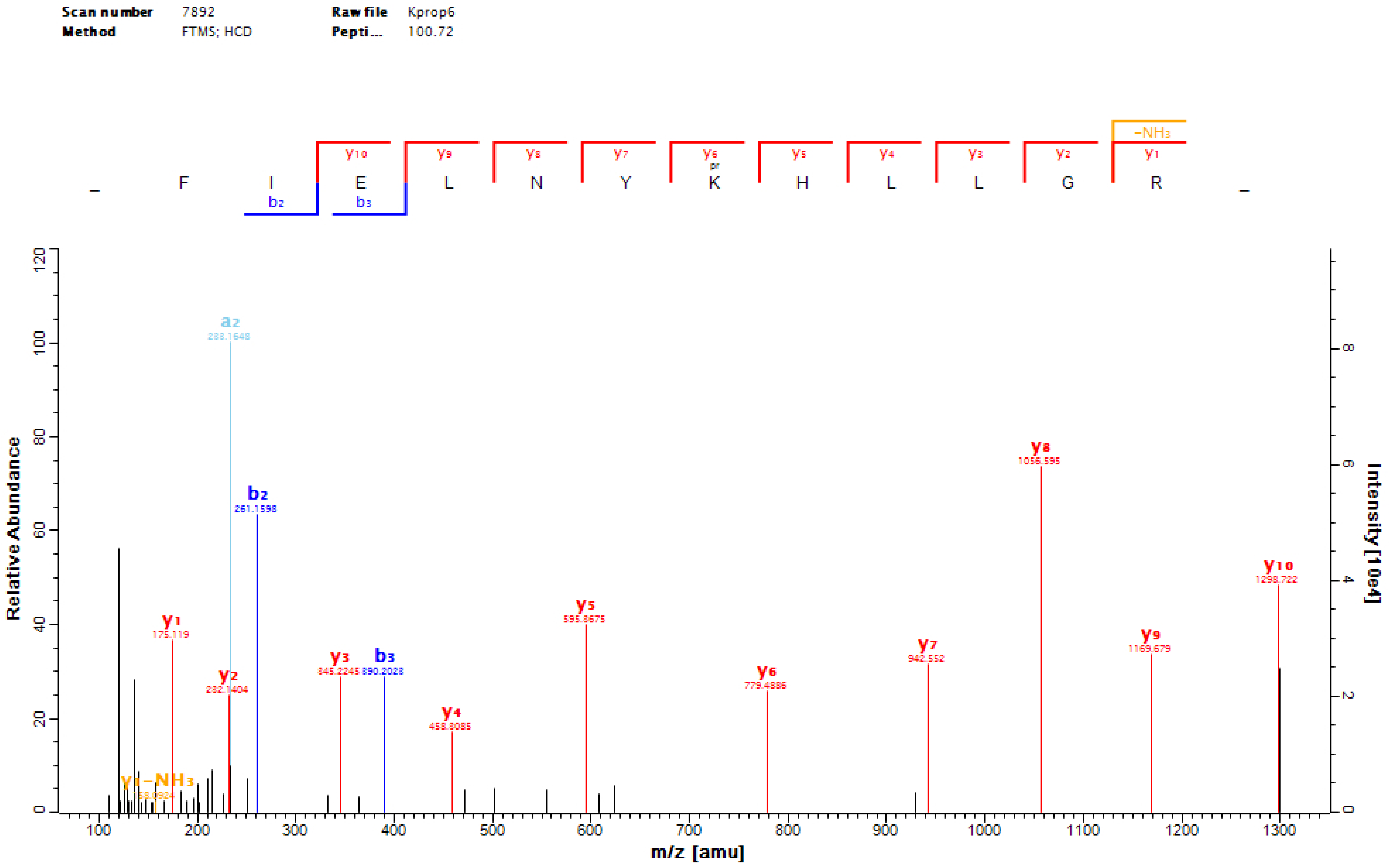

Scan number 7892
Method FTMS; HCD
Raw file Kprop6
Pepti... 100.72
_ F I E L N Y K H L L G R _
b2 b3
y10 y9 y8 y7 y6 y5 y4 y3 y2 y1
pr
-NH3
a2 233.1648
b2 261.1598
y1 175.119
y2 232.1404
y3 345.2245
b3 390.2023
y4 458.3085
y5 595.3675
y6 779.4886
y7 942.552
y8 1056.595
y9 1169.679
y10 1298.722
y1-NH3
Relative Abundance
Intensity [10e4]
m/z [amu]

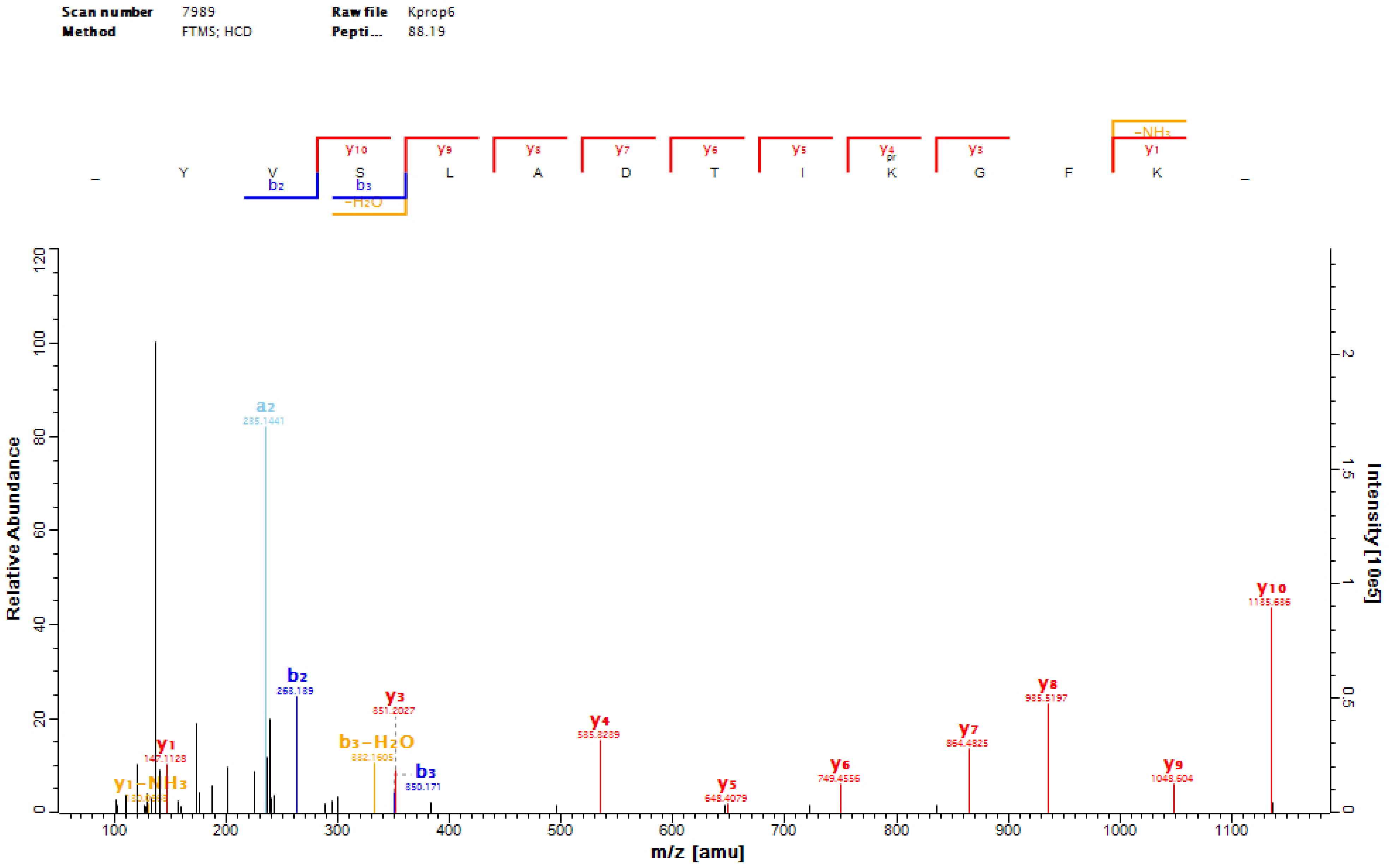
Scan number 7989
Method FTMS; HCD
Raw file Kprop6
Pepti... 88.19
Y V S L A D T I K G F K
y10 y9 y8 y7 y6 y5 y4 y3 y1
-NH3
b2 b3
-H2O
Relative Abundance
Intensity [10e5]
m/z [amu]
a2
235.1441
b2
263.139
y3
351.2027
b3-H2O
332.1605
b3
350.171
y1
147.1128
y1-NH3
y4
535.3239
y5
648.4079
y6
749.4556
y7
864.4825
y8
935.5197
y9
1048.604
y10
1135.636

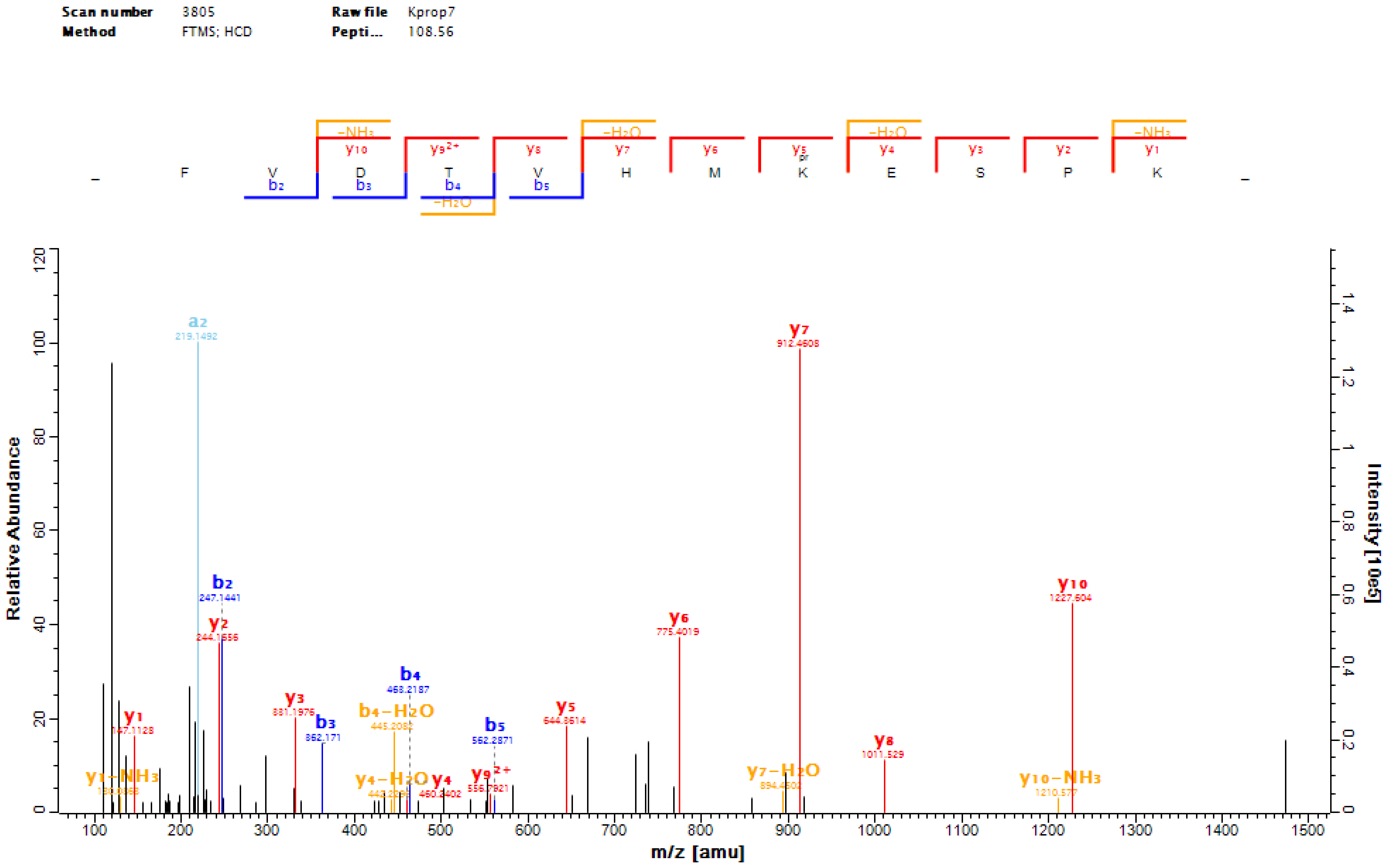

Scan number 3805
Raw file Kprop7
Method FTMS; HCD
Pepti... 108.56
_ F V D T V H M K E S P K _
a2 219.1492
b2 247.1441
y2
y1 147.1128
y3 331.1976
b3 362.171
b4 463.2187
b4−H2O 445.2082
y4−H2O
y4
y9 2+
b5 562.2871
y5 644.3614
y6 775.4019
y7 912.4608
y7−H2O 894.4502
y8 1011.529
y10 1227.604
y10−NH3 1210.577
Relative Abundance
Intensity [10e5]
m/z [amu]

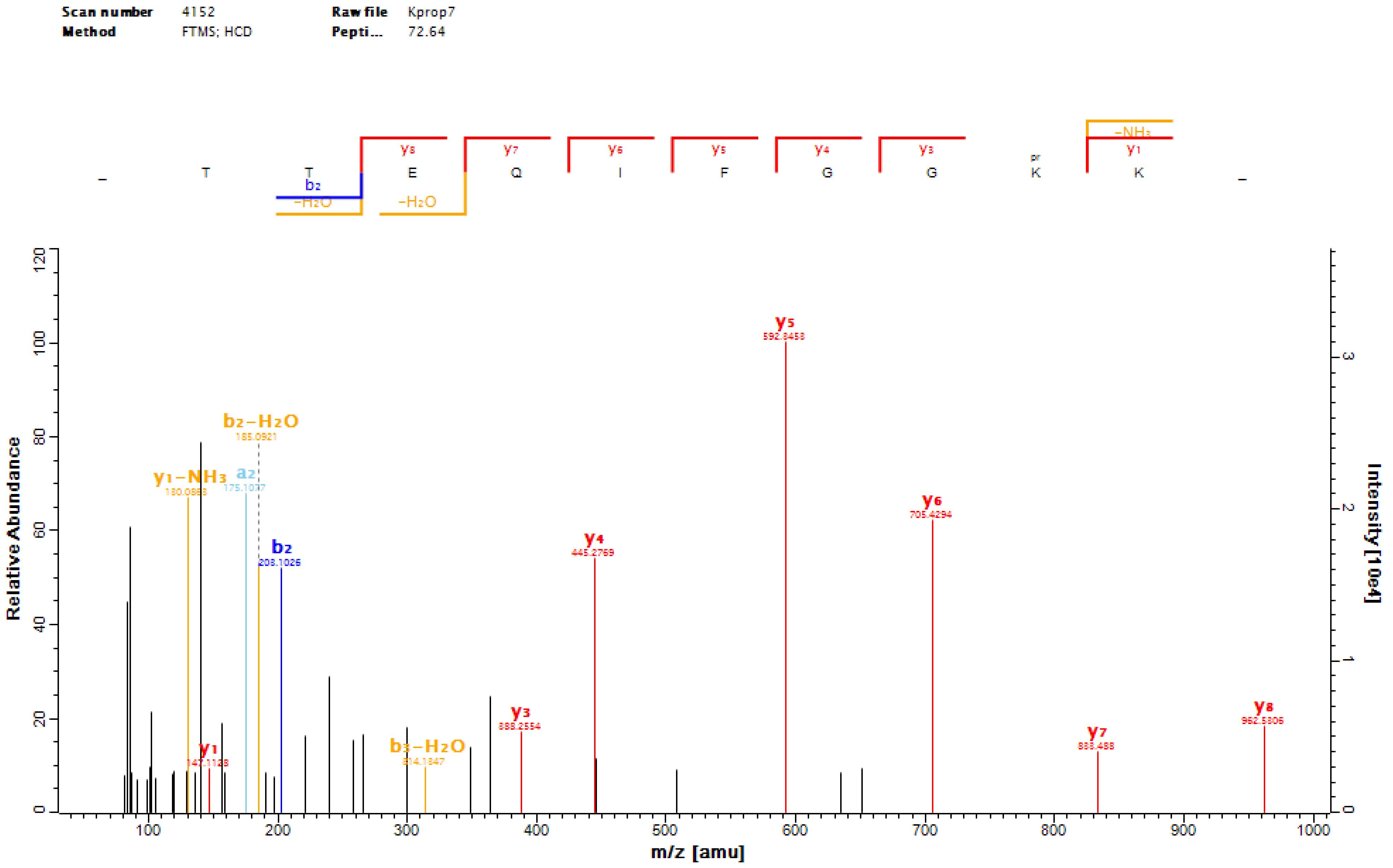
Scan number 4152
Raw file Kprop7
Method FTMS; HCD
Pepti... 72.64
T T E Q I F G G K K
pr
b2
-H2O
-H2O
y8 y7 y6 y5 y4 y3 y1
-NH3
y5 592.3453
y6 705.4294
y4 445.2769
b2-H2O 185.0921
y1-NH3 130.0863
a2 175.1077
b2 203.1026
y3 388.2554
y8 962.5306
y7 833.488
y1 147.1128
b3-H2O 314.1847
Relative Abundance
Intensity [10e4]
m/z [amu]

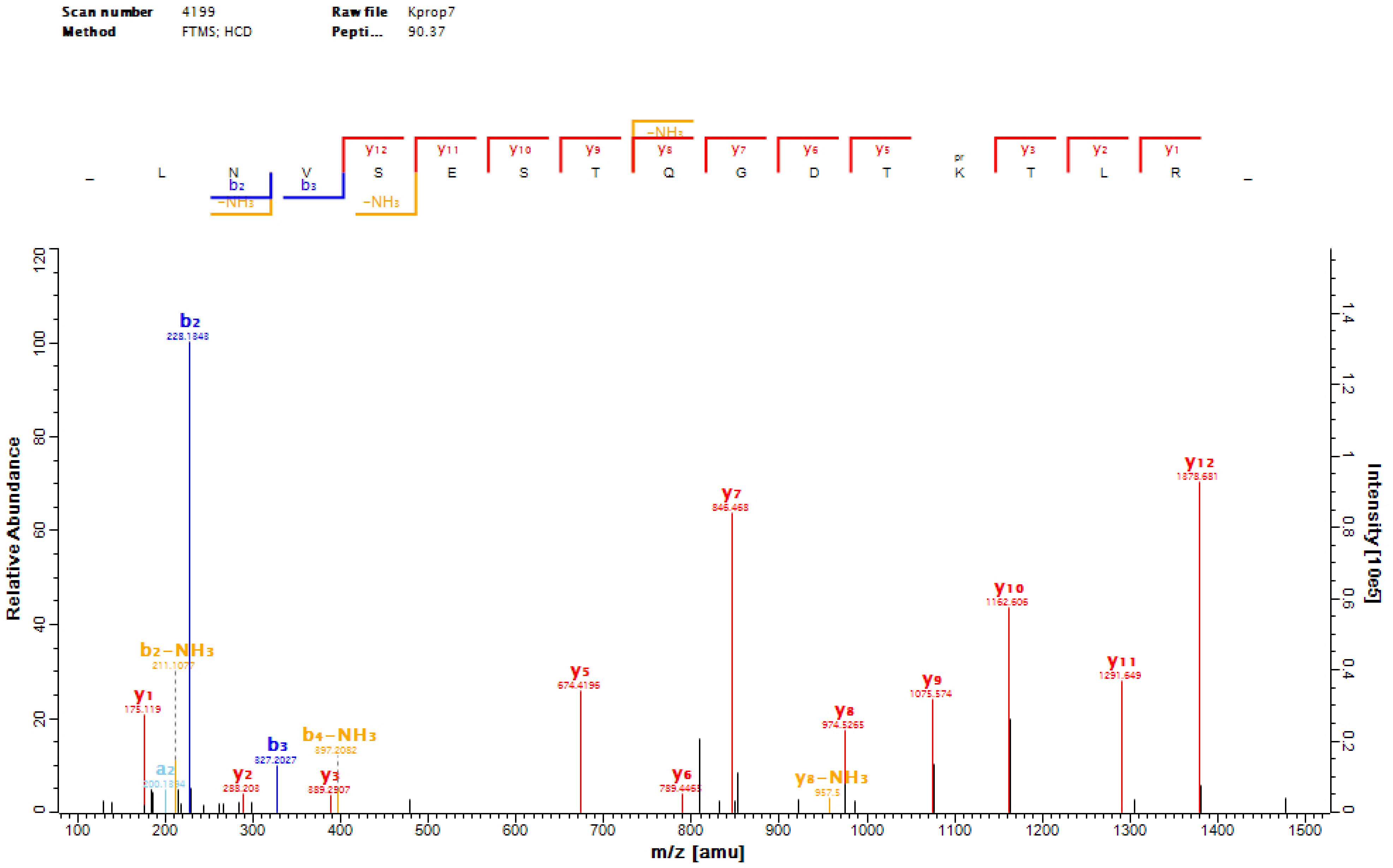

Scan number 4199
Method FTMS; HCD
Raw file Kprop7
Pepti... 90.37
_ L N V S E S T Q G D T K T L R _
pr
b2
b3
-NH3
y12 y11 y10 y9 y8 y7 y6 y5 y3 y2 y1
Relative Abundance
Intensity [10e5]
m/z [amu]
b2 228.1848
b2-NH3 211.1077
y1 175.119
a2 200.1894
y2 288.203
b3 327.2027
b4-NH3 397.2062
y3 389.2507
y5 674.4196
y6 789.4463
y7 846.468
y8-NH3 957.5
y8 974.5265
y9 1075.574
y10 1162.606
y11 1291.649
y12 1378.681

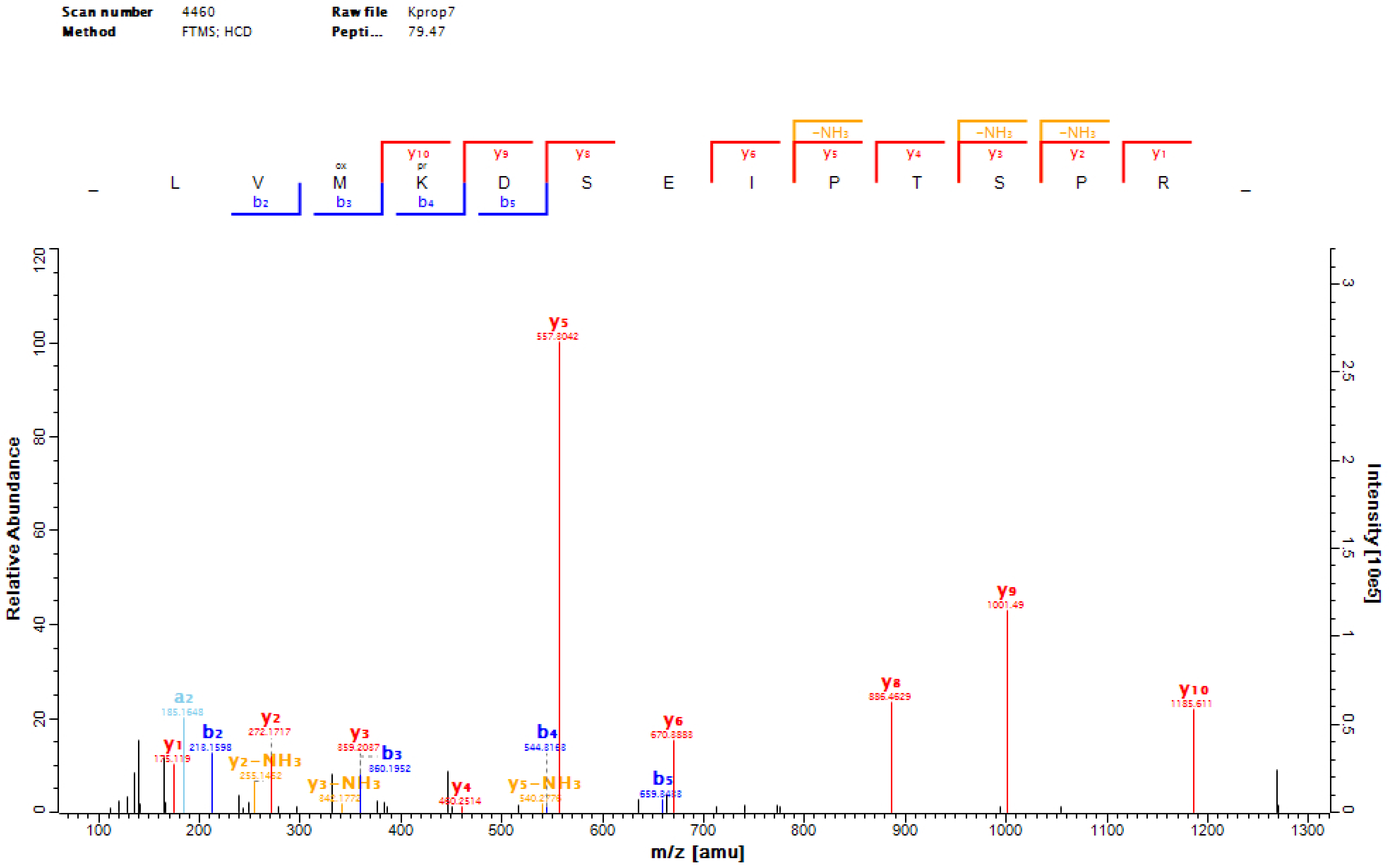
Scan number 4460
Raw file Kprop7
Method FTMS; HCD
Pepti... 79.47
_ L V M K D S E I P T S P R _
ox
pr
b2 b3 b4 b5
y10 y9 y8 y6 y5 y4 y3 y2 y1
-NH3
y5 557.3042
y9 1001.49
y8 886.4629
y10 1185.611
y6 670.3883
a2 185.1648
b2 213.1598
y1 175.119
y2 272.1717
y2-NH3 255.1452
y3 359.2037
b3 360.1952
y3-NH3 342.1772
y4 460.2514
b4 544.3163
y5-NH3 540.2776
b5 659.3433
Relative Abundance
Intensity [10e5]
m/z [amu]

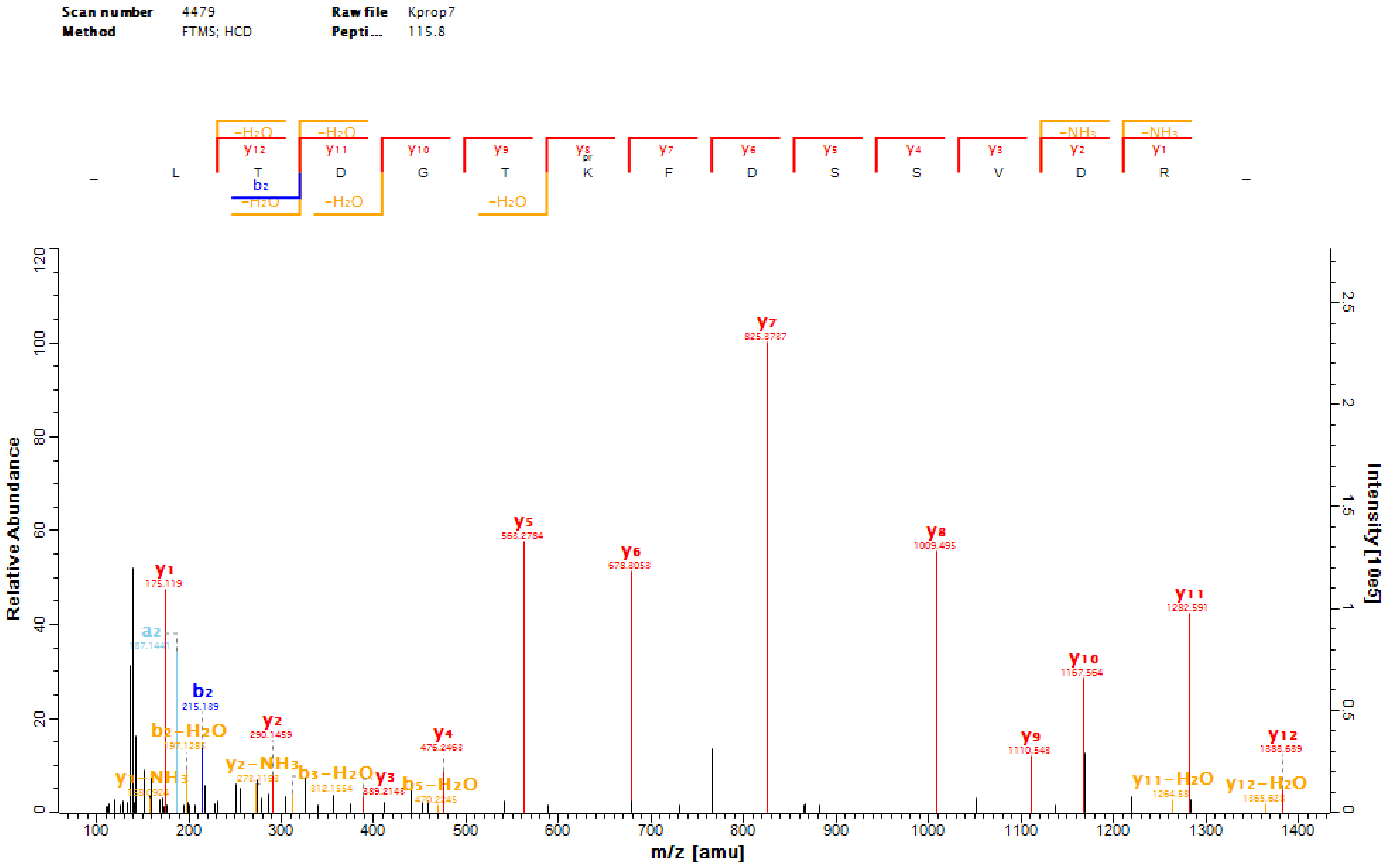
Scan number 4479
Raw file Kprop7
Method FTMS; HCD
Pepti... 115.8
_ L T D G T K F D S S V D R _
y12 y11 y10 y9 y8 y7 y6 y5 y4 y3 y2 y1
b2
-H2O
-NH3
Relative Abundance
Intensity [10e5]
m/z [amu]
y1 175.119
a2 187.1441
b2 215.139
b2-H2O 197.1285
y1-NH3
y2 290.1459
y2-NH3 273.1193
b3-H2O 312.1554
y3 389.2143
b5-H2O 470.2245
y4 476.2463
y5 563.2784
y6 678.3053
y7 825.3737
y8 1009.495
y9 1110.543
y10 1167.564
y11 1282.591
y11-H2O 1264.58
y12 1383.639
y12-H2O 1365.628

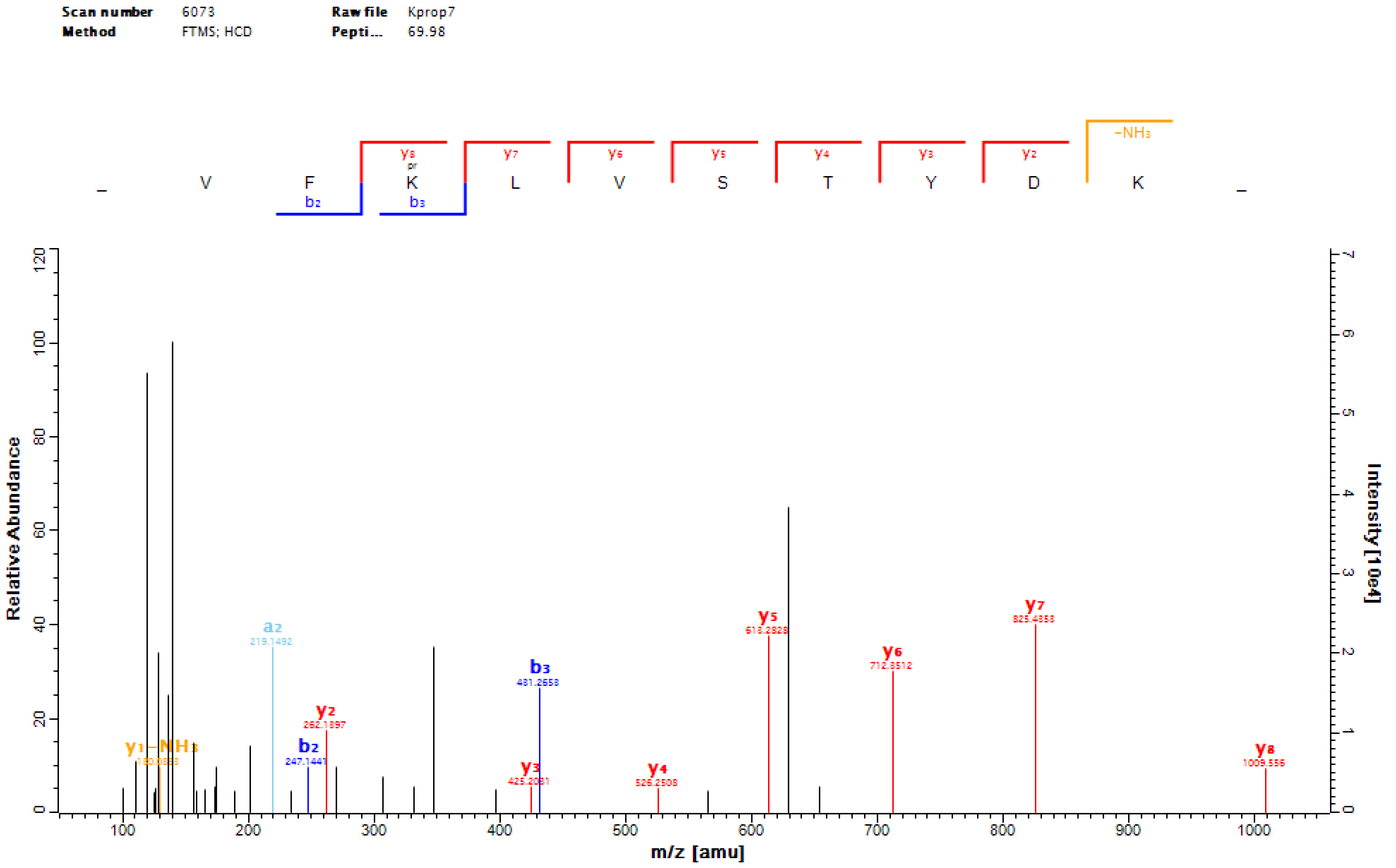

Scan number 6073
Method FTMS; HCD
Raw file Kprop7
Pepti... 69.98
_ V F K L V S T Y D K _
y8 y7 y6 y5 y4 y3 y2 -NH3
b2 b3
a2 219.1492
y2 262.1897
b2 247.1441
b3 431.2653
y3 425.2081
y4 526.2508
y5 613.2828
y6 712.3512
y7 825.4353
y8 1009.556
y1-NH3
Relative Abundance
Intensity [10e4]
m/z [amu]

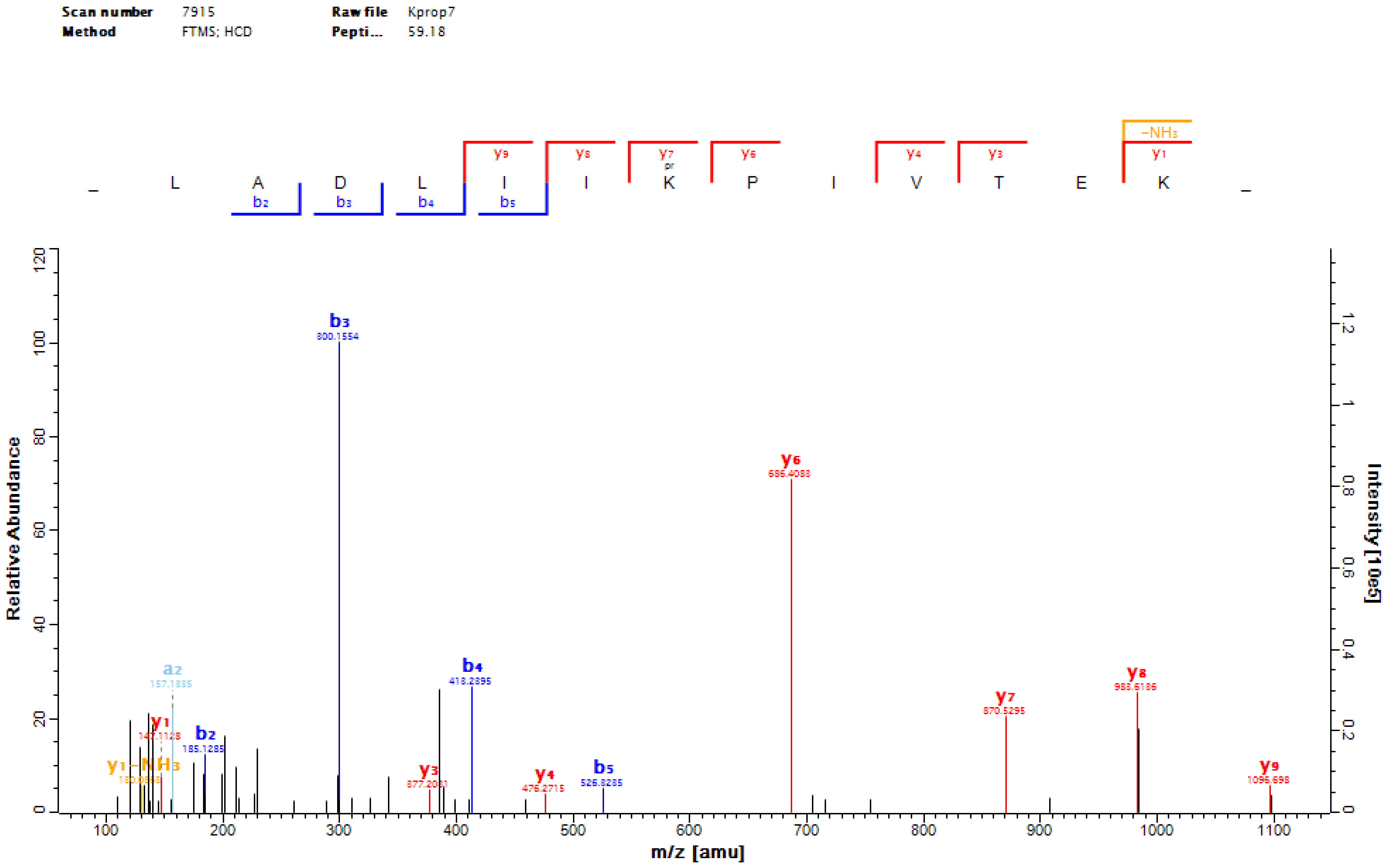

Scan number 7915
Raw file Kprop7
Method FTMS; HCD
Pepti... 59.18
_ L A D L I I K P I V T E K _
b2 b3 b4 b5
y9 y8 y7 y6 y4 y3 y1
-NH3
b3 300.1554
y6 686.4083
a2 157.1335
b4 413.2395
y7 870.5295
y8 983.6136
y1
b2 185.1285
y1-NH3
y3
y4 476.2715
b5 526.3235
y9 1096.698
Relative Abundance
Intensity [10e5]
m/z [amu]

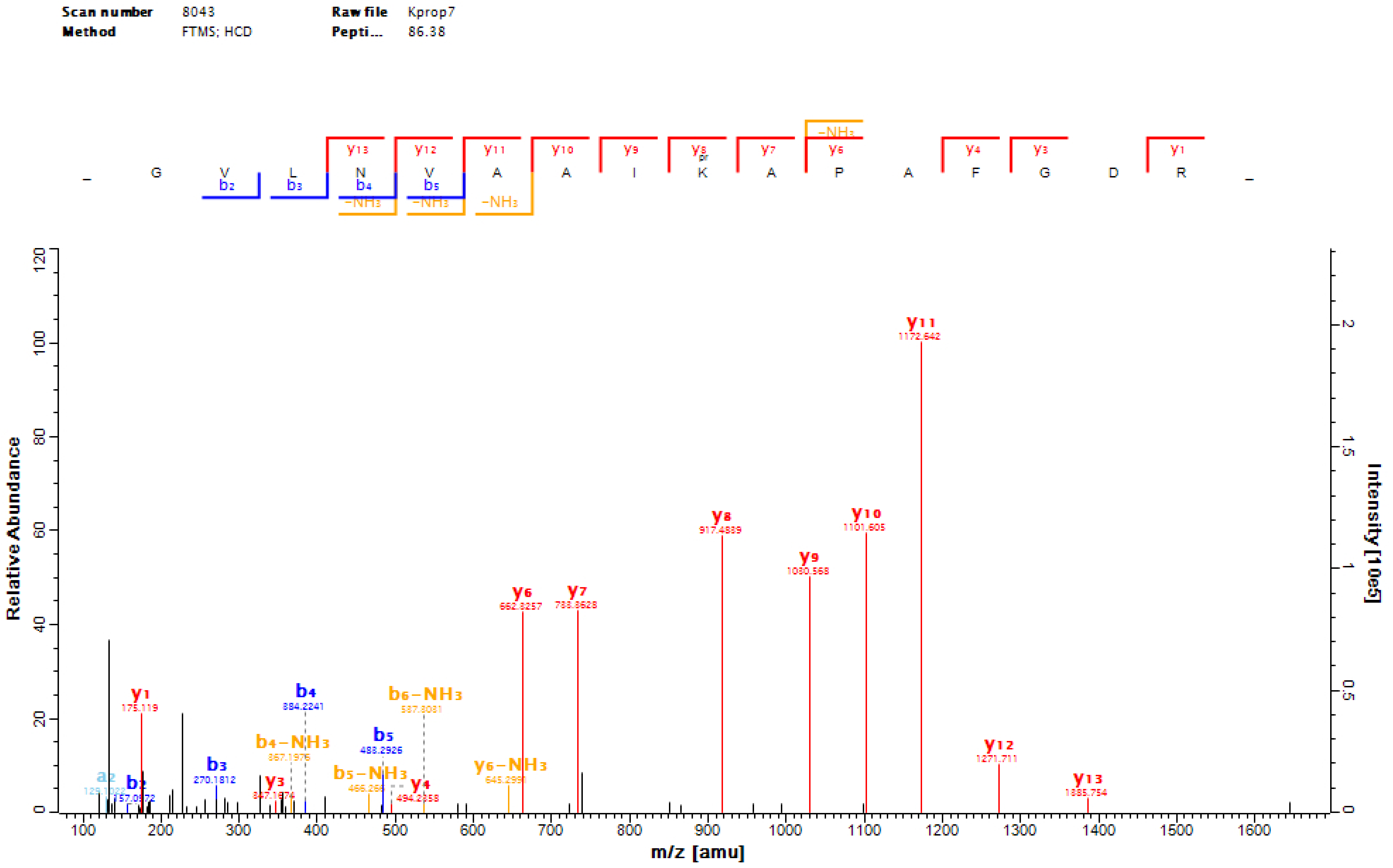

Scan number 8043
Method FTMS; HCD
Raw file Kprop7
Pepti... 86.38
G V L N V A A I K A P A F G D R
Relative Abundance
Intensity [10e5]
m/z [amu]
y1 175.119
b3 270.1812
b4 384.2241
b4−NH3
b5 483.2926
b6−NH3
y6−NH3
y6 662.3257
y7
y8 917.4889
y9
y10 1101.605
y11 1172.642
y12 1271.711
y13

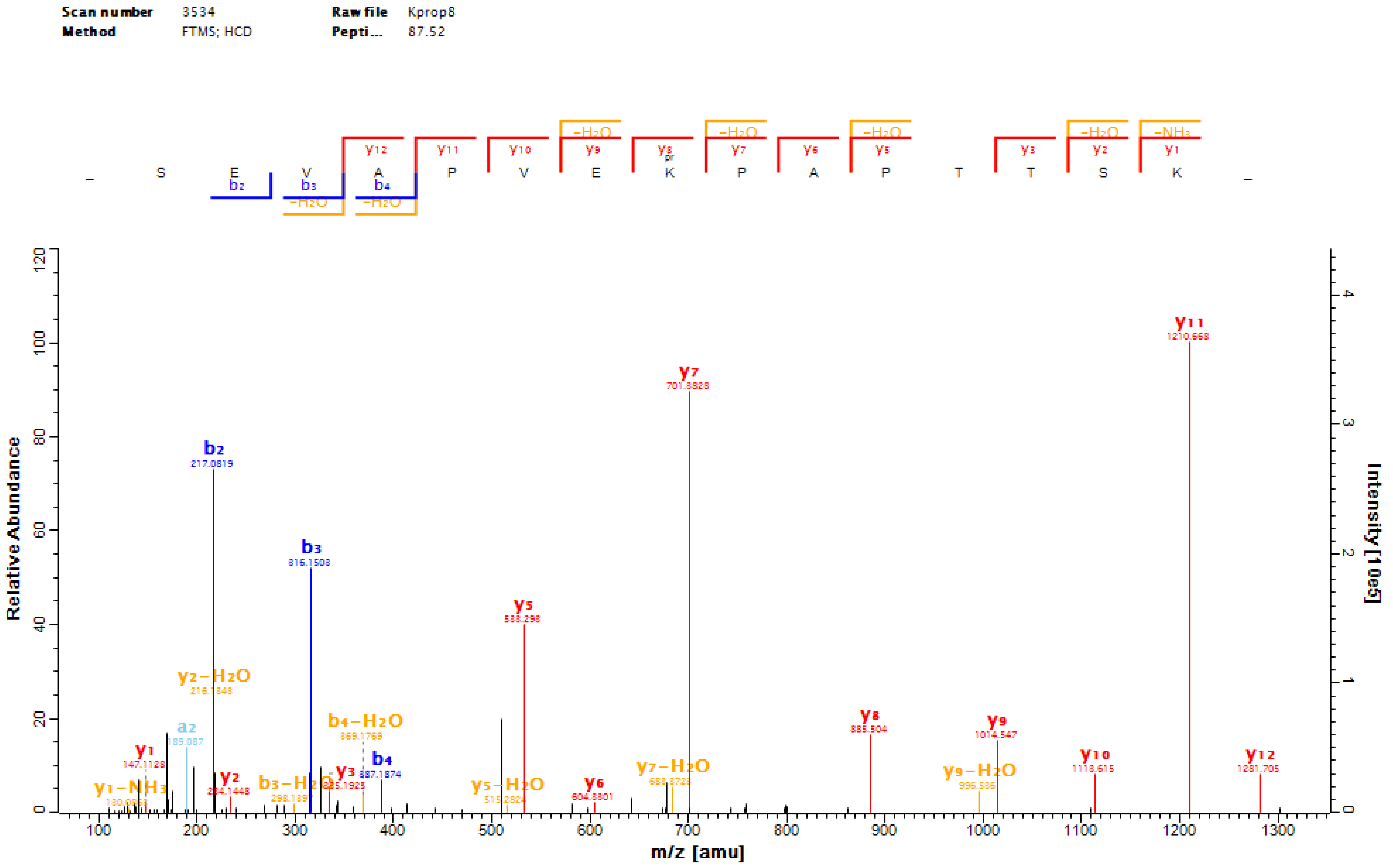
Scan number 3534
Raw file Kprop8
Method FTMS; HCD
Pepti... 87.52
_ S E V A P V E K P A P T T S K _
Relative Abundance
Intensity [10e5]
m/z [amu]
b2 217.0819
b3 316.1508
b4 387.1874
a2 189.087
y1 147.1128
y2 234.1448
y3 335.1925
y5 533.293
y6 604.3301
y7 701.3828
y8 885.504
y9 1014.547
y10 1113.615
y11 1210.668
y12 1281.705
y1−NH3 130.0863
y2−H2O 216.1343
b3−H2O 298.1397
b4−H2O 369.1769
y5−H2O 515.2824
y7−H2O 683.3723
y9−H2O 996.536

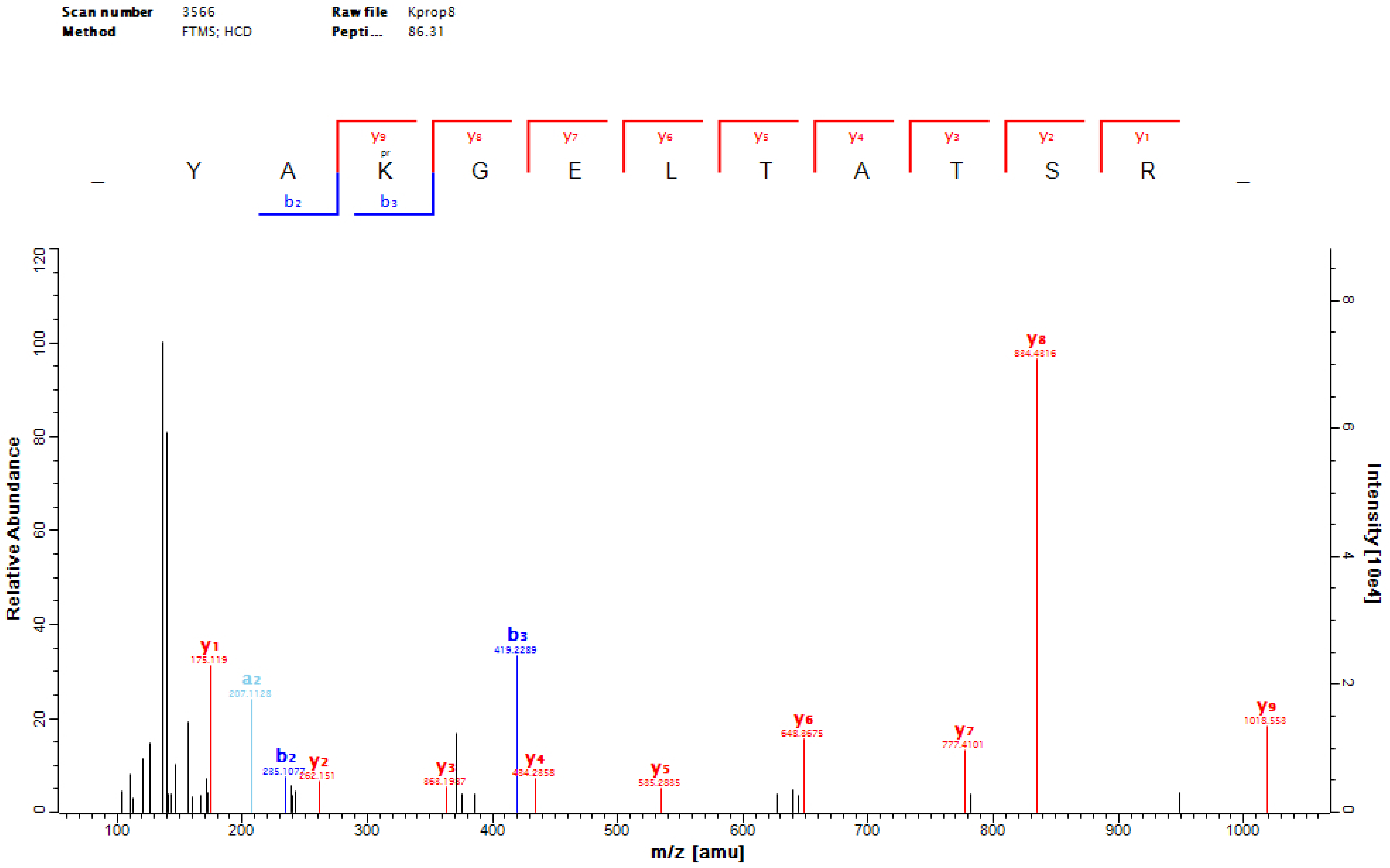

Scan number 3566
Method FTMS; HCD
Raw file Kprop8
Pepti... 86.31
_ Y A K G E L T A T S R _
pr
y9 y8 y7 y6 y5 y4 y3 y2 y1
b2 b3
y1 175.119
a2 207.1128
b2 235.1072
y2 262.151
y3 363.1987
b3 419.2289
y4 434.2858
y5 535.2835
y6 648.3675
y7 777.4101
y8 834.4316
y9 1018.553
Relative Abundance
Intensity [10e4]
m/z [amu]

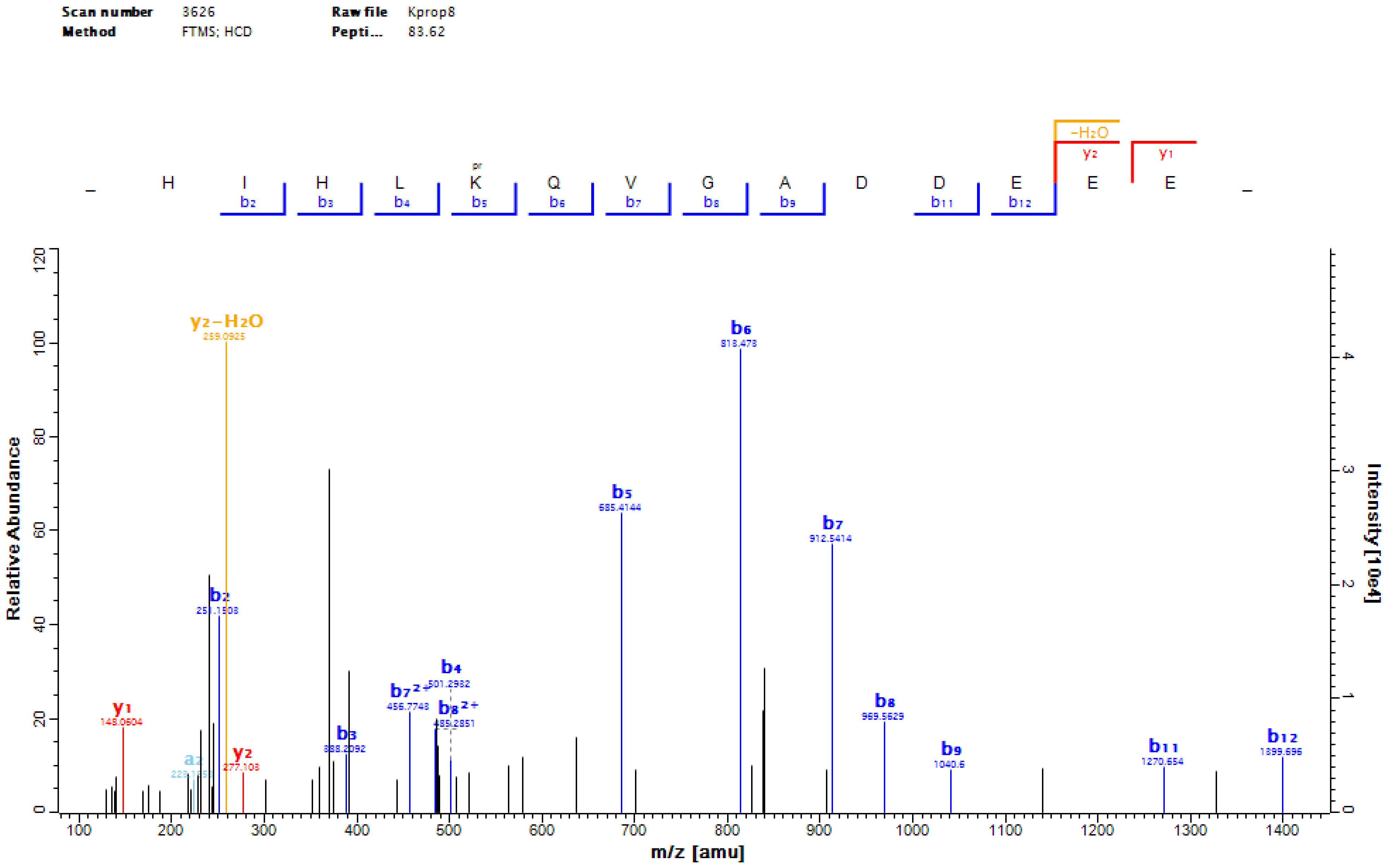

Scan number 3626
Method FTMS; HCD
Raw file Kprop8
Pepti... 83.62
_ H I H L K Q V G A D D E E E _
pr
b2 b3 b4 b5 b6 b7 b8 b9 b11 b12
-H2O
y2
y1
y1 148.0604
a2
y2-H2O 259.0925
b2
y2 277.108
b3 388.2092
b7 2+ 456.7748
b4 501.2982
b8 2+ 485.2851
b5 685.4144
b6 818.473
b7 912.5414
b8 969.5629
b9 1040.6
b11 1270.654
b12 1399.696
Relative Abundance
Intensity [10e4]
m/z [amu]

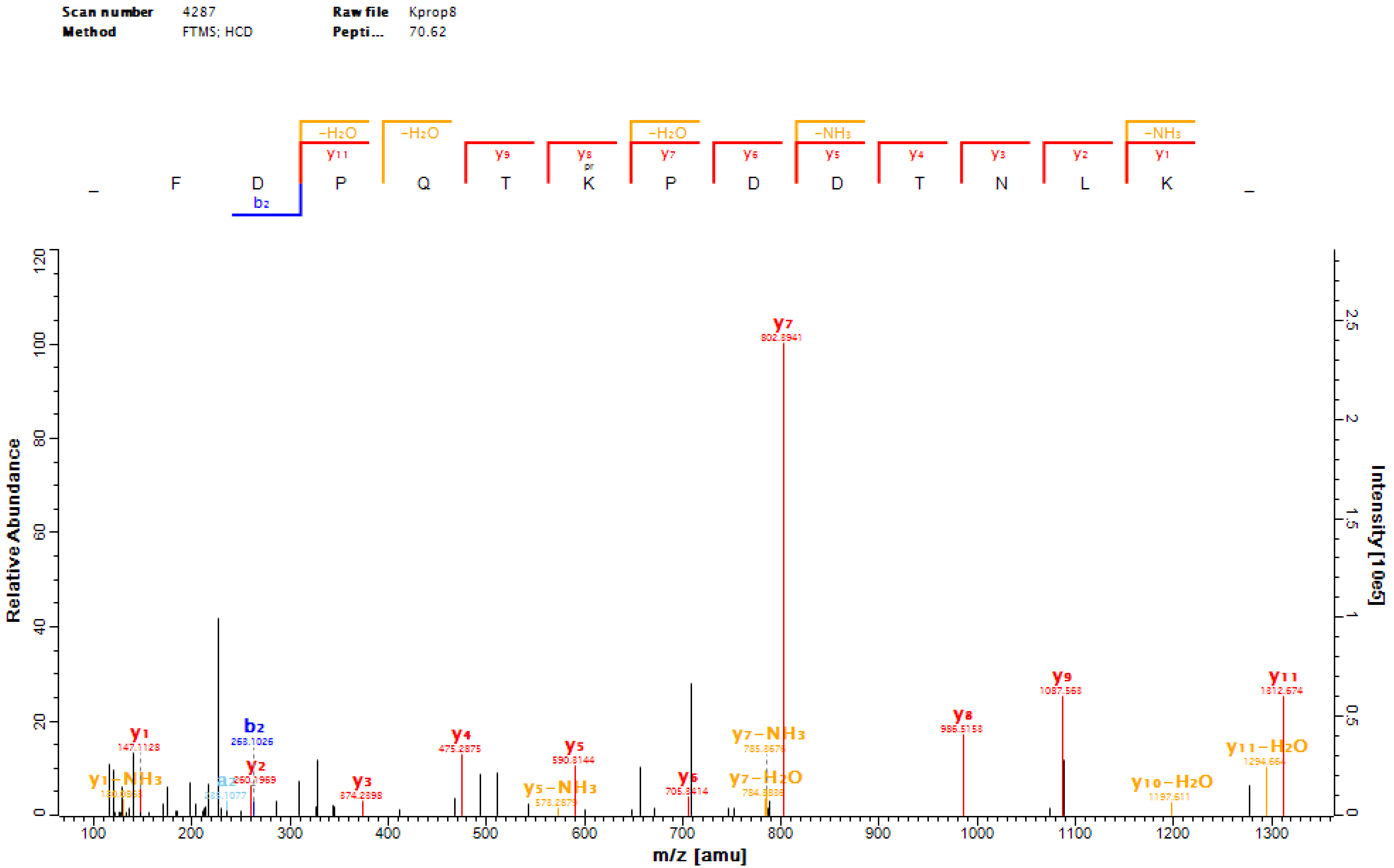

Scan number 4287
Raw file Kprop8
Method FTMS; HCD
Pepti... 70.62
_ F D P Q T K P D D T N L K _
b2
y11 y9 y8 y7 y6 y5 y4 y3 y2 y1
-H2O -H2O -H2O -NH3 -NH3
Relative Abundance
Intensity [10e5]
m/z [amu]
y1 147.1128
y1-NH3
b2 263.1026
a2
y2 260.1969
y3 374.2898
y4 475.2875
y5 590.3144
y5-NH3
y6 705.3414
y7-NH3
y7-H2O
y7 802.3941
y8 986.5153
y9 1087.563
y10-H2O 1197.611
y11-H2O
y11 1312.674

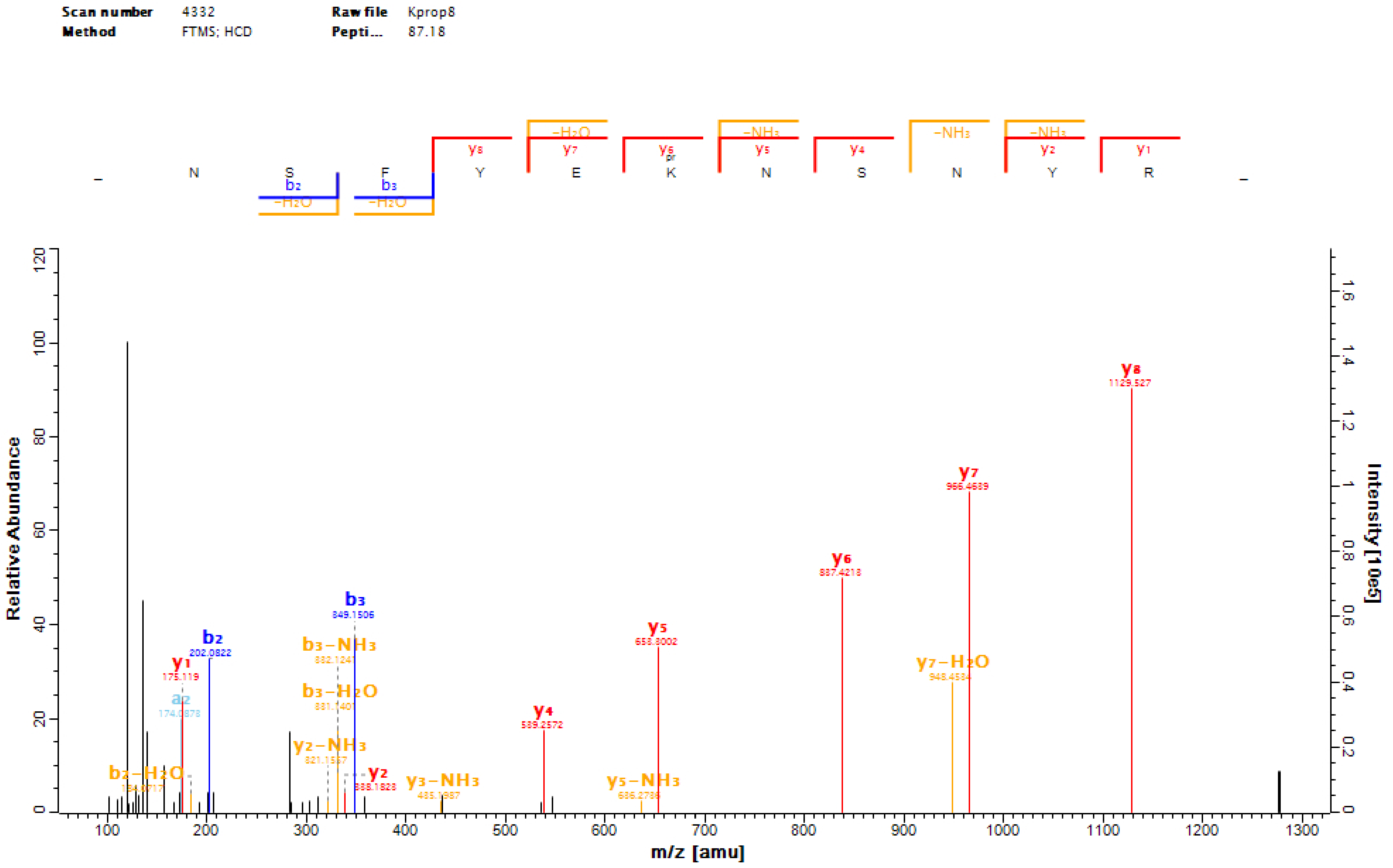

Scan number 4332
Raw file Kprop8
Method FTMS; HCD
Pepti... 87.18
_ N S F Y E K N S N Y R _
b2 b3 -H2O -H2O
y8 y7 y6 y5 y4 y2 y1
-H2O -NH3 -NH3 -NH3
y8
1129.527
y7
966.4639
y6
837.4213
y5
653.3002
y4
539.2572
y7-H2O
948.4534
y5-NH3
636.2736
y3-NH3
435.1987
b3
349.1506
b3-NH3
332.1241
b3-H2O
331.1401
y2-NH3
321.1557
y2
338.1823
b2
202.0822
y1
175.119
a2
174.0873
b2-H2O
184.0717
Relative Abundance
Intensity [10e5]
m/z [amu]

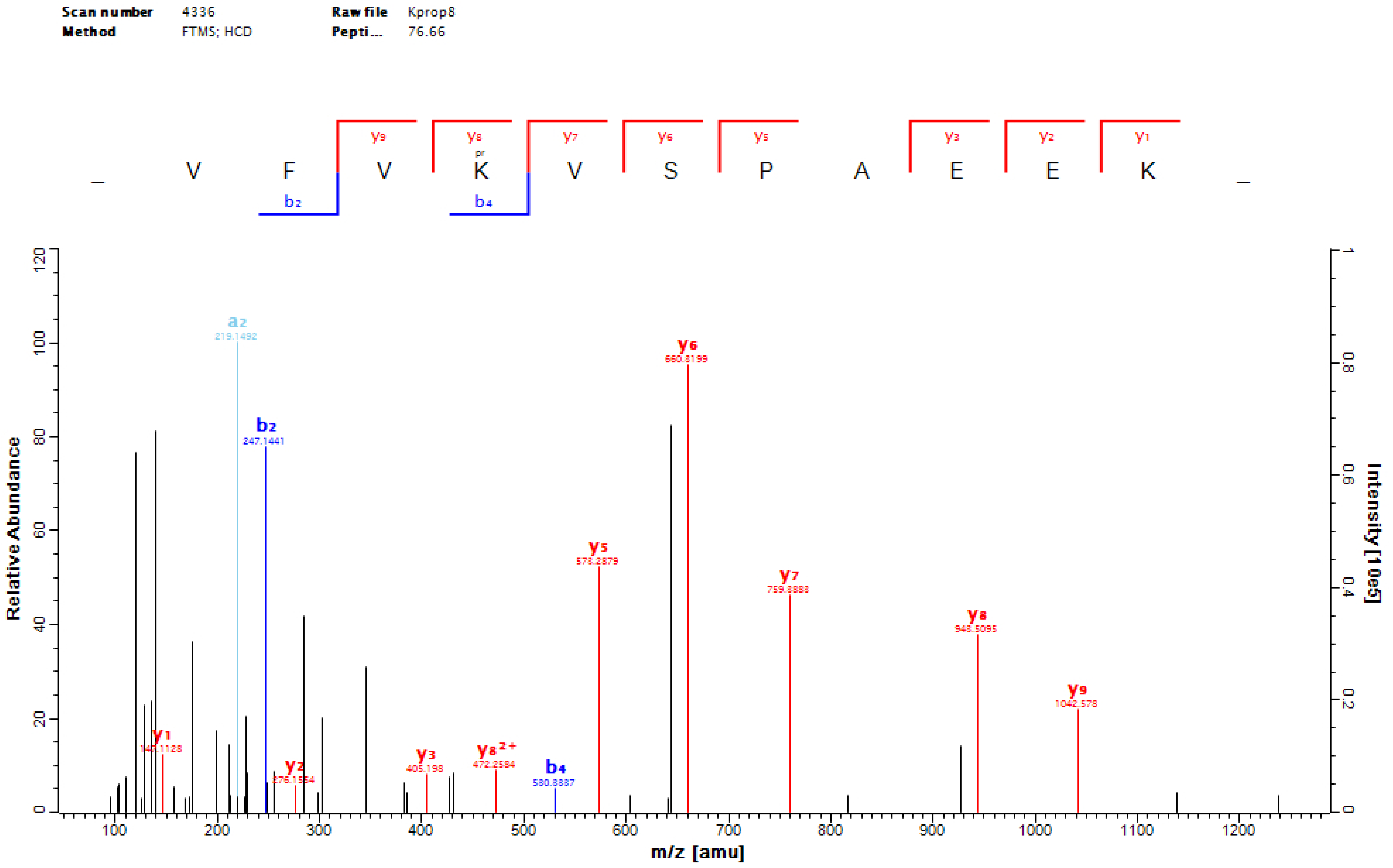

Scan number 4336
Method FTMS; HCD
Raw file Kprop8
Pepti... 76.66
_ V F V K V S P A E E K _
y9 y8 y7 y6 y5 y3 y2 y1
b2 b4
a2
219.1492
b2
247.1441
y1
y2
y3
405.198
y8 2+
472.2584
b4
y5
y6
y7
y8
y9
1042.578
Relative Abundance
Intensity [10e5]
m/z [amu]

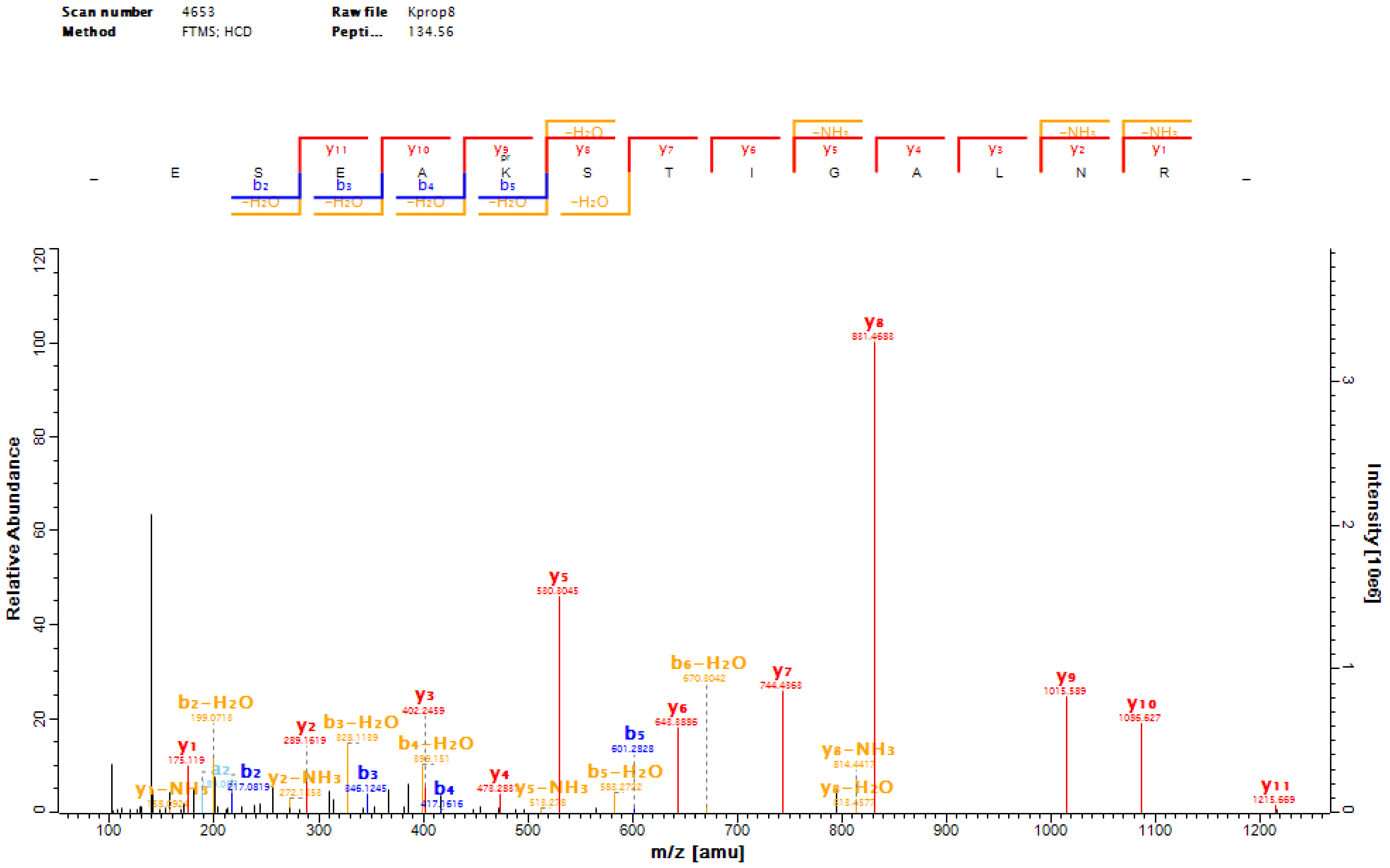

Scan number 4653
Method FTMS; HCD
Raw file Kprop8
Pepti... 134.56
_ E S E A K S T I G A L N R _
y11 y10 y9 y8 y7 y6 y5 y4 y3 y2 y1
b2 b3 b4 b5
-H2O
-NH3
Relative Abundance
Intensity [10e6]
m/z [amu]
y8 831.4683
y5 530.3045
b6-H2O 670.3042
y7 744.4363
y9 1015.589
y10 1086.627
y6 643.3886
y3 402.2459
b2-H2O 199.0713
y2 289.1619
b3-H2O 328.1139
b4-H2O 399.151
b5 601.2828
y8-NH3 814.4417
y1 175.119
b2 217.0819
b3 346.1245
b4 417.1616
y4 473.2831
b5-H2O 583.2722
y5-NH3 513.278
y8-H2O 813.4577
y2-NH3 272.1353
y1-NH3 158.0924
y11 1215.669
a2

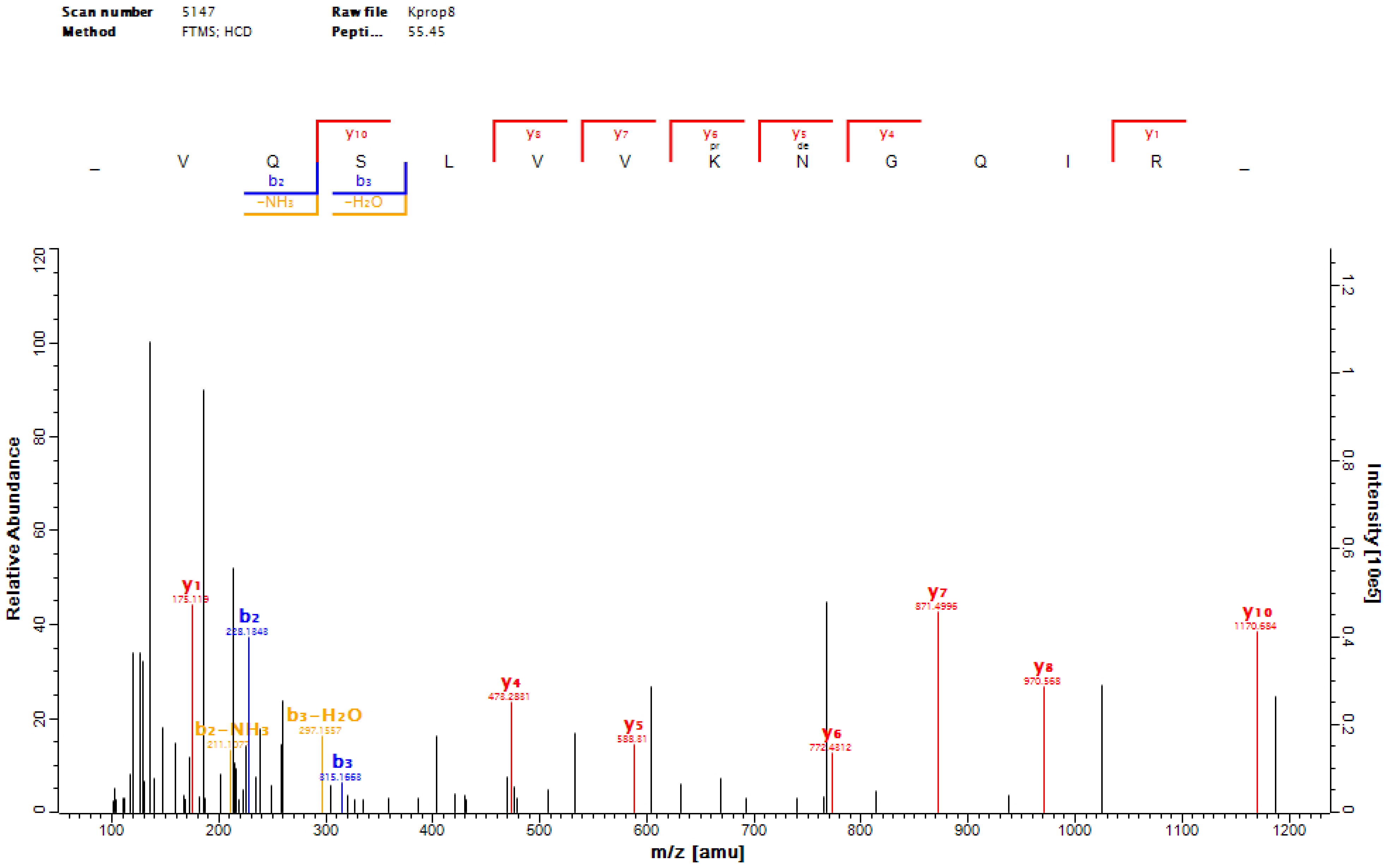
Scan number 5147
Method FTMS; HCD
Raw file Kprop8
Pepti... 55.45
_ V Q S L V V K N G Q I R _
y10 y8 y7 y6 y5 y4 y1
b2 b3
-NH3 -H2O
y1 175.119
b2 228.1343
b2-NH3 211.1077
b3-H2O 297.1557
b3 315.1663
y4 476.2831
y5 588.31
y6 772.4312
y7 871.4996
y8 970.568
y10 1170.684
Relative Abundance
Intensity [10e5]
m/z [amu]

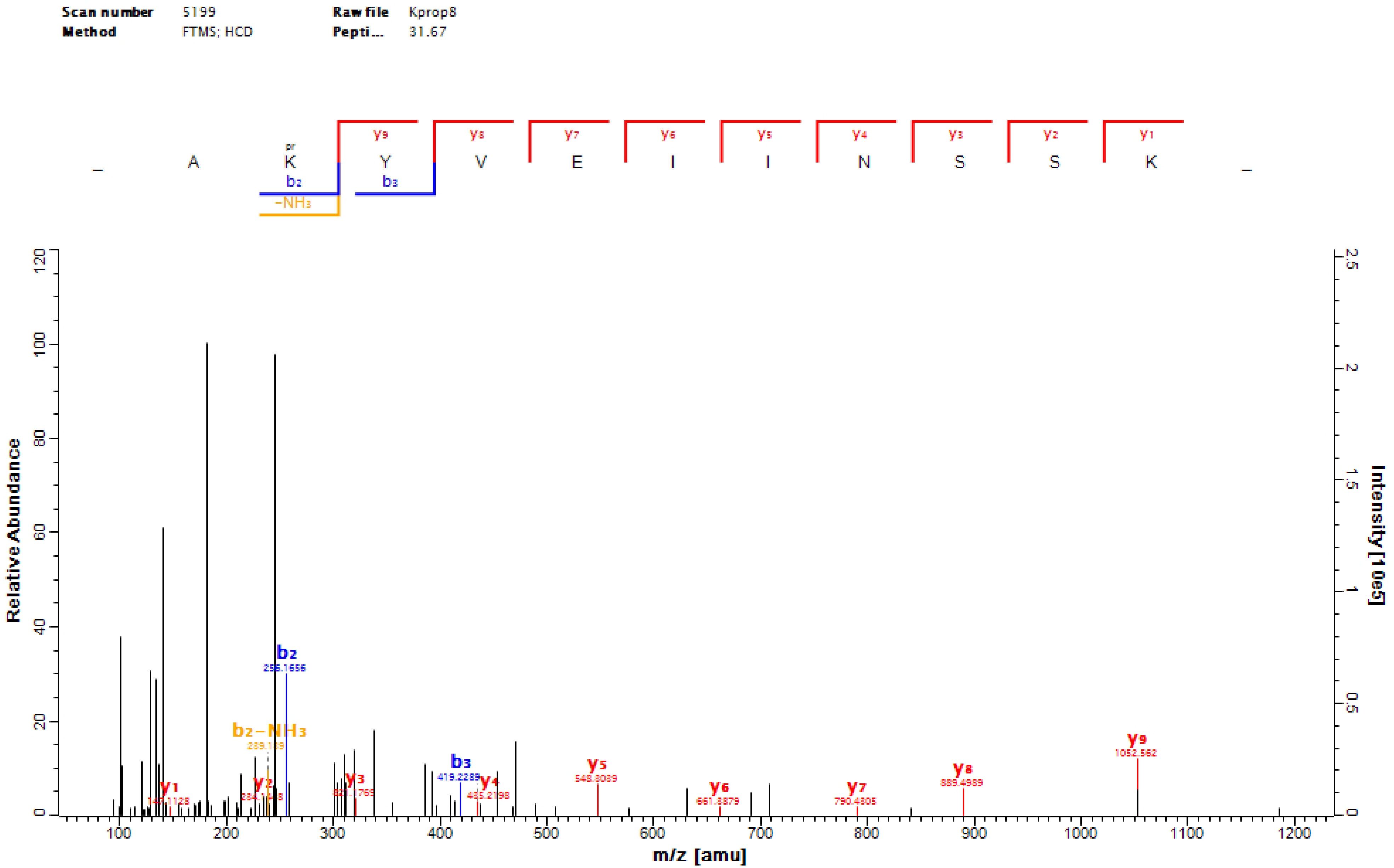

Scan number 5199
Raw file Kprop8
Method FTMS; HCD
Pepti... 31.67
_ A K Y V E I I N S S K _
b2
b3
-NH3
y9 y8 y7 y6 y5 y4 y3 y2 y1
b2 256.1656
b2-NH3 239.139
b3 419.2289
y5 548.3039
y6 661.3879
y7 790.4305
y8 889.4989
y9 1052.562
Relative Abundance
Intensity [10e5]
m/z [amu]

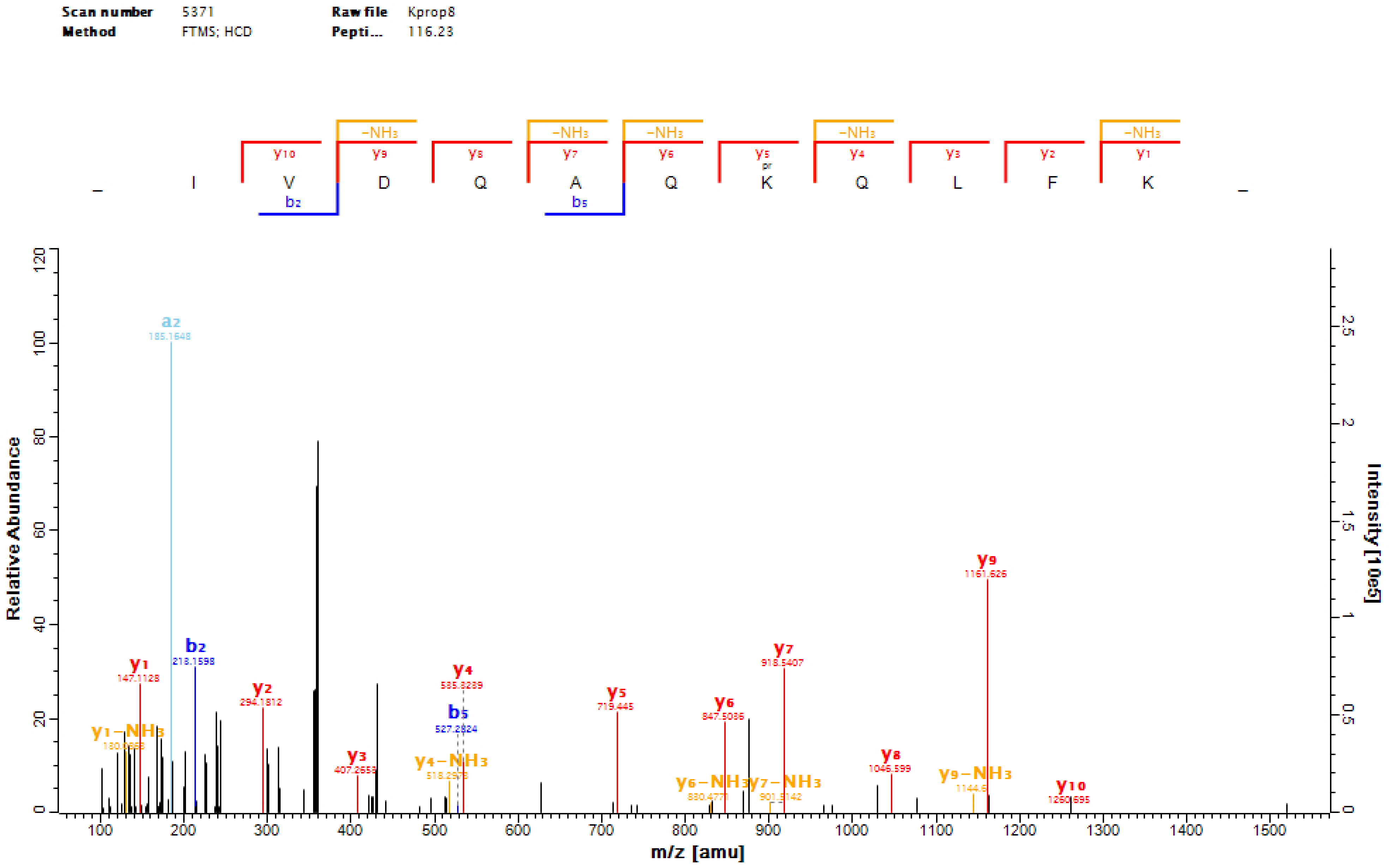
Scan number 5371
Method FTMS; HCD
Raw file Kprop8
Pepti... 116.23
_ I V D Q A Q K Q L F K _
y10 y9 y8 y7 y6 y5 y4 y3 y2 y1
-NH3
b2 b5
Relative Abundance
Intensity [10e5]
m/z [amu]
a2 185.1648
b2 218.1598
y1 147.1128
y1-NH3
y2 294.1812
y3 407.2658
y4 535.3289
y4-NH3 518.2978
b5 527.2824
y5 719.445
y6 847.5086
y6-NH3 830.4771
y7 918.5407
y7-NH3 901.5142
y8 1046.599
y9 1161.626
y9-NH3 1144.6
y10
100 200 300 400 500 600 700 800 900 1000 1100 1200 1300 1400 1500
0 20 40 60 80 100 120
0 0.5 1 1.5 2 2.5

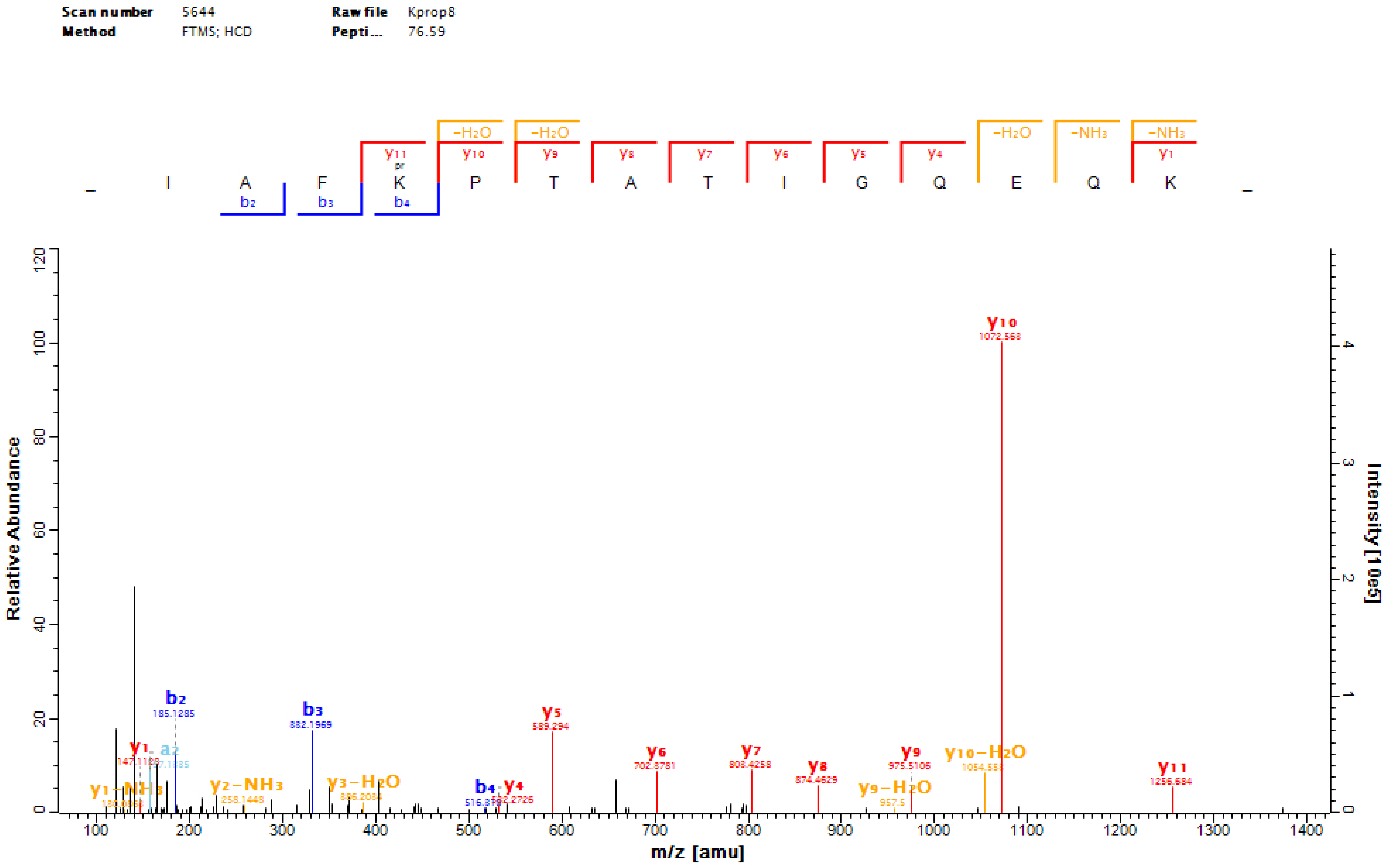

Scan number 5644
Raw file Kprop8
Method FTMS; HCD
Pepti... 76.59
_ I A F K P T A T I G Q E Q K _
b2 b3 b4
y11 y10 y9 y8 y7 y6 y5 y4 y1
-H2O -H2O -H2O -NH3 -NH3
Relative Abundance
Intensity [10e5]
m/z [amu]
y10 1072.563
b2 185.1285
b3 332.1969
y5 589.294
y6 702.3781
y7 803.4258
y8 874.4629
y9 975.5106
y10-H2O 1054.553
y11 1256.684
y9-H2O 957.5
y1 147.1128
a2
y1-NH3
y2-NH3 258.1448
y3-H2O 386.2034
b4 516.378
y4 532.2726

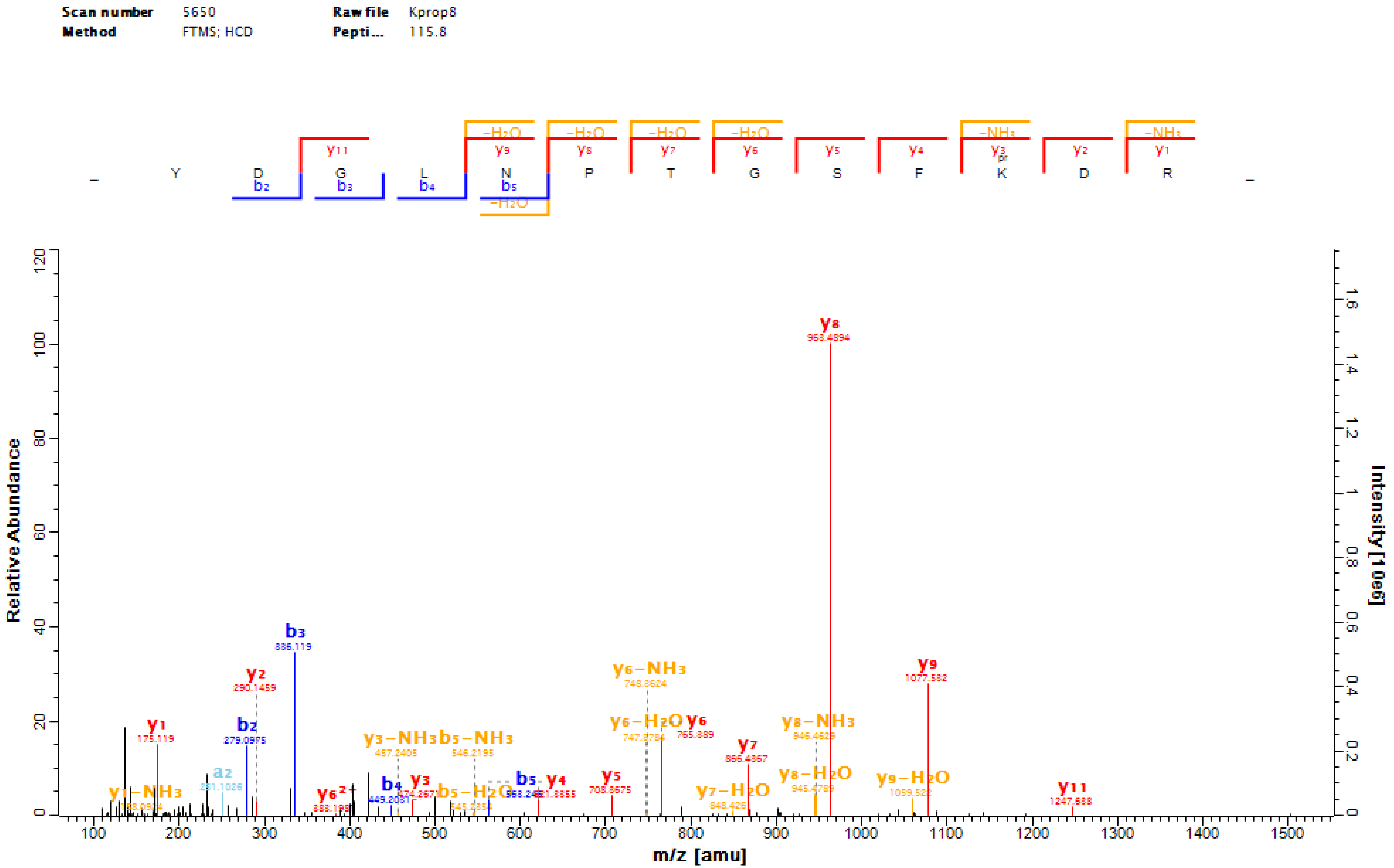

Scan number 5650
Raw file Kprop8
Method FTMS; HCD
Pepti... 115.8
Y D G L N P T G S F K D R
y11 y9 y8 y7 y6 y5 y4 y3 y2 y1
b2 b3 b4 b5
y8
963.4894
y9
1077.582
b3
336.119
y2
290.1459
b2
279.0975
y1
175.119
a2
y6-NH3
748.3624
y6-H2O
747.8784
y6
765.889
y7
866.4367
y8-NH3
946.4629
y8-H2O
y9-H2O
y7-H2O
y5
708.3675
y4
y3
b4
b5
y3-NH3
457.2405
b5-NH3
546.2195
b5-H2O
y1-NH3
y6 2+
y11
1247.688
Relative Abundance
Intensity [10e6]
m/z [amu]

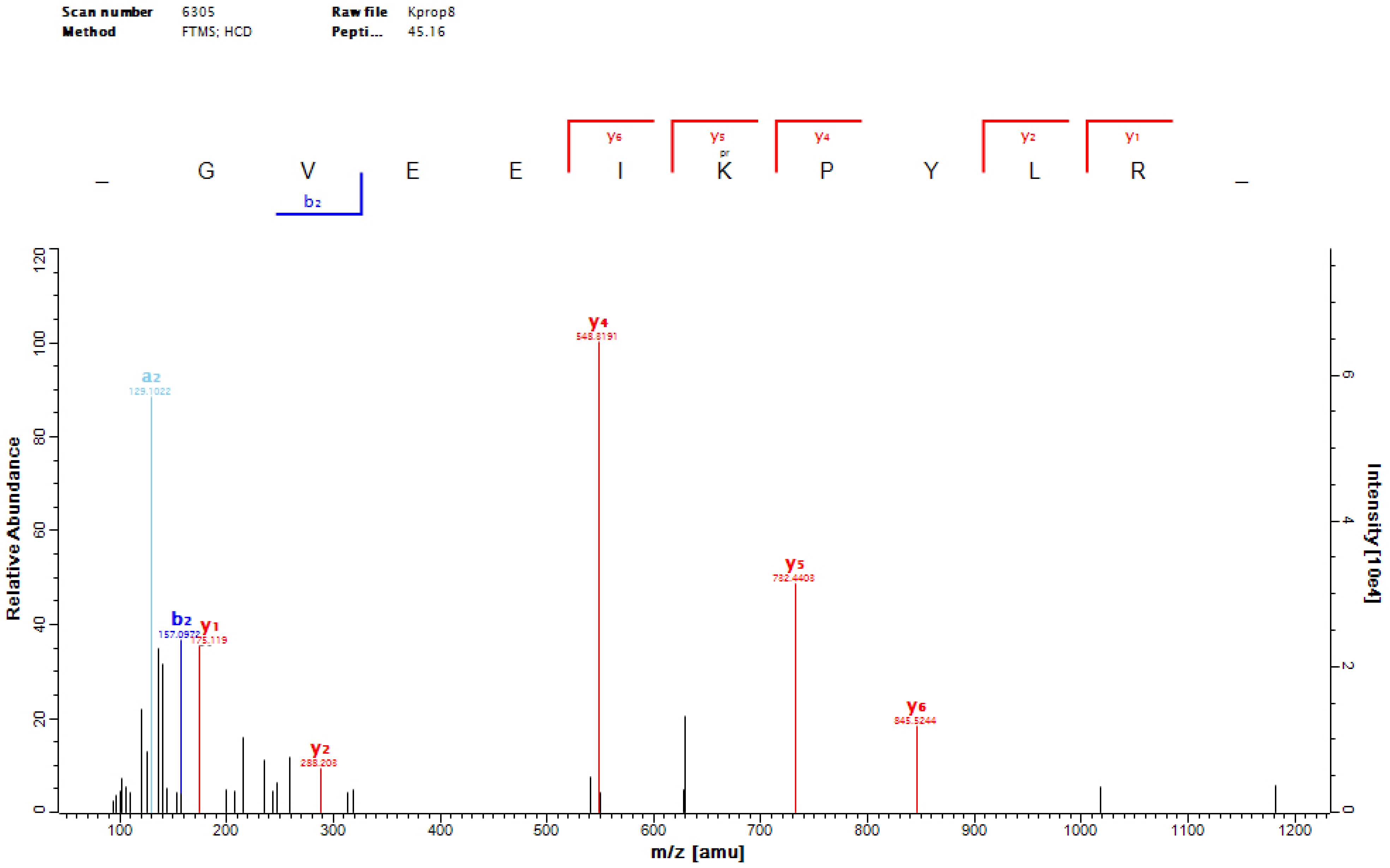
Scan number 6305
Raw file Kprop8
Method FTMS; HCD
Pepti... 45.16
_ G V E E I K P Y L R _
y6 y5 y4 y2 y1
b2
a2 129.1022
b2 157.0972
y1 175.119
y2 288.203
y4 548.8191
y5 732.4403
y6 845.5244
Relative Abundance
Intensity [10e4]
m/z [amu]

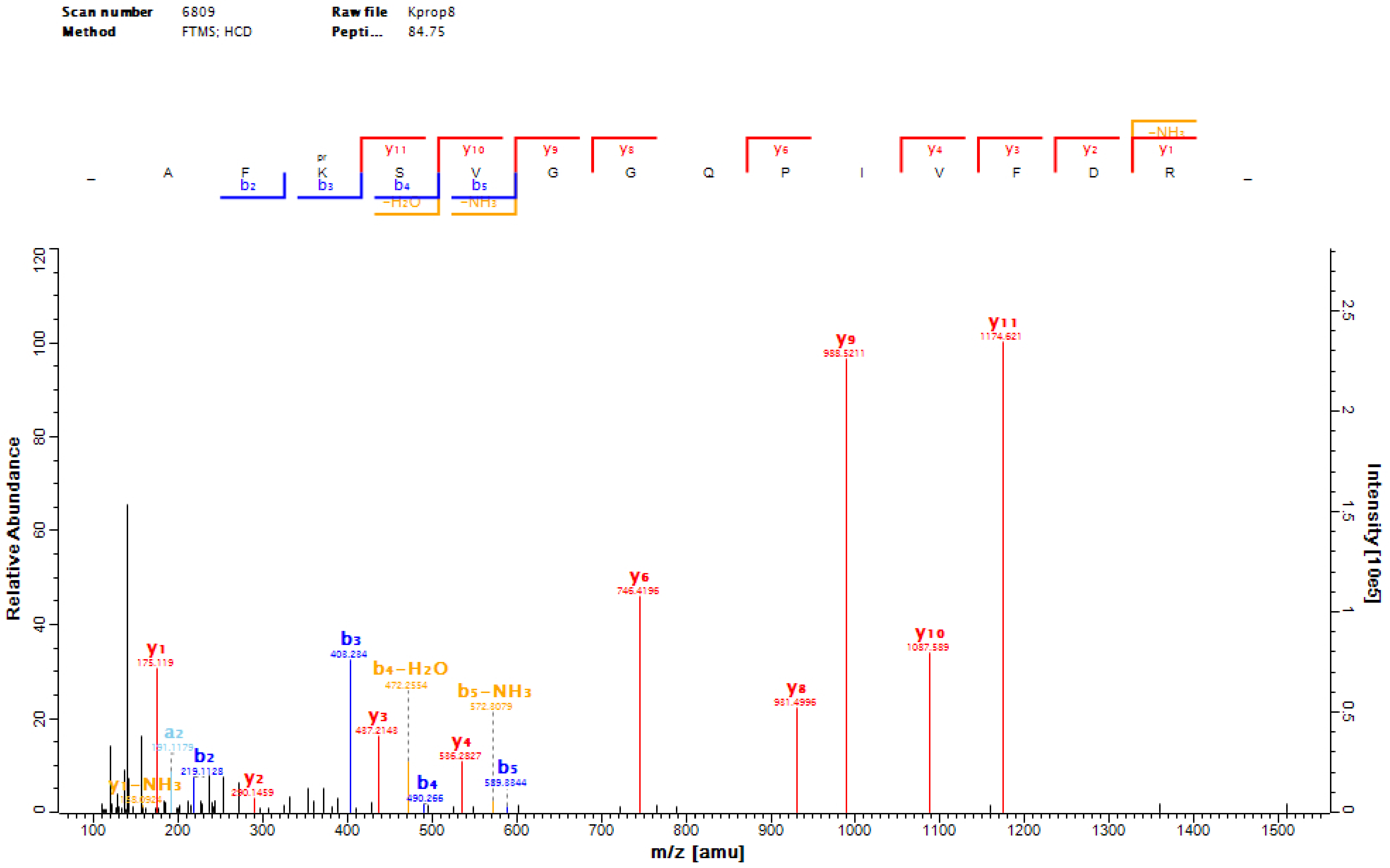
Scan number 6809
Method FTMS; HCD
Raw file Kprop8
Pepti... 84.75
_ A F pr K S V G G Q P I V F D R _
b2 b3 b4 b5
-H2O -NH3
y11 y10 y9 y8 y6 y4 y3 y2 y1
-NH3
Relative Abundance
Intensity [10e5]
m/z [amu]
y1 175.119
y1-NH3
a2
b2 219.1128
y2
b3 403.284
b4-H2O 472.2554
y3 437.2148
b4 490.266
b5-NH3 572.3079
y4 536.2827
b5 589.3344
y6 746.4196
y8 931.4996
y9 988.5211
y10 1087.589
y11 1174.621

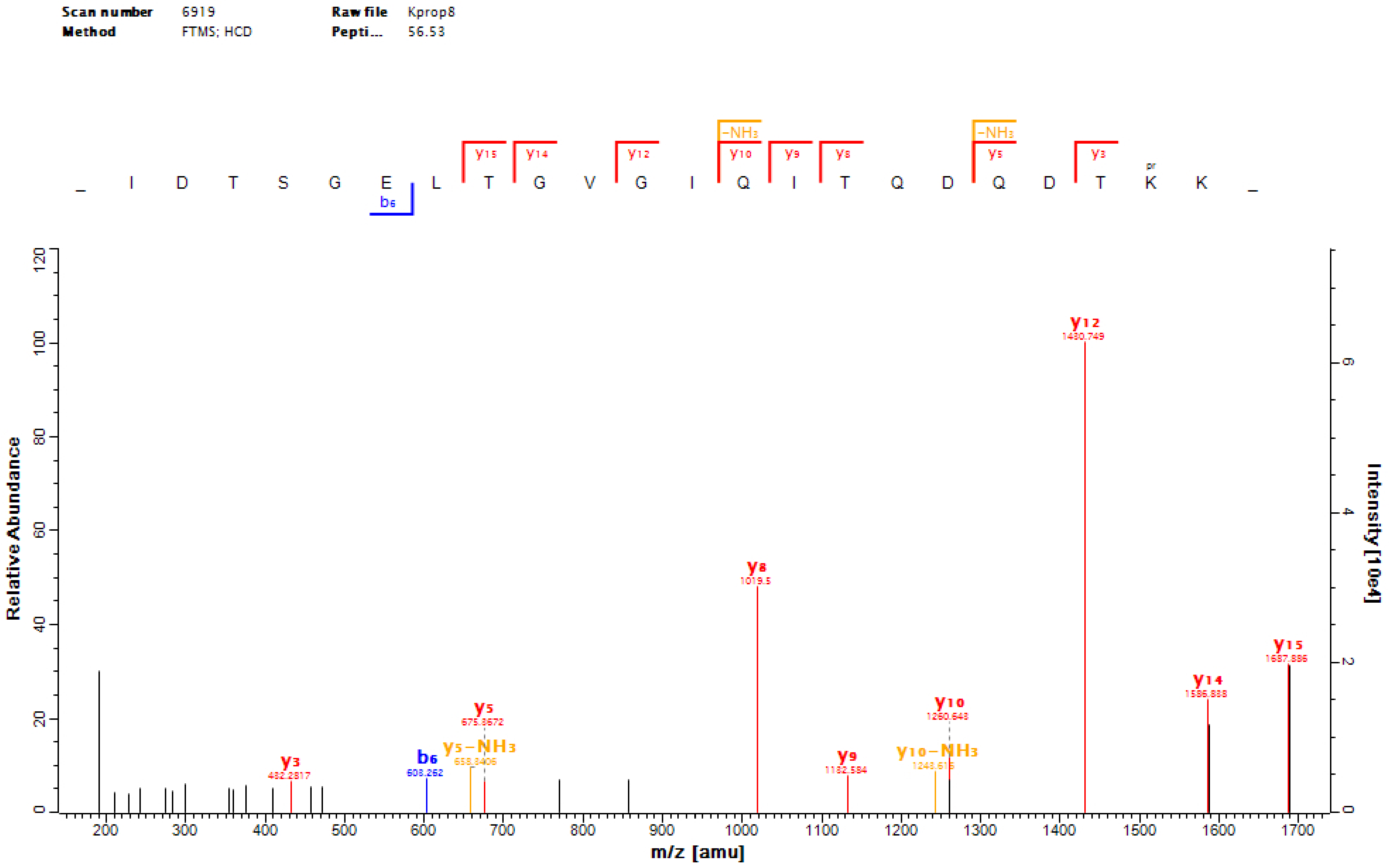
Scan number 6919
Raw file Kprop8
Method FTMS; HCD
Pepti... 56.53
_ I D T S G E L T G V G I Q I T Q D Q D T K K _
y15 y14 y12 y10 y9 y8 y5 y3
-NH3
b6
pr
Relative Abundance
Intensity [10e4]
m/z [amu]
y12
1430.749
y8
1019.5
y15
1687.886
y14
y10
1260.643
y10-NH3
1243.616
y9
y5
675.3672
y5-NH3
658.3406
b6
603.262
y3

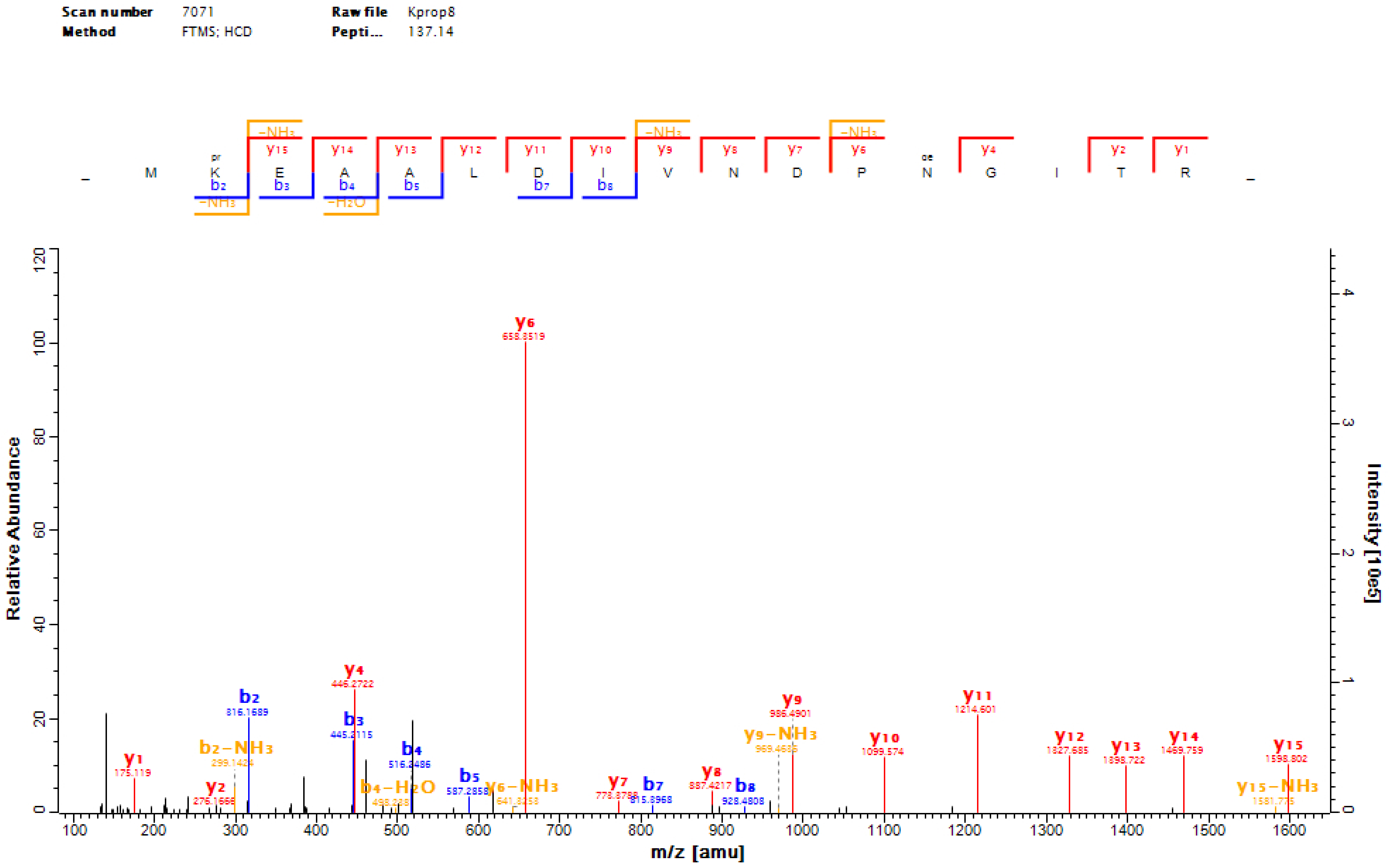

Scan number 7071
Raw file Kprop8
Method FTMS; HCD
Pepti... 137.14
_ M K E A A L D I V N D P N G I T R _
y15 y14 y13 y12 y11 y10 y9 y8 y7 y6 y4 y2 y1
b2 b3 b4 b5 b7 b8
-NH3
-H2O
Relative Abundance
Intensity [10e5]
m/z [amu]
y6 658.3519
y4 446.2722
b2 316.1689
b3 445.2115
b4 516.2486
b5 587.2858
y1 175.119
y2 276.1666
b2-NH3 299.1424
b4-H2O
y6-NH3
y7
b7
y8 887.4217
b8 928.4808
y9 986.4901
y9-NH3
y10 1099.574
y11 1214.601
y12 1327.685
y13 1398.722
y14 1469.759
y15 1598.802
y15-NH3

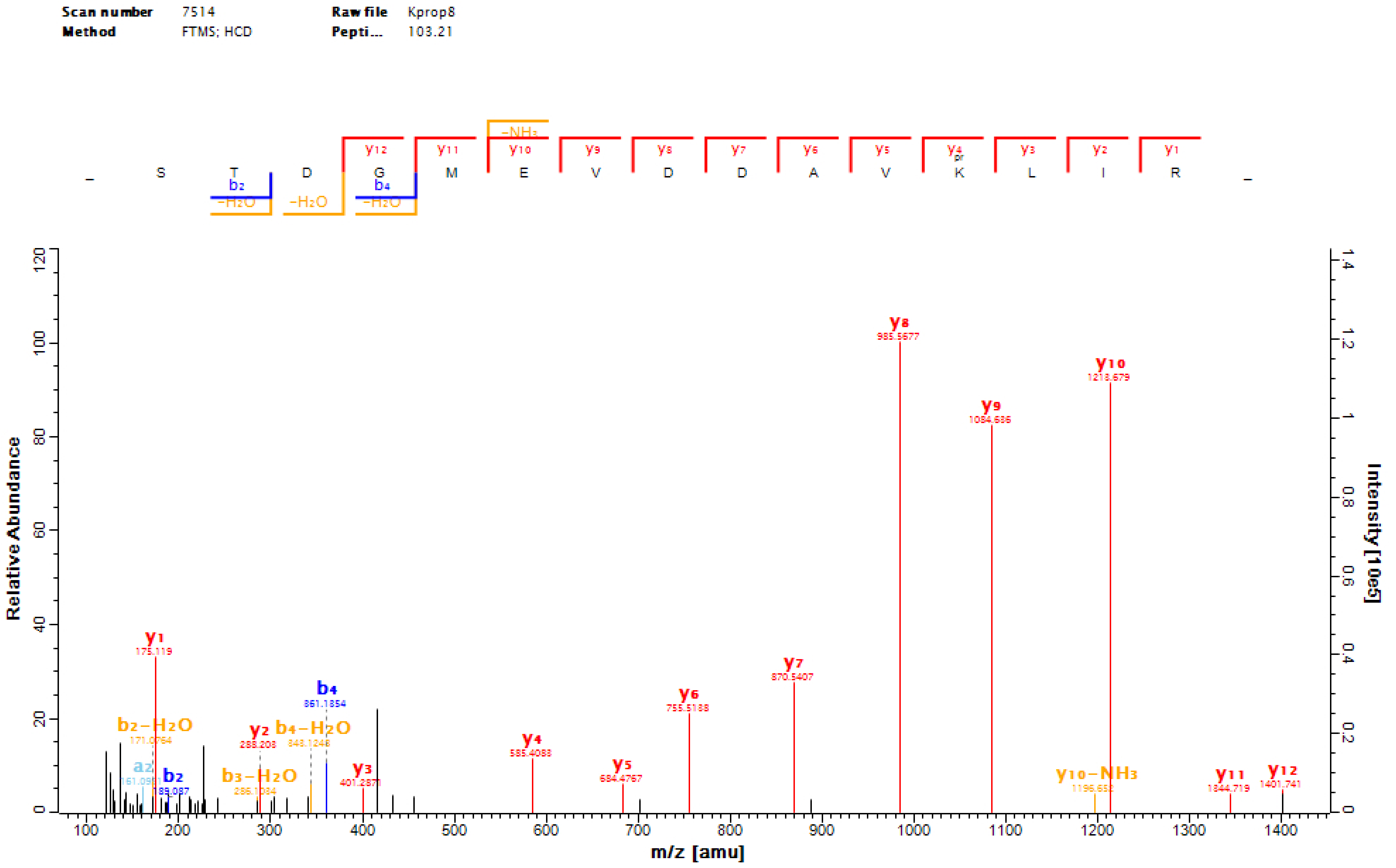

Scan number 7514
Raw file Kprop8
Method FTMS; HCD
Pepti... 103.21
_ S T D G M E V D D A V K L I R _
Relative Abundance
Intensity [10e5]
m/z [amu]
y1 175.119
y8 985.5677
y9 1084.686
y10 1213.679
y7 870.5407
y6 755.5188
y4 585.4088
y5 684.4767
y2 288.208
y3 401.2871
b4 361.1854
b2 189.087
y11 1344.719
y12 1401.741

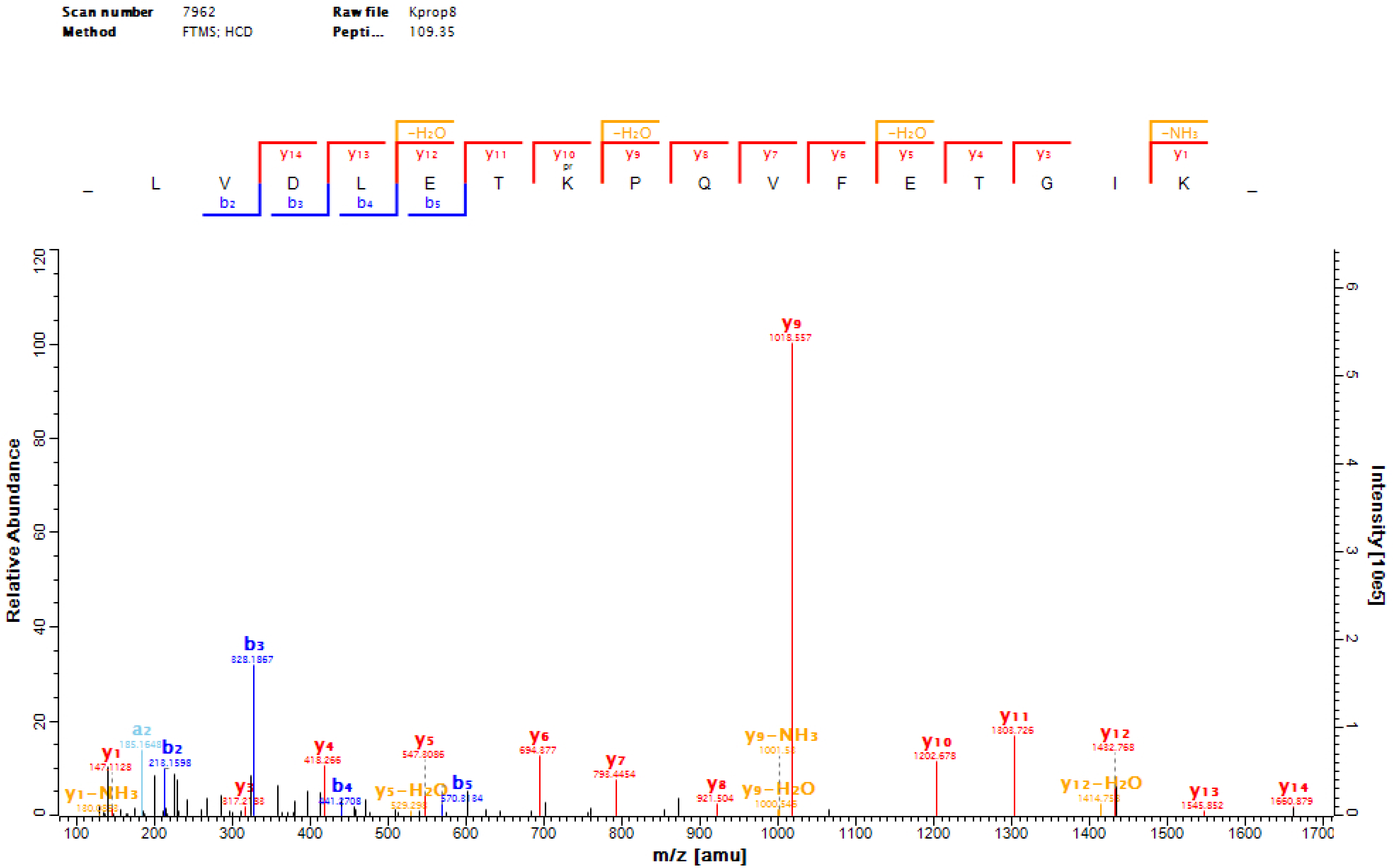

Scan number 7962
Method FTMS; HCD
Raw file Kprop8
Pepti... 109.35
_ L V D L E T K P Q V F E T G I K _
Relative Abundance
Intensity [10e5]
m/z [amu]
y9 1018.557
b3 328.1867
a2 185.1648
b2 213.1598
y1 147.1128
y1−NH3
y3
b4
y4 418.266
y5 547.3086
y5−H2O
b5
y6 694.377
y7 793.4454
y8 921.504
y9−NH3
y9−H2O
y10 1202.678
y11 1303.726
y12 1432.768
y12−H2O
y13 1545.852
y14 1660.879

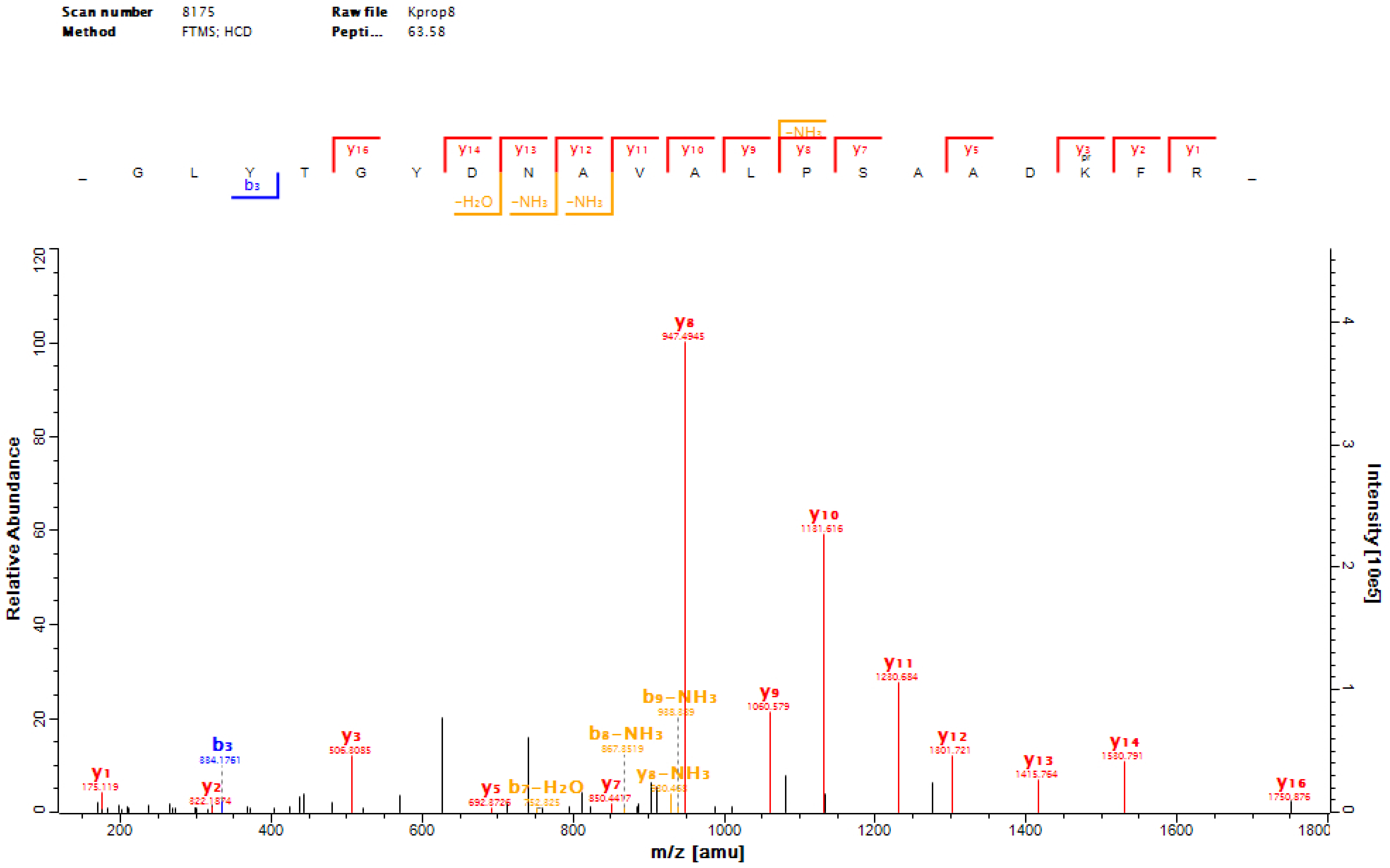

Scan number 8175
Raw file Kprop8
Method FTMS; HCD
Pepti... 63.58
_ G L Y T G Y D N A V A L P S A A D K F R _
y16 y14 y13 y12 y11 y10 y9 y8 y7 y5 y3 y2 y1
b3
-H2O -NH3 -NH3 -NH3
Relative Abundance
Intensity [10e5]
m/z [amu]
y8
947.4945
y10
1131.616
y11
1230.684
y9
1060.579
b9-NH3
b8-NH3
y8-NH3
b7-H2O
y1
175.119
b3
y2
y3
y5
y7
y12
y13
y14
y16

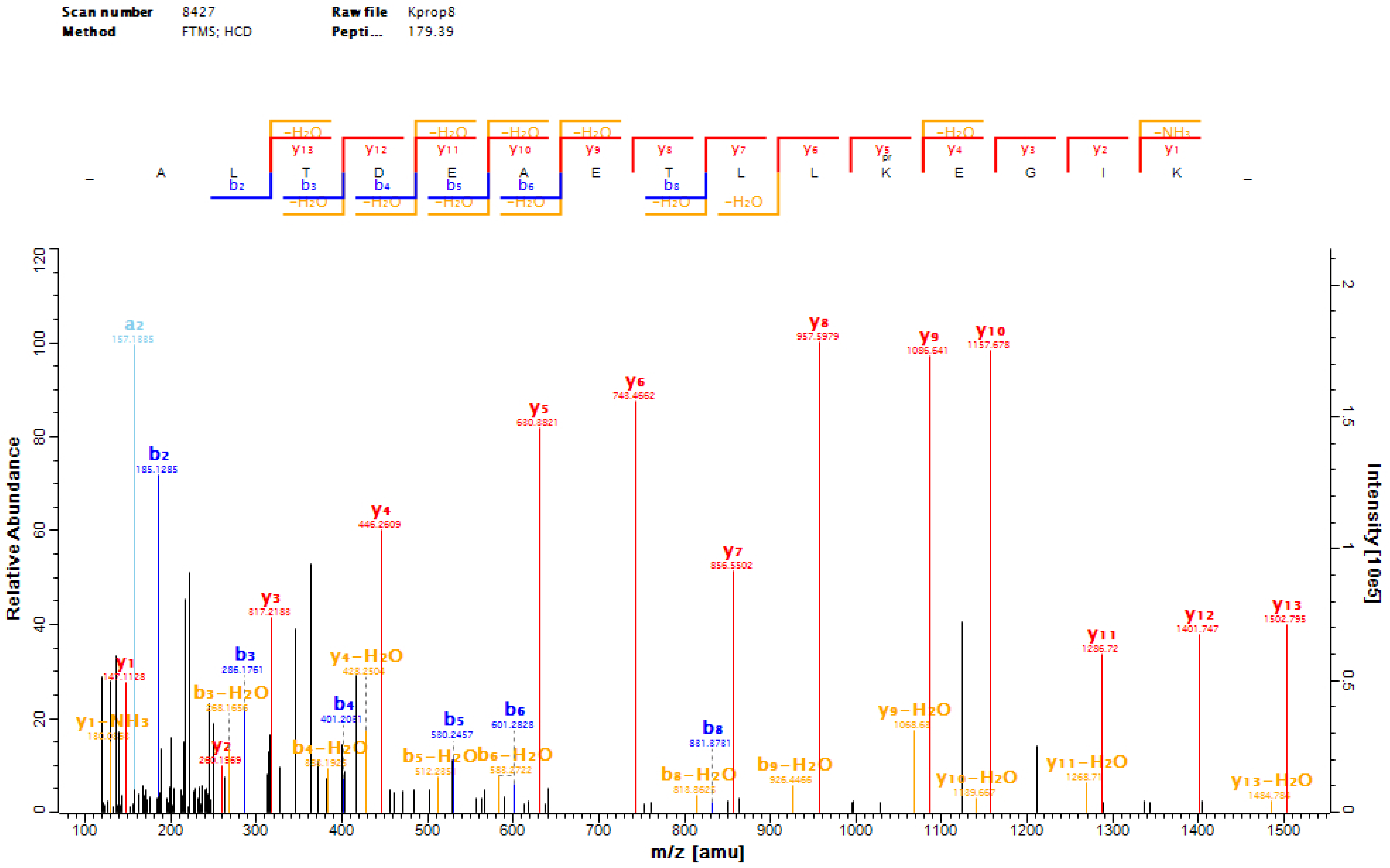
Scan number 8427
Method FTMS; HCD
Raw file Kprop8
Pepti... 179.39
A L T D E A E T L L K E G I K
Relative Abundance
Intensity [10e5]
m/z [amu]
a2 157.1335
b2 185.1285
y1 147.1128
y1−NH3
b3 286.1761
b3−H2O 268.1656
y2
y3 317.2183
y4 446.2609
y4−H2O 428.2504
b4 401.2081
b4−H2O
b5 530.2457
b5−H2O 512.235
b6 601.2828
b6−H2O 583.2722
y5 630.3821
y6 748.4662
y7 856.5502
y8 957.5979
b8 831.3781
b8−H2O 813.3626
b9−H2O 926.4466
y9 1086.641
y9−H2O 1068.63
y10 1157.678
y10−H2O 1139.667
y11 1286.72
y11−H2O 1268.71
y12 1401.747
y13 1502.795
y13−H2O 1484.784

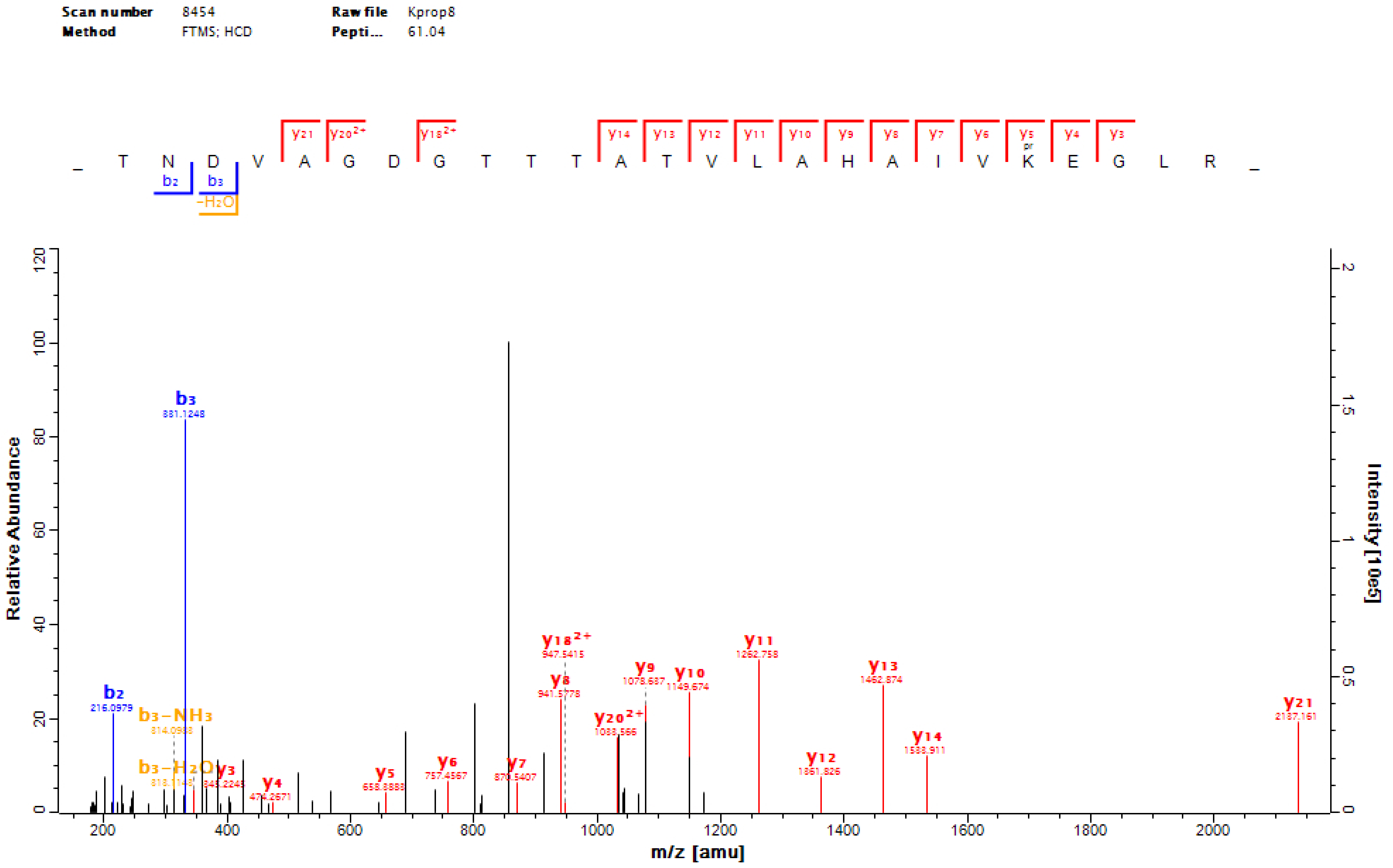

Scan number 8454
Raw file Kprop8
Method FTMS; HCD
Pepti... 61.04
_ T N D V A G D G T T T A T V L A H A I V K E G L R _
b2
b3
-H2O
y21 y20 2+ y18 2+ y14 y13 y12 y11 y10 y9 y8 y7 y6 y5 y4 y3
b3
b2
216.0979
b3-NH3
b3-H2O
y3
y4
y5
y6
757.4567
y7
870.5407
y8
941.5778
y18 2+
947.5415
y20 2+
y9
y10
1149.674
y11
1262.758
y12
1361.826
y13
1462.874
y14
1533.911
y21
2137.161
Relative Abundance
Intensity [10e5]
m/z [amu]

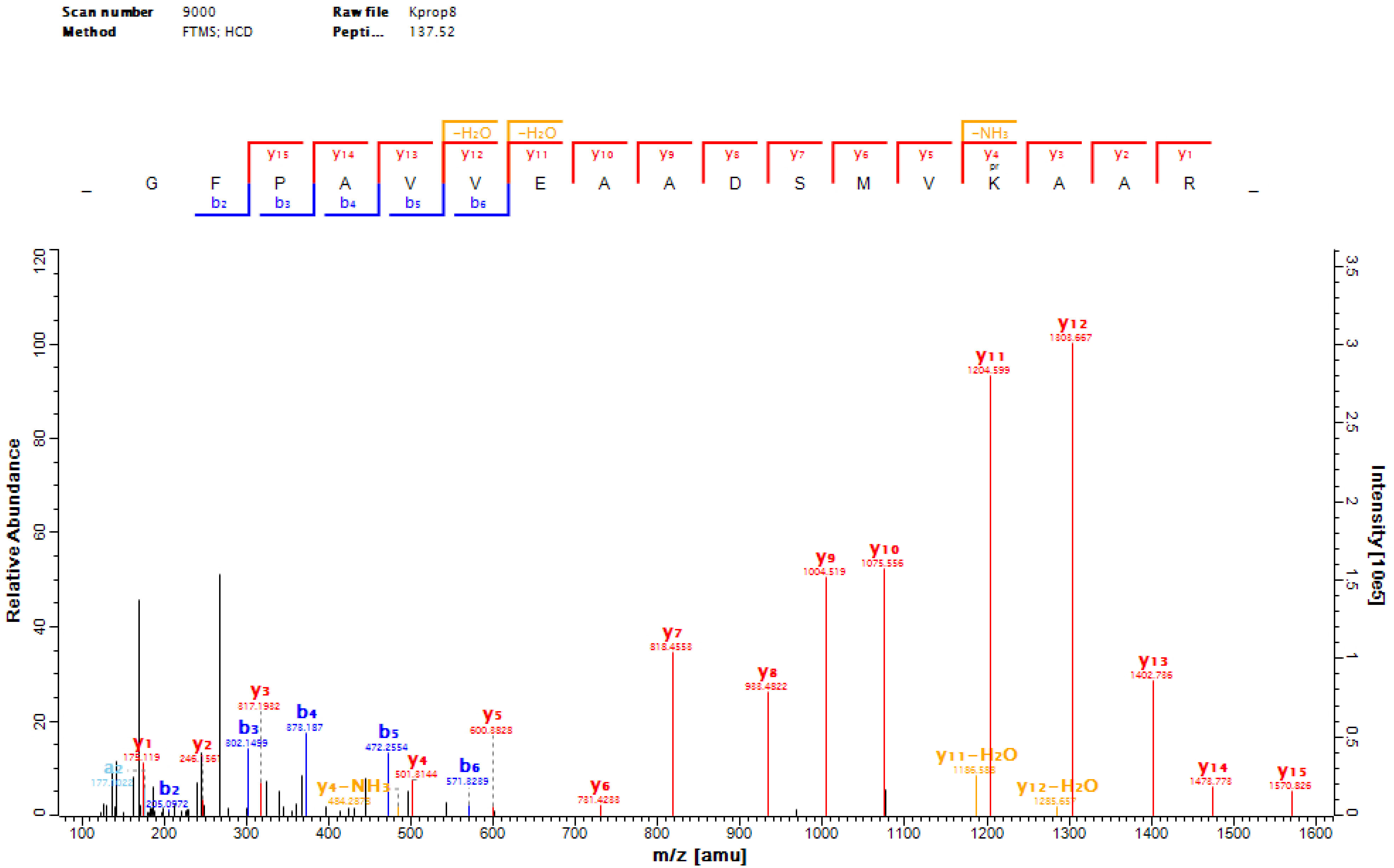

Scan number 9000
Method FTMS; HCD
Raw file Kprop8
Pepti... 137.52
_ G F P A V V E A A D S M V K A A R _
Relative Abundance
Intensity [10e5]
m/z [amu]
y1
y2
y3
b2
b3
b4
b5
b6
y4
y5
y6
y7
y8
y9
y10
y11
y12
y13
y14
y15
y4−NH3
y11−H2O
y12−H2O
a2
