# Supplementary material for: Lysine Propionylation is a Widespread Post-Translational Modification Involved in Regulation of Photosynthesis and Metabolism in Cyanobacteria
Source: Int J Mol Sci. 2019 Sep 26;20(19):4792. doi: 10.3390/ijms20194792 (PMC6801645; doi:10.3390/ijms20194792)
Supplement: Supplementary file 1 [file ijms-20-04792-s001.zip › SI/SI-R1.doc]

**Supplementary Figures and Legends**

**Figure S1.** Overview of the proteomics results. (A) Distribution of peptide score and mass error. (B) Histogram depicting the number of propionylation sites identified per protein.

**Figure S2.** Distribution of propionylated proteins based on their biological processes, molecular functions and cellular localization.

**Figure S3.** Interaction network of identified propionylated proteins. Identified propionylated proteins were highlighted in yellow.

**Figure S4.** Profiling of identified propionylation sites on FbpI. (A)The representation of the corresponding MS/MS spectra assigned to the propionylated peptides from Fbp. A comprehensive series of b and y fragment ions were annotated. (B) Multiple sequence alignment of FbpI from different species. The conserved propionylation sites were marked by the red arrow.

**Figure S5.** Mutations of FbpI were verified by DNA sequencing and MS searches. (A) Site-directed mutations were verified by DNA sequencing. Propionylated or non-propionylated peptides of FbpI in the wild type andmutants were further confirmed by MS analysis. (B) K156 and K336 propionylation sites in wild type. (C) K156R mutation site and K336 propionylation site in K156R mutant. (D) K156 propionylation site and K336R mutation site in K336R mutant.

**Figure S6.** Identification of propionylation sites on PsaD and immunoblotting analysis of propionylation level at lysine 132 on PsaD. MS/MS spectra assigned to the propionylated peptides of VYPSGEVQYLHPADGVFPEK(pr)VNEGR (A), IGQNPEPVTIK(pr)FSGK (B) and IQDYK(pr)IYR (C) from photosystem I subunit II (PsaD). (D) Immunoblotting analysis of propionylation level of PsaD in *Synechocystis* after exposure to various stresses for 12h and 24h. NC: normal condition, –N: nitrogen deficiency, +Glu: glucose condition, +NaCl: sodium chloride, Heat: 42°Ctreatment, -Fe: iron deficiency, -Cu: copper deficiency.
